# Sedimentary geology of the middle Carboniferous of the Donbas region (Dniepr-Donets basin, Ukraine)

**DOI:** 10.1038/srep09099

**Published:** 2015-03-20

**Authors:** Douwe J. J. van Hinsbergen, Hemmo A. Abels, Wolter Bosch, Flora Boekhout, Alexander Kitchka, Maartje Hamers, Douwe G. van der Meer, Mark Geluk, Randell A. Stephenson

**Affiliations:** 1 Department of Earth Sciences, University of Utrecht, Budapestlaan 4, 3584 CD Utrecht, the Netherlands; 2 Institut für Geologie und Paläontologie, Westfälische Wilhelms-Universität, Corrensstrasse 24, 48149 Münster; 3 Nexen Petroleum UK Ltd., Charter Place, Vine Street, Uxbridge, Middlesex, UB8 1JG, United Kingdom; 4 SE NaukaNaftogaz Res. Inst., NJSC Naftogaz of Ukraine, 8 Kyivska St., 08132 Vyshneve, Ukraine; 5 Shell International Exploration and Production B.V., Kessler Park 1, 2288 GS Rijswijk, the Netherlands; 6 School of Geosciences, Meston Building, King's College, University of Aberdeen, Aberdeen AB24 3UE, UK

## Abstract

The Paleozoic Dniepr-Donets Basin in Belarus, Ukraine, and Russia forms a major hydrocarbon province. Although well- and seismic data have established a 20 km thick stratigraphy, field-studies of its sediments are scarce. The inverted Donbas segment (Ukraine) exposes the middle Carboniferous part of the basin's stratigraphy. Here, we provide detailed sedimentological data from 13 sections that cover 1.5 of the total of 5 km of the Bashkirian and Moscovian stages and assess the paleoenvironment and paleo-current directions. Middle Carboniferous deposition occurred in a shelf environment, with coal deposition, subordinate fluvial facies, and abundant lower and middle shoreface facies, comprising an intercalated package of potential source and reservoir rocks. Sedimentary facies indicate a paleodepth range from below storm wave base to near-coastal swamp environments. Sedimentation and subsidence were hence in pace, with subtle facies changes likely representing relative sea-level changes. Paleocurrent directions are remarkably consistently southeastward in time and space in the different sedimentary facies across the Donbas Fold Belt, illustrating a dominant sedimentary infill along the basin axis, with little basin margin influence. This suggests that the middle Carboniferous stratigraphy of the Dniepr-Donets basin to the northwest probably contains significant amounts of fluvial sandstones, important for assessing hydrocarbon reservoir potential.

## Introduction

**Figure 1:**
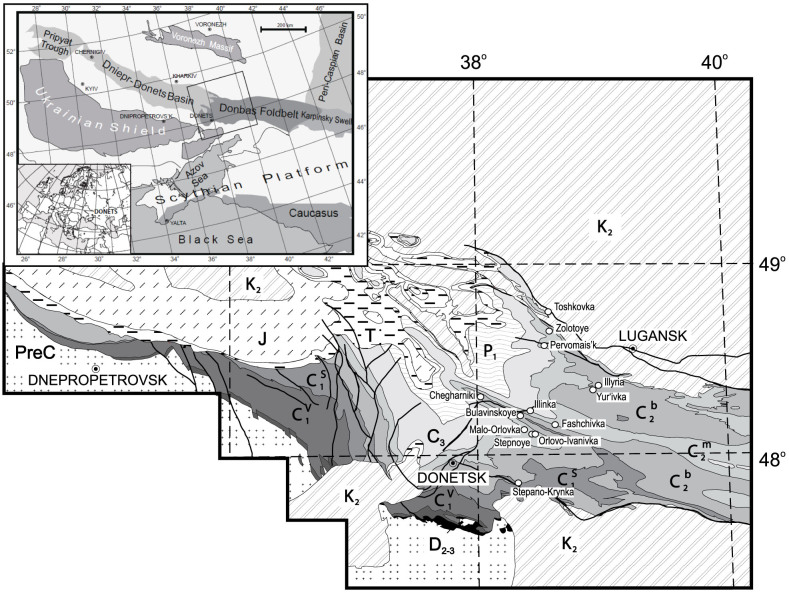
Geological map of the Donbas Foldbelt, and in the inset, the location of the Donbas in the regional East European structural framework (modified from Stovba and Stephenson^31^).

The Dniepr-Donets Basin (DDB) forms a major hydrocarbon and coal basin in Eastern Europe with significant commercial significance^1^,^2^,^3^,^4^,^5^,^6^,^7^, forming a intra-cratonic, deep rift with up to 22 km of sediment^1^,^8^ that underwent its main basin fill history between the late Devonian and Permian^9^,^10^,^11^. It is located between the Ukrainian Shield to the south and the Voronezh Massif to the north, in the southwest of the East European Craton (EEC; Fig. 1). The basin's infill is poorly exposed, and knowledge about its sedimentary evolution largely relies on subsurface data. The eastern part of the basin, however, has been inverted in Permian and younger times and forms the Donbas Foldbelt, where Devonian and younger (volcano-) sedimentary rocks of the DDB are exposed^12^,^13^.

The Donbas Foldbelt is characterised by WNW-ESE trending long-wavelength folds and faults^14^,^15^ (Fig. 1). As a result of Permian and/or Late Cretaceous/Paleogene inversion of the Donbas Foldbelt^16^,^17^,^18^,^19^, Carboniferous sediments are exposed. The middle Carboniferous is exposed within the axial zone of the Donbas Foldbelt as well as near the southern margin, where it overlies pre-and syn-rift Devonian to Lower Carboniferous sediments. Outcrops in the Donbas Foldbelt are mainly confined to road and river sections and quarries. We present detailed sedimentological descriptions and paleoenvironmental interpretations from 13 sections covering 1.5 km of a total of ~5 km Bashkirian and Moscovian stratigraphy. We briefly discuss the potential implications of our findings for the sedimentary geology of the DDB farther to the northwest, where exposures of the middle Carboniferous are absent.

### Geological setting

The DDB overlies an Archean to Lower Proterozoic crystalline basement^20^,^21^ and trends NW-SE from Belarus through Ukraine to Russia, connecting with the Karpinsky Swell to the east (Fig. 1). The Donbas segment of the DDB formed at the southern margin of the EEC, which belonged to Laurussia in the Late Paleozoic and was located at near-equatorial latitudes during the Early Carboniferous moving to ~15° northerly latitudes in the Permian^22^,^23^,^24^,^25^. Towards the southeast, the width and thickness of the basin fill, the intensity of inversion-related deformation, the degree of metamorphism of its exposed sediments, as well as the degree of syn-rift volcanic activity increase^26^. Basin inversion occurred in two or three phases in Permo-Triassic and Late Cretaceous-Early Cenozoic time^16^,^17^,^18^,^19^. Shortening of the Donbas Foldbelt may be Permian^27^,^28^,^29^,^30^ or Late Cretaceous-Early Cenozoic in age^31^,^32^. Inversion occurred along WNW-ESE rift-bounding faults, and formed a central ‘Main Anticline’^33^ flanked by gentle folds. To the north, the Donbas Foldbelt borders the Voronezh Massif (Fig. 1) along large thrusts and reverse faults; in the south the contact of the Donbas Foldbelt with the Ukrainian Shield is formed by reverse faults^8^,^32^.

Sedimentation in the DDB started in Middle Devonian time with deposition of pre-rift sediments under platform conditions. Judging from the absence of marginal facies, and based on low-temperature geochronology, that platform originally extended far beyond the present limits of the basin^34^,^35^. The main rifting phase that formed the DDB started in the Late Devonian (370–363 Ma^36^,^37^ and was associated with basement doming and mafic to intermediate magmatism^13^. Devonian rifting led to widespread salt-deposition, reflected by local diapirs in the Donbas Foldbelt^38^. Salt formations probably filled deep-water basins, and were preceded by deposition of organic-rich anoxic shales and carbonates^2^. Uppermost Devonian rocks formed large sub-aqueous clastic fans along the southern basin margin and containing shales interbedded in coarser clastics. A total thickness of 4–5 km of the syn-rift basin infill was estimated^2^.

A post-rift sag sequence is bounded by pre-Carboniferous and pre-Triassic unconformities^12^. Following the first stages of rift reactivation in the Visean, the Ukrainian shield was covered by a thin layer of upper Visean and younger sediments. A large volume of fluvial clastic material was transported by river systems from the northwest along the basin axis, prograding into a deeper water basin that existed since Devonian time^39^. Serpukhovian and younger Carboniferous sediments commonly consist of cyclothems of marine limestone or shale at the bottom to coal and paleosol beds at the top and form the focus of this study^40^.

During the Carboniferous and much of the Early Permian, the DDB gradually subsided. The rate of subsidence was high; the thickness of the Carboniferous-Lower Permian, dominantly clastic stratigraphy increases from 2–3 km in the northwest to about 11 km in the southeast of the basin. Increased aridity and relative sea level fall during the early Permian resulted in deposition of red beds, carbonates, and evaporites^16^,^41^,^42^,^43^,^44^. Upper Permian sedimentary rocks are absent and post-Permian deposits are fluvio-lacustrine to shallow marine clastics and carbonates with a maximum thickness of ~2–2.5 km^12^.

**Figure 2:**
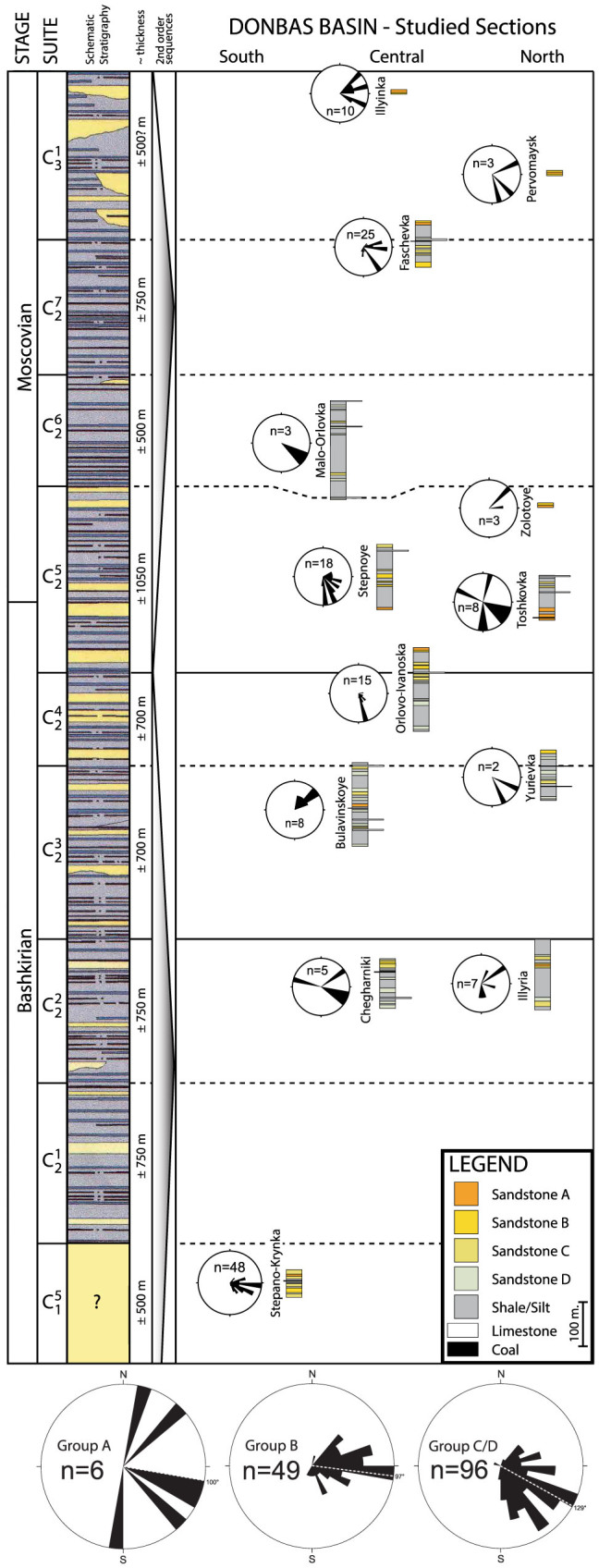
Middle Carboniferous stratigraphy showing the lithostratigraphic position of the sections studied in this paper in context eastern European stages, regional suites, marker limestones, and biozones of the Bashkirian and Moscovian stages^3^. Rose diagrams in bottom panel indicate paleocurrent directions per sandstone type showing a general E-SE paleocurrent, i.e. parallel to the basin axis.

The Carboniferous stratigraphy of the Donbas region studied here has been divided into lithostratigraphical suites^45^. Four suites are recognized in the Mississippian, nine in the Early and Middle Pennsylvanian (or Bashkirian and Moscovian; Fig. 2). The ‘middle’ Carboniferous is more than 5 km thick (~3 km Bashkirian and 2–2.5 km of Moscovian^12^,^46^,^47^). Limestone beds form regional markers in the successive suites and have been labelled with a letter from the Latin alphabet accordingly. Smaller or regionally less coherent limestone beds within suites were assigned subscript successive numbers (Fig. 2). Additional subdivision has been made on the basis of the fossil content of the succession. Biozones have been coded differently from the suites, and the boundaries do not all correspond with the suite boundaries (Fig. 2). Biostratigraphy and suites subdivision form the basis for correlation of the Donbas stratigraphy to the Western European and American stratigraphies^48^.

## Results

**Figure 3:**
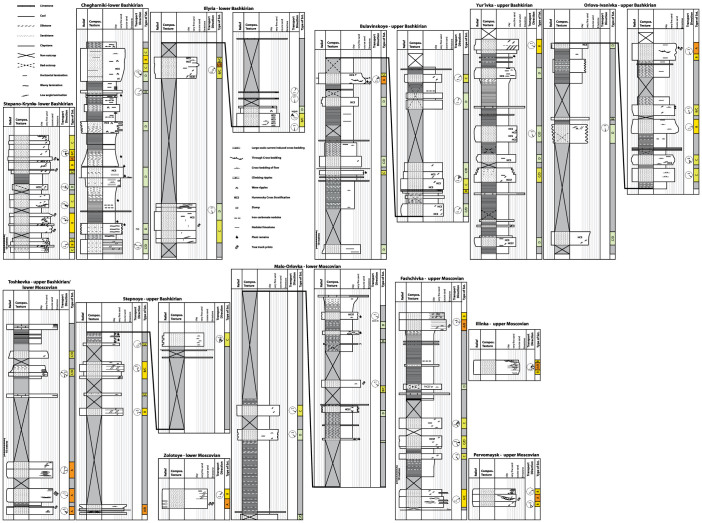
Sedimentary logs of the sections studied from middle Carboniferous stratigraphy of the Donbas region. Locations of the sections are indicated on Figure 1. See online appendix for detailed descriptions, field photographs, and extensive documentation of all sections.

Thirteen sections in the Donbas region were studied in detail (Figs. 1, 2, 3), covering over 1.5 of the total of ~5 km of middle Carboniferous stratigraphy (Fig. 2). The studied sections together represent the majority of available outcrops of Bashkirian and Moscovian stratigraphy in the area. Extensive descriptions of each section are given in the Supplementary Material, with detailed sedimentary logs, descriptions, field photographs and section locations. Here, we briefly describe the sections in stratigraphic order.

**Figure 4:**
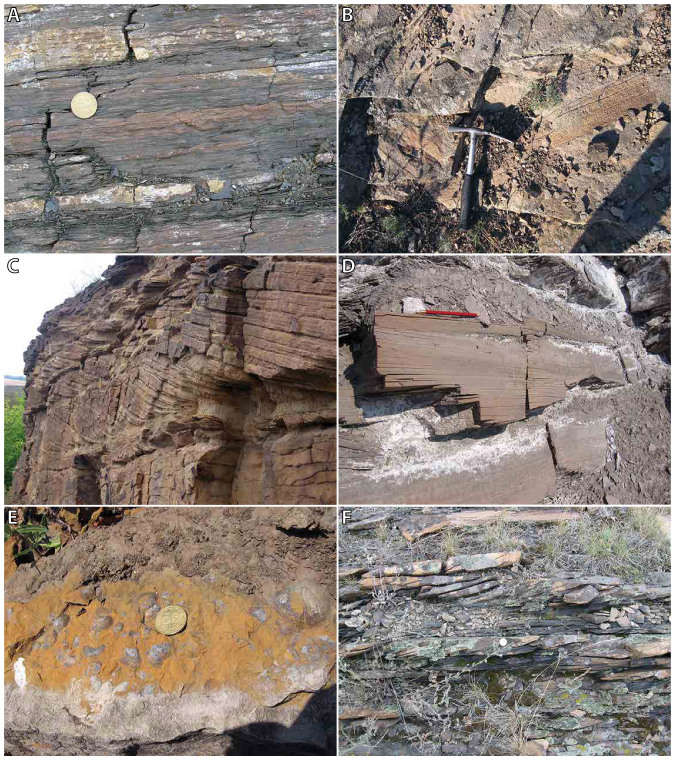
Field photographs of various characteristic lithologies (photos by H.A. Abels and W. Bosch). (A) wavey laminated very fine sand and siltstone, Chegharniki section; (B) Tree trunk prints in sandstones, Stepano-Krinka section; (C) Large-scale cross-bedding, Pervomaisk section; (D) Hummocky cross stratification, Chegharniki section; (E) Brachiopods in fine sandstones, Malo-Orlovka section; (F) Cross-bedded sandstones, Stepnoye section.

The oldest part of the stratigraphy was studied in the 65 m thick *Stepano-Krynka* section (lower Bashkirian), dominated by sandstone with subordinate clay and silt and an occasional coal bed. Most sandstones in the section are well-sorted middle sand with abundant current-induced cross-bedding. It contains intervals with tree trunk prints (Fig. 4B). One, relatively thin sandstone (SK.2A) is present that is much finer grained and contains hummocky cross stratification. Paleocurrents were east to southeast.

The *Chegharniki* section is early Bashkirian in age, and covers 118 m. The section has 39% sandstone content, with the rest shales. In the middle part of the section, sandstones are fine and well sorted (Fig. 4A), and occasionally show hummocky cross-stratification (Fig. 4D). The basal sandstone shows current-induced cross beds. The topmost thick sandstone is coarser-grained, shows larger scale foresets, and some intervals with very poor sorting. In the section plant remains are common. Sparse paleocurrent measurements suggest an eastward paleocurrent direction.

Section *Illyria* is early Bashkirian in age and is characterized by a relative high amount (73%) of poorly exposed shales and silts. The top of the section is characterised by fine to middle well-sorted sand with large scale swaley cross stratification.

The upper Bashkirian *Bulavinskoye* section exposes well-sorted fine sandstone units that contain hummocky cross-stratification or current-induced large-scale cross-beds (set height ~50 cm). Paleocurrent directions in these sandstones are consistently northeast. A few sandstones in the section are coarser grained and occasionally contain tree trunk prints, poor sorting, and massive bedding. This unit also shows some trough-like structures. Limestone beds are present in the middle of shaly intervals. One coal interval has been observed. Below the studied section, a thick interval of shales (~200 m) is present, overlying a thick sand-rich interval (~200 m), containing coarse-grained fluvial sandstones.

The sandstones from the upper Bashkirian *Yur'ivka* section are generally well sorted, display plane bed lamination, are fine to middle grained, and show hummocky cross stratification. The uppermost sandstone contains large-scale current-induced cross- bedding. Thick limestones are present within shale-dominated intervals.

The stratigraphy of the upper Bashkirian *Orlovo-Ivanivka* section is dominated by thick shale and silt intervals with well-sorted, very-fine to fine-grained sandstones at the top, and coarser sand intervals at the base. Cross-beds indicate a consistent east to southeast paleocurrent direction.

The lower part of the upper Bashkirian-lower Moscovian *Toshkovka* section is characterised by coarse-grained, poorly sorted, sometimes massive, tree trunk print-bearing sandstone units. Some large-scale trough cross stratification is present. Above this interval, a thick section of shale contains a single thick limestone bed and is overlain by fine-grained sandstones. Paleocurrent directions indicate dominant paleo-flow to the southeast. The section is topped by a thick limestone bed, overlying shales with coal layers.

The upper Bashkirian *Stepnoye* section has a low amount of sand and is dominated by poorly exposed shales. The sandstones in the section consist mainly of moderately to well-sorted, middle to fine sandstones that display small to large-scale current-induced cross-bedding (Fig. 4F). Paleocurrent measurements are south-eastward with ~120° of variation.

The lower Moscovian *Zolotoye* sandstone can be divided into two parts that show different sedimentary characteristics. The lower part shows poorly sorted, bedded, massive coarse sandstone. The upper part consists of better-sorted coarse sandstone with large-scale current-induced cross-bedding.

The lower Moscovian *Malo-Orlovka* section is contains a high amount of shales and siltstones with brachiopod-rich beds (Fig. 4E), and subordinate sandstone. Only three sandstone units are present that have a grainsize larger than very fine sand to silt.

The upper Moscovian *Fashchivka* section is characterized by prominent sandstone units intercalated in shales and silts. Half of these units are well sorted, fine to middle sandstones with current-induced intermediate scale (~25 cm) crossbeds and some indications for hummocky cross-stratification. The basal and top unit consists of poorly sorted coarse, locally massive sandstones with tree trunk prints. Several intervals contain large-scale current-induced cross-beds (~50–100 cm) with variable paleocurrent directions. These units are fining upwards, with massive intervals in the base and better-developed current-induced crossbeds towards the top. The average paleocurrent direction towards the southeast, with almost 180 degrees variability.

The upper Moscovian *Pervomaysk* sandstone is coarsening upwards towards the middle, and fining upwards towards the top of the unit. The lower fine to middle sand part is characterized by current induced cross-bedding (Fig. 4C). The middle part is much coarser grained and less well sorted. Large trough structures and tree trunk prints were observed. The top part is finer-grained and contains abundant mega- and intermediate scale current-induced crossbedding. Three measured paleocurrent directions suggest SE paleoflow. The sandstone overlies a shale-dominated stratigraphy with a limestone bed and immediately below the Pervomaysk sandstone a transition interval of sandy silts occurs.

The upper Moscovian *Illinka* sandstone is very coarse and poorly-sorted, but shows well-developed current-induced large-scale cross-bedding. The basal and top parts of the sandstone show slightly better sorting and finer material. Towards the top, trough cross-beds occur. Paleocurrent measurements show consistent ENE paleoflow with ~90° of variation. Above the sandstone, a shale interval is exposed, with one thick limestone interlayer.

### Generalised lithological characteristics and interpretation

The middle Carboniferous stratigraphy in the Donbas region is characterized by a large amount of (poorly exposed) shales and siltstones with intercalations of sandstone bodies and some relatively thin limestone beds. Shales have a slaty character but no newly grown mica has been observed showing sub-lower greenschist facies metamorphism. Hereafter, a generalized sedimentary description of the middle Carboniferous lithologies is given.

Limestone beds, generally up to several meters thick, form regionally well-traceable, basin-wide markers used for lithostratigraphic correlation in the Donbas. They are the only lithology with marine fossils^46^,^49^. The limestones in the middle Carboniferous of the Donbas area intercalate with shale intervals. Limestones are mainly black mud- to wackestones. Some contain macroscopically recognisable fossils, such as crinoids and brachiopods. Limestone beds are typically 0.5–1.5 m thick. We found rare shell prints in the abundant claystones that are interbedded with the various sandstone facies, but did not encounter calcareous fossils or ichnofossils, although these have been reported^12^. Within the claystones, thin intervals (<50 cm) occur with high organic carbon content, in which some small plant remains have been found, occasionally up to anthracite grade. These represent periods of relative sea-level lowstand, with probably near-shore swamp formation. The sedimentary facies suggest that deposition of the middle Carboniferous stratigraphy occurred in a narrow bathymetry range, from around the fair-weather wave base to coastal swamp areas. The abundant presence of coal layers within the shale and silt intervals illustrates the flatness and shallowness of the basin. Anthracite intervals are abundant in the middle Carboniferous stratigraphy of the Donbas region^50^,^51^. The coal intervals in the Donbas region are usually related to continental low-moor depositional environments during transgressions^52^ and have a wide extent with some seams covering the entire basin^50^.

Clay, silt, and limestones intercalate with sandstones in the middle Carboniferous of the Donbas region. We identify four sandstone facies according to their sedimentological characteristics. In the sedimentary logs of Fig. 3, these interpreted classes are indicated in a separate column. Sandstones of different facies often occur in stacked sequences. Based on visual inspection with a hand lens, the mineralogical composition of the sandstones is generally ~40% quartz, ~25% feldspar, ~25% rock fragments, heavy minerals and detrital mica. Grains have mostly sub-angular and high spherical shapes. Hereafter, sedimentary characteristics per distinguished group (A to D) are given.

Sandstones of Group A include (very) coarse, poorly sorted sandstones, which often contain tree trunk prints and other large plant remains. Massive beds are common although occasionally upper plane bed, large-scale trough structures, and well-graded current-induced trough cross-bedding occur. Grainsizes vary from fine sand to small pebbles of around 2–3 cm. Grading from fine sand to pebble-sized grains occurs but non-graded intervals are more prominent. The thickness of Group A sandstone intervals mainly varies between 1 to 5 m and they often occur in the lower half of a thick sandstone body where they are topped by sandstones of groups B and C (Fig. 3). Some of the very coarse intervals appear to only consist of quartz grains, but also intervals relatively rich in feldspar occur. Sandstones of Group A are least abundant of the four types of sandstones and only few paleocurrent measurements were collected, which indicate variable, but generally eastward paleoflow directions (Fig. 2).

The massive bedding, poor sorting, coarse grain size up to pebble-size, and large-scale trough structures are interpreted to reflect mass flow deposition. The frequent occurrence of prints of transported tree trunks suggests a terrestrial or near-shore environment. Hence, we suggest that these beds originate from short-lived high-energy fluvial pulses with high sedimentation rate preventing development of channel systems including point-bar sequences. Erosional surfaces have not been observed, which may indicate short-lived high runoff and sediment supply events that dumped coarse to pebbly sediments in the basin. The presence of immature sandstones, especially in the north, may indicate proximity to the source area. The preservation of the large tree trunk remnants shows low oxygen content at the site of deposition due to high sedimentation rate. Beds of Group A are frequently followed by beds of Group B which contain sedimentological indications for tidal influence, and we suggest that group A sandstones reflect river mouth or proximal deltaic mass-deposition events in times of high run-off.

Sandstones in Group B consist of middle to coarse-grained, moderately-sorted sands with current-induced cross beds. Some large foresets are up to 1 m thick, but most sets are around 20 to 50 cm high. Set boundaries tend to be parallel to the dominant layering and are continuous, especially when large Few tree trunk prints and some plant remains occur. Paleocurrents are eastward (Fig. 2).

The mainly horizontal set boundaries and set thicknesses of up to 1 m observed in these sandstones are interpreted to reflect high sediment supply and sufficient accommodation space. The lack of erosional surfaces and trough structures, as well as the finer sediment drapes on the set boundaries suggest tidal influence. Group B sandstones frequently follow on Group A sandstones and are hence interpreted as the lower energy continuation of the mass-flow deposits in a river mouth bar or upper delta regime, in shallow (marine) waters. We found no clear evidence for barrier island systems or long-shore bars, suggesting a river-dominated deltaic clastic regime.

Sandstones of group C consist of fine to middle-grained, well-sorted sandstones with current-induced small to intermediate scale (~15–30 cm) cross-beds. Some hummocky cross-stratification is present especially in the basal and top parts of the units where these structures intercalate with current-induced tabular cross-beds, with minor trough cross-stratification The sandstones of group C are generally 4 to 10 m thick. Some thin layers of finer sediments intercalate, mainly silts. Plant remains, mostly leaves and small branches, occur. Individual foresets are often well separated by films of finer sediment. We binned the paleocurrent directions measured in Groups C and D, showing generally SE-ward paleoflow (Fig. 2).

Sandstones in this group lack large-scale continuous current-induced cross-beds and are interpreted to result from lower or more continuous sediment supply and weaker currents. The occasional presence of hummocky cross-stratification in the basal and top parts of the sandstone unit in this group suggests open water environments. We therefore ascribe sandstones in this group to a lower to middle shoreface environment^53^. Some influence of tides may be represented by clear separation of individual foresets. Hence, Group C sandstones may either result from decreasing energy during deposition following deposition of Group B sandstones, or a more distal equivalent of Group B.

Sandstones of Group D are very-fine, to fine sands that are well sorted. Hummocky cross-stratification is common, as well as lower plane bed horizontal stratification and some small-scale wavy lamination. Sandstones with these characteristics are normally 10 cm, but occasionally up to a few meters thick. Locally, they form well-bedded units, with beds of around 10 to 50 cm. At many places they alternate with layers of silt and locally shales and rarely contain plant remains. Units of this group are generally laterally very continuous at outcrop scale. Sandstone units form the transitional lithology between intervals of shale and sandstone beds of other groups.

The fine sandstones in this group are interpreted as quieter marine deposits that lack evidence for proximity of a river mouth as suggested by the previously described groups. The sandstones are well sorted indicating some distance between source area and site of deposition. The common occurrence of hummocky cross-stratification and lower plane bed together with intercalation with (and transition to) mudstones suggests they are the most distal, lowest energy sandstone deposits observed in the stratigraphy. The occasional ripple cross-lamination in silts suggest sedimentation in water depths still above the storm wave base, but the absence of drapes on foresets and clear separation of sets that would suggest tidal influence leads us to interpret the depositional depth as below the fair-weather wave base^54^,^55^. This facies is therefore ascribed to lower shoreface environments^53^.

## Discussion

We report field observations and interpretations from the sedimentary geology of the middle Carboniferous stratigraphy of the Donbas Foldbelt, providing new environmental information in addition to the basin reconstructions based on previous field observations, and mostly borehole and seismic data^3^,^12^,^49^,^56^,^57^,^58^,^59^. Analysis of the sedimentary characteristics of the sandstone successions in the Donbas Foldbelt, the best exposed lithology, shows that the depositional environment varied between the terrestrial, low-energy coal environments and storm-wave base paleobathymetries: We interpret the four different groups of sandstones described above as river mouth to deltaic environments, with water depths generally confined to the upper tens of meters, above the storm wave-base. Fine-grained sediments including clay and silt intercalated with the most distal sandstone facies, our Group D, still provide evidence for some wave action during deposition, indicating that the storm wave-base can generally be regarded as the deepest marine facies observed.

The apparent continuity of the sheet-like sandstone bodies, the clear wave-generated sedimentary characteristics without showing beach deposits, the absence of erosional (sub-marine) channel systems and turbidites in studied sections, and the correlation potential of the thin limestones and coals all indicate the basin has been a very low gradient and shallow epi-continental shelf sea. The interpretation of the sandstones as fluvial, delta-front mouthbar, upper, middle and lower shoreface, and shelf sediments does however not clarify the specific absolute coastal profile that was present in the basin. The depth of fair-weather wave base commonly lies at approximately 5 to 15 m^60^, so shelf deposition was possibly just slightly deeper. Our facies interpretation suggests that throughout the middle Carboniferous, water depths throughout the Donbas region rarely exceeded 20–30 m. Throughout most of its exposed stratigraphy, the basin was in such relatively deep conditions for most of the time, and only short intervals of shallower conditions occurred, indicated by the deposition of sandstone types A and B, and the absence of paleosols, rooted vegetation, and clear erosion surfaces.

The clean, carbonate-poor, non-bioturbated sandstones containing tree trunks suggest high sedimentation rates in the basin^54^. The lack of erosional levels suggests that the succession is fairly continuous, as does the absence of evidence for paleosols. During the ~12 Myr of middle Carboniferous time, approximately 5 km of sediment was deposited, averaging to ~40 cm/kyr, in line with the sedimentary facies that indicate rapid dumping of clastics in a rapidly subsiding basin.

The narrow paleobathymetry range and the absence of evidence for large hiatuses in the studied sections show that rapid subsidence and sedimentation kept pace throughout the middle Carboniferous in the Donbas. Moreover, the absence of syn-sedimentary deformational features, internal angular unconformities and major mass-flow deposits – other than those of Group A sandstones, which are interpreted as rapidly dumped sandstones during high run-off events, suggest that subsidence was fast but gradual, in line with earlier interpretations that the middle Carboniferous was a time of thermo-tectonic subsidence of the DDB^12^,^40^.

The regionally and temporally very consistent SE-directed paleocurrent measurements (Fig. 2) also attest to this interpretation: sediment transportation was dominantly along-axis throughout the middle Carboniferous without clear local perturbations. These new data are more or less in line with the few published data on paleocurrent directions, which showed paleoflow to the south and southeast^49^.

The margins of the DDB in the Donbas in the middle Carboniferous must have been further to the north and south than currently outcropping, and were probably of low topography: basin inversion phases after the Carboniferous resulted in some shortening of the north-south extent of the Donbas Foldbelt^31^,^32^ and likely erosion of postrift sediments at the rift shoulders, and the sedimentary characteristics and paleoflow directions do not show signs for basin marginal facies. The consistent along-axis paleocurrent directions indicating southeastward paleo-flow, in combination with shallow-marine to paralic paleoenvironments suggest that further to the west, where the middle Carboniferous cannot be found in outcrop, similar, or fluvial, sand dominated stratigraphies can be expected. Our results suggest that the middle Carboniferous to the northwest of the Donbas region in the DDB thus likely contains abundant, more proximal fluvial sandstones, which may be important for the reservoir potential in this part of the stratigraphy.

## Conclusions

We present a field study of the sedimentology of the middle Carboniferous stratigraphy in the Donbas Foldbelt, eastern Ukraine. Our conclusions are:

  1) Deposition in the Donbas occurred throughout the middle Carboniferous in a shelf setting, with water depths rarely deeper than the storm wave-base. The deposition of coal amidst clay and siltstone, combined with the subordinate abundance of major fluvial environments or erosional unconformities suggest that the environment became terrestrial at the coastal level, without regressions leading to significant erosion of previously deposited sediments. All other sedimentary facies fall in paleobathymetry ranges between these extremes, with sandstone intercalations that were deposited mainly on the middle to lower shoreface and sometimes in a fluvial mouthbar system.

  2) Sedimentation throughout the middle Carboniferous of the Donbas kept pace with subsidence at high rates of ~40 cm/kyr averaged over the entire ~12 Myr time span represented by the stratigraphy. The absence of evidence for major syn-sedimentary faulting within our sections and the very consistent paleocurrent directions without major local deflections are in line with previous interpretations that subsidence and creation of accommodation space was dominated by thermo-tectonic subsidence of the DDB in a post-rift setting.

  3) The facies changes recorded in our sections are probably controlled by relative sea-level changes on the order of tens of meters superimposed on the continuous thermo-tectonic subsidence trend.

  4) A regionally and temporally very consistent paleoflow direction throughout the middle Carboniferous in all different sandstone types suggests paleo-currents dominated by along-axis infill of an identical northwest to southeast basin configuration as seen nowadays, with minor influence of infill from the basin margins.

  5) The coals, sands and shales present in the Donbas Foldbelt, comprise an intercalated stacked package of potential source rocks, reservoir sands and sealing lithologies, which likely continue towards the northwest into the Dniepr-Donets basin (DBB), one of the major hydrocarbon provinces of Europe. Our consistent SE-ward paleocurrent measurements across the middle Carboniferous of the Donbas Foldbelt suggests that the time-equivalent deposits at depth further to the northwest in the DDB are probably dominated by fluvial, sand-rich deposits, relevant for the assessment of reservoir potential.

## Methods

Results in this paper were obtained through field observation of sedimentary rocks using a magnetic compass, a hammer, a hand lense, a centimetre, a notebook, a pencil, and a digital camera.

## Author Contributions

D.J.J.v.H., D.G.M., M.G. and R.A.S. designed the research. D.J.J.v.H., H.A., W.B., F.B., A.K., M.H., D.G.v.d.M., M.G. and R.A.S. performed field research. D.J.J.v.H., H.A.A., F.B. and A.K. wrote the manuscript. D.J.J.v.H., H.A.A., W.B. and F.B. designed the figures. H.A.A. and W.B. wrote the online appendix. All authors reviewed the manuscript.

## Supplementary Material

Supplementary Information Supplementary Information
