# Supplementary material for: Sedimentary geology of the middle Carboniferous of the Donbas region (Dniepr-Donets basin, Ukraine)
Source: Sci Rep. 2015 Mar 20;5:9099. doi: 10.1038/srep09099 (PMC4367162; doi:10.1038/srep09099)
Supplement: Supplementary Information [file srep09099-s1.pdf]

Supplementary Information to:

Sedimentary geology of the middle Carboniferous of the Donbas region (Dniepr-Donets basin, Ukraine)

DOUWE J.J. VAN HINSBERGEN[1], HEMMO A. ABELS[1], WOLTER BOSCH[1], FLORA BOEKHOUT[2], ALEXANDER KITCHKA[4], MAARTJE HAMERS[1], DOUWE G. VAN DER MEER[3], MARK GELUK[5], RANDELL A. STEPHENSON[6]

1 *Department of Earth Sciences, University of Utrecht, Budapestlaan 4, 3584 CD Utrecht, the Netherlands*

2 *Institut für Geologie und Paläontologie, Westfälische Wilhelms-Universität, Corrensstrasse 24, 48149 Münster*

3 *Nexen Petroleum UK Ltd., Charter Place, Vine Street, Uxbridge, Middlesex, UB8 1JG, United Kingdom*

4 *SE NaukaNaftogaz Res. Inst., NJSC Naftogaz of Ukraine, 8 Kyivska St., 08132 Vyshneve, Ukraine*

5 *Shell International Exploration and Production B.V., Kessler Park 1, 2288 GS Rijswijk, the Netherlands*

6 *School of Geosciences, Meston Building, King's College, University of Aberdeen, Aberdeen AB24 3UE, UK*

Contains:

Extensive field documentation of all sedimentary sections, in alphabetic order, studied in the Donbas region, eastern Ukraine. All photos were taken by H.A. Abels and W. Bosch. Maps modified from Stovba and Stephenson[31].

Section: ***Bulavinskoye***

Location: 37U 0451370 UTM 5344133, central area

Situation: Bulavinskoye section is situated just to east of the village Булавинское (Bulavinskoye) in a river outcrop on the road to Олбховатка (Olbchovatka). Where the road goes down the top of the section starts immediately to the south of the road, on the west-flank of a small river.

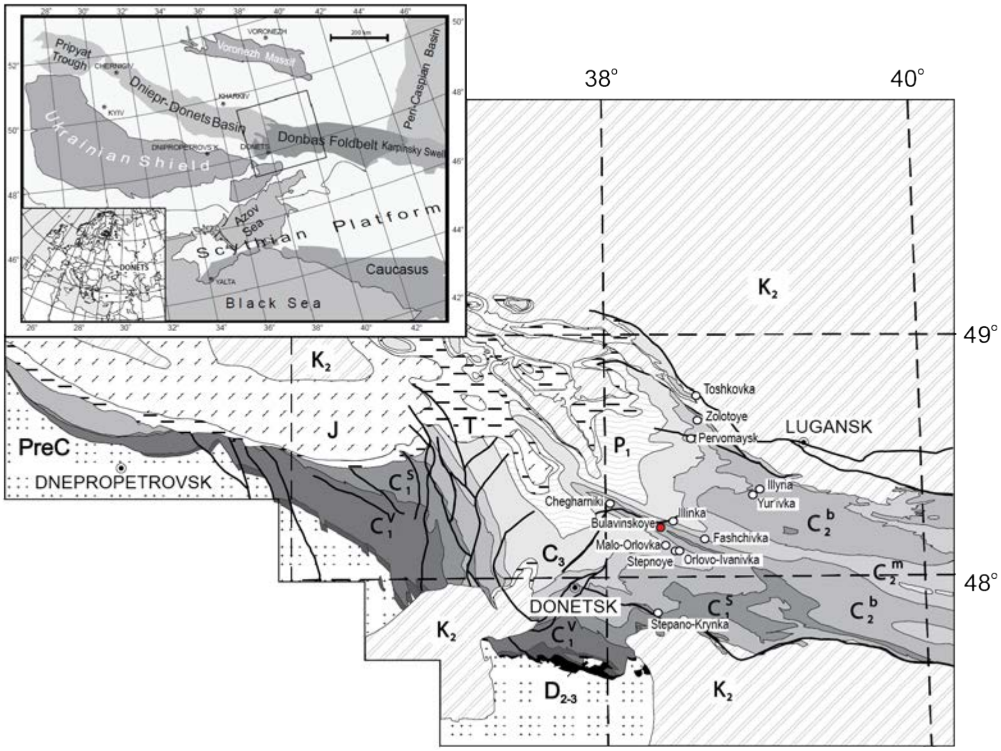

Age: **upper Bashkirian, C_2^3**, below limestone J_1

% Sand: **31 %**

Thickness: **180 m**

Sedimentology:

The Bulavinskoye section is characterised by fairly identical well-sorted fine sandstone units that contain hummocky cross-stratification or current induced large scale cross stratification (set height ~50 cm) and belong to Groups C and D. Paleocurrent directions in these sandstones are consistently northeast (Figure XB). A few sandstones in the section show coarser grains of which one bears tree trunk prints, bad sorting, and structure-less massive beds (Group A). This unit also shows some trough-like structures. Limestone beds are present in the middle of shaley intervals, and one close to a sandstone interval. One coal interval has been observed. Below the studied section a long interval of shales (~200 m?) is present and below that a thick sand-rich interval (~200 m?) has been observed in which among others coarse fluvial sandstones are present (Group A).

Environmental interpretation:

Most sandstones in the Bulavinskoye section belong to the Groups D and C, and are interpreted as middle to lower shoreface depositional environments. The presence of a sandstone of Group A indicates a pronounced sea level lowering in the middle of the section, also the occurrence of a coal layer close to the latter sandstone reveals this observation. The long interval of shales below the section is interpreted as a pronounced sea level high-stand.

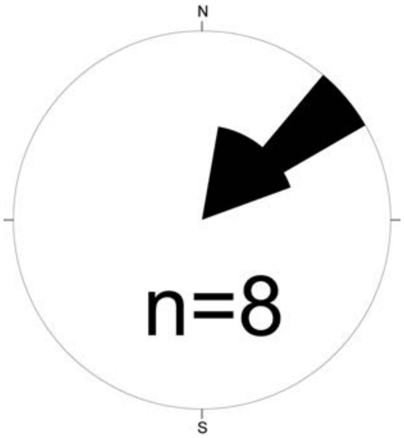

Figure XB. Rose diagram of all measured paleocurrent directions in the Bulavinskoye section. Petals in classes of 10°, largest petal 2 values and 25% of all.

MACROSCOPIC DESCRIPTION of **BULAVINSKOYE section 1-2**

Stratigraphic Units	Photographs	Columnar Section - scale 1 : 500 (Relief; Compos. Texture; shale/clay, fine silt, coarse silt, very fine sand, fine sand, middle sand, coarse sand, very coarse sand, granules, limestone)	Transport Direction	Type of Sst.	Additional DESCRIPTION and remarks

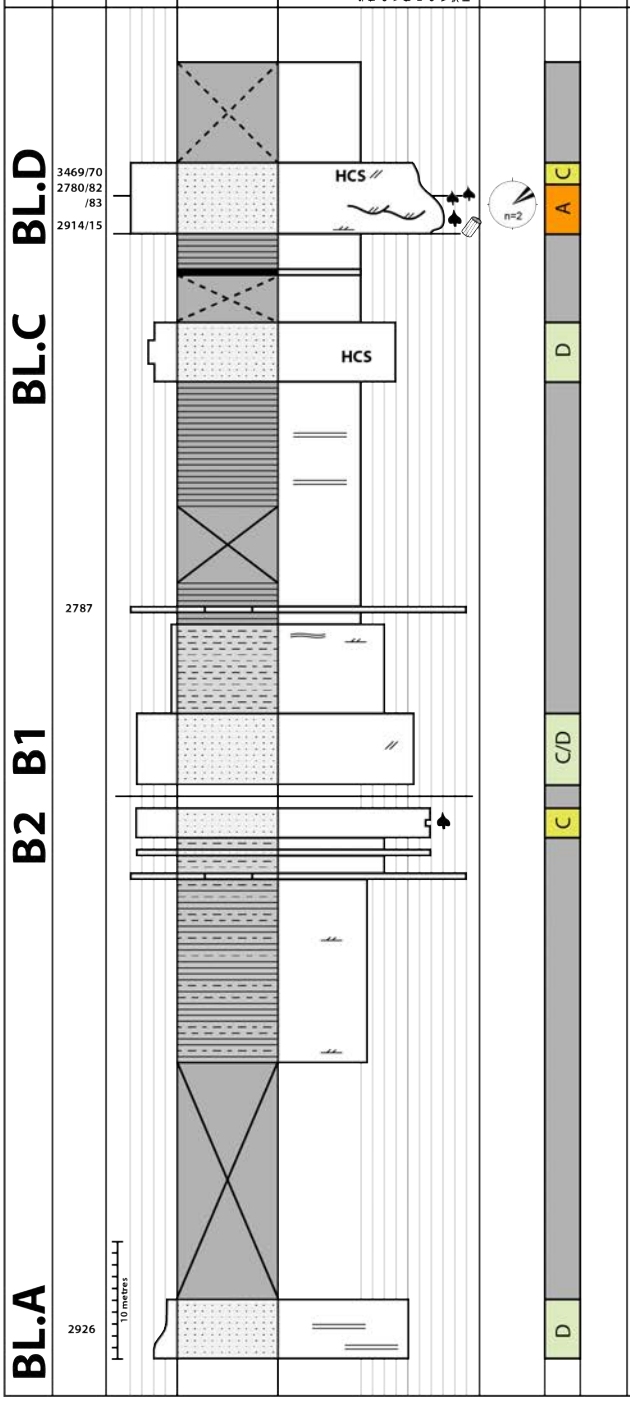

Continued on page 2-2

sandstone unit with at bottom middle sand with small-scale foresets and organic remains (until tree trunks of ~50 cm), in the middle part pebbly sandstone with troughs, organic remains, and mega-foresets(pointbars?), in top part fine-middle sand with HCS and foresets up to 30 cm in height. Fault disturbance occurs at bottom and in middle part, possibly reduced present thickness of unit considerably by removing softer material in between. BP 087/62 N

mica-rich silty shale

badly outcropping coal seem of ~50 cm in thickness

hummocky cross-stratified very-fine sandstone unit with increasing induration to middle and decreasing to top, Qtz, mica, feldspar(altered).

grey silty shale unit, no iron carbonates observed

LIMESTONE; limestone of H-series, brachiopod bearing black mudstone, 21 cm thick

Shaley coarse silt unit with very small cross-beds (< 2 cm, wave induced?) and wavy lamination, lower part badly outcropping

fine - middle sand unit with cross-beds of flow, not perfect outcrop, so no direction could be measured, mineral content similar to other units, bedding fairly thin

fault, possibly not major, decreases thickness of stratigraphy a bit

rather coarse sandstone unit consisting of mainly Qtz, white colour, quartzite

LIMESTONE: mudstone and packstone of 40 cm thickness, black colour, mainly bioclasts that are turitella-like shells and shell fragments

interval of moderately outcropping silty shales, with few intervals with silt beds that contain small cross-beds, no iron carbonate seen, grey colour not dark

not outcropping interval due to small river; most probably no sandstone units in this interval

fine sandstone unit with most probably horizontal lamination, no cut off seen, but outcrop not too good, BP 109/68 N

End of Section below there is a thick shale interval (over 60 m) and below that an interval with several thick sandstones appear (base of suite?)

MACROSCOPIC DESCRIPTION of BULAVINSKOYE section 2-2

Stratigraphic Units	Photographs	Columnar Section - scale 1 : 500 (Relief; Compos. Texture; shale/clay, fine silt, coarse silt, very fine sand, fine sand, middle sand, coarse sand, very coarse sand, granules, limestone)	Transport Direction	Type of Sst.		Additional DESCRIPTION and remarks

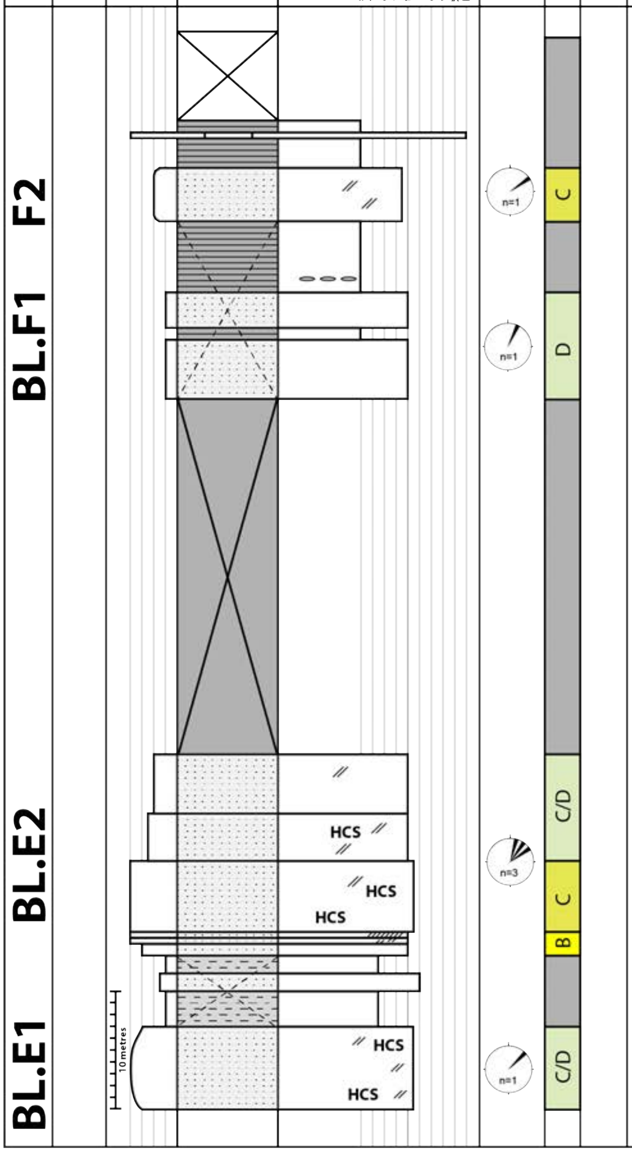

Top of Section ends at road

shale unit with fine sandstone (10cm thick) and limestone (black mudstone) close to each other (< 5 cm), no plant remains, shale has grey colour

very fine to fine sand unit full of mega-foresets (~ 50 cm in thickness), well sorted, p.c. direction dominant to one direction, mica, qtz, red minerals (oxidised feldspar?), black minerals, weathered unit, BP 086/78 N

bad outcropping shale interval, in lower part with a bit better outcrop grade iron carbonate nodules, colour grey

fine sand unit, badly exposed, no structures recognised, not drillable

badly outcropping fine sand unit with large cross-beds (height < 20 cm) in few good outcrops, BP 079/80 N

non-outcrop, heavily weathered shales?, soil very blackish, bit of digging doesn't help

fine sandstone unit, not perfect outcrop, large foresets <30 cm in height, of interval 80% sand and 20% shaley

idem as unit on top, more indurated, idem paleocurrent direction (outcrop obs.)

well-sorted fine to middle sandstone unit consisting of mega-foresets up to 1 meter in thickness and hummocky cross stratification, HCS hard to see due to bad outcrop, consists of mainly qtz, mica, red oxidised mineral (feldspar), and black minerals, fairly weathered, in toe-sets also middle sand grains, in lower part interval with alternating silt beds (2-5 cm) and fine foresetted sandstone beds (10-15 cm)

fine to middle sand unit, well-sorted, current induced mega-cross-beds, foreset height up to 50 cm, same mineral content as sand above, idem direction (outcrop obs.)

Continuation from page 1-2

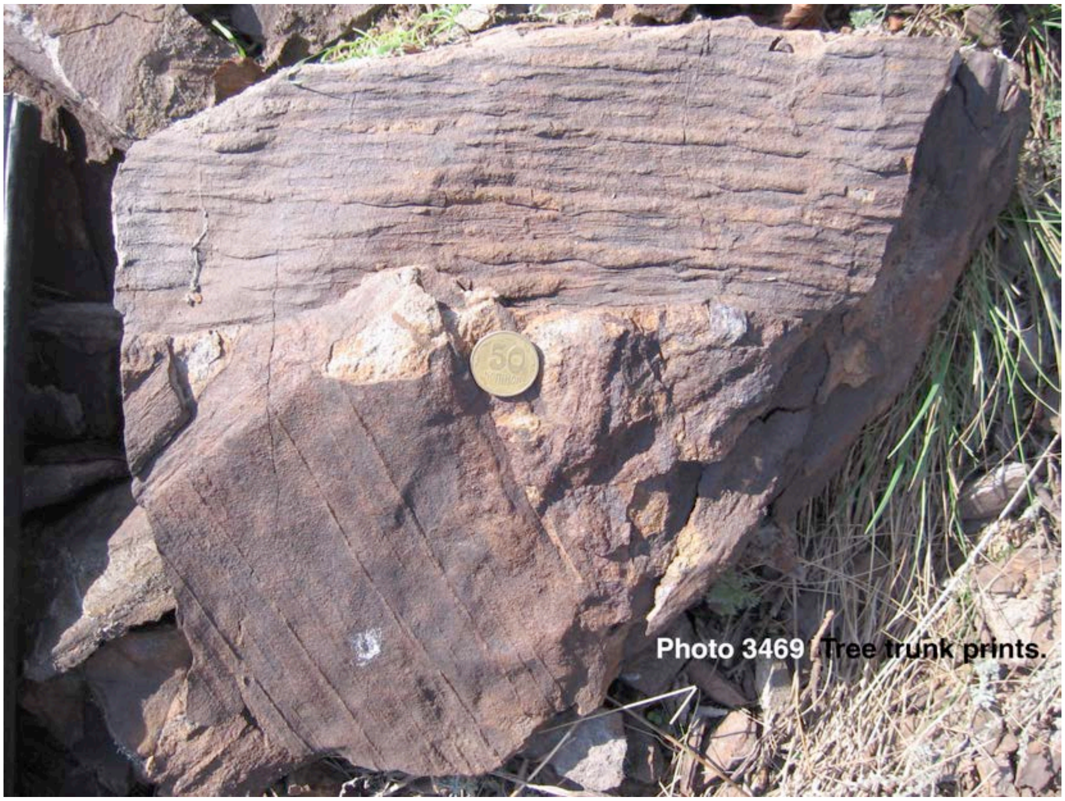

Photo 3469 Tree trunk prints.

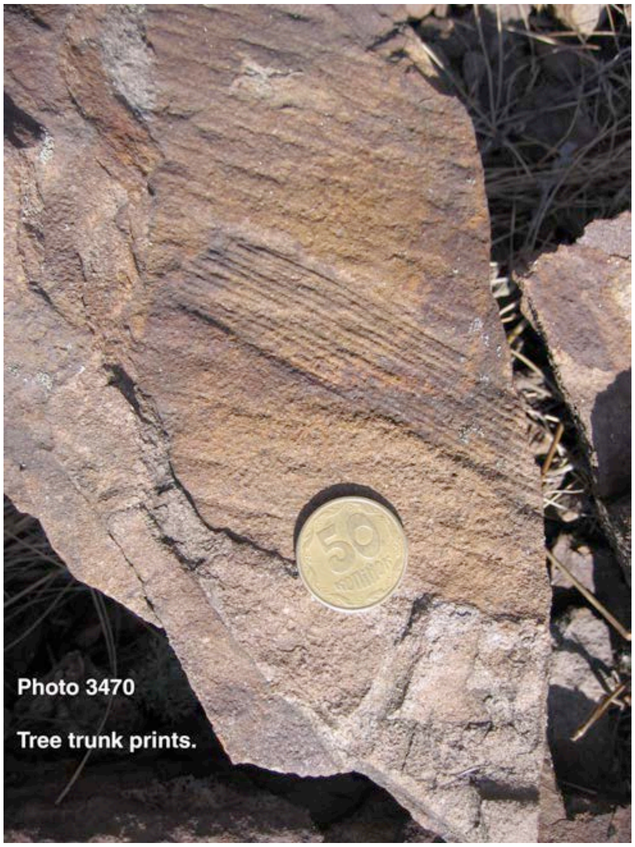

Photo 3470

Tree trunk prints.

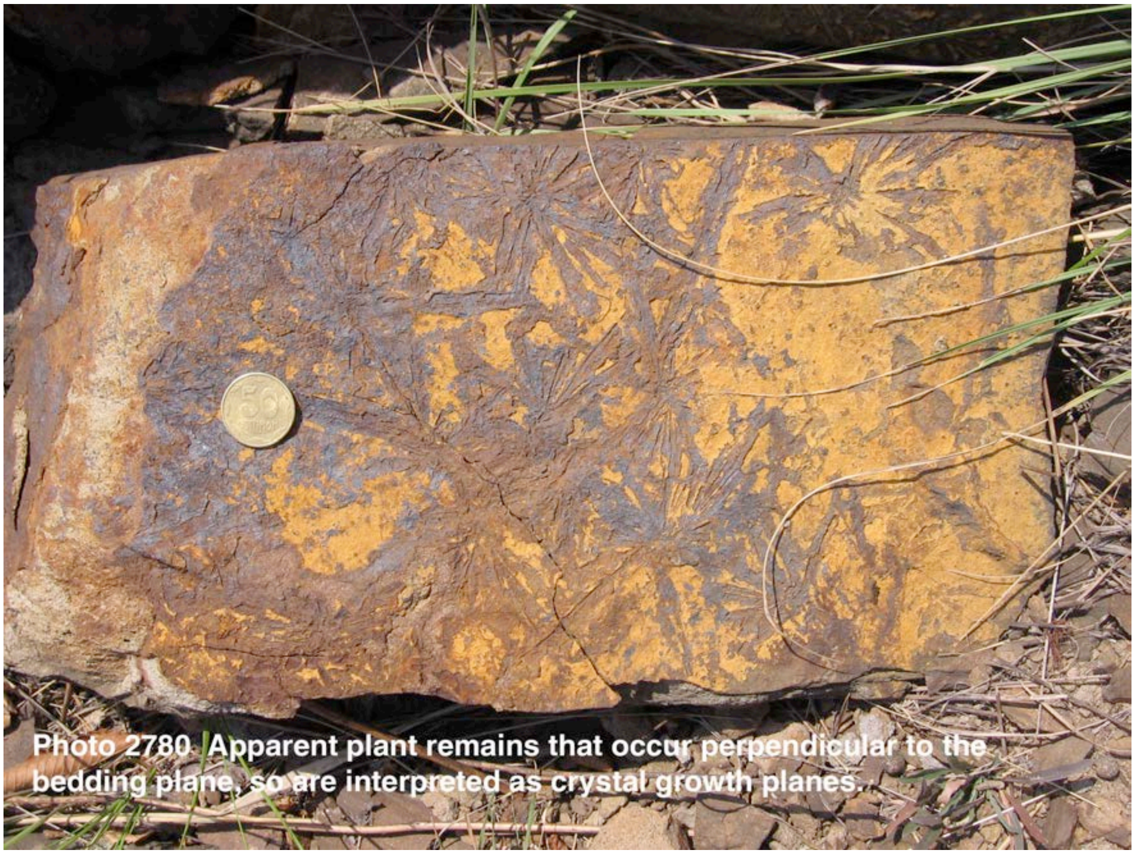

Photo 2780 Apparent plant remains that occur perpendicular to the bedding plane, so are interpreted as crystal growth planes.

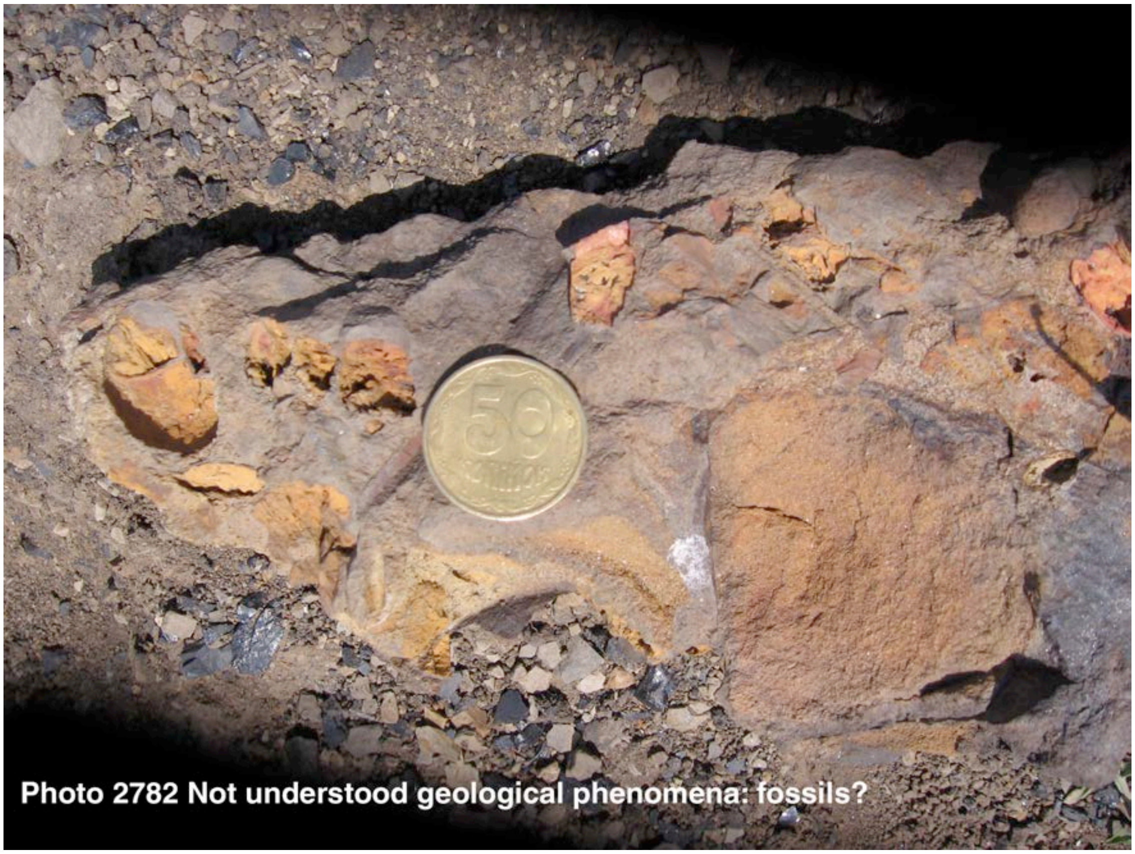

Photo 2782 Not understood geological phenomena: fossils?

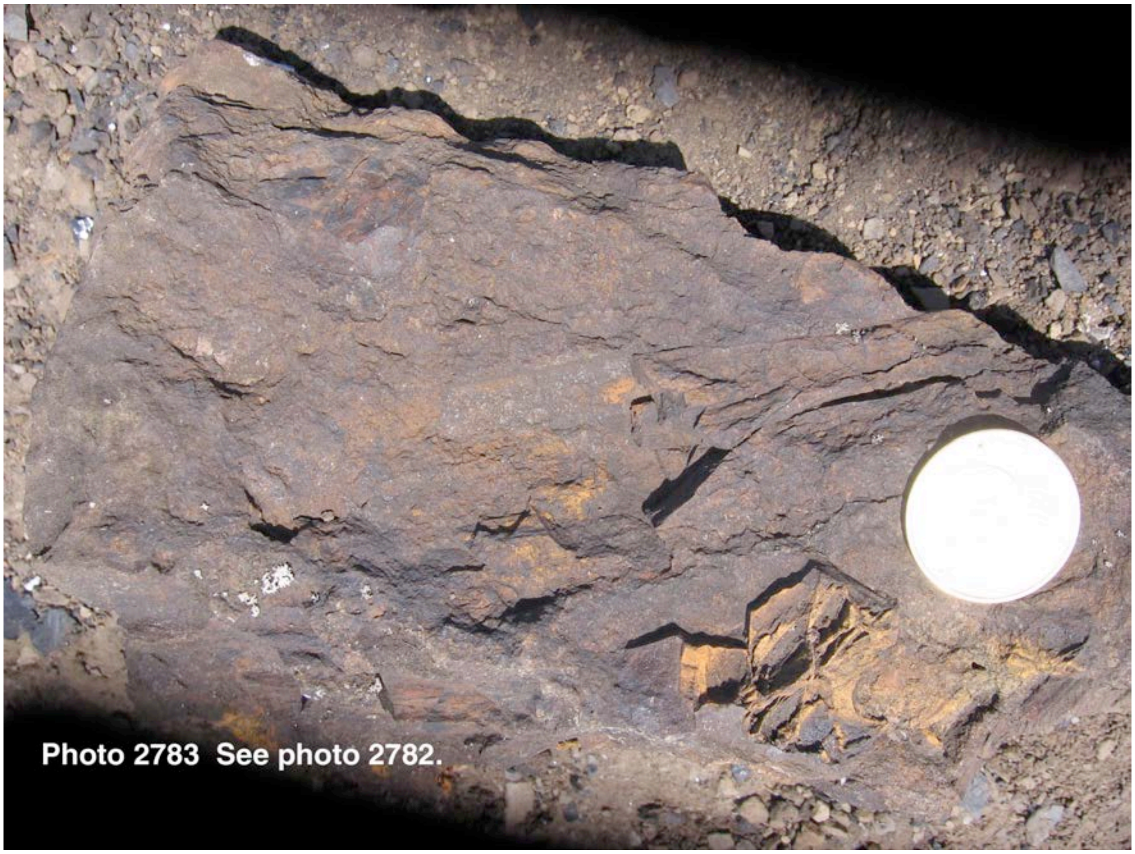
Photo 2783 See photo 2782.

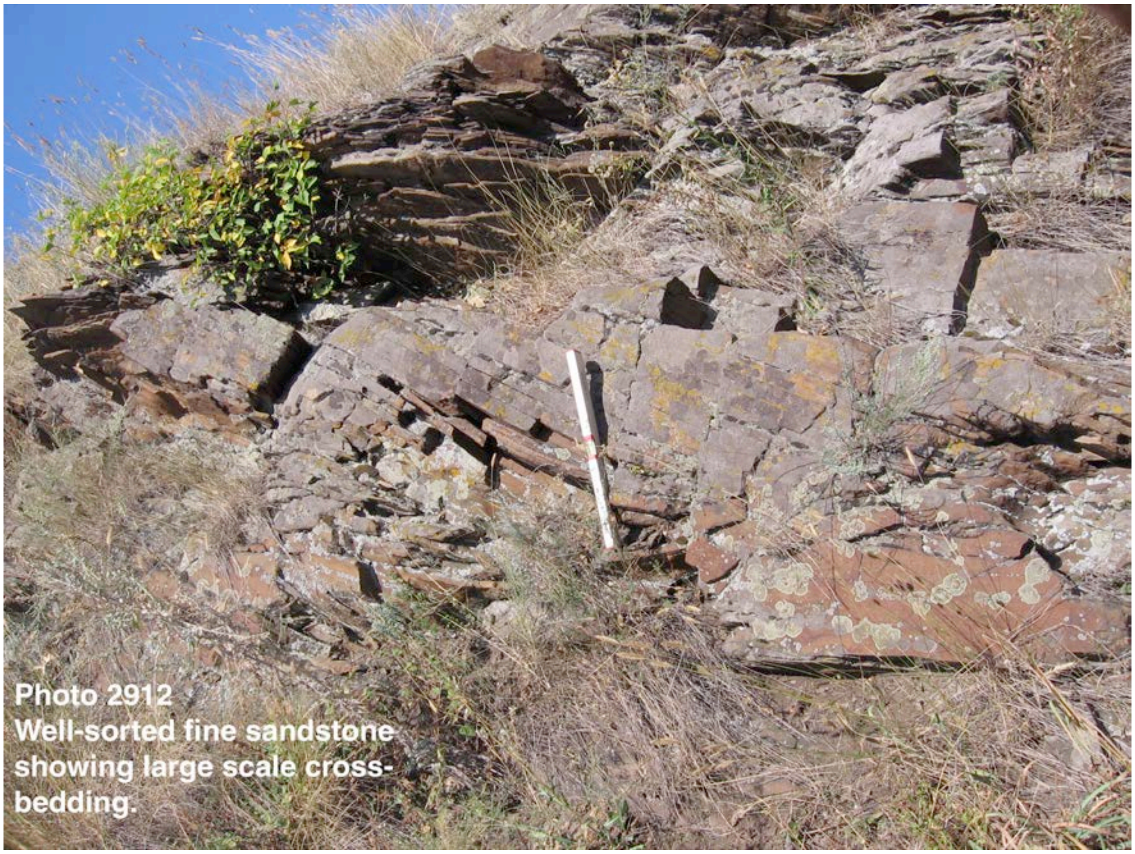
Photo 2912
Well-sorted fine sandstone showing large scale cross-bedding.

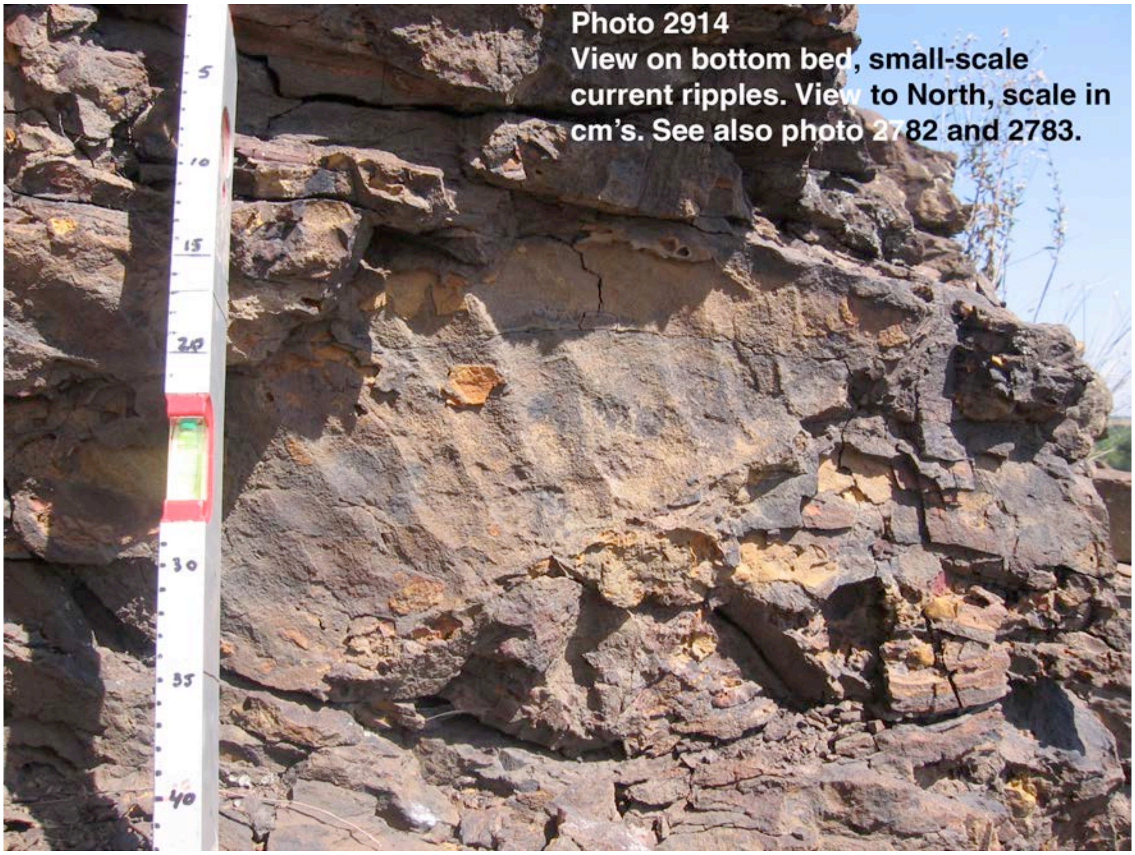

Photo 2914
View on bottom bed, small-scale current ripples. View to North, scale in cm's. See also photo 2782 and 2783.

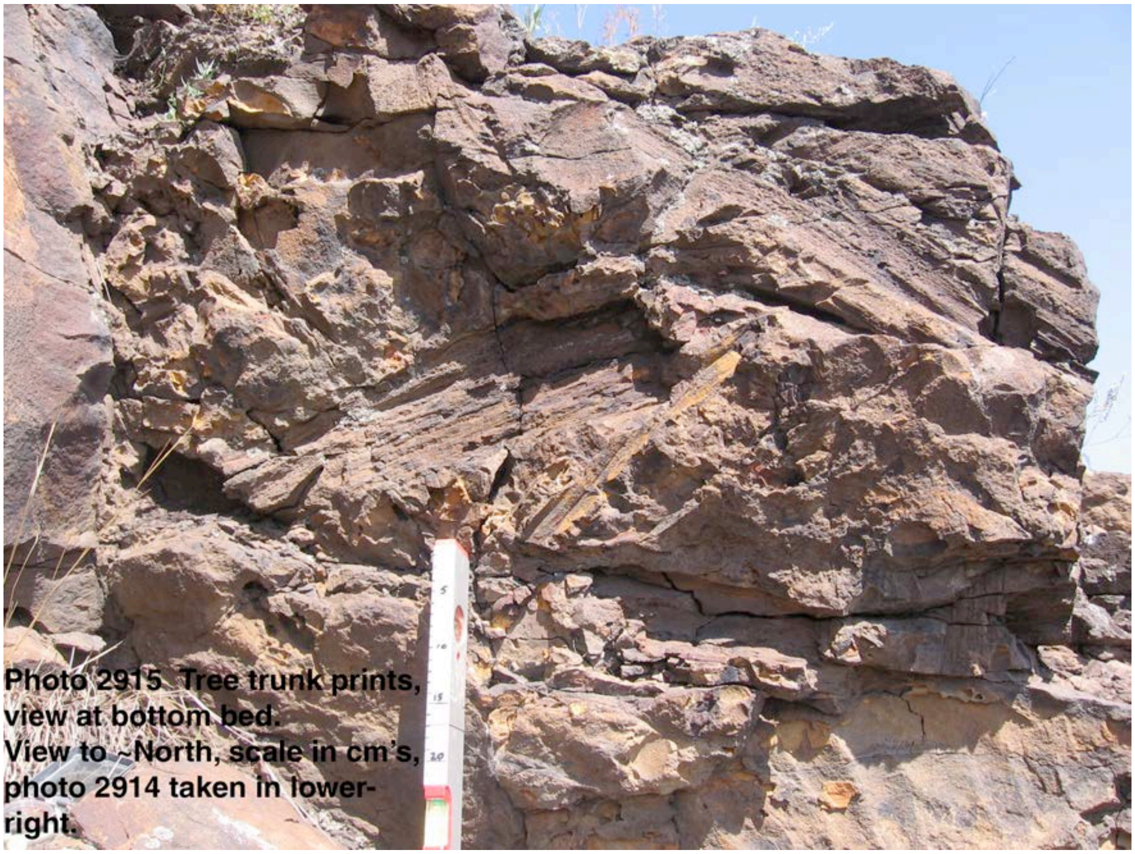

Photo 2915 Tree trunk prints, view at bottom bed.
View to ~North, scale in cm's, photo 2914 taken in lower-right.

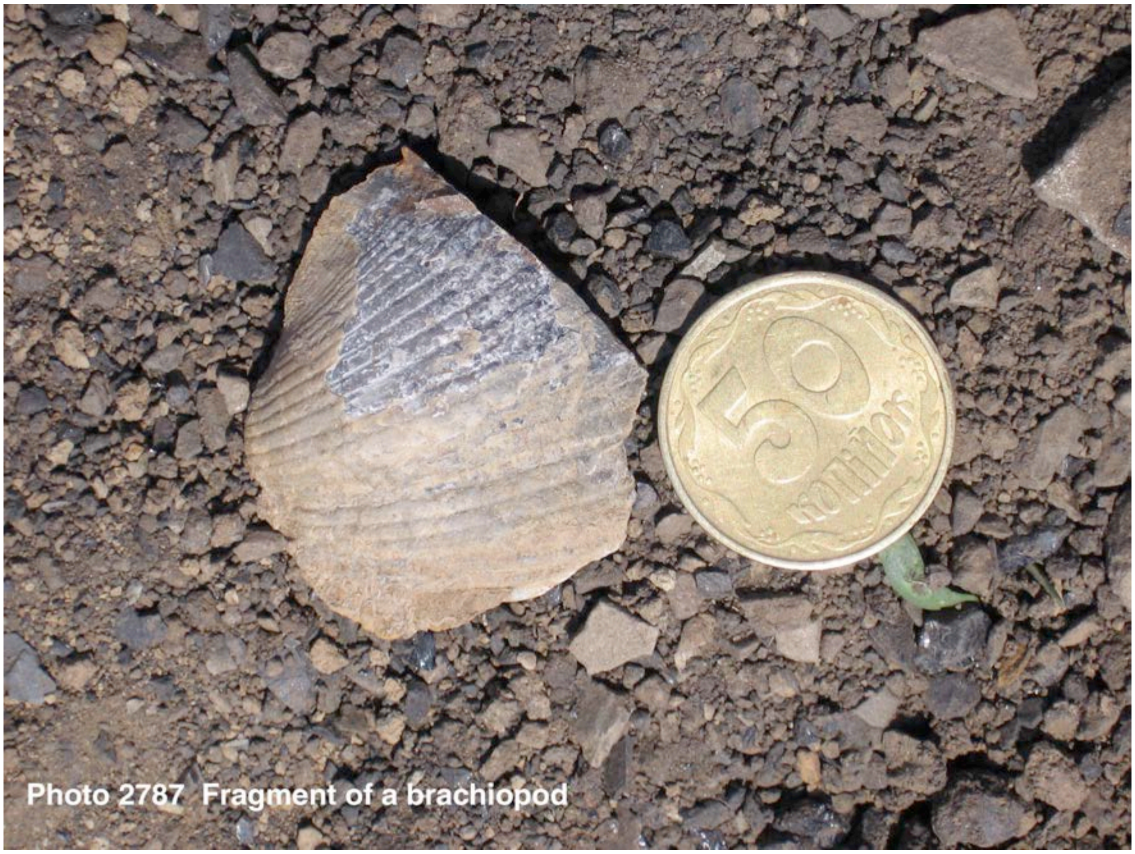

Photo 2787 Fragment of a brachiopod

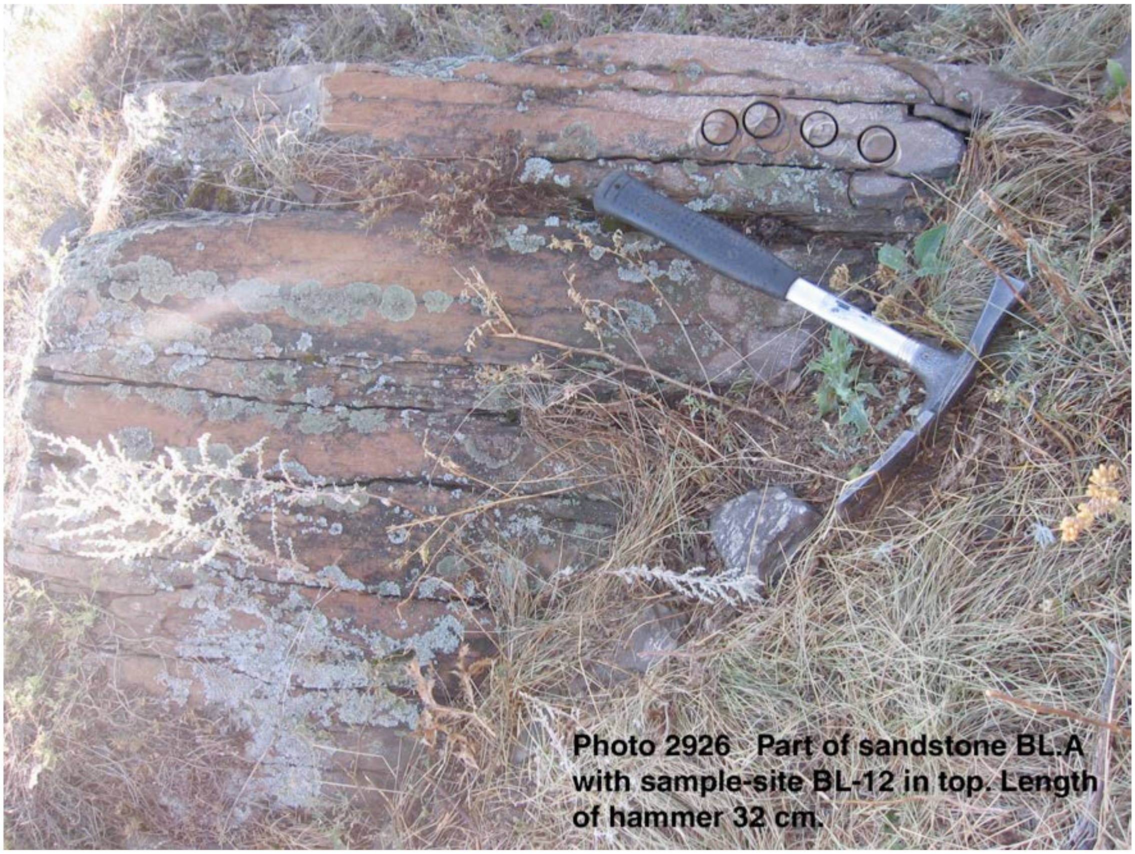

Photo 2926 Part of sandstone BL.A with sample-site BL-12 in top. Length of hammer 32 cm.

Section: ***Chegharniki***

Location: 37U 0426556 UTM 5355167, central area

Situation: Chegharniki section is named after the abandoned mercury quarry with the same name situated in the northern part of the city Горловка (Gorlovka). It can be reached by taking the main road southwest around the city to the area called Комарова (Kotarova). North of this area several quarries are situated on a row from east to west. The Chegharniki can be reached by taking a small road to the east (just after a bus stop) where the old white quarry tower is situated just to the west. The tower can be seen from distance.

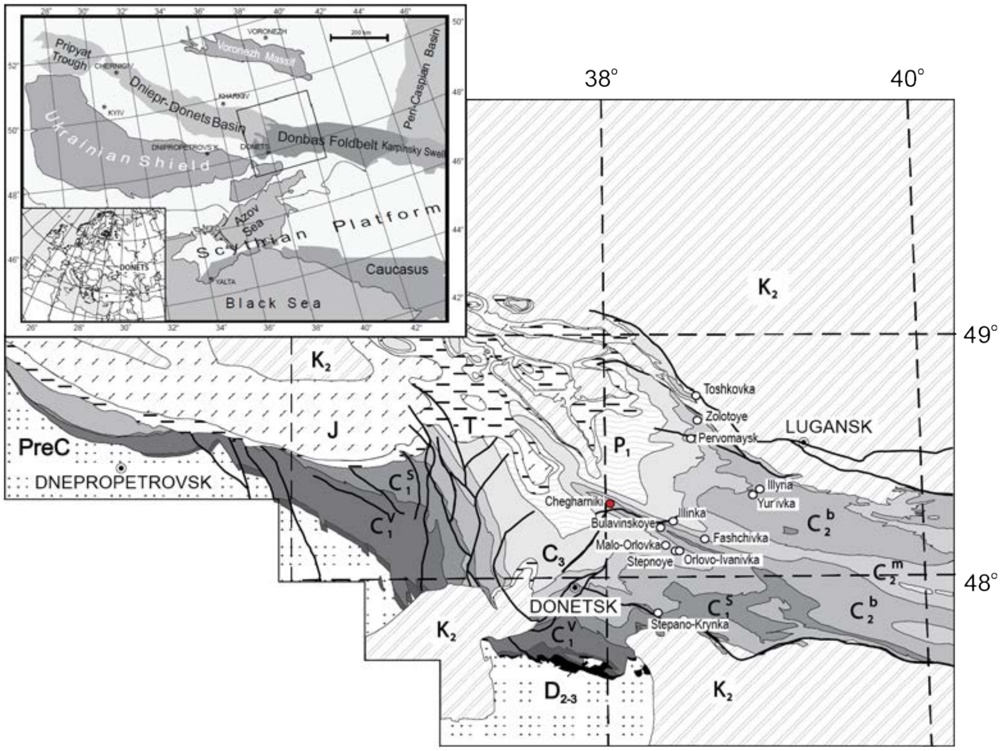

Age: **lower Bashkirian, C_2^2**, just below limestone H_1

% Sand: **39 %**

Thickness: **118 m**

Sedimentology:

The stratigraphy of the Chegharniki section is characterised by a relatively high sand content with respect to shales. In the middle and largest part of the section these sandstones are fine and well sorted and belong to Group D. The basal sandstone is fine too but shows current induced cross beds and belongs to Group C or D. The topmost very thick sandstone is coarser, show larger scale foresets, and some intervals with very poor sorting, therefore it has most characteristics from Group B and some of Group A. In this sandstone unit CH.D some bundling of finer and coarser individual foreset laminae is observed. In the section plant remains are common and no tree trunk prints have been found. The

sedimentary log of this section contains details for the shaley and silty intervals because the section has been studied in a good quarry outcrop. Too few paleocurrent measurements could be measured to derive a statistically correct paleocurrent direction for the complete section (Figure XC). The measured directions point to an eastward flow of currents, except for one measurement in the lower part of the section.

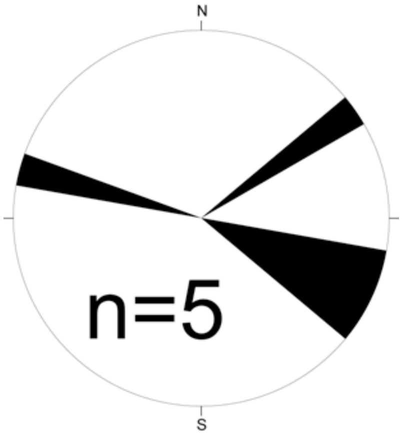

Figure XC. Rose diagram of all measured paleocurrent directions in the Chegharniki section. Petals in groups of 10°, largest petal 1 measurement and 20% of total.

Environmental interpretation:

The characteristics of the Chegharniki section point at a lower to middle shoreface depositional environment for the largest part of the section. Only at the top the sandstone unit aims at proximity of the continent and the sediments are interpreted as deltaic to upper shoreface with maybe minor fluvial intercalations. The presence of small scale wave cross bedding may be confusing as these sedimentary characteristics also often occur in very shallow water environments. The facies association indicates however deeper water environments and wavy lamination is known to also occur at greater bathymetry, even between depth of occurrence of hummocky cross-stratification and storm wave base (Duke et al., 1985). Above the upper shoreface sandstone at the top the environment becomes deeper again.

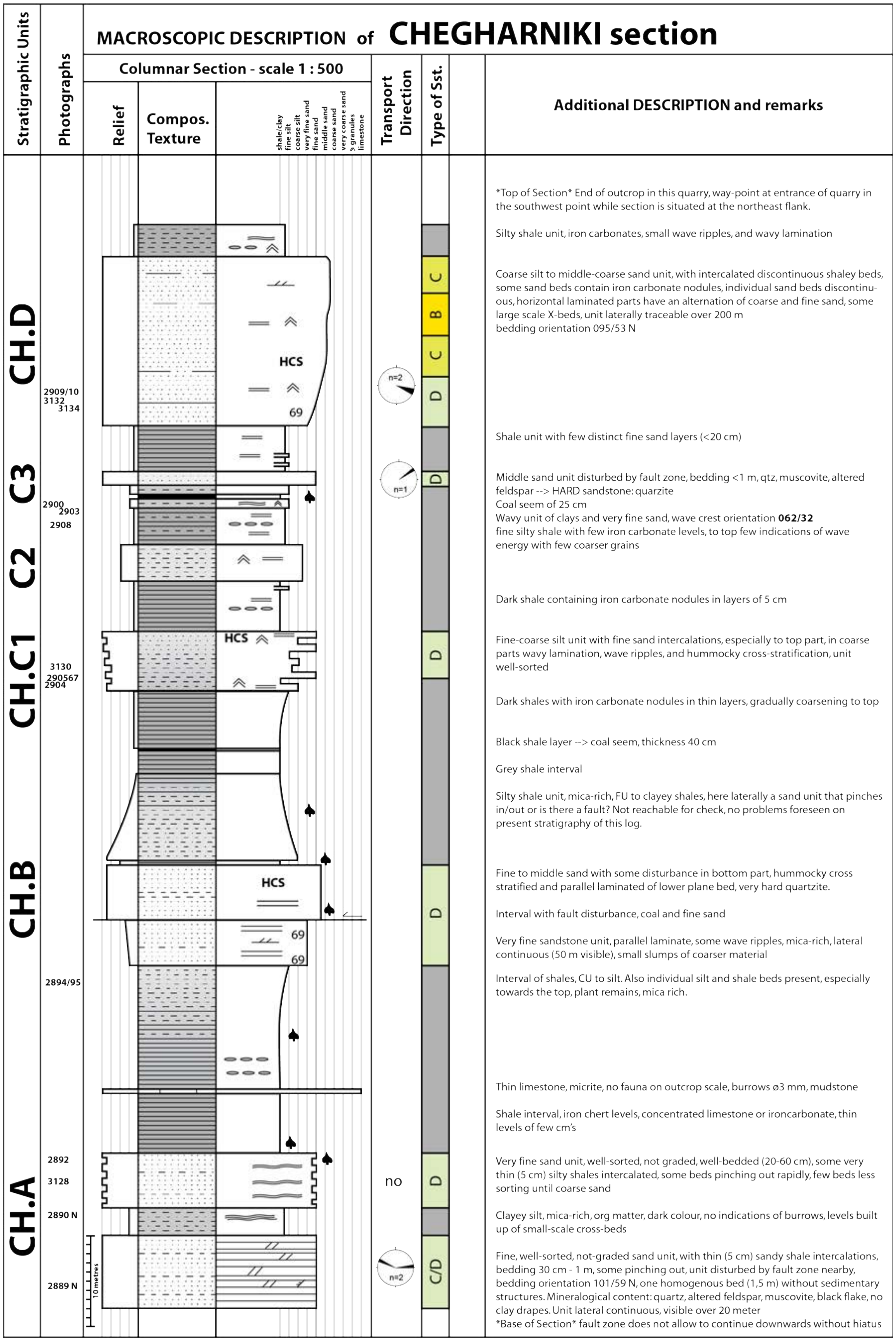
MACROSCOPIC DESCRIPTION of CHEGHARNIKI section
Stratigraphic Units
Photographs
Columnar Section - scale 1 : 500
Relief
Compos. Texture
shale/clay
fine silt
coarse silt
very fine sand
fine sand
middle sand
coarse sand
very coarse sand
› granules
limestone
Transport Direction
Type of Sst.
Additional DESCRIPTION and remarks
CH.D
C3
C2
CH.C1
CH.B
CH.A
2909/10
3132
3134
2900
2903
2908
3130
290567
2904
2894/95
2892
3128
2890 N
2889 N
10 metres
HCS
69
n=2
n=1
no
C
B
D
C/D
Top of Section End of outcrop in this quarry, way-point at entrance of quarry in the southwest point while section is situated at the northeast flank.
Silty shale unit, iron carbonates, small wave ripples, and wavy lamination
Coarse silt to middle-coarse sand unit, with intercalated discontinuous shaley beds, some sand beds contain iron carbonate nodules, individual sand beds discontinuous, horizontal laminated parts have an alternation of coarse and fine sand, some large scale X-beds, unit laterally traceable over 200 m
bedding orientation 095/53 N
Shale unit with few distinct fine sand layers (<20 cm)
Middle sand unit disturbed by fault zone, bedding <1 m, qtz, muscovite, altered feldspar --> HARD sandstone: quartzite
Coal seem of 25 cm
Wavy unit of clays and very fine sand, wave crest orientation 062/32
fine silty shale with few iron carbonate levels, to top few indications of wave energy with few coarser grains
Dark shale containing iron carbonate nodules in layers of 5 cm
Fine-coarse silt unit with fine sand intercalations, especially to top part, in coarse parts wavy lamination, wave ripples, and hummocky cross-stratification, unit well-sorted
Dark shales with iron carbonate nodules in thin layers, gradually coarsening to top
Black shale layer --> coal seem, thickness 40 cm
Grey shale interval
Silty shale unit, mica-rich, FU to clayey shales, here laterally a sand unit that pinches in/out or is there a fault? Not reachable for check, no problems foreseen on present stratigraphy of this log.
Fine to middle sand with some disturbance in bottom part, hummocky cross stratified and parallel laminated of lower plane bed, very hard quartzite.
Interval with fault disturbance, coal and fine sand
Very fine sandstone unit, parallel laminate, some wave ripples, mica-rich, lateral continuous (50 m visible), small slumps of coarser material
Interval of shales, CU to silt. Also individual silt and shale beds present, especially towards the top, plant remains, mica rich.
Thin limestone, micrite, no fauna on outcrop scale, burrows ø3 mm, mudstone
Shale interval, iron chert levels, concentrated limestone or ironcarbonate, thin levels of few cm's
Very fine sand unit, well-sorted, not graded, well-bedded (20-60 cm), some very thin (5 cm) silty shales intercalated, some beds pinching out rapidly, few beds less sorting until coarse sand
Clayey silt, mica-rich, org matter, dark colour, no indications of burrows, levels built up of small-scale cross-beds
Fine, well-sorted, not-graded sand unit, with thin (5 cm) sandy shale intercalations, bedding 30 cm - 1 m, some pinching out, unit disturbed by fault zone nearby, bedding orientation 101/59 N, one homogenous bed (1,5 m) without sedimentary structures. Mineralogical content: quartz, altered feldspar, muscovite, black flake, no clay drapes. Unit lateral continuous, visible over 20 meter
Base of Section fault zone does not allow to continue downwards without hiatus

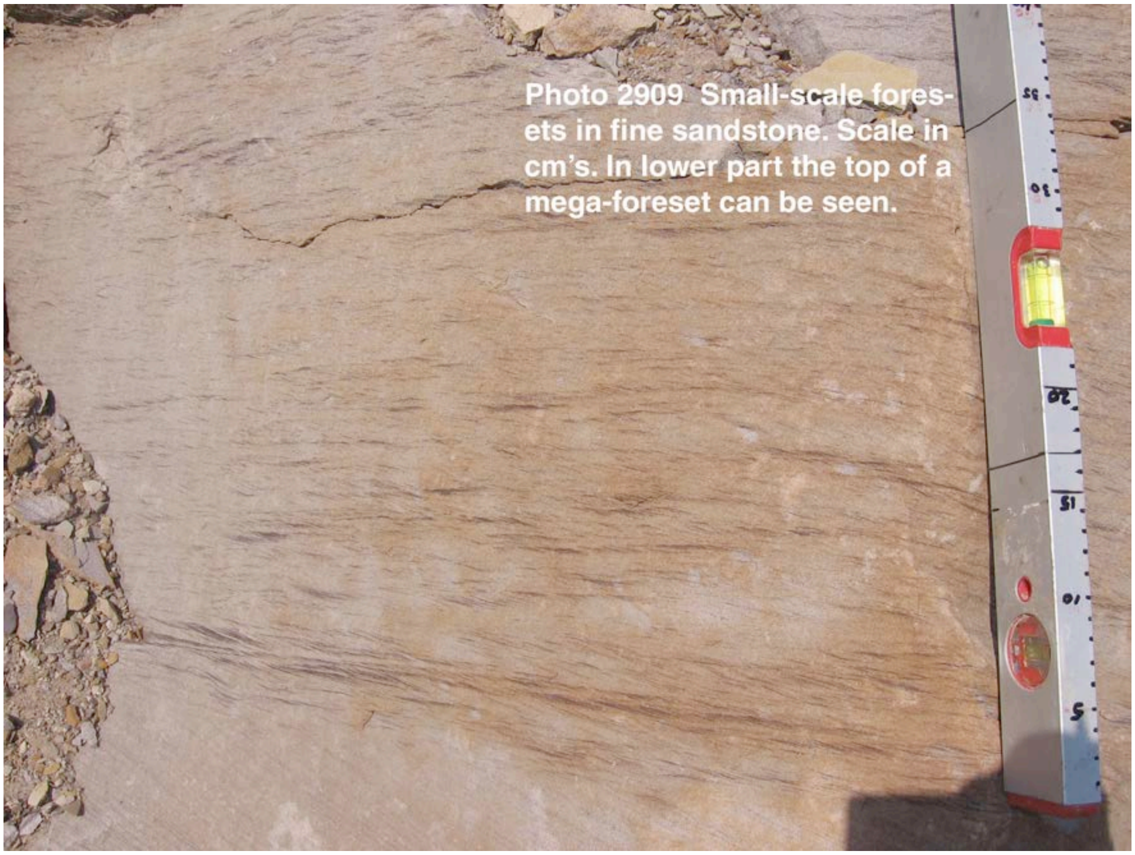

Photo 2909 Small-scale foresets in fine sandstone. Scale in cm's. In lower part the top of a mega-foreset can be seen.

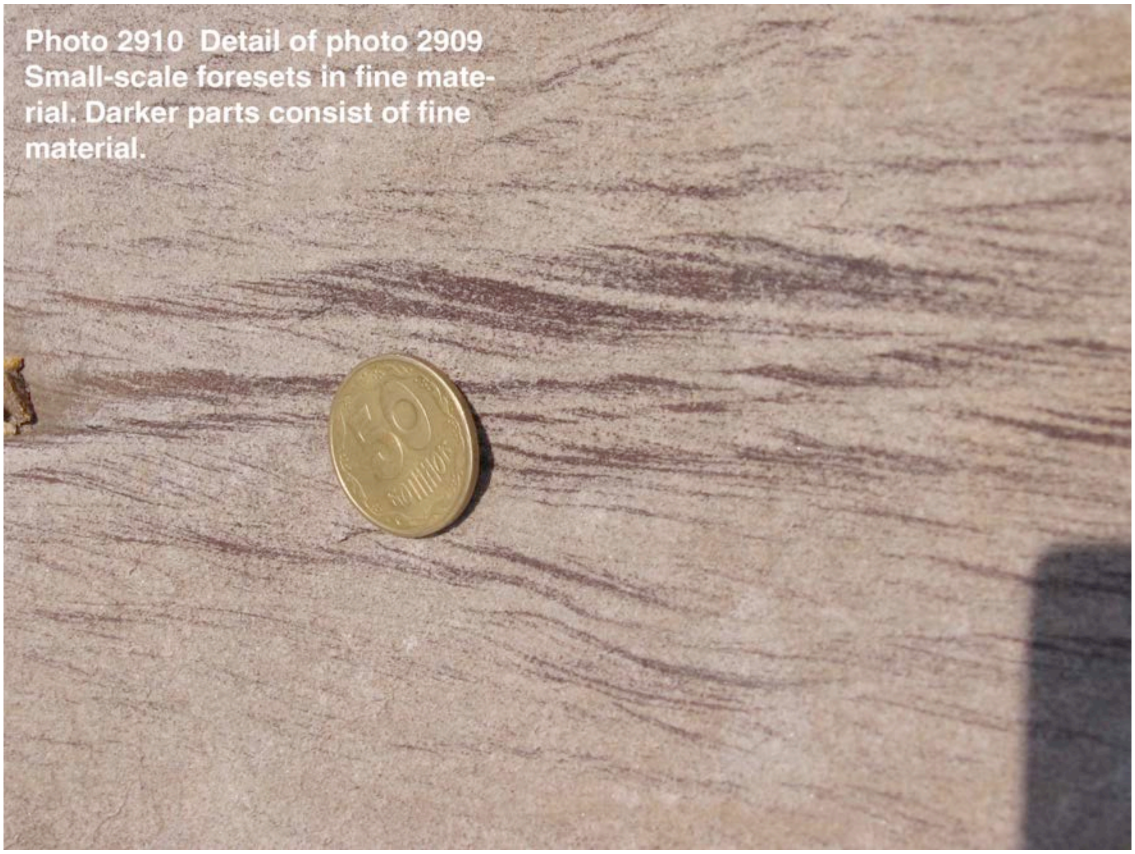

Photo 2910 Detail of photo 2909 Small-scale foresets in fine material. Darker parts consist of fine material.

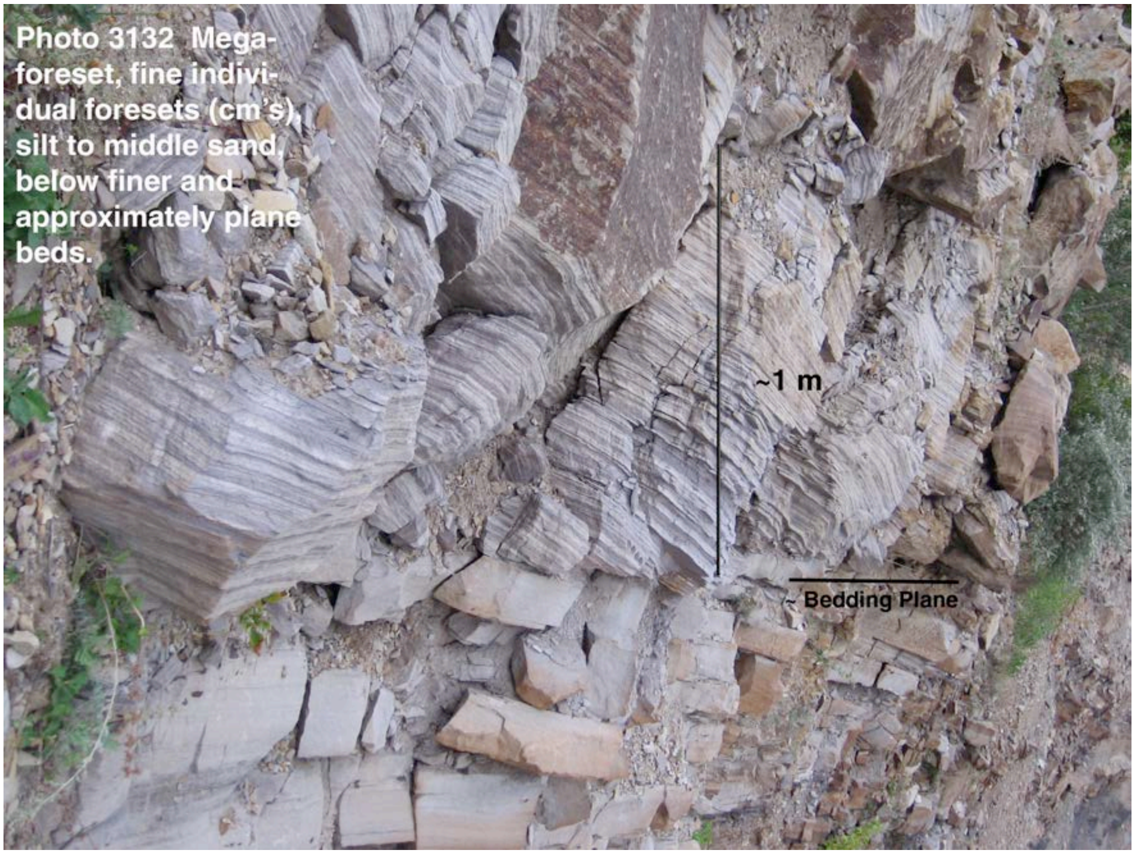

Photo 3132 Mega-foreset, fine individual foresets (cm's), silt to middle sand, below finer and approximately plane beds.

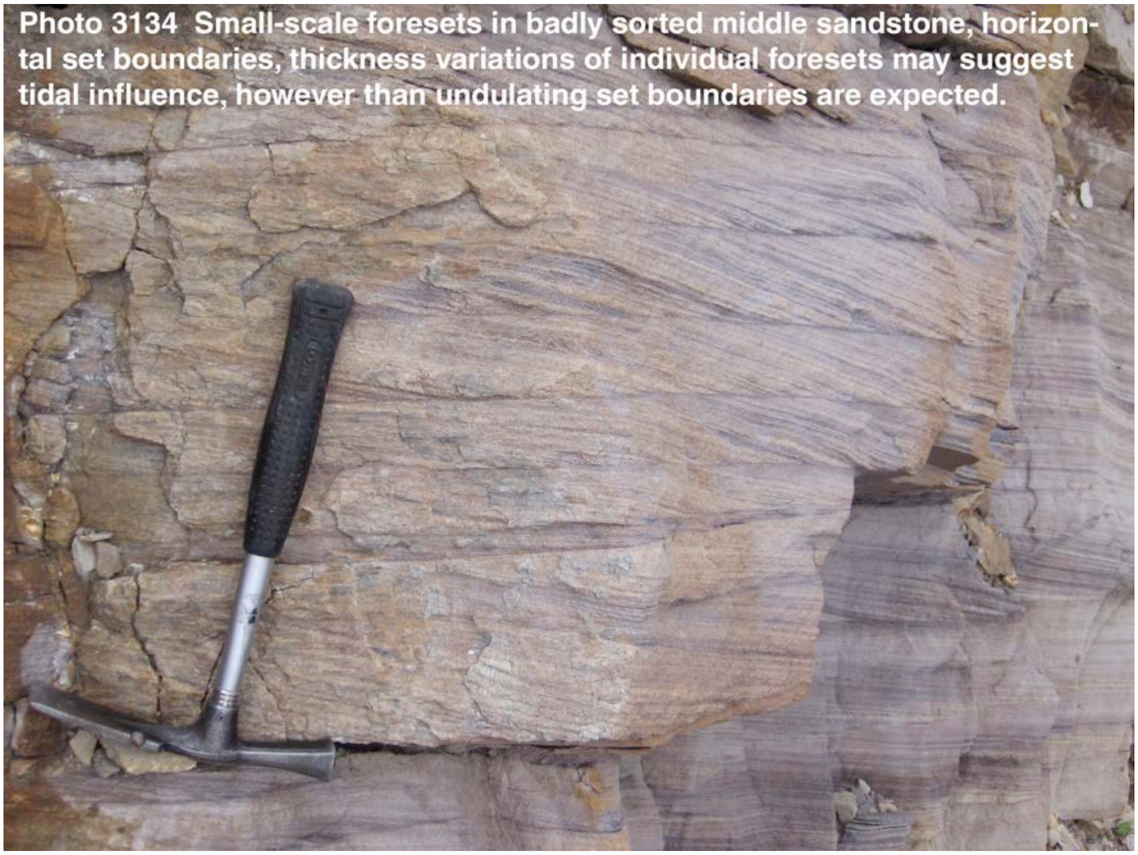

Photo 3134 Small-scale foresets in badly sorted middle sandstone, horizontal set boundaries, thickness variations of individual foresets may suggest tidal influence, however than undulating set boundaries are expected.

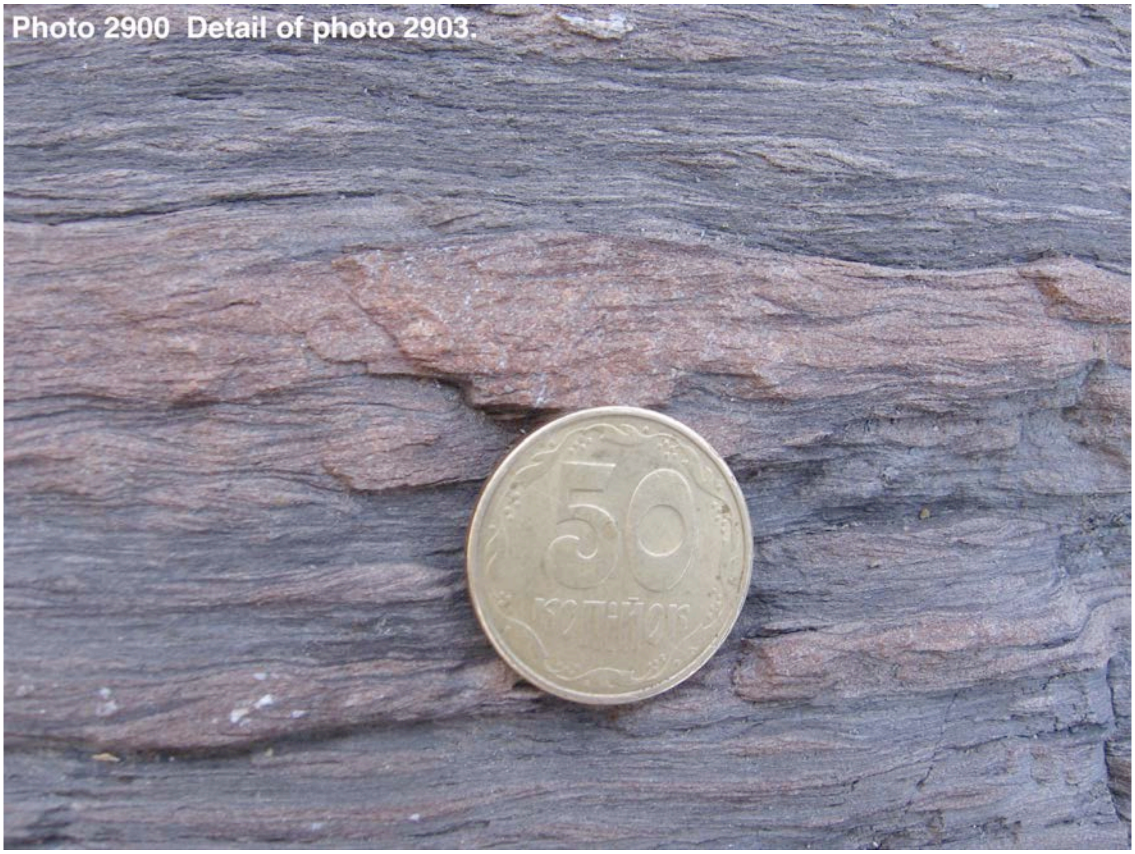

Photo 2900 Detail of photo 2903.

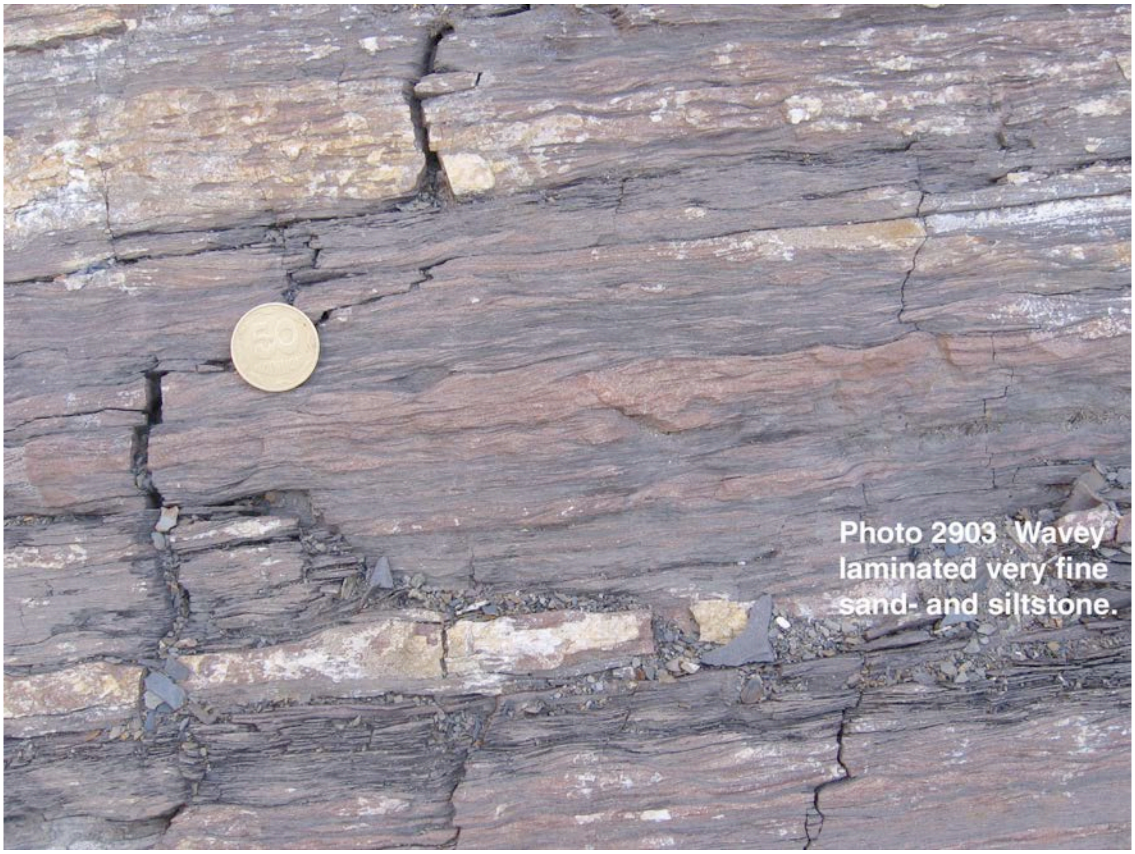

Photo 2903 Wavey laminated very fine sand- and siltstone.

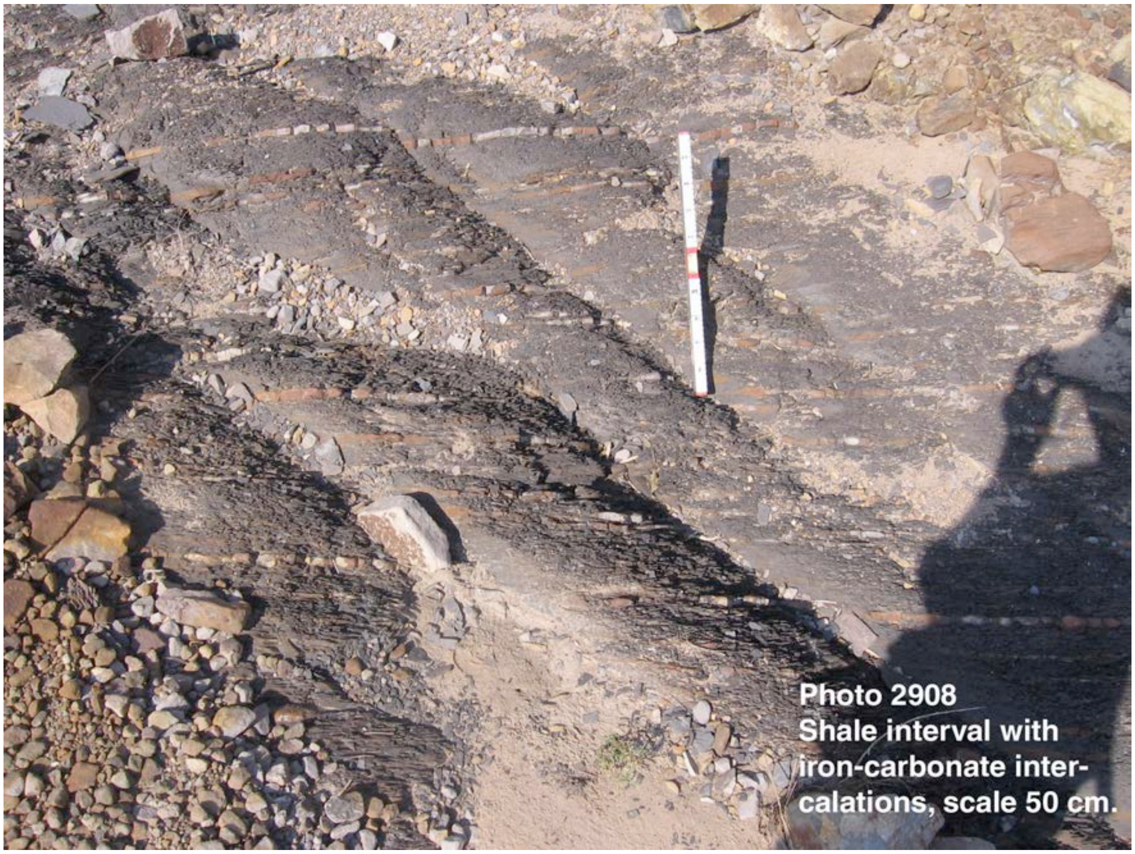

Photo 2908
Shale interval with iron-carbonate intercalations, scale 50 cm.

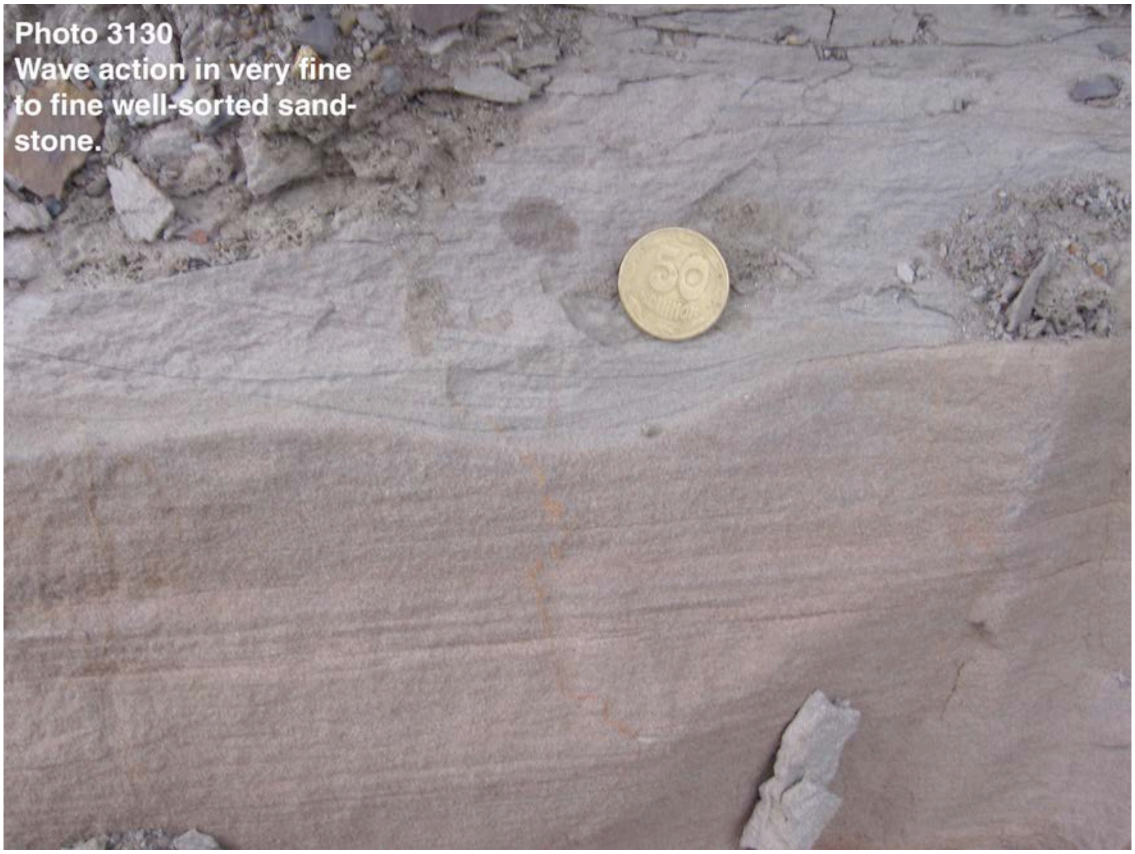

Photo 3130
Wave action in very fine to fine well-sorted sandstone.

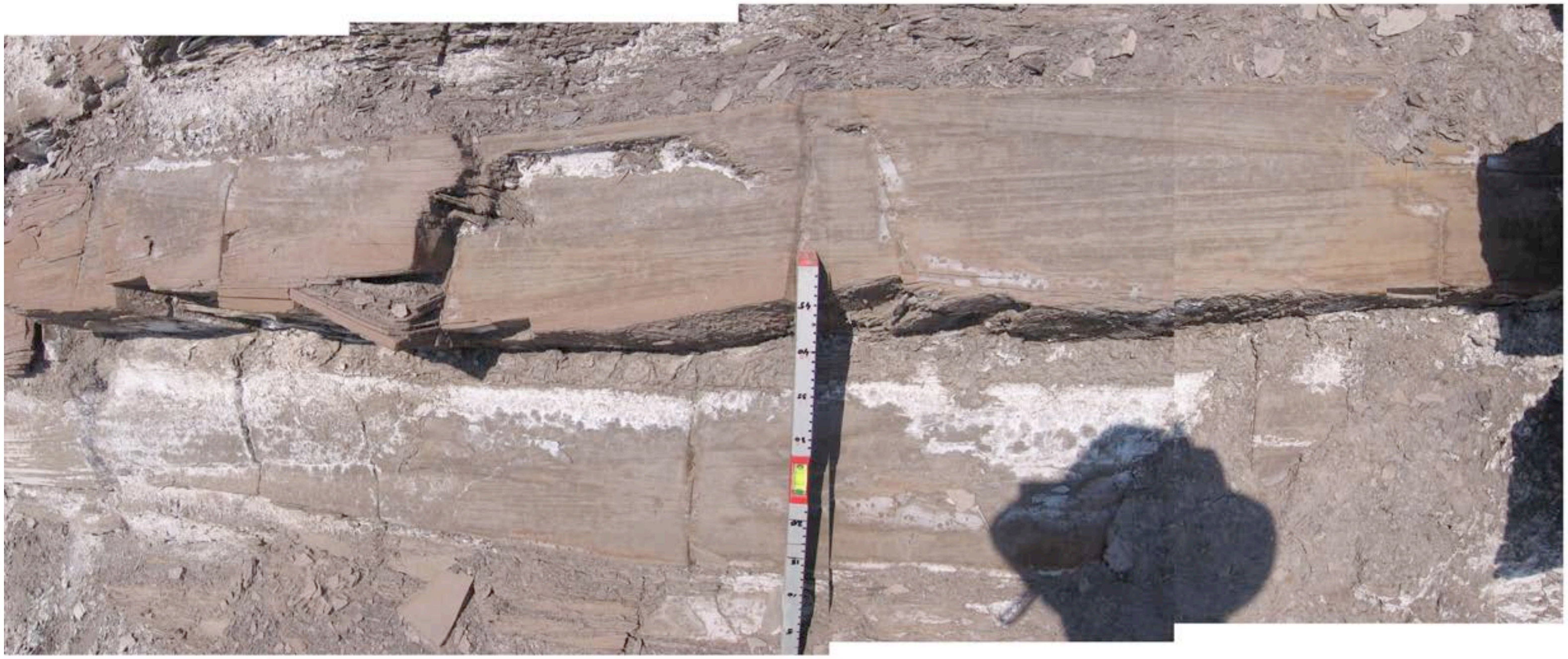

Photos 2905/06/07 Very fine sandstone bed, hummocky cross stratified, scale in cm's.

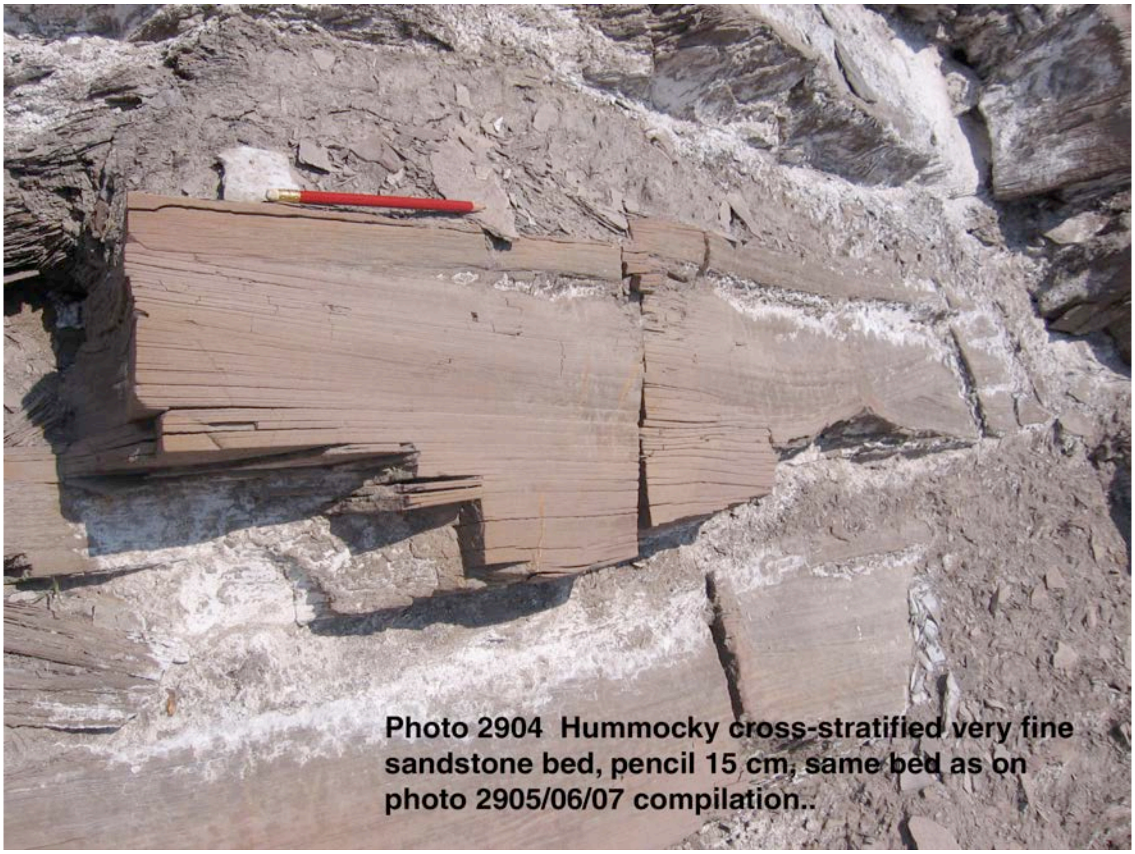

Photo 2904 Hummocky cross-stratified very fine sandstone bed, pencil 15 cm, same bed as on photo 2905/06/07 compilation..

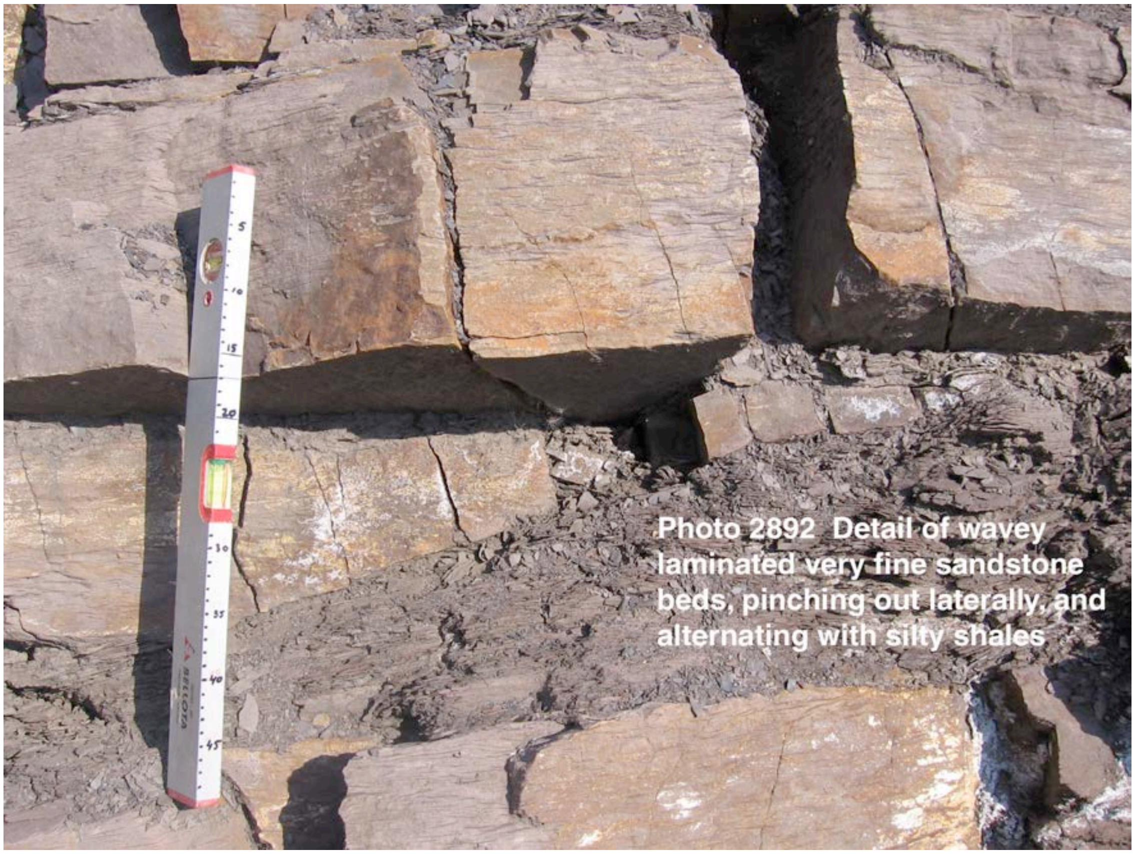

Photo 2892 Detail of wavey laminated very fine sandstone beds, pinching out laterally, and alternating with silty shales

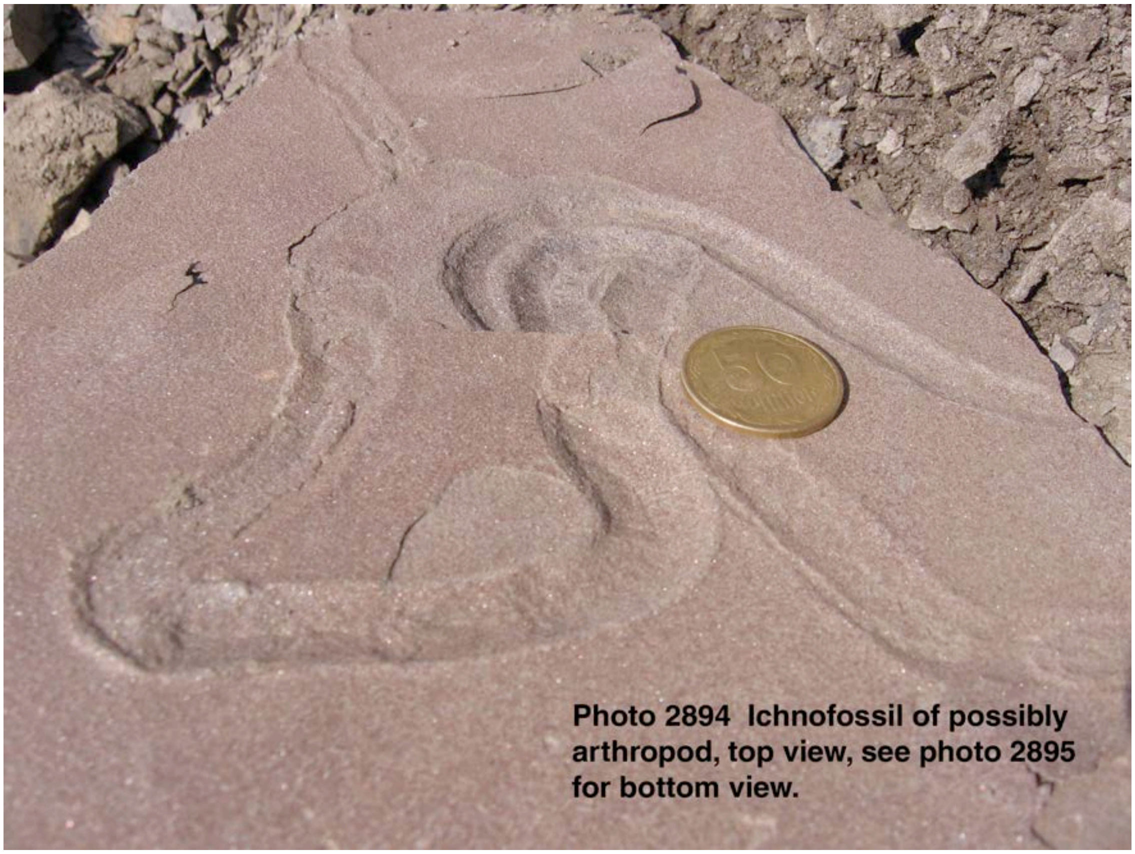

Photo 2894 Ichnofossil of possibly arthropod, top view, see photo 2895 for bottom view.

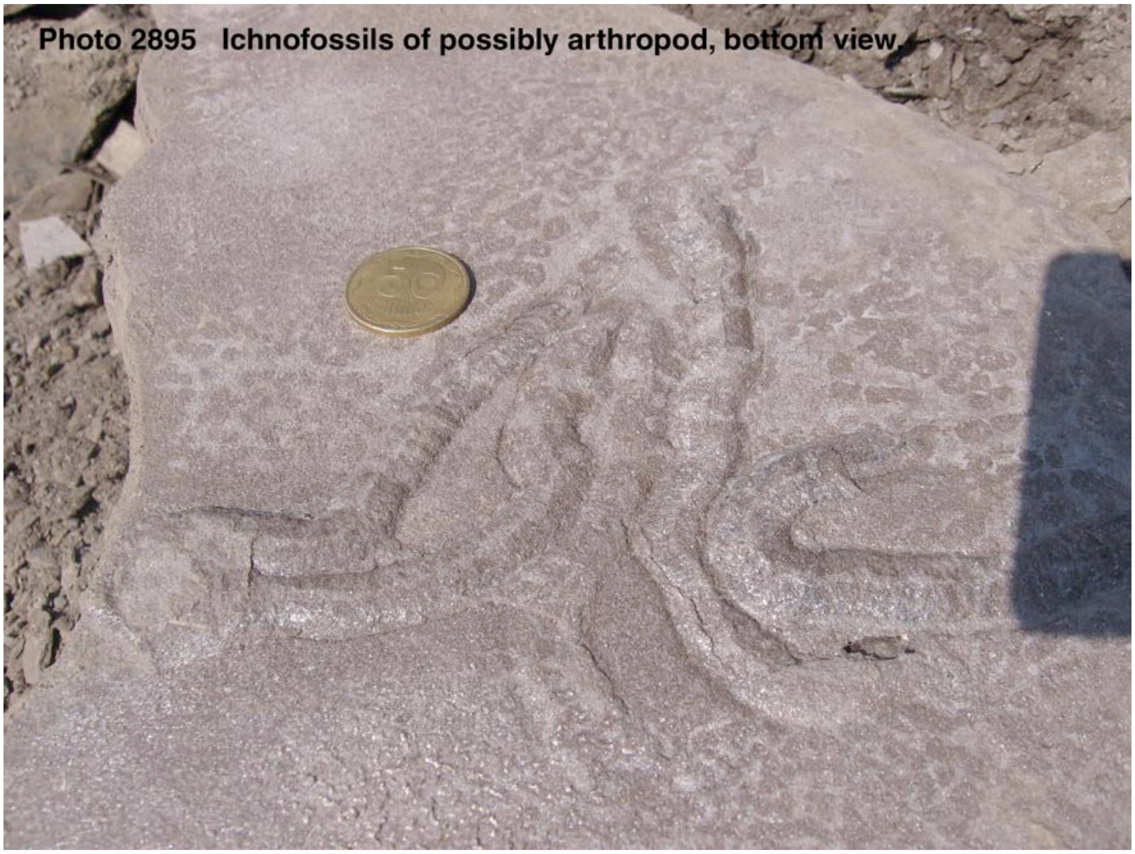

Photo 2895 Ichnofossils of possibly arthropod, bottom view.

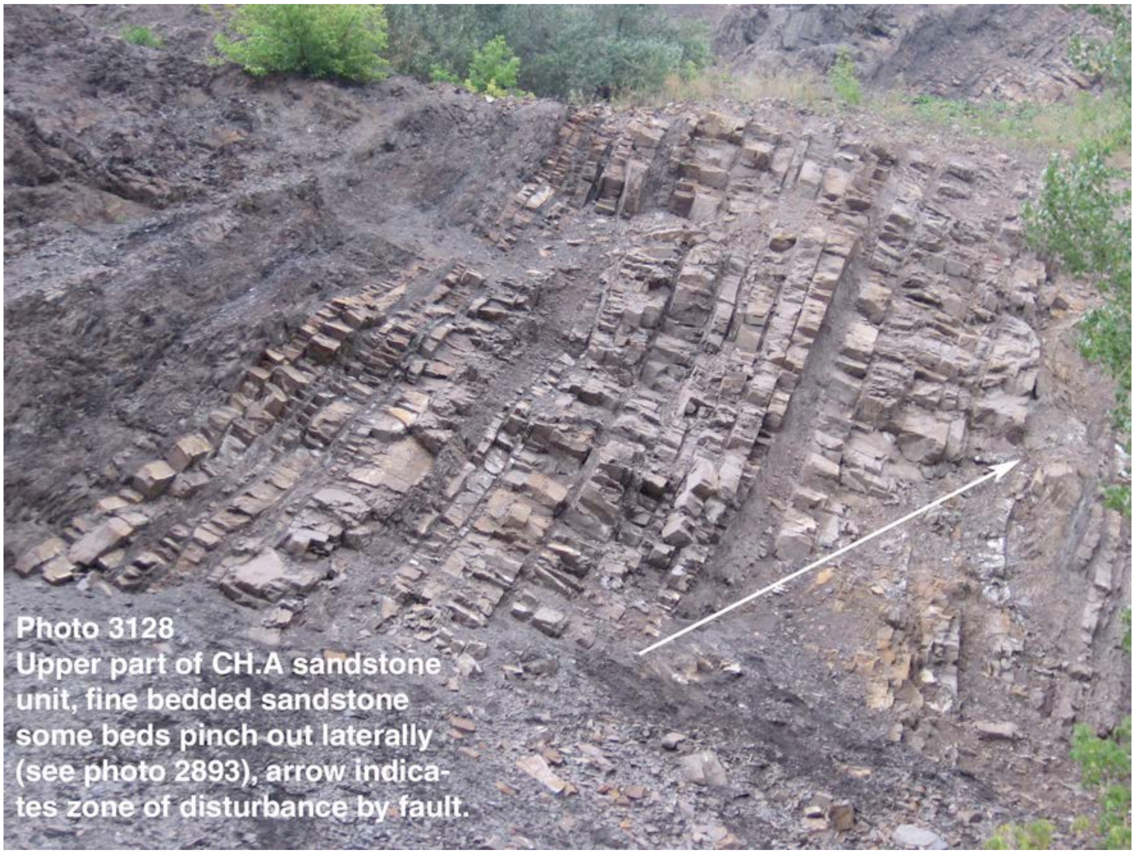
Photo 3128
Upper part of CH.A sandstone unit, fine bedded sandstone some beds pinch out laterally (see photo 2893), arrow indicates zone of disturbance by fault.

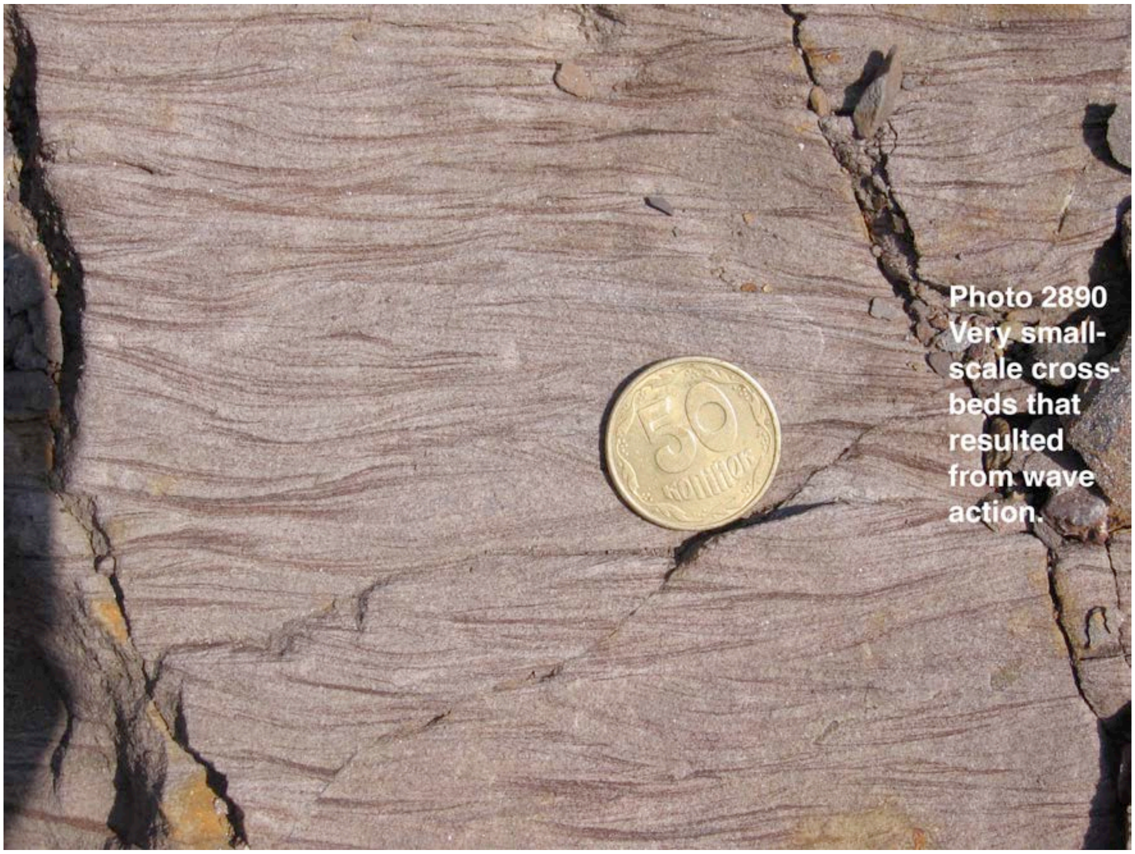
Photo 2890
Very small-scale cross-beds that resulted from wave action.
50
копійок

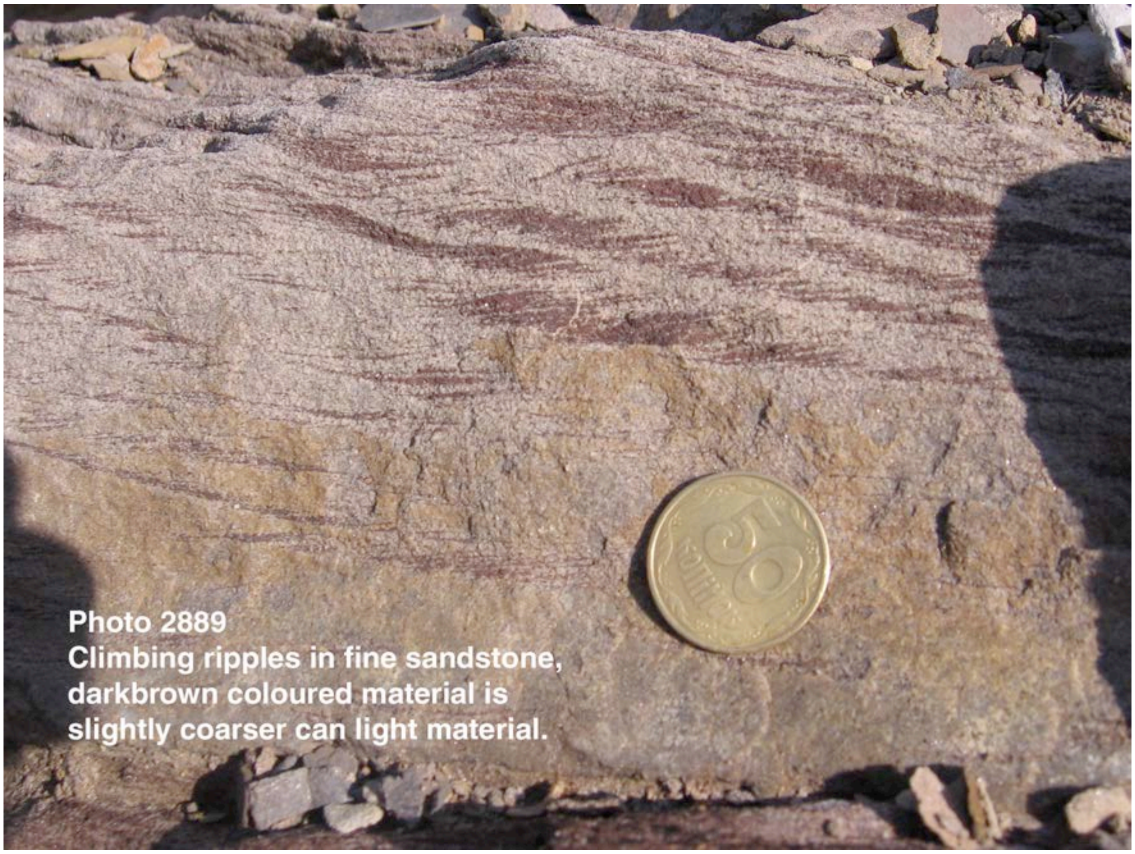
Photo 2889
Climbing ripples in fine sandstone,
darkbrown coloured material is
slightly coarser can light material.

Section: ***Fashchivka***

Location: 37U 0472949 UTM 5339402, central area

Situation: The Fashchivka section is named after the village called Фащевка (the one south of M-03), that is situated just northeast of the section. The section can be reached by taking the road to the east from a statue of Lenin at the village's main square and following that road to south and southwest for about 3 km, also outside the village.

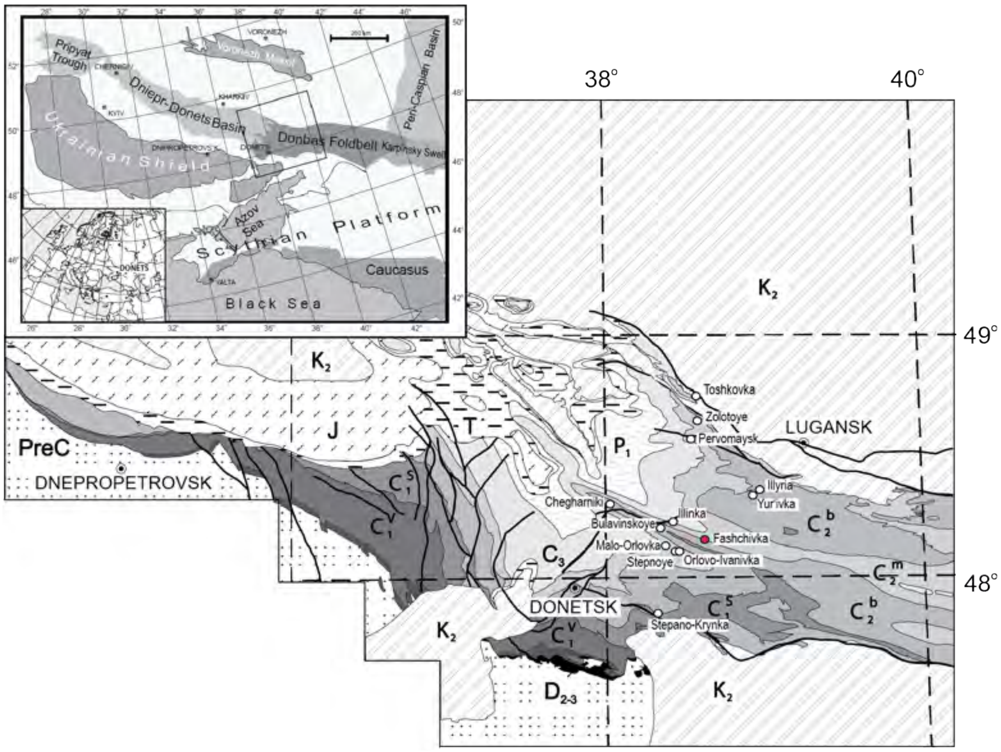

Age: **upper Moscovian, C_2^7**, around limestone M_7

% Sand: **37 %**

Thickness: **109 m**

Sedimentology:

The Fashchivka section is characterised by fairly prominent sandstone units intercalated in shales and silts. Half of these units are well-sorted fine to middle sandstones with current induced intermediate scale (~25 cm) cross-beds and some indications for hummocky cross-stratification. These belong to Group C. The basal and top unit consists of poorly sorted coarse sandstones with intervals showing a massive character and tree trunk prints, and intervals with large scale current induced cross-beds (~ 50-100 cm) to variable directions. These sandstones (F.A and F.C) belong to Group A. These units show fining upwards, with massive intervals in the base and more developed current induced cross-beds to the top. All units show an average paleocurrent direction to the southeast, with almost 180 degrees variability (Figure XF). No clear difference in paleocurrent direction have been measured between the two distinguished

sandstone types of Group C in the middle of the section and Group A at the base and in the top.

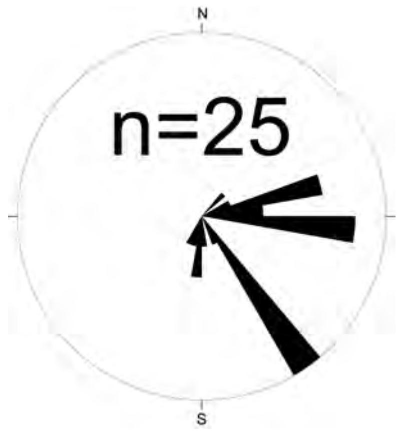

Figure XF. Rose diagram of all measured paleocurrent directions in the Fashchivka section. Petals in groups of 10°, largest petal has 6 measurements and represents 24% of the total.

Environmental interpretation:

The coarse poorly sorted sandstones that belong to Group A are interpreted as fluvial deposits. From all studied sections sandstone unit F.C has the best developed fluvial current induced cross bedding at its top (PHOTO 3329). This interval is seen as a prolonged interval of fluvial environment. The finer better sorted sandstones are interpreted as middle shoreface deposits according to the interpretation of Group C. We interpret the silts and shales as open deeper water deposits.

MACROSCOPIC DESCRIPTION of FASHCHIVKA section

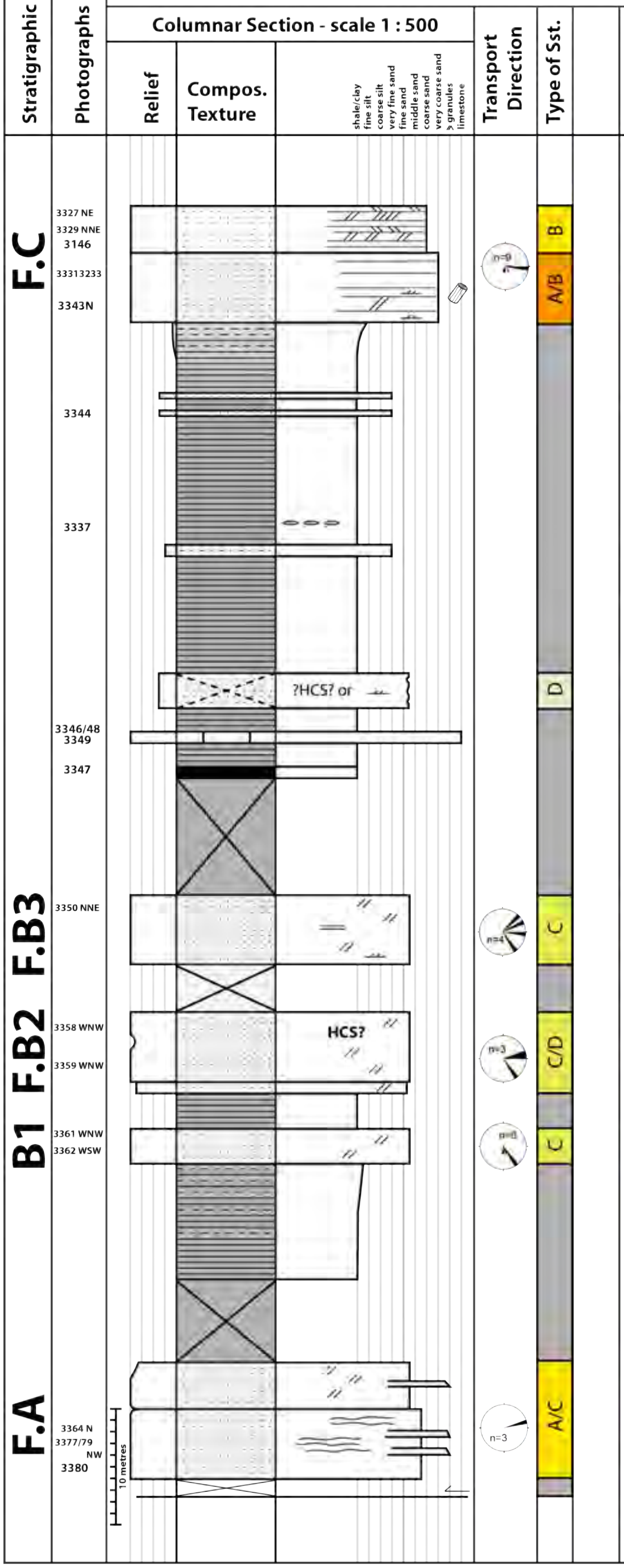

Additional DESCRIPTION and remarks

Top of Section Some outcrops upwards present.

Coarse sandstone unit, badly sorted (fine to very coarse), almost no grading, unit completely built up of mega-foresets of height ~50 cm, fast changing flow directions, short or no toe-sets, individual foreset laminae thickness 2-4 cm --> due to clay?, cut off planes ~ horizontal, no concave cut-off planes, no point bars. Qtz (some greenish), hornblende, feldspar; bit variable percentages through unit. Lower part: coarser unit with beds built up of small foresets of height 5 cm, badly sorted, grains up to granule, tree trunks.

Fine well-sorted sand beds, with horizontal lamination of lower plane bed, BP 080/12 N

Shell fragment bearing shale with iron chert (siderite) nodules

mica-rich

upper part of the section logged 750 metre to east with respect to lower part

Moderately to badly outcropping sandstone unit with some cross-beds that most probably relate to HCS, but this cannot be fully verified, otherwise current flow is to E and W.
LIMESTONE M7: in outcrop discontinuous, a bound/packstone of stromatopores with limestone-debris; crinoids, shell fragments and complete shells (ostracods? --> thinly shelled), and corals. All bioclasts small --> brackish lagoon?
COAL: sample called 'COAL BELOW M7'

Mega-cross bedded fine to middle sandstone unit, X-beds current induced, set height ~25 cm, well-sorted, no grading, mineralogical content quartz, feldspar and black minerals, BP 088/07 N and 090/06 N

Sandy non-outcrop due to recent debris from unit above

Fine to middle sandstone unit with some concave cut-offs in top part and mega-foresets in lower, sedimentary structures difficult to examine, foreset height 25-50 cm, no grading, well sorted, hard quartzite, too hard to drill

Foreset height 20 cm, all flow directions ~ in same direction

Fine to middle sandstone unit, well-sorted, cross-bedded, set boundaries horizontal, flow directions dominantly to SE, but variable, no grading, BP 088/10 N

Fine to middle sandstone, quite well-sorted but layers with small-pebble sized grains, those badly sorted, mega foresets allover of height 25-50 cm, all flow to ~NE BP 100/10 N
Middle sandstone, badly sorted, fine to granules grains, apparent horizontal chaotic layering (photos), no grading, reworked clay pebbles, no wood found (expected) --> fluvial sandstone?

Fault with apparant strike-slip movement, most probably dextral movement, ~fault plane 120/46 NNE (joints)
End of Section Below fault a sandstone with character similar to F.B unit, but it is not F.B unit, outcrop possibly continues further downwards.

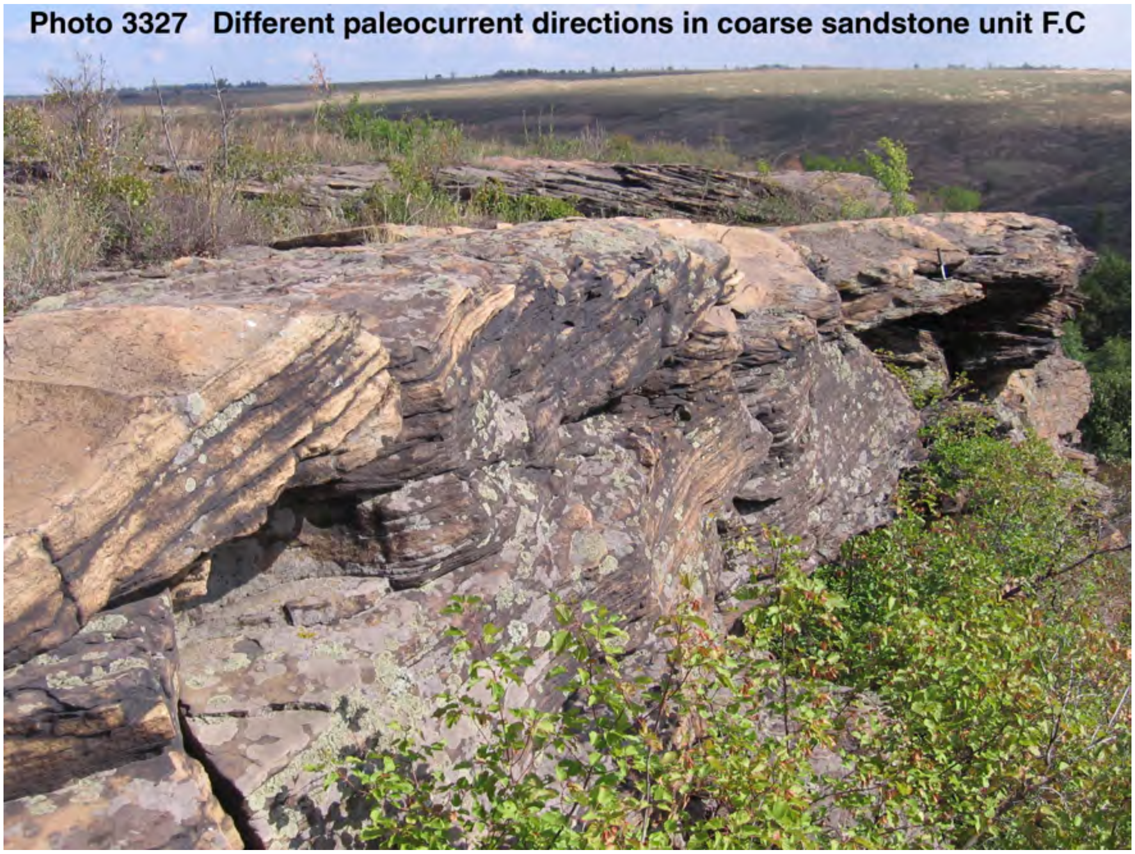

Photo 3327 Different paleocurrent directions in coarse sandstone unit F.C

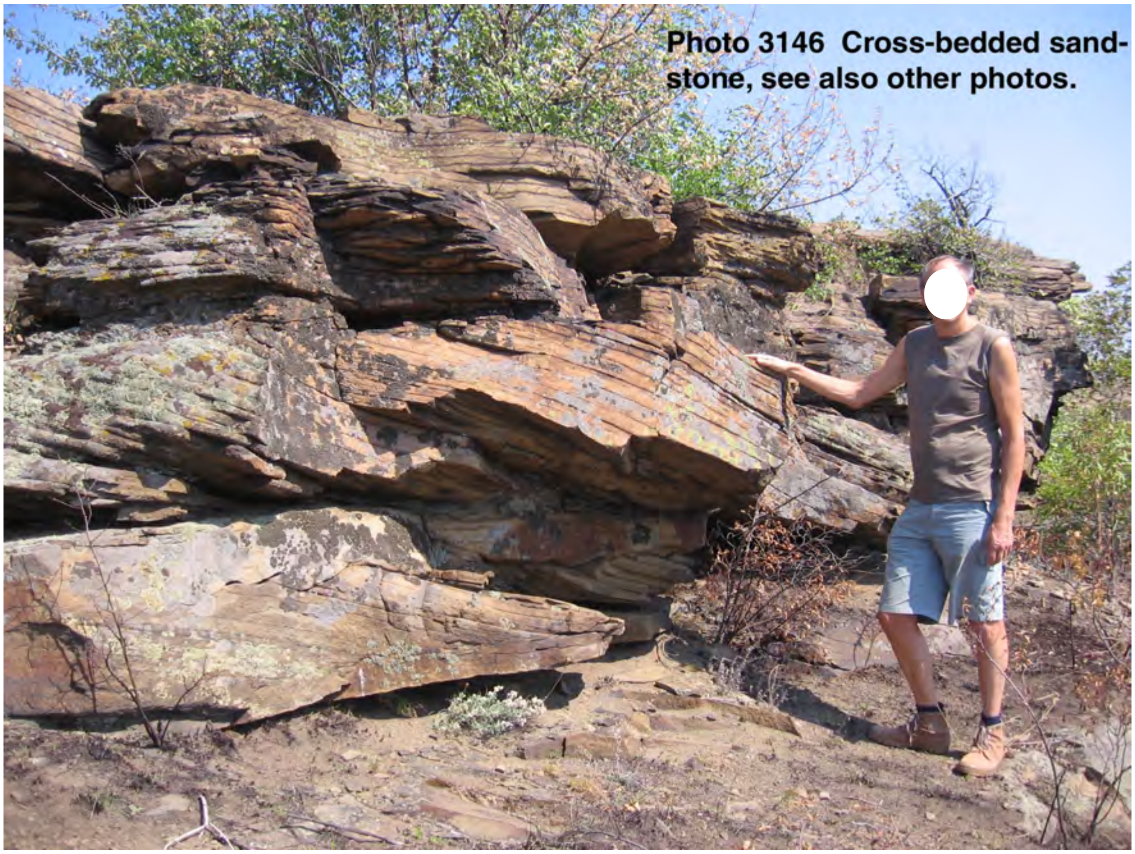

Photo 3146 Cross-bedded sandstone, see also other photos.

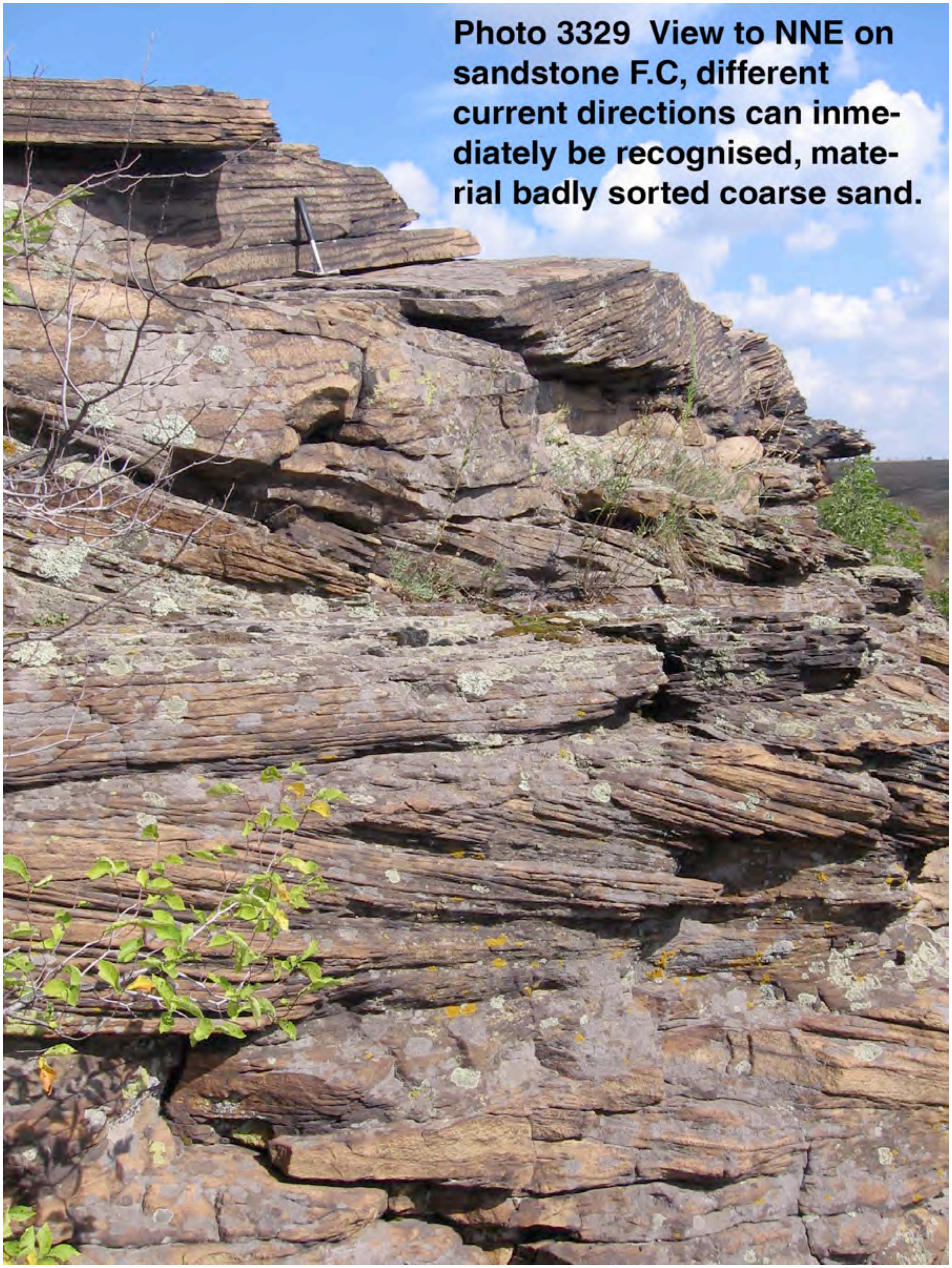
Photo 3329 View to NNE on sandstone F.C, different current directions can inme-diately be recognised, mate-rial badly sorted coarse sand.

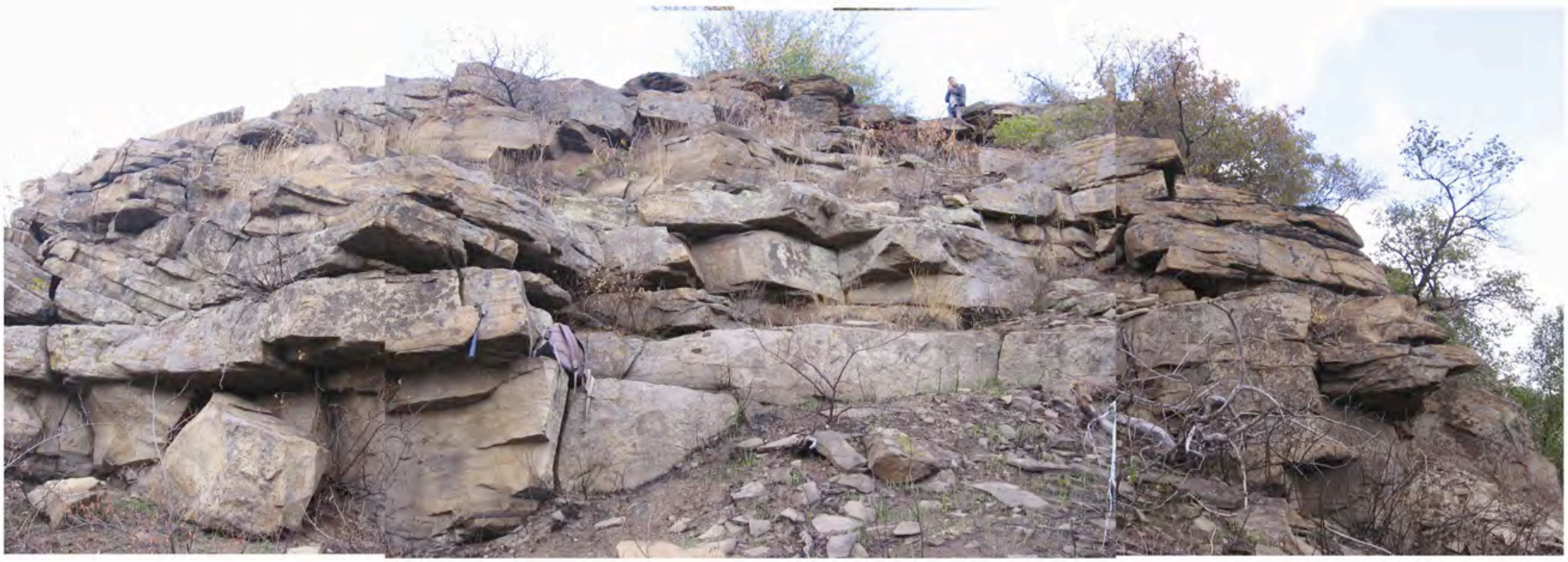

Photo 3331/32/33 View to north of lower part sandstone F.C, just above hammer mega-foreset can be seen, other beds massive and apparent structure-less.

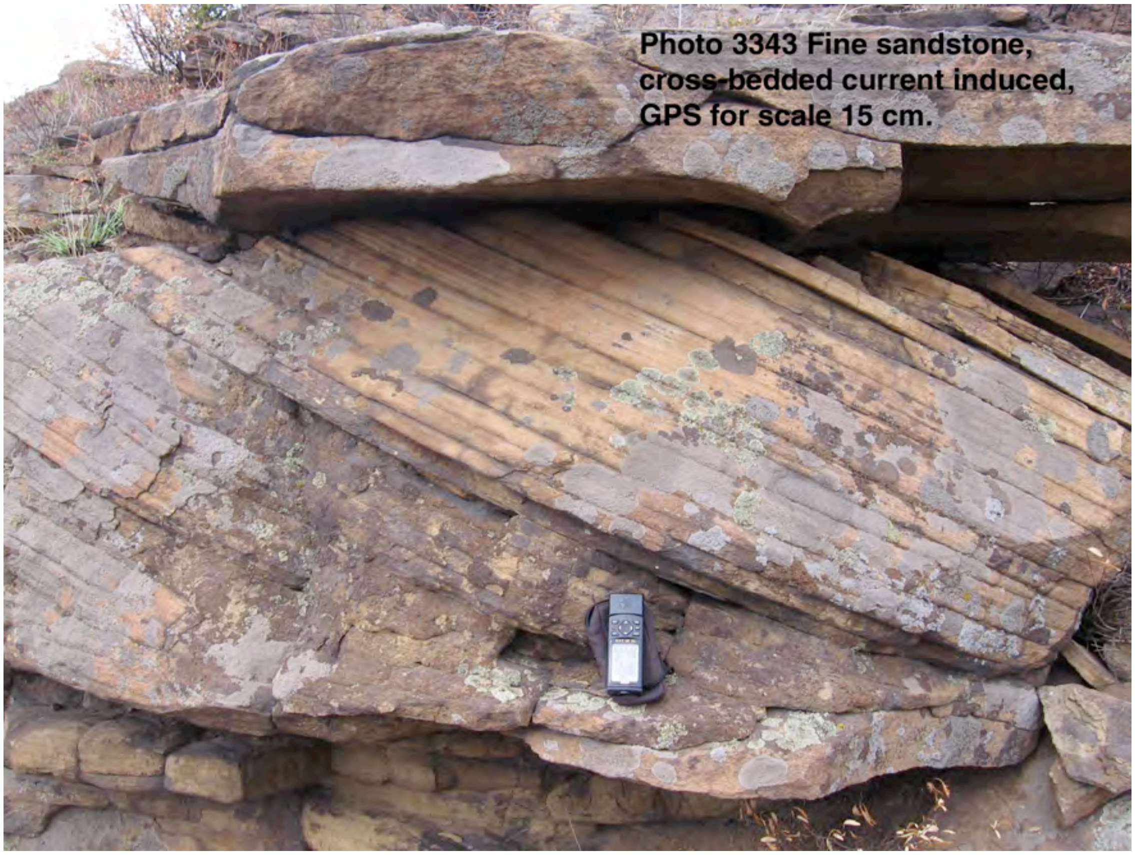

Photo 3343 Fine sandstone, cross-bedded current induced, GPS for scale 15 cm.

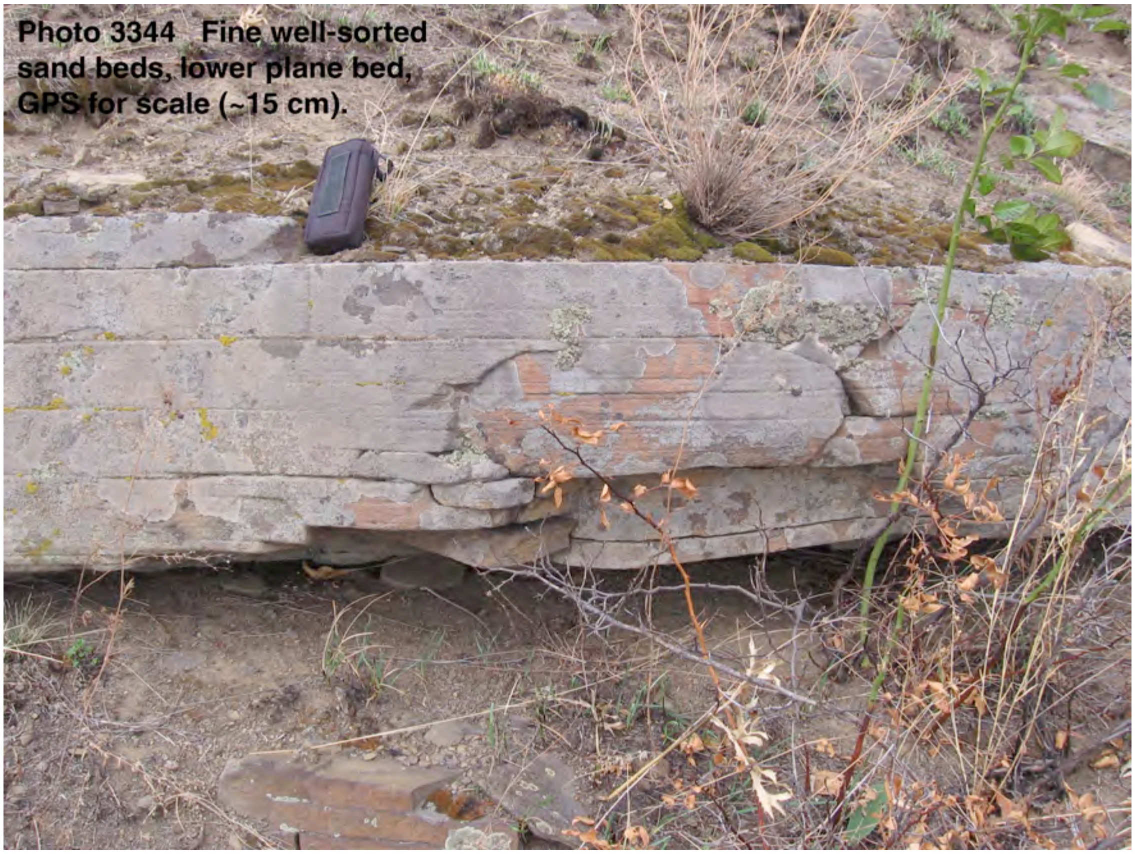

Photo 3344 Fine well-sorted sand beds, lower plane bed, GPS for scale (~15 cm).

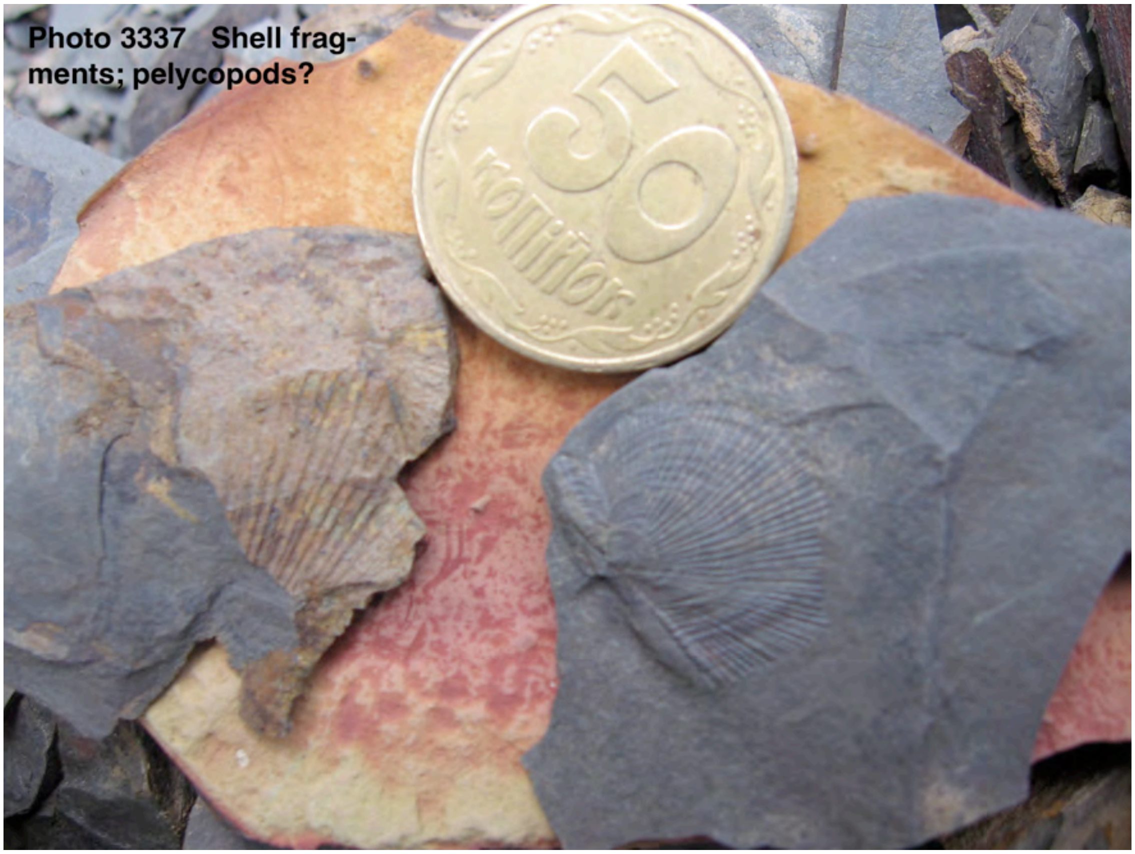

Photo 3337 Shell fragments; pelycopods?

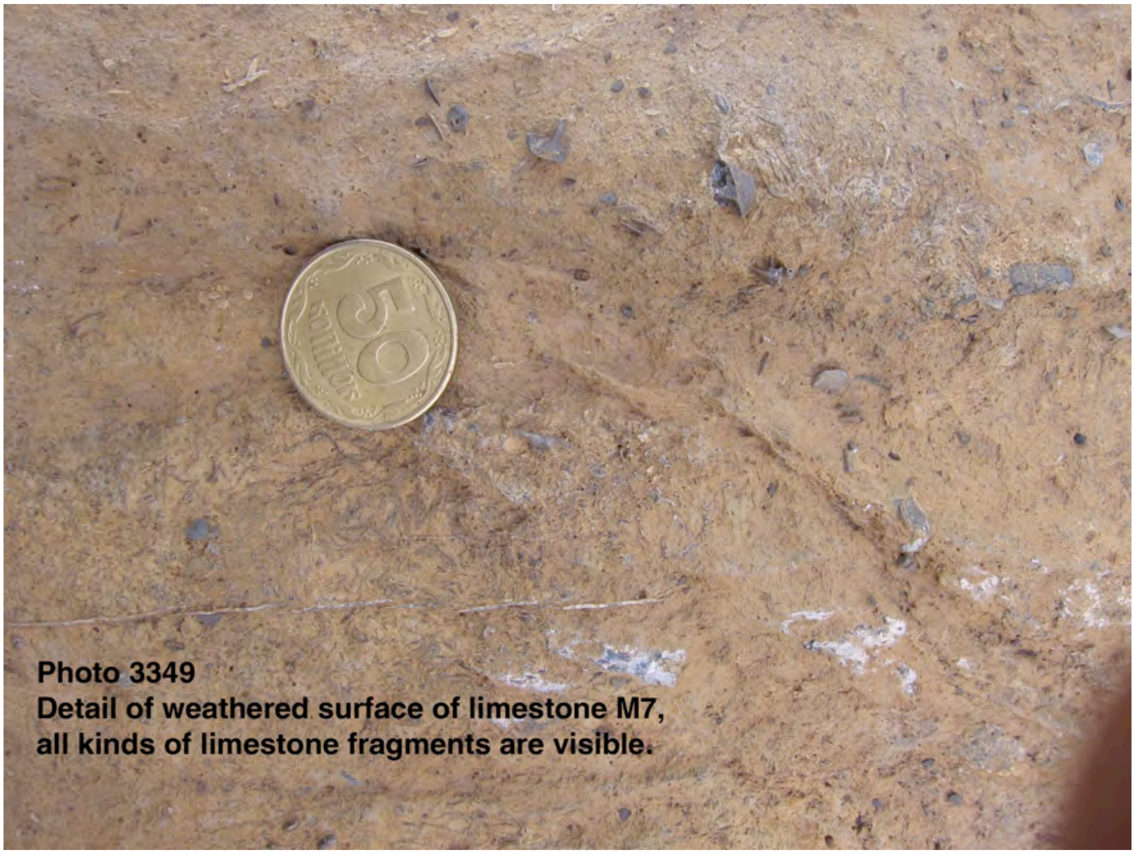

Photo 3349
Detail of weathered surface of limestone M7, all kinds of limestone fragments are visible.

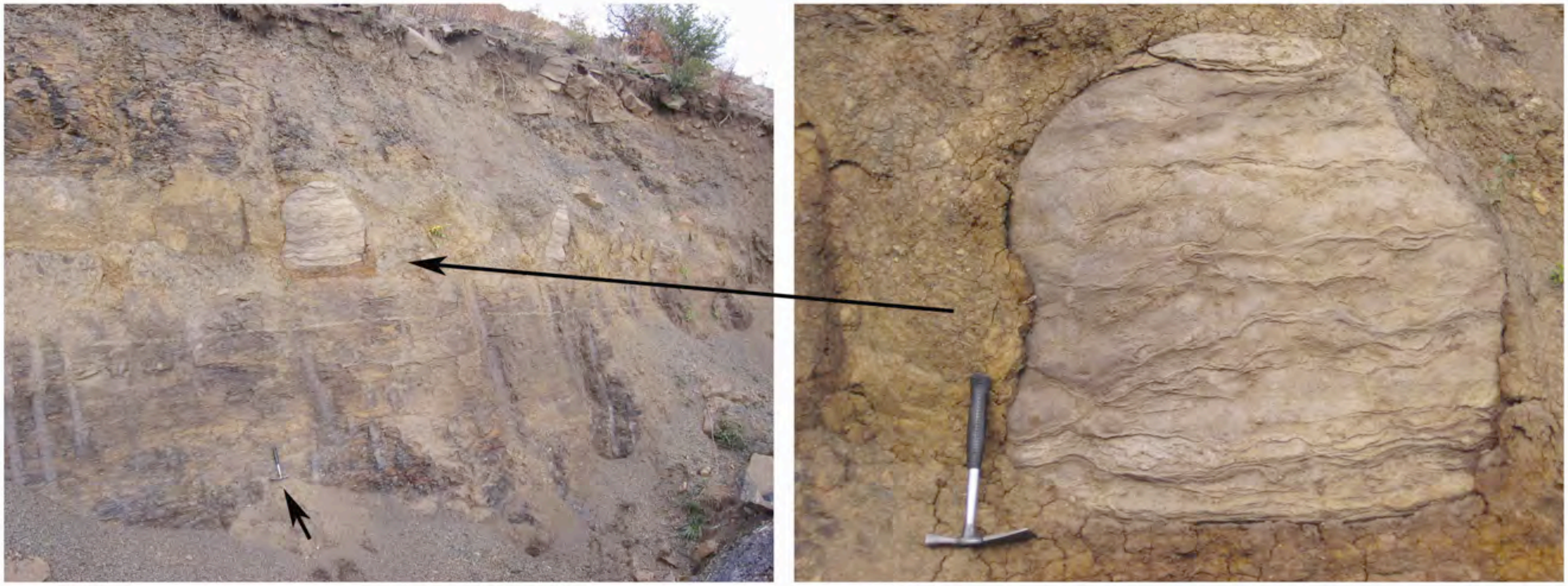

Photo 3346 (left) and 3348 Shale interval with variably outcropping limestone bed, detail of limestone on right photo, limestone bed and places where bed is not outcropping the level is capped by horizontal layering, so no syn-sedimentary process is expected to be the cause for the strange outcrop, at base of left photo "coal below M7" is positioned, hammer for scale.

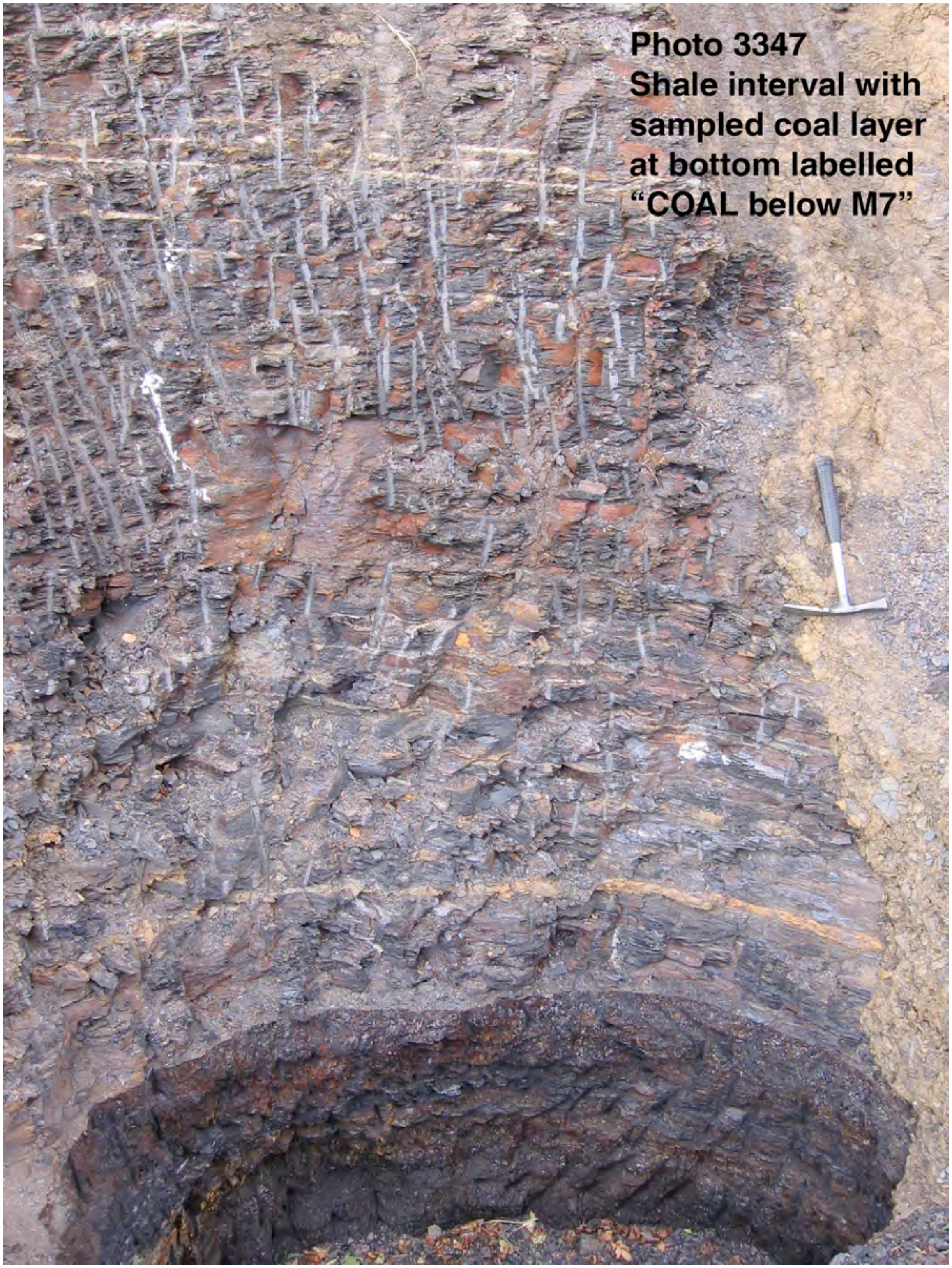
Photo 3347
Shale interval with
sampled coal layer
at bottom labelled
“COAL below M7”

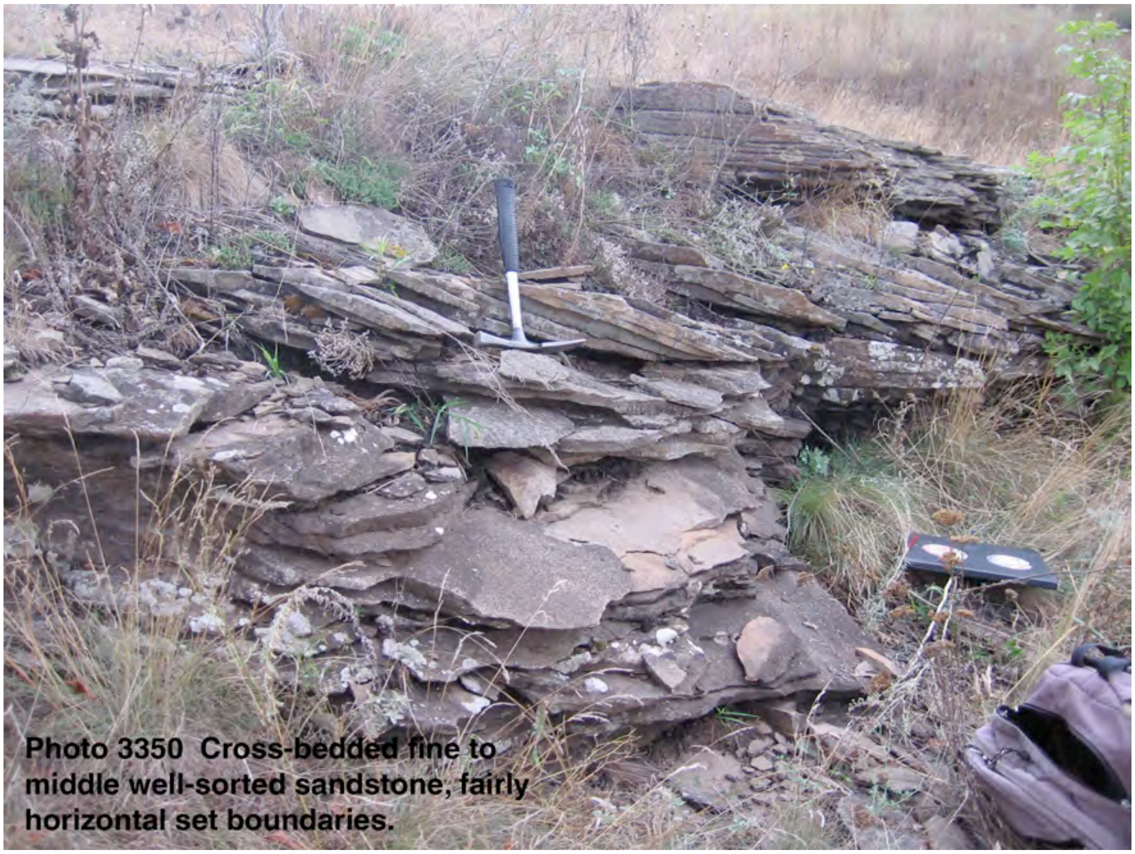

Photo 3350 Cross-bedded fine to middle well-sorted sandstone, fairly horizontal set boundaries.

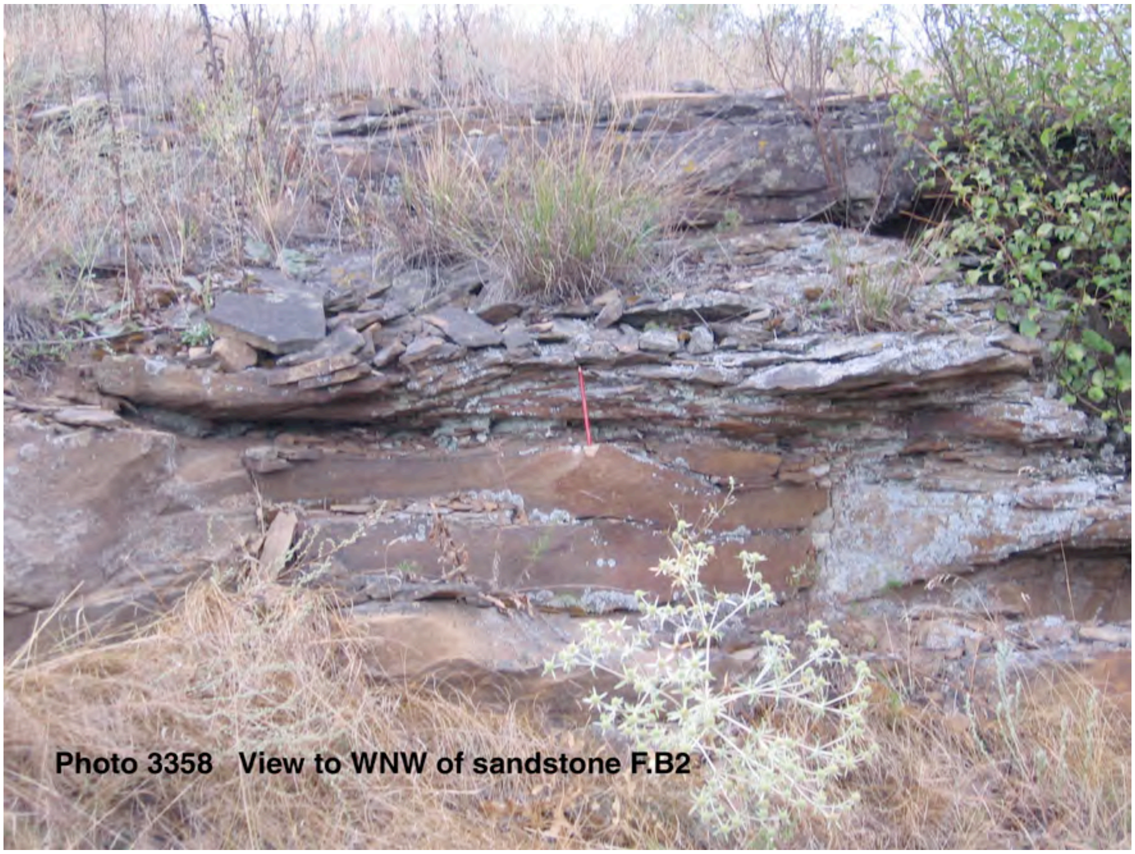

Photo 3358 View to WNW of sandstone F.B2

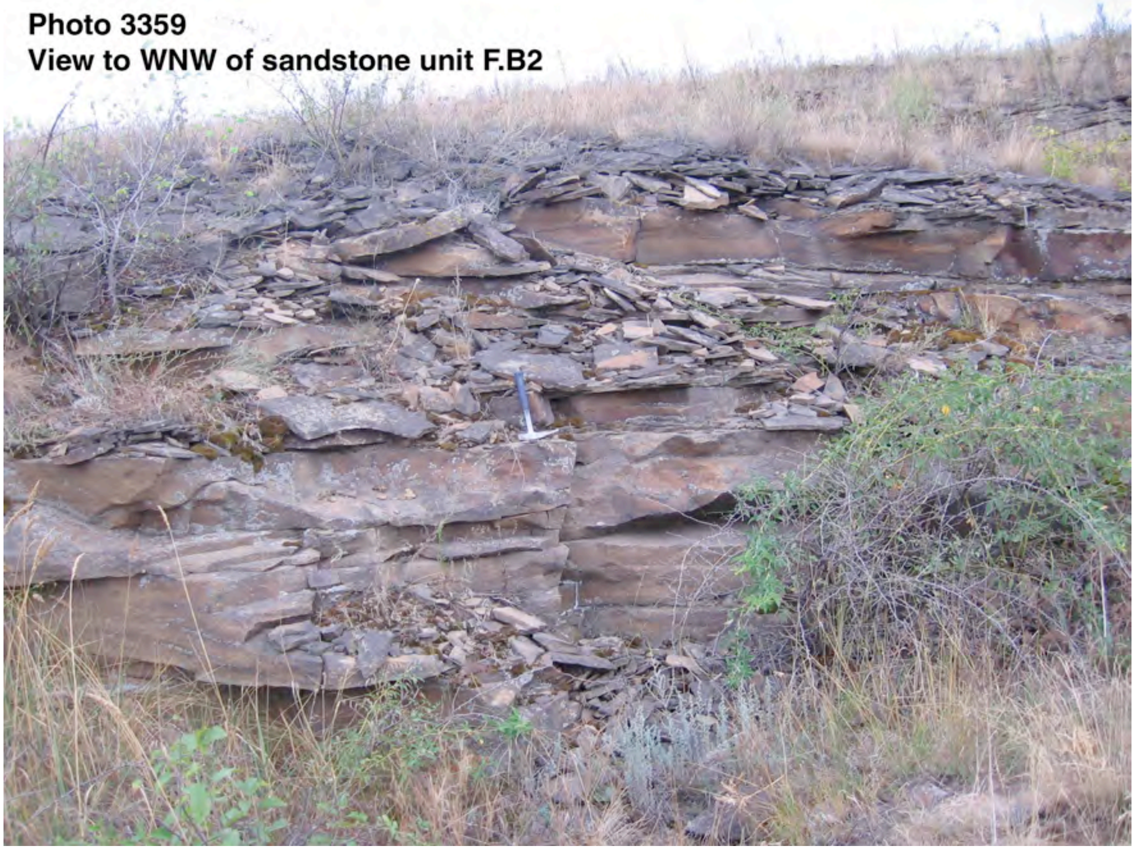
Photo 3359
View to WNW of sandstone unit F.B2

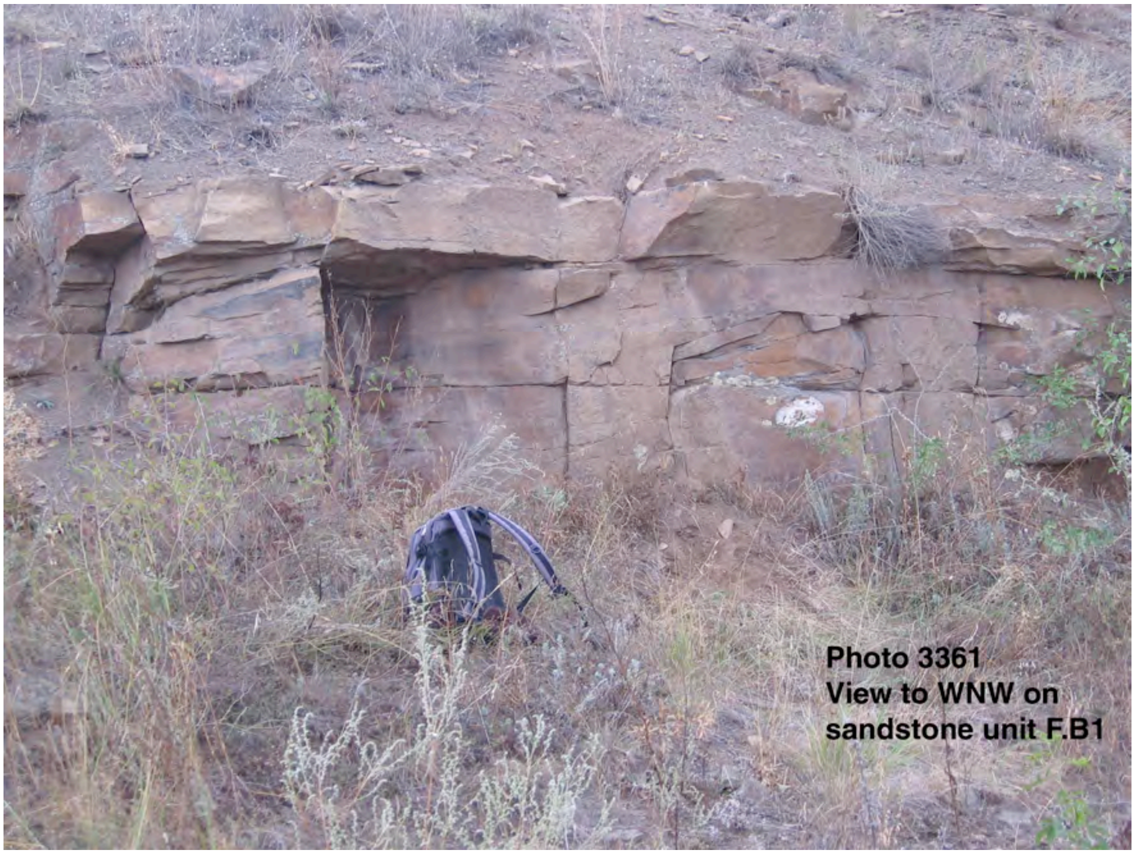
Photo 3361
View to WNW on
sandstone unit F.B1

Photo 3362 View to WSW on sandstone unit F.B1

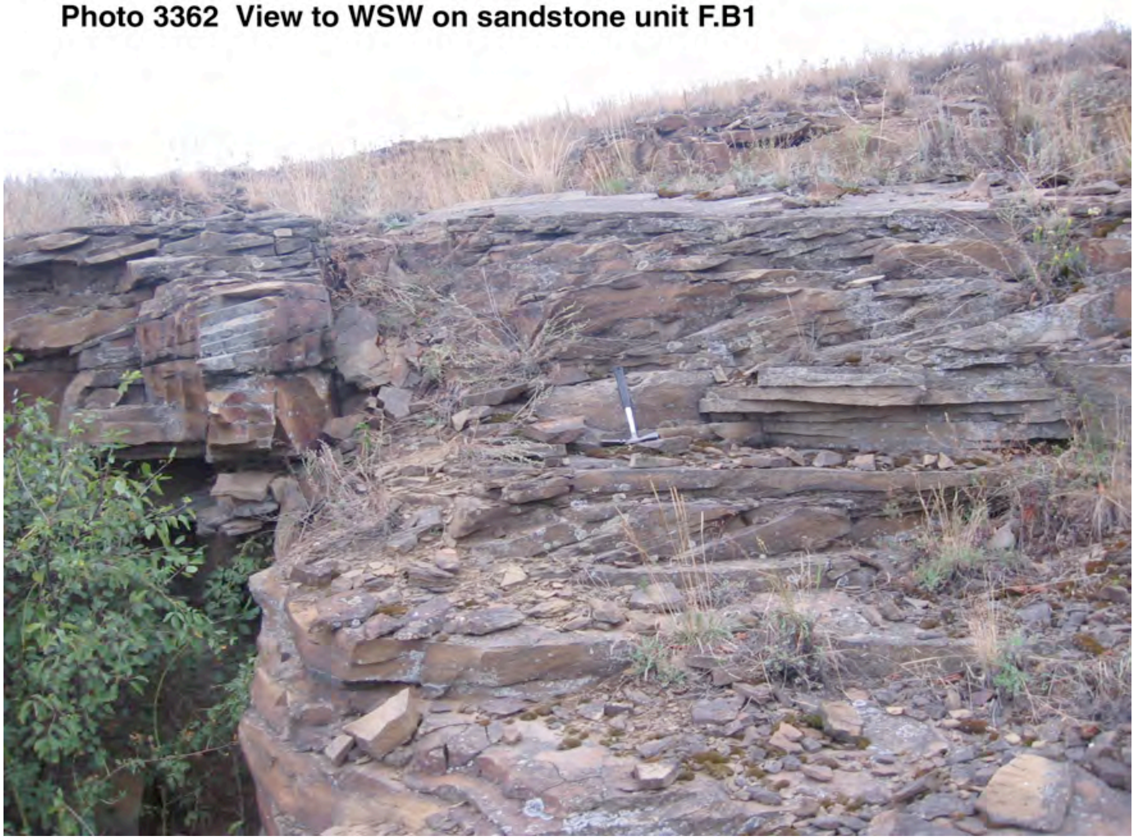

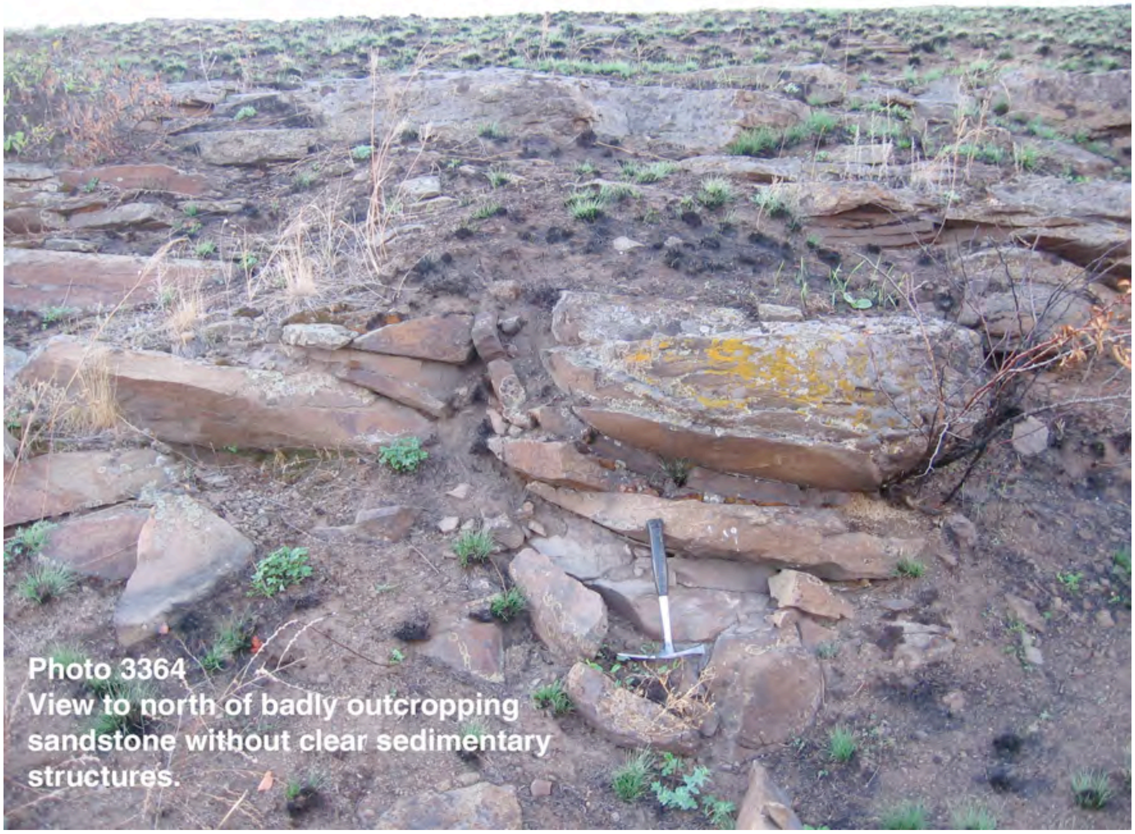

Photo 3364
View to north of badly outcropping sandstone without clear sedimentary structures.

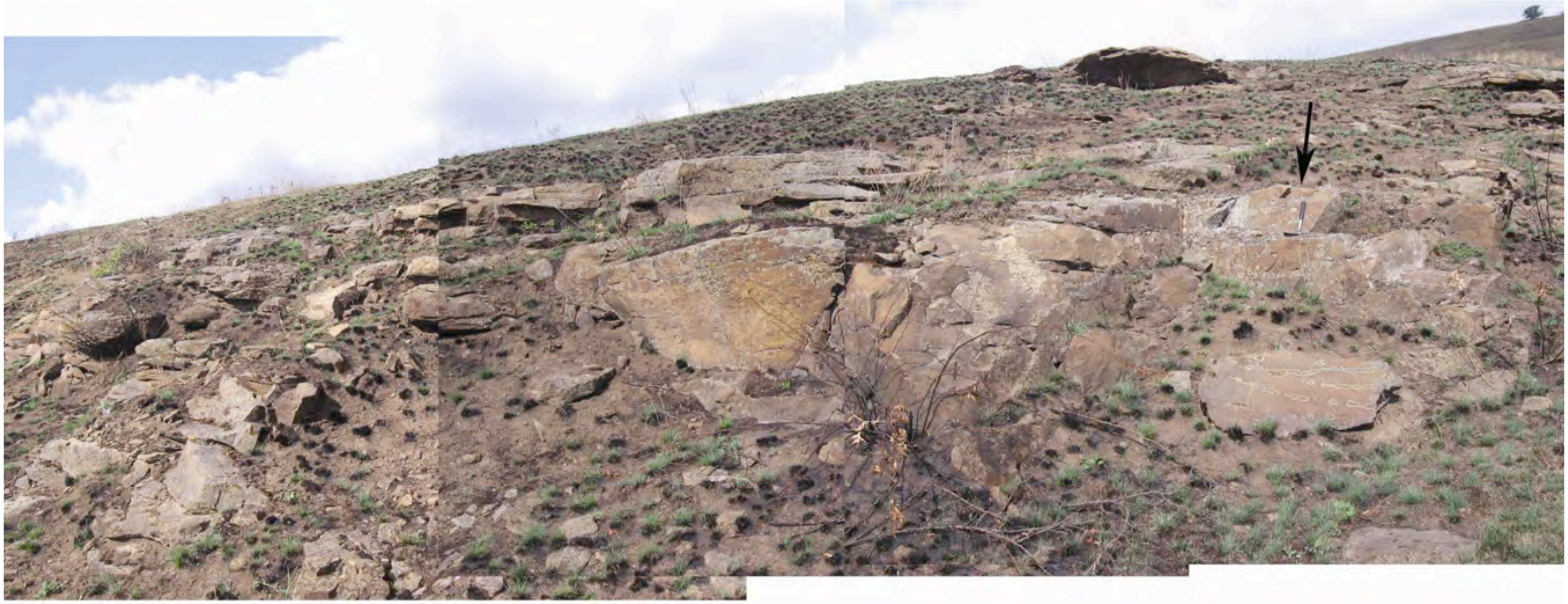

Photo 3377/78/79 Bad outcrop of massive structureless sandstone, hammer for scale.

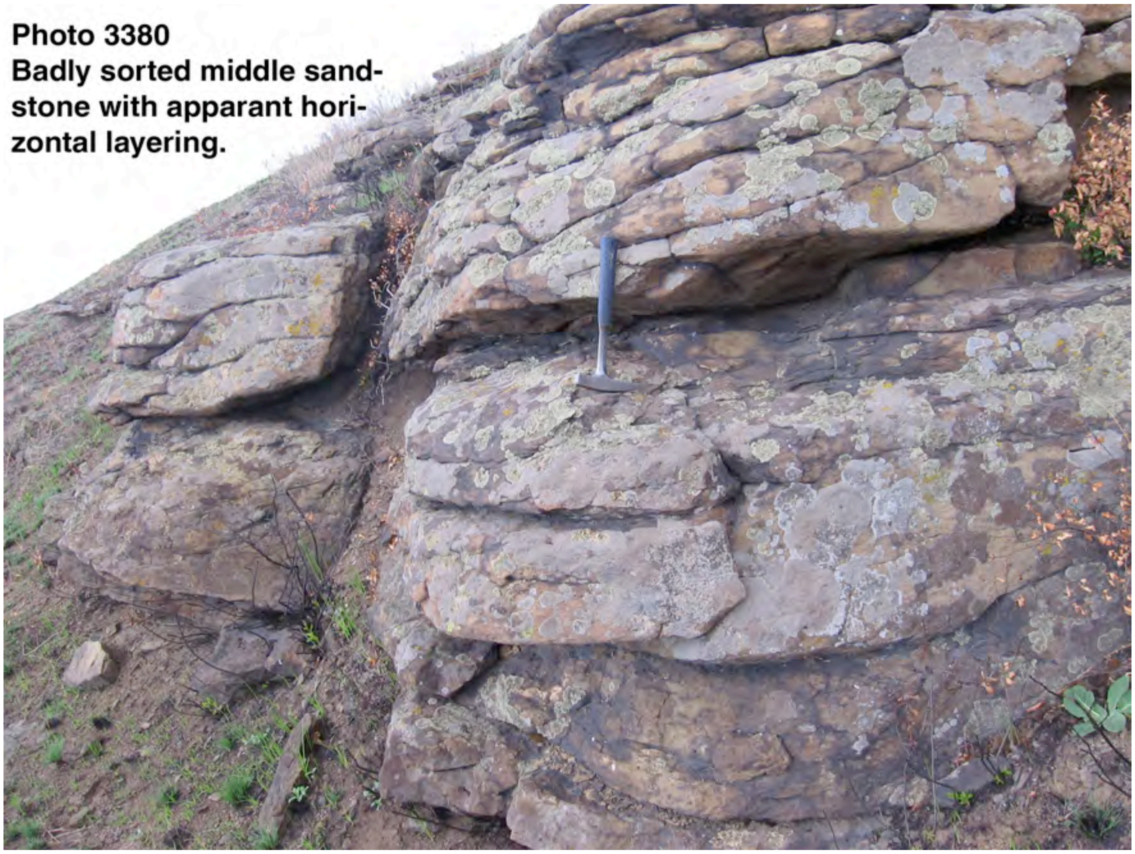
Photo 3380
Badly sorted middle sand-
stone with apparent hori-
zontal layering.

Section: ***Illinka***

Location: 37U 0457528 UTM 5347233, central area

Situation: Illinka sandstone is named after the village Ильцнка (Illinka). The sandstone can be found along a small footpath that starts to the west at the point in the northern end of the village, where the paved road ends.

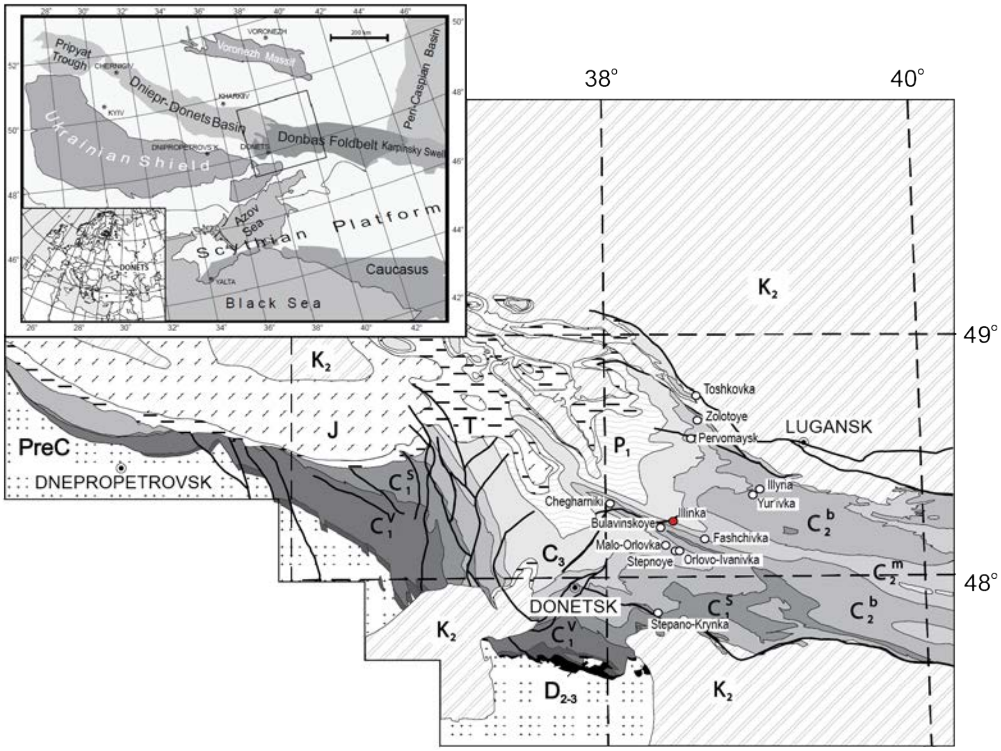

Age: **upper Moscovian, C_3^1**, below limestone O_1

% Sand: **n / a**

Thickness: **8 m**

Sedimentology:

The Illinka sandstone is not directly fitting into one of the defined sandstone groups. This is because the sandstone consists of very coarse and poorly sorted grains, but show very well-developed current induced large scale cross bedding. None of the defined groups shows these characteristics, however they come closest to a mixture of Group A and B. The basal and top parts of the sandstone show slightly better sorting and finer material. Also in the top trough cross beds occur. Paleocurrent analysis shows a fairly consistent flow to the east-northeast with around 90° of variation. Above the sandstone outcrop there is a shale interval and a thick limestone. Downwards a few sandstones have been found also showing huge lateral continuity. The whole interval is not very sandstone rich.

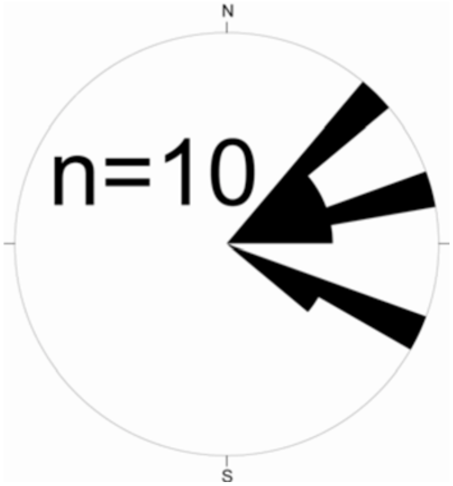

Figure XIL. Rose diagram of all measured paleocurrent directions in the Illinka section. Petals in groups of 10°, largest petal has 2 measurements and represents 20% of the total.

Environmental interpretation:

The depositional environment of the Illinka sandstone is interpreted to have been relatively close to the coast. This is inferred from the poor sorting, (very) coarse grains, and large scale current induced cross bedding. However no signs for a well-developed fluvial system is found, because there is for example no large scale trough cross bedding. Also in the basal and top parts indications are present for deeper water environments like from Group B and C. Therefore we prefer an interpretation in which the main environment of the Illinka section is situation very close to the river mouth at the sea-side.

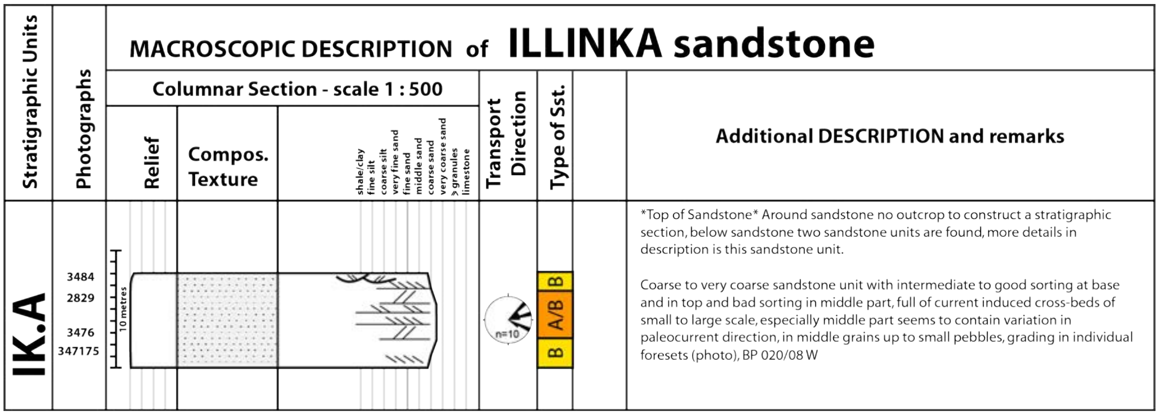

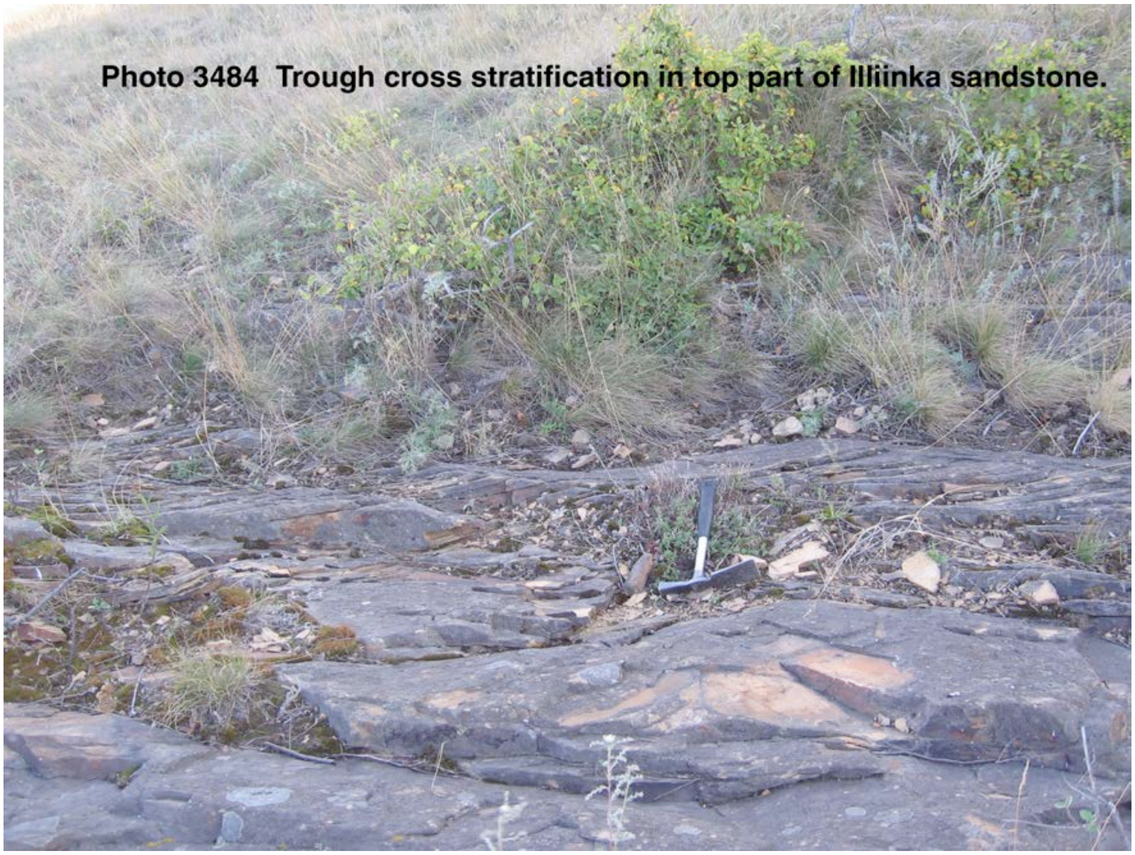

Photo 3484 Trough cross stratification in top part of Illiinka sandstone.

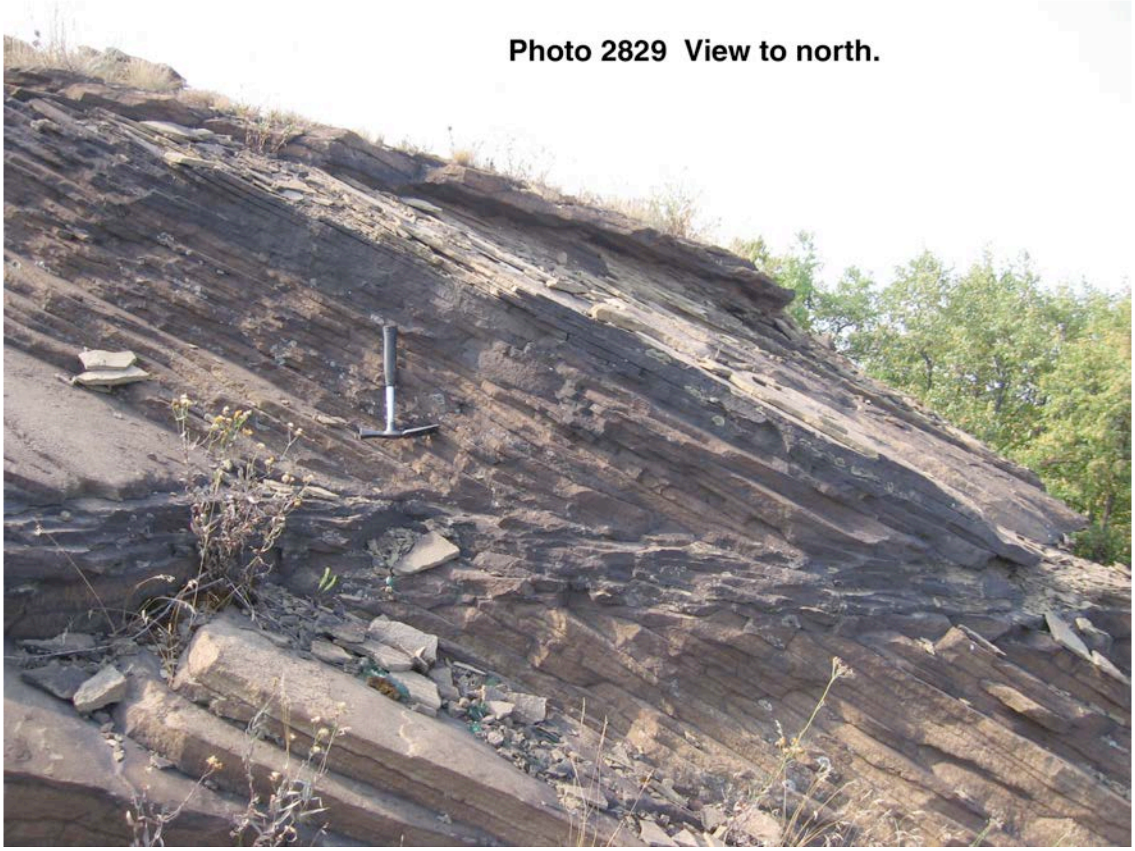

Photo 2829 View to north.

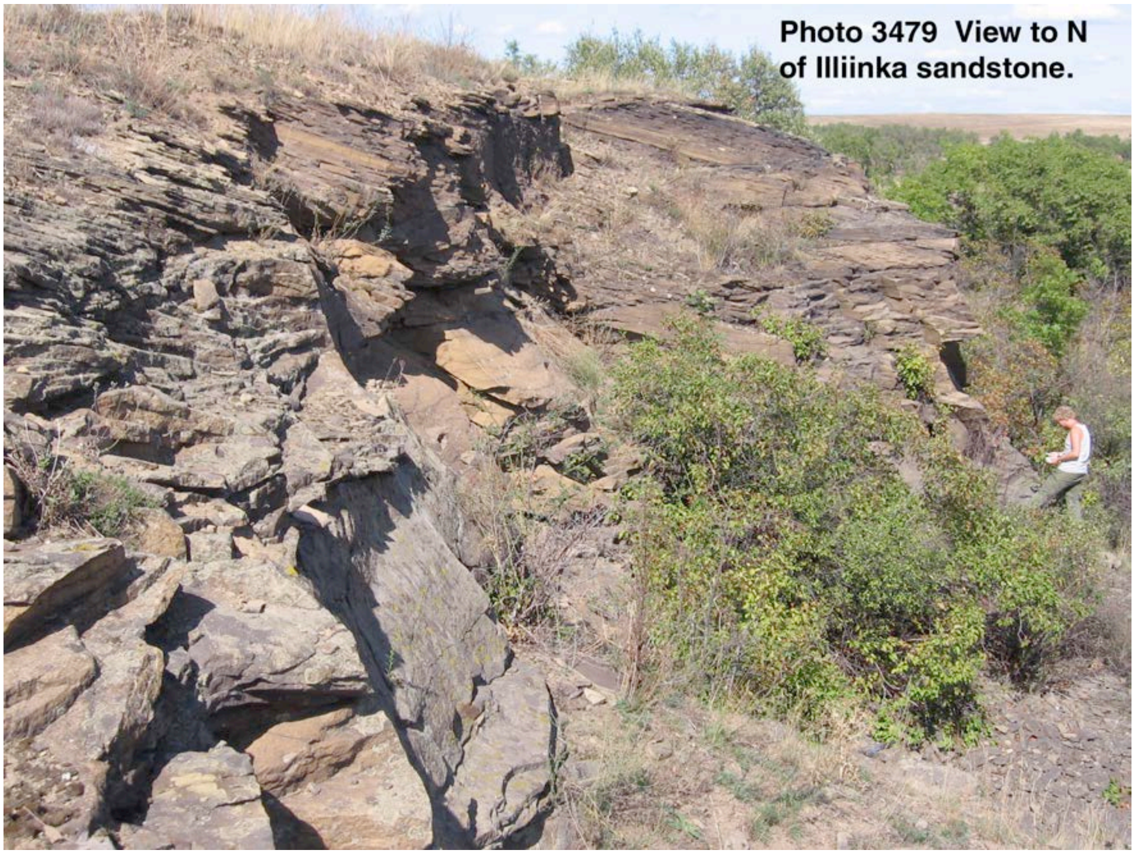

Photo 3479 View to N of Illiinka sandstone.

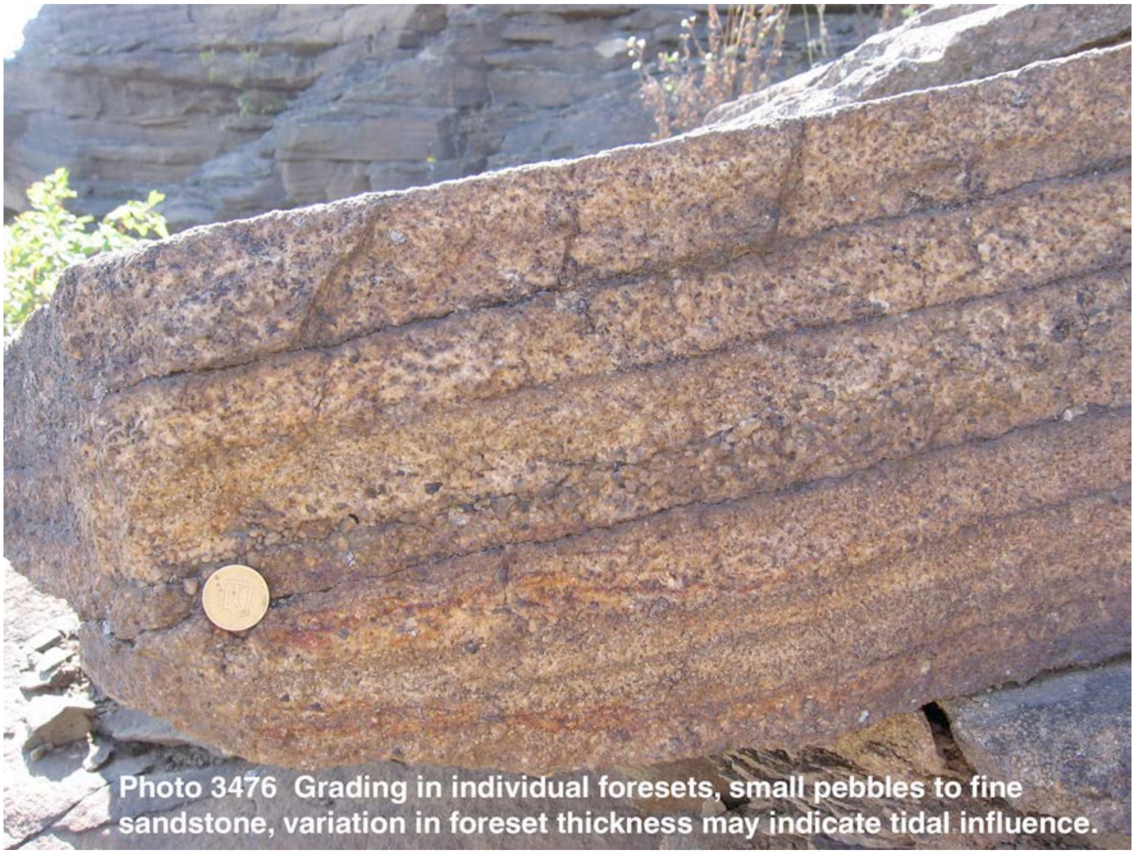

Photo 3476 Grading in individual foresets, small pebbles to fine sandstone, variation in foreset thickness may indicate tidal influence.

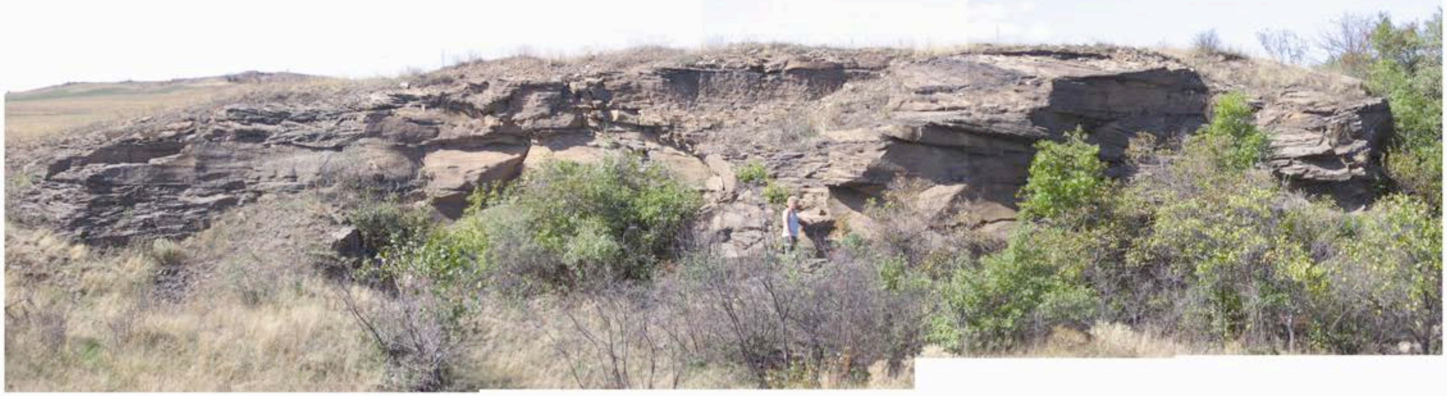

Photo 3471-75   View to west on middle and upper part of ILLIINKA sandstone.

section: ***Illyria***

Location: 37U 0499835 UTM 5361662, northern area

Situation: Illyria section is named after the village Иллирия (Illyria). To reach the section by car from the east, the centre is passed by a turn to the right on a T-crossing, then another turn to the right after passing a bridge. Keep right on the unpaved roads afterwards, but do not cross the water anymore.

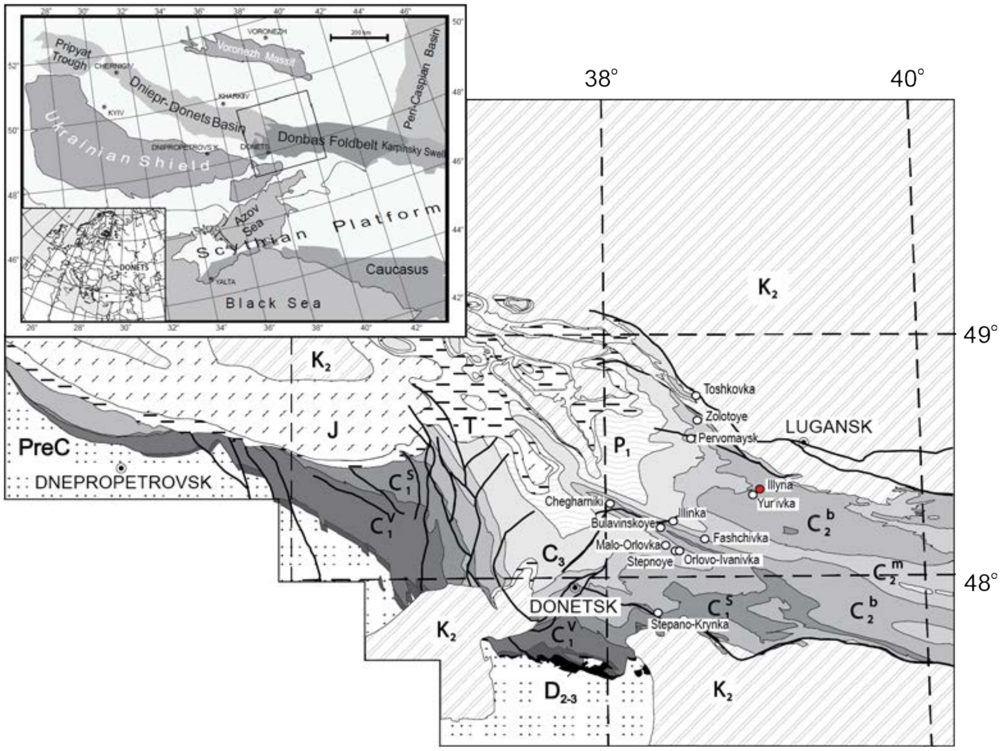

Age: **lower Bashkirian, C_2^2**, sandstone H_1 in top of section

% Sand: **27 %**

Thickness: **167 m**

Sedimentology:

The Illyria section is characterised by a relative high amount of non-outcropping shales and silts. The sandstone outcropping grade is very different for the three units present. The lowermost reveals characteristics of Group A to B in the middle and some of Group C especially at the top, but is also disturbed by diagenesis that makes the observation of sedimentary characteristics difficult. The middle unit shows features of Group C all over with one thin level with Group A in the middle. The uppermost unit show features that are not described in one of the defined groups. It is characterised by fine to middle well sorted sand with large scale swaley cross stratification. This unit is relatively thick and very continuous in outcrop.

Environmental interpretation:

The Illyria sediments show all characteristics for shelf sedimentation (the shales) with intercalations of lower to upper shoreface environments. The two fluvial impulses are not prominent. Swaley cross stratification is usually found in an identical environment as hummocky cross stratification, although more energy is involved. We therefore place the unit that is full of this stratification (IL.C1) in a little higher energetic environment as of Group C, the middle to upper shoreface regime, also because this unit is coarser grained than the normally hummocky cross stratified sandstones.

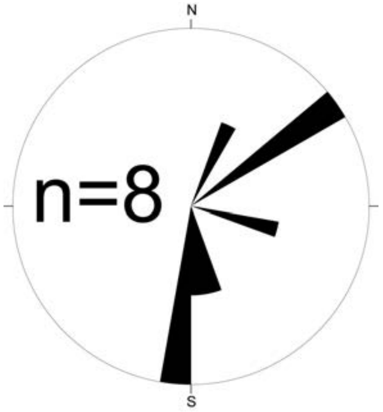

Figure XIK1. Rose diagram of all measured paleocurrent directions in the Illyria section. Petals in groups of 10°, largest petal has 2 measurements and represents 28% of the total.

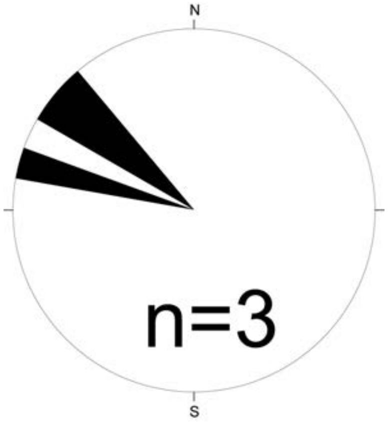

Figure XIK2. Rose diagram of the three measured wave directions in the Illyria sections. Petals in groups of 10°, all petals represent 1 measurement.

MACROSCOPIC DESCRIPTION of ILLYRIA section 1/2

Stratigraphic Units | Photographs | Columnar Section - scale 1 : 500 (Relief; Compos. Texture; shale/clay, fine silt, coarse silt, very fine sand, fine sand, middle sand, coarse sand, very coarse sand, > granules, limestone) | Transport Direction | Type of Sst. | Additional DESCRIPTION and remarks

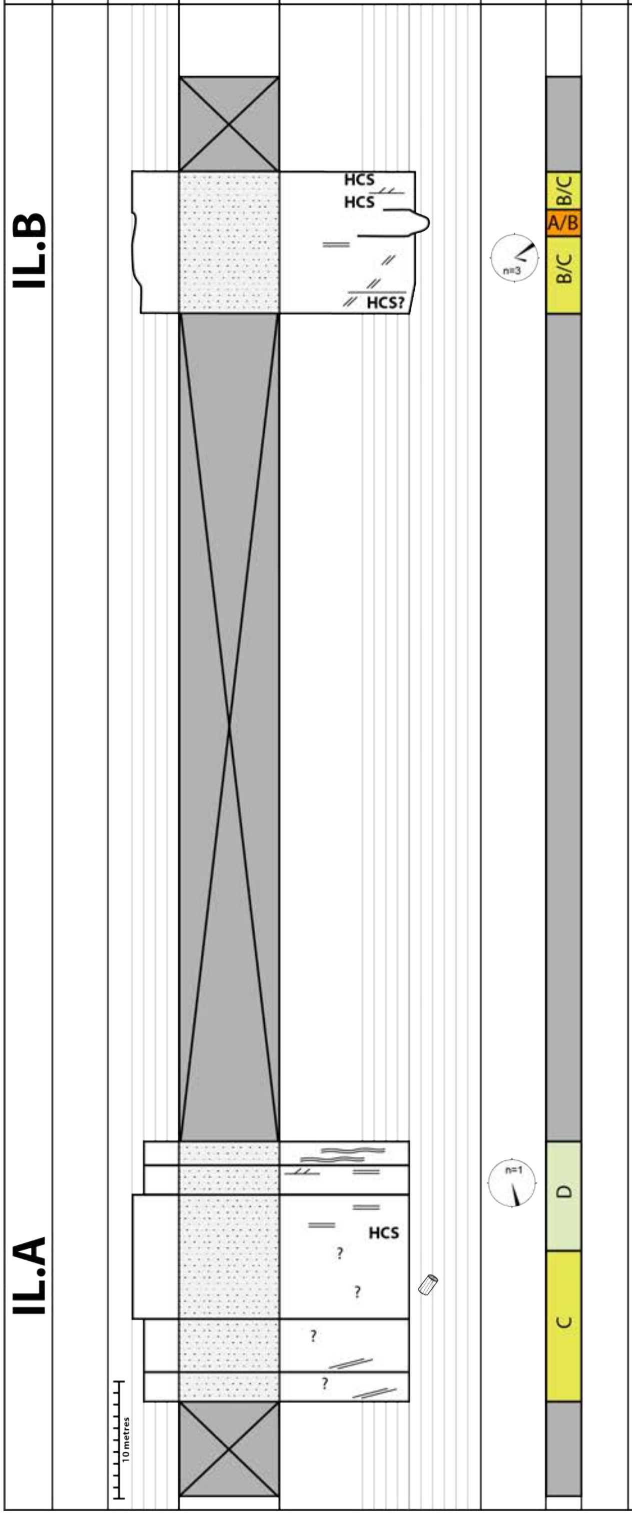

continued on page 2-2

fine to middle sandstone unit with dominant mega-foresets and current rippels up to 70 cm height, but average 20 cm. Two intervals with HCS in top, paleocurrent direction looks a bit variable on outcrop scale. One interval with coarser material without sedimentary structures, less sorted. Qtz, feldspar, little mica. Sorting not clearly visible, most possible OK. In outcrop not very continuous due to a fault or easy weathering.

Bedding plane orientation 057/35 NW and 062/32 NW

Top fine sst unit with wavey-lamination in top not sure if wave or current induced

Fine sst unit with parallel lamination of lower plane bed, laminae thickness <8 cm

Lower interval homogenous with many lines that do not resemble any sedimentary structure, nor structural structure, disturbed by fault zone, lenses of shale, a few places where HCS can be recognised or horizontal lamination, very hard unit, no signs of grading seen. Qtz, feldspar, mica, black mineral, all ~25%, in outcrop this unit fairly contiuous over more than 400 meter

Interval with cross-beds and some HCS, not easy to find paleocurrent directions or detect the HCS, the horizontal lamination or low-angle lamination look like mega HCS.

End of Section More sandstone units stratigraphically below this section can be studied a bit more to the East of Illiria.

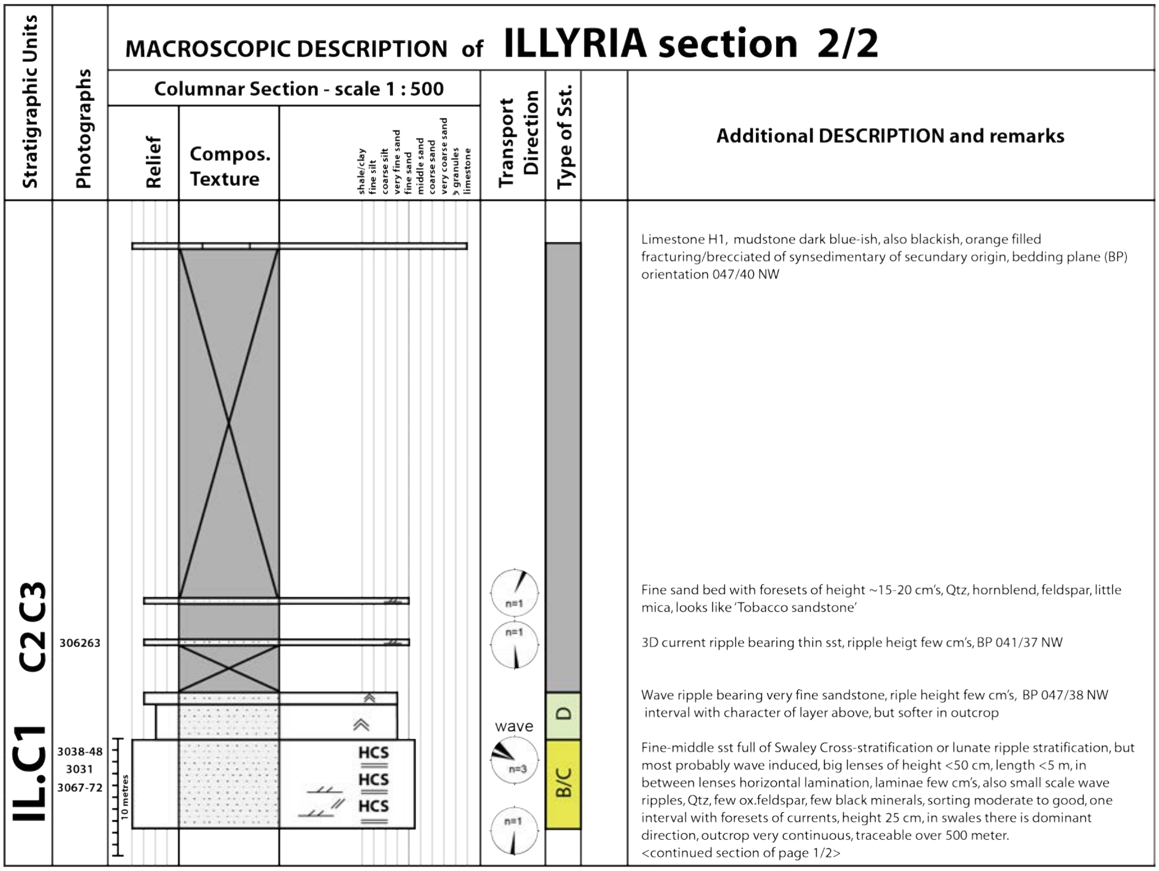
MACROSCOPIC DESCRIPTION of ILLYRIA section 2/2
Stratigraphic Units
Photographs
Columnar Section - scale 1 : 500
Relief
Compos. Texture
shale/clay
fine silt
coarse silt
very fine sand
fine sand
middle sand
coarse sand
very coarse sand
granules
limestone
Transport Direction
Type of Sst.
Additional DESCRIPTION and remarks
IL.C1
C2 C3
306263
3038-48
3031
3067-72
10 metres
HCS
HCS
HCS
wave
n=1
n=1
n=3
n=1
D
B/C
Limestone H1, mudstone dark blue-ish, also blackish, orange filled fracturing/brecciated of synsedimentary of secundary origin, bedding plane (BP) orientation 047/40 NW
Fine sand bed with foresets of height ~15-20 cm's, Qtz, hornblend, feldspar, little mica, looks like 'Tobacco sandstone'
3D current ripple bearing thin sst, ripple heigt few cm's, BP 041/37 NW
Wave ripple bearing very fine sandstone, riple height few cm's, BP 047/38 NW interval with character of layer above, but softer in outcrop
Fine-middle sst full of Swaley Cross-stratification or lunate ripple stratification, but most probably wave induced, big lenses of height <50 cm, length <5 m, in between lenses horizontal lamination, laminae few cm's, also small scale wave ripples, Qtz, few ox.feldspar, few black minerals, sorting moderate to good, one interval with foresets of currents, height 25 cm, in swales there is dominant direction, outcrop very continuous, traceable over 500 meter.
<continued section of page 1/2>

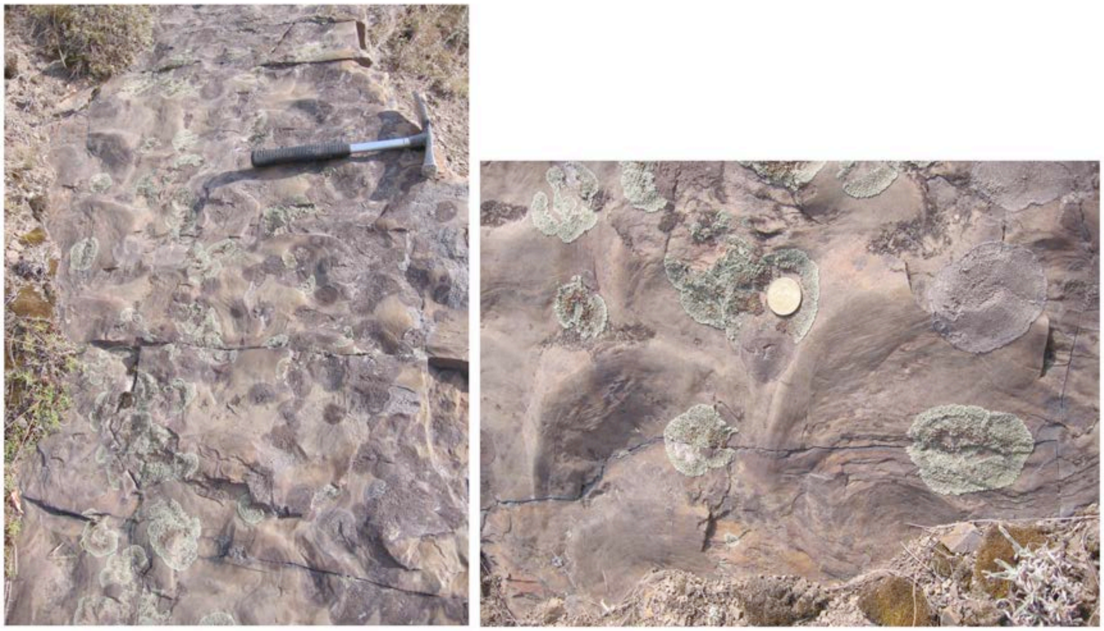

Photo 3062 (left) and 3063 **3D current ripples weathered out on a dip slope**

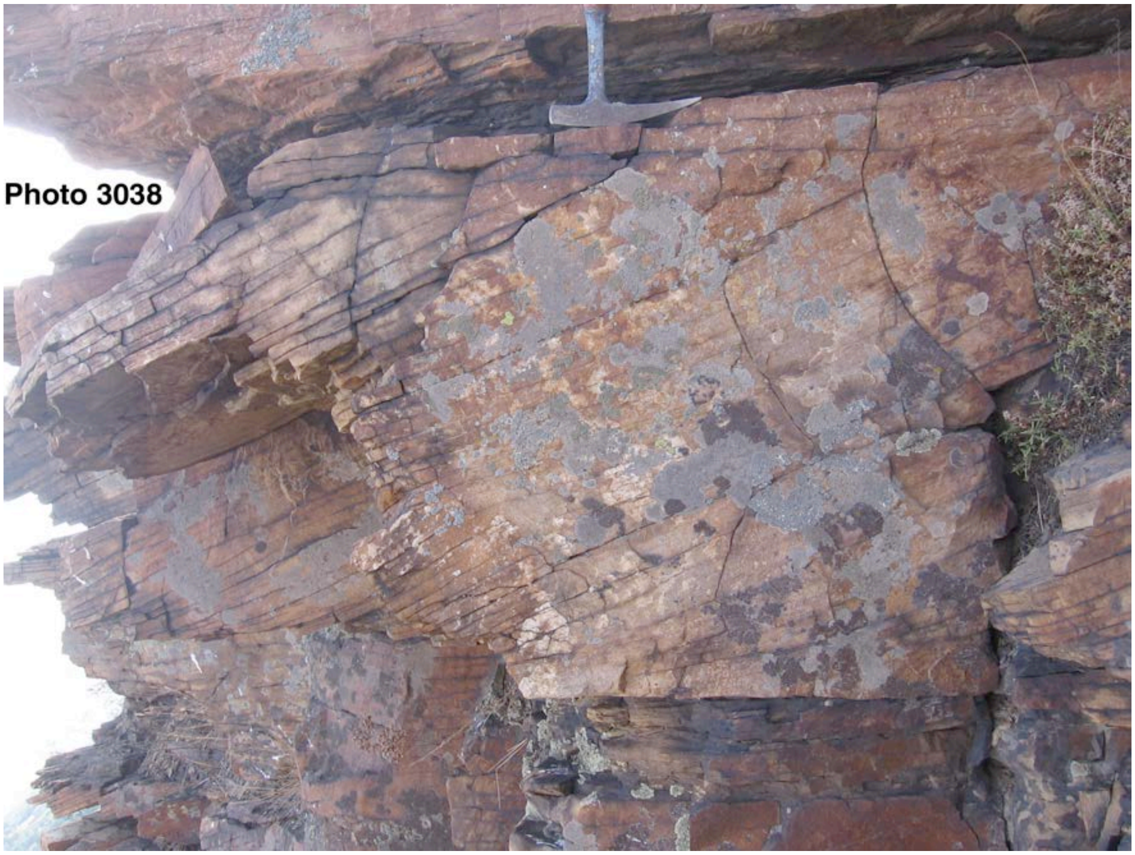

Photo 3038

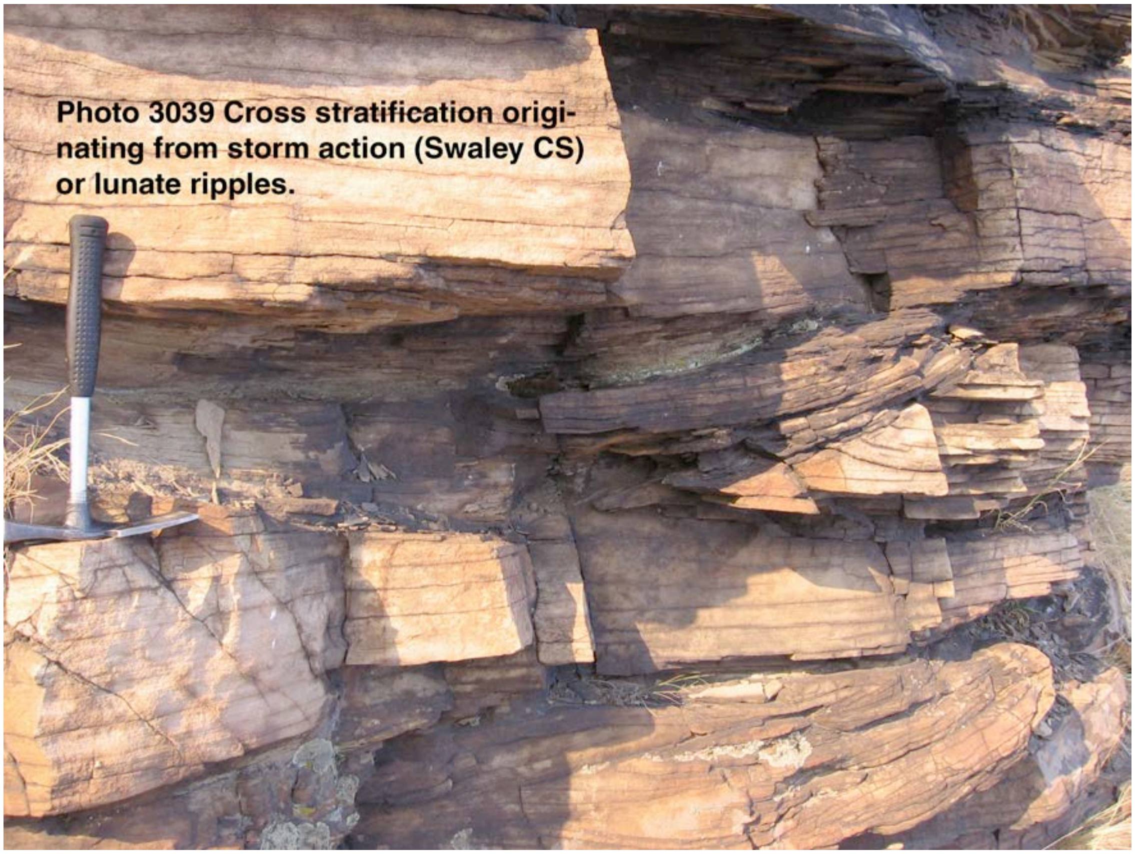

Photo 3039 Cross stratification originating from storm action (Swaley CS) or lunate ripples.

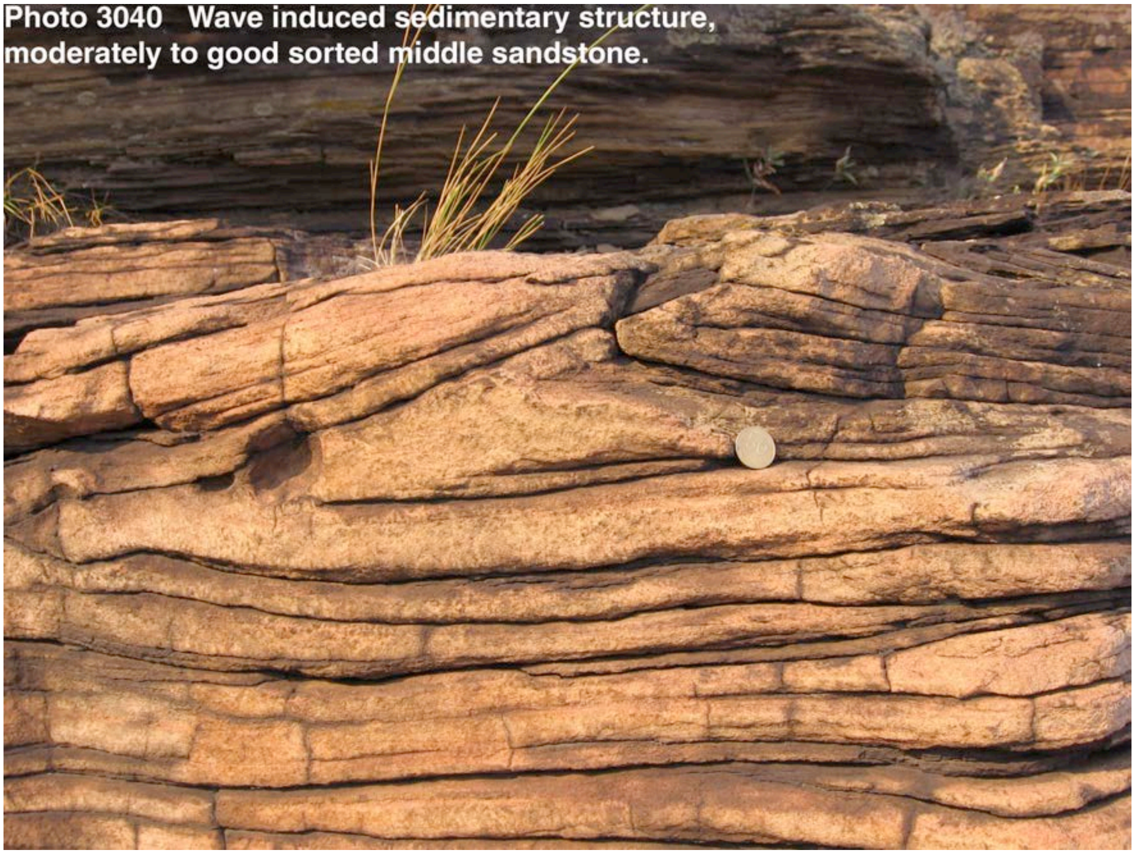

Photo 3040 Wave induced sedimentary structure, moderately to good sorted middle sandstone.

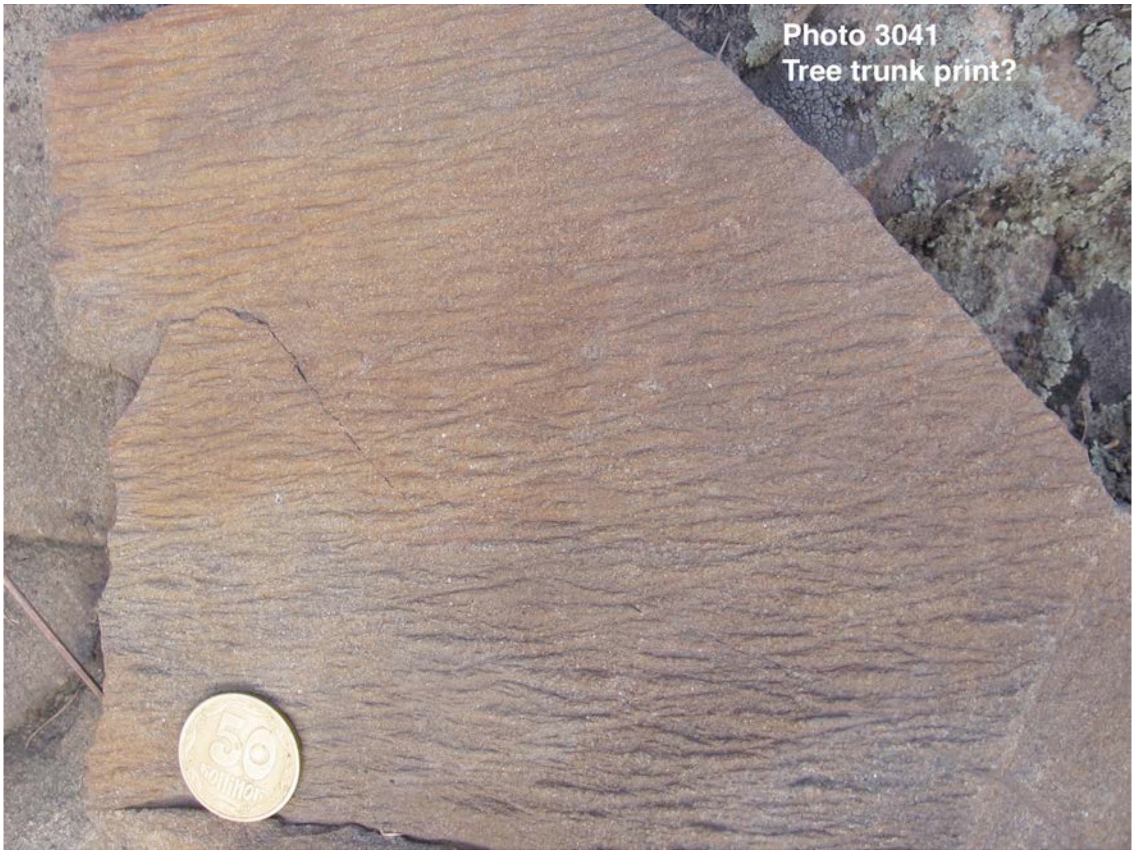
Photo 3041
Tree trunk print?

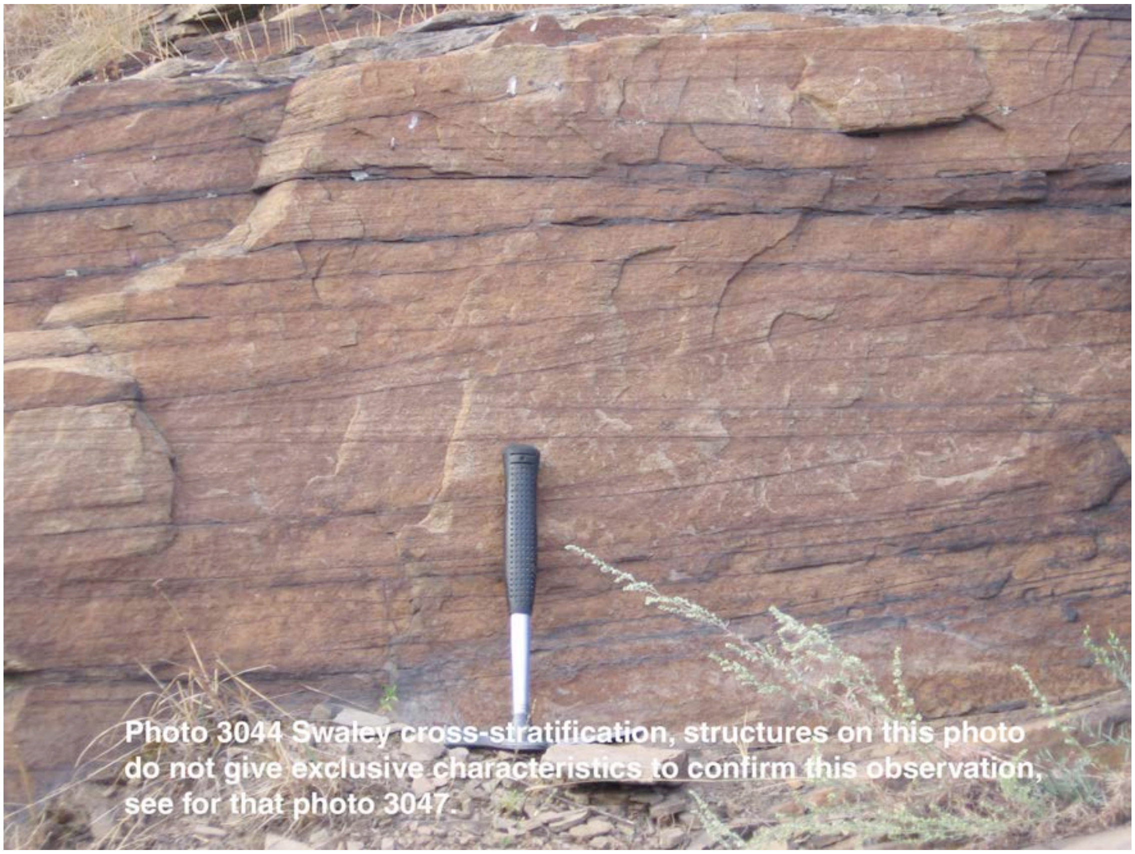
Photo 3044 Swaley cross-stratification, structures on this photo do not give exclusive characteristics to confirm this observation, see for that photo 3047.

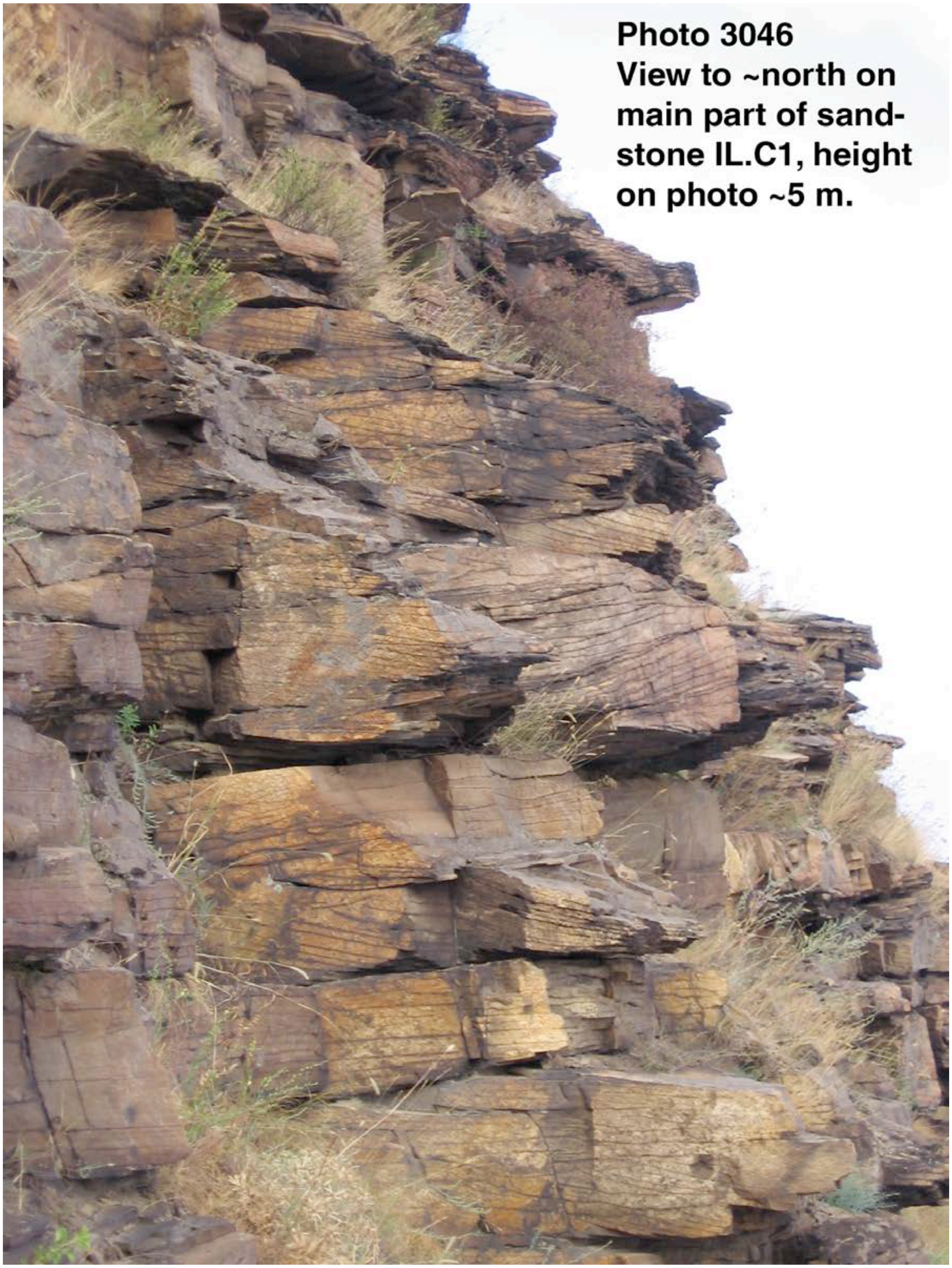

Photo 3046
View to ~north on main part of sandstone IL.C1, height on photo ~5 m.

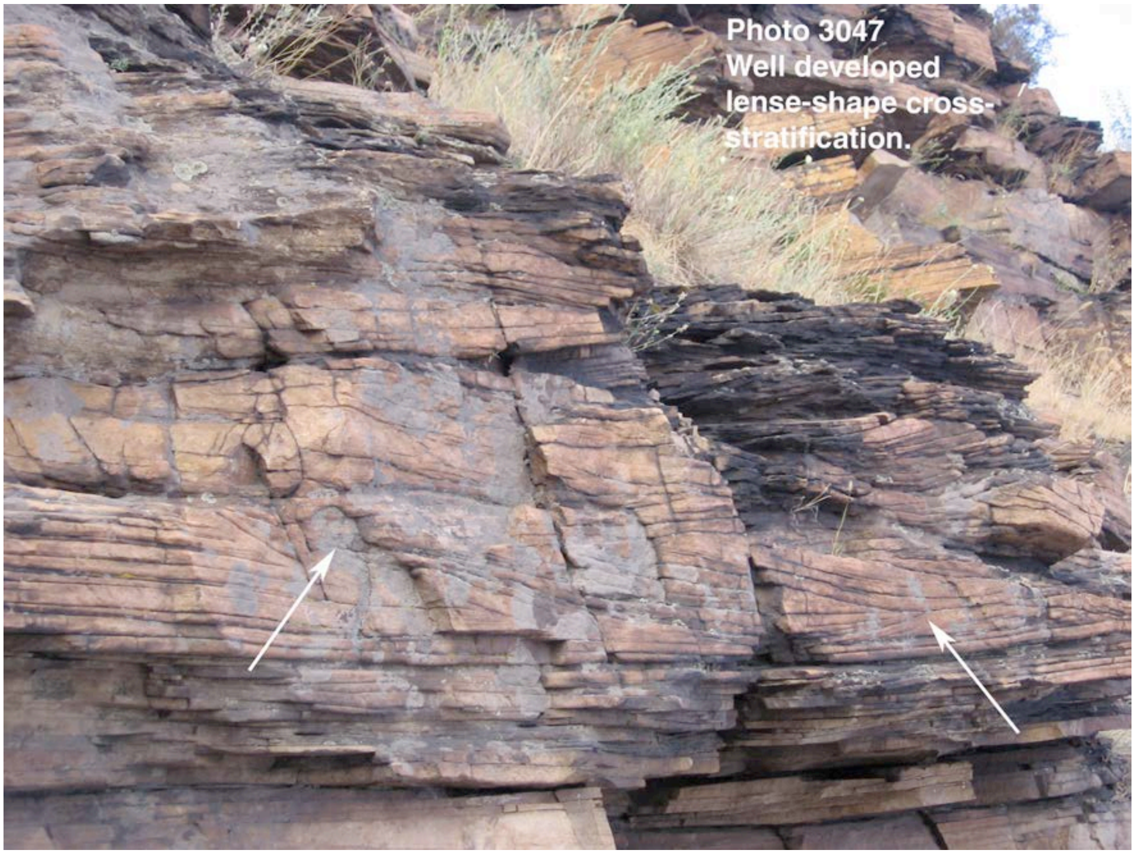

Photo 3047
Well developed
lense-shape cross-
stratification.

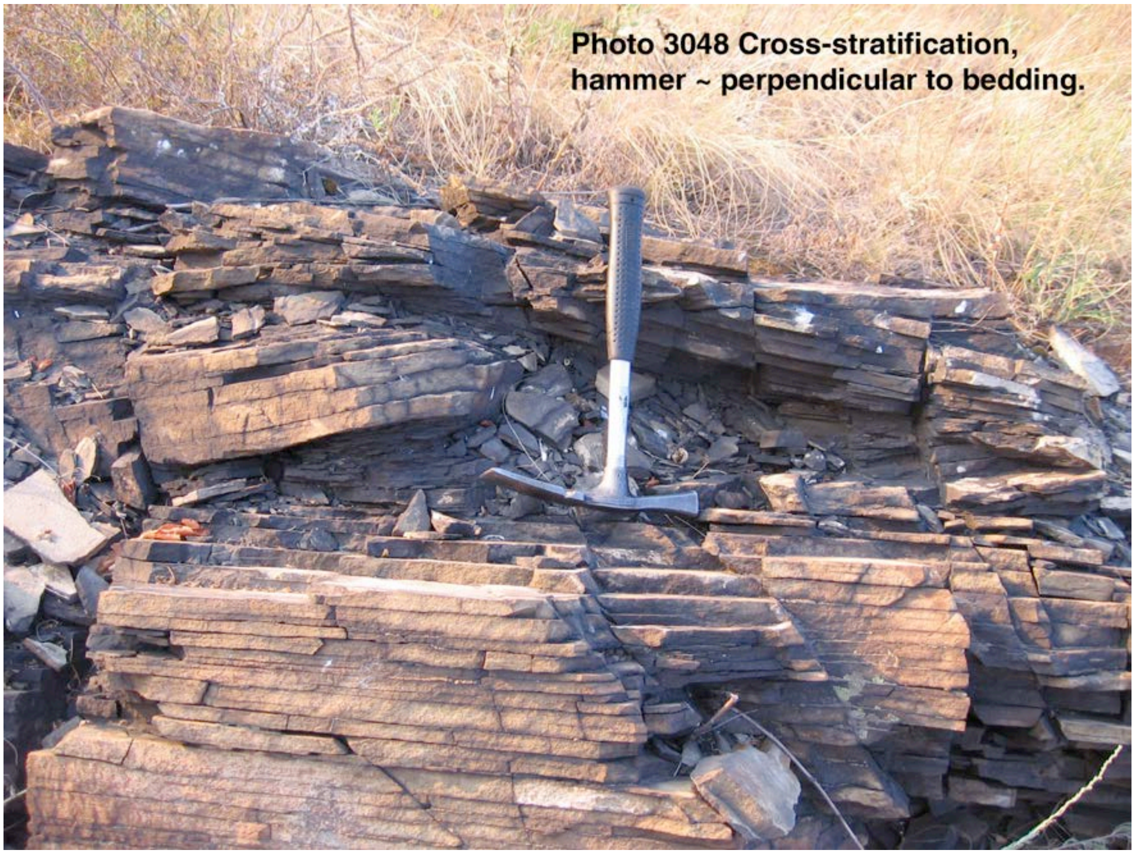

Photo 3048 Cross-stratification,
hammer ~ perpendicular to bedding.

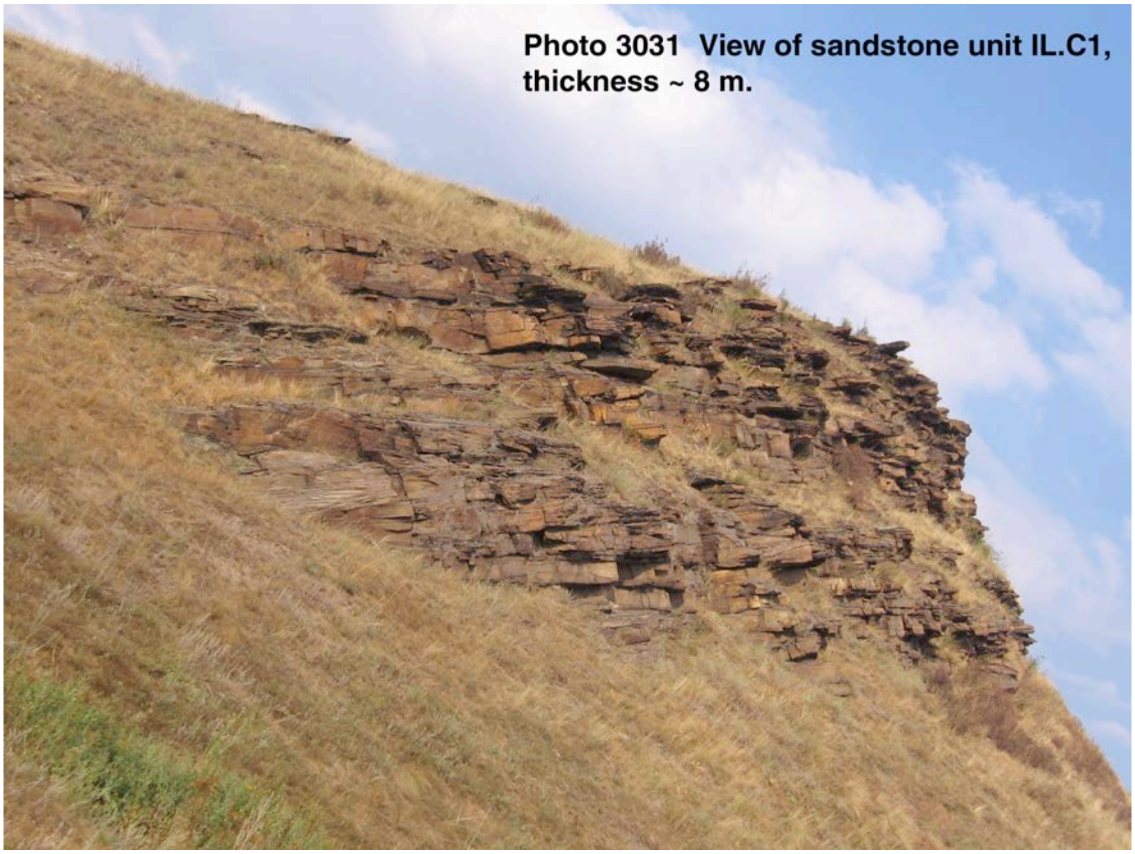

Photo 3031 View of sandstone unit IL.C1, thickness ~ 8 m.

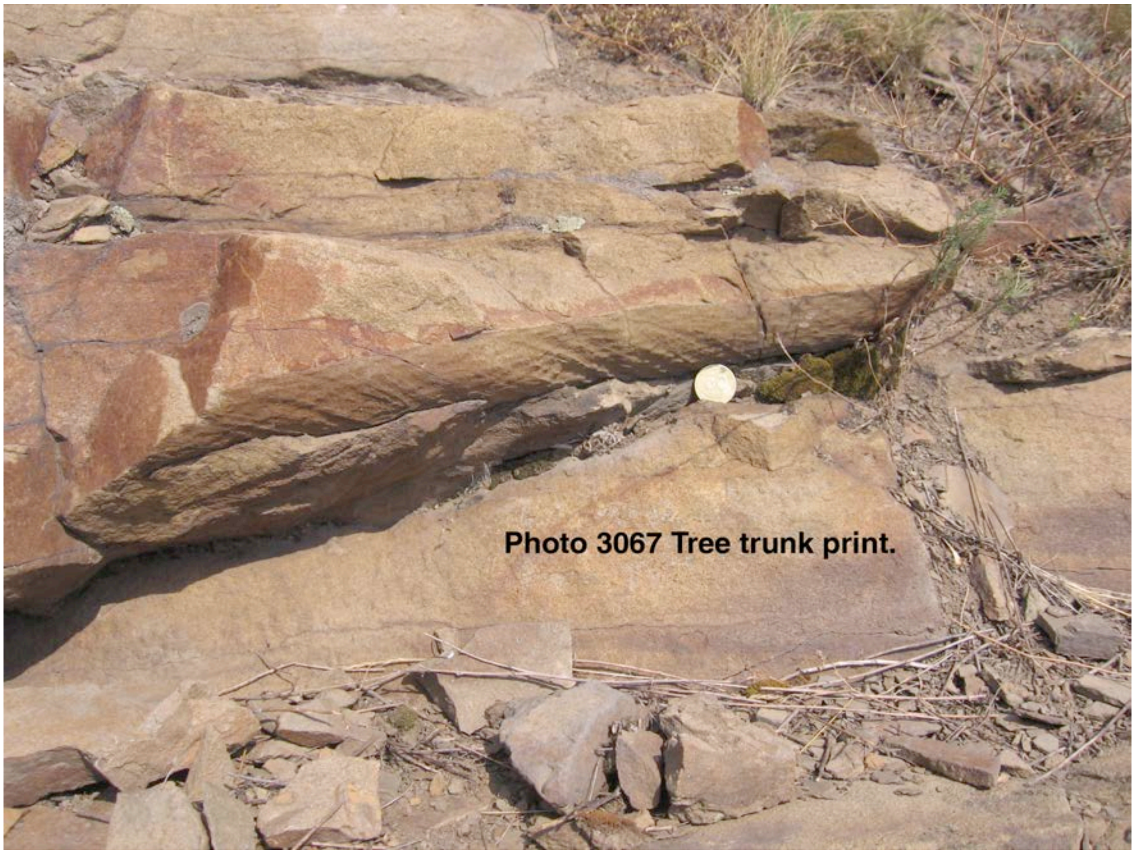

Photo 3067 Tree trunk print.

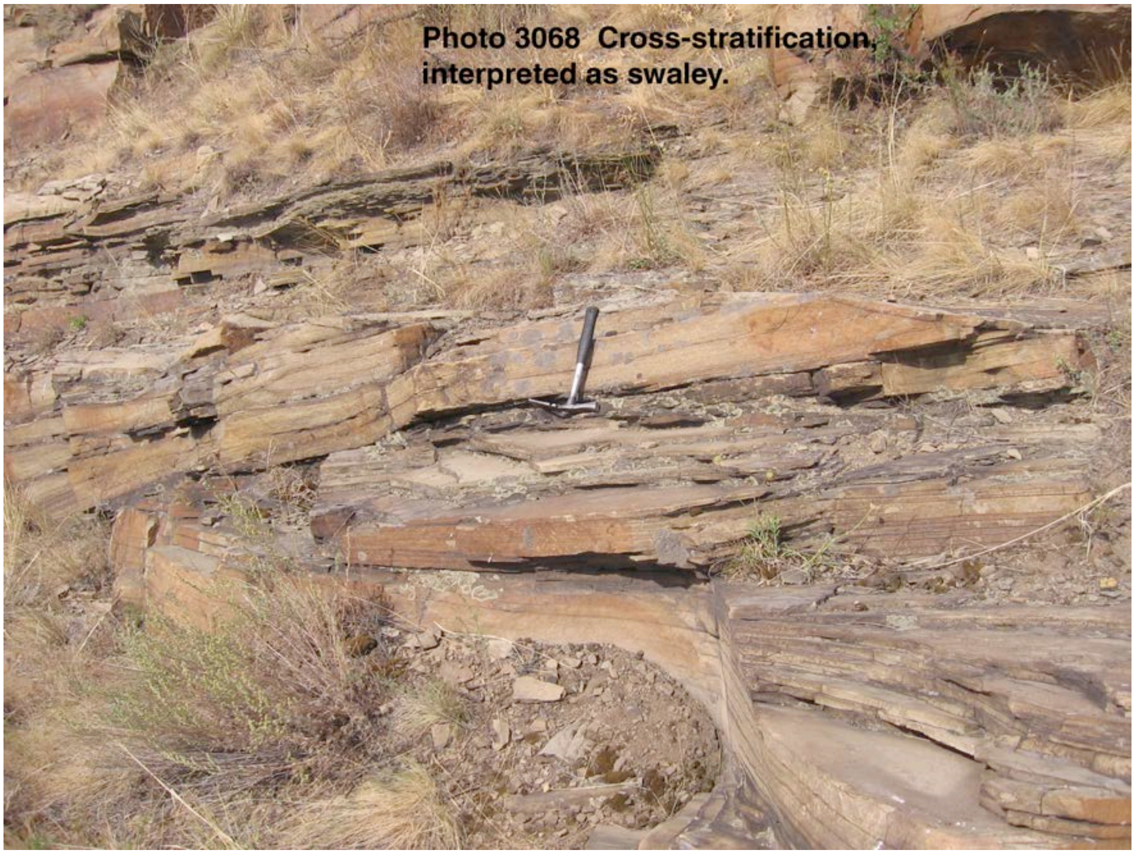

Photo 3068 Cross-stratification, interpreted as swaley.

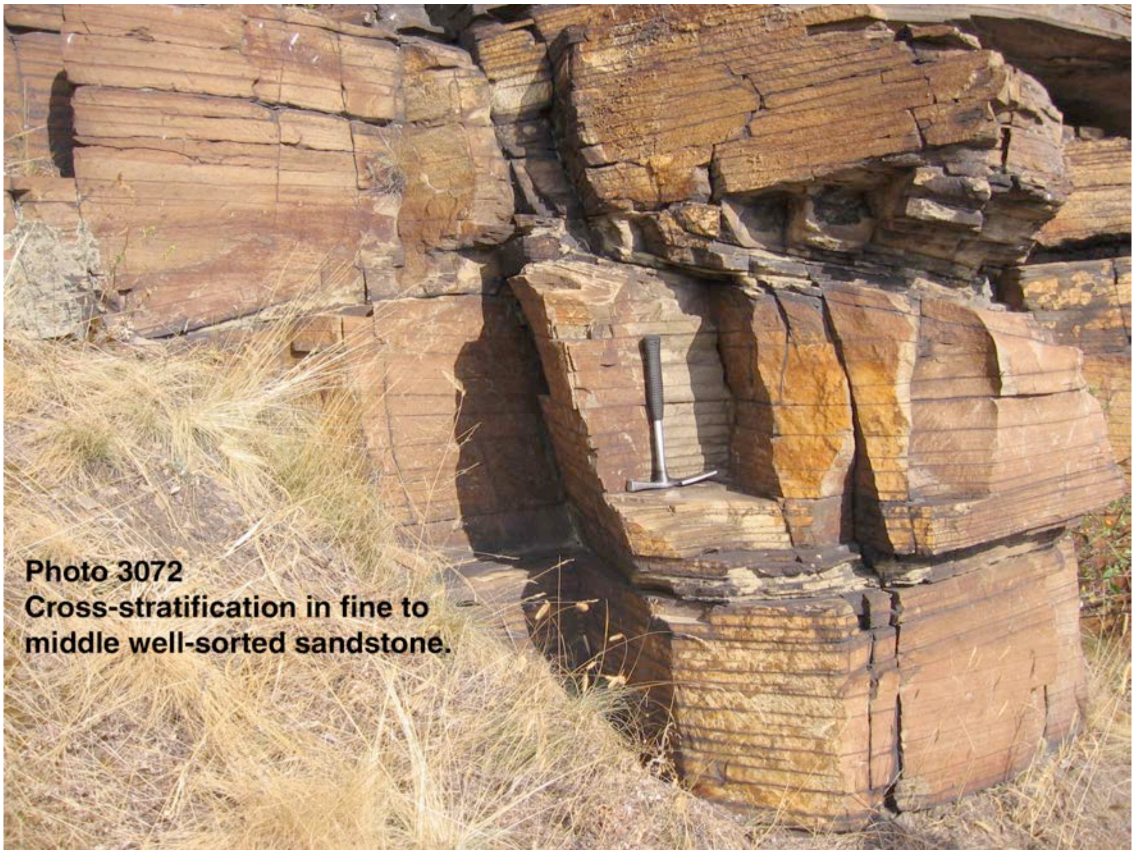

Photo 3072
Cross-stratification in fine to middle well-sorted sandstone.

Section: ***Malo-Orlovka***

Location: 37U 0453774 UTM 5336206, central area

Situation: The Malo-Orlovka section is situated in a river/lake outcrop close to the road in between Малоорловка (Malo-Orlovka) and Новоорловка (Novoorlovka).

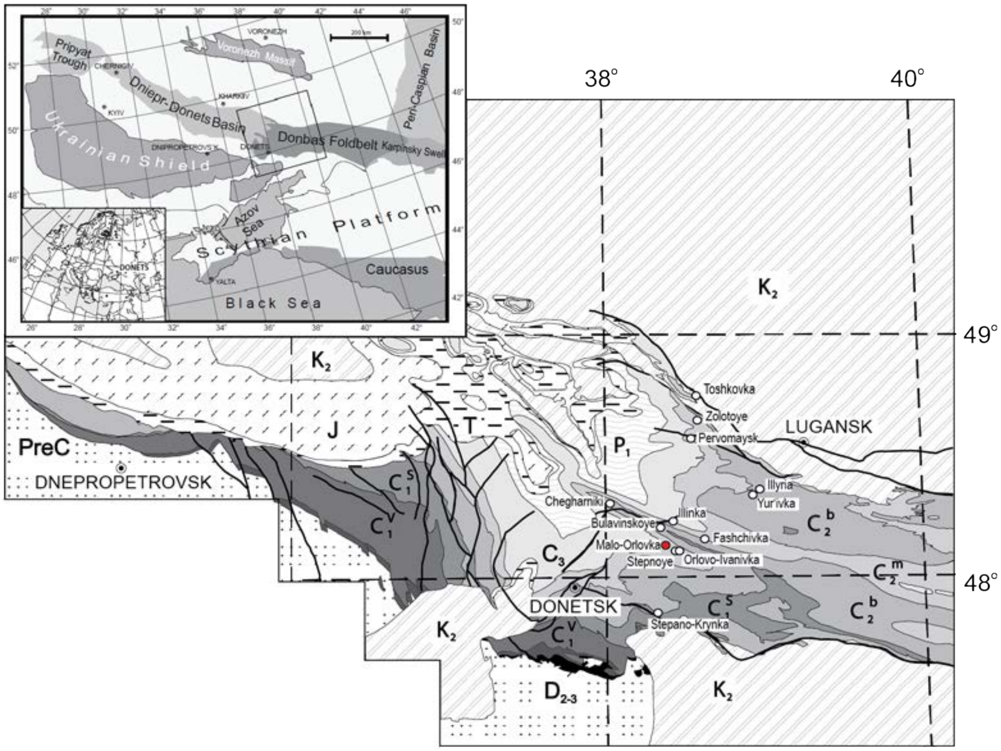

Age: **lower Moscovian, C_2^6,** just below limestone M_1

% Sand: **12 %**

Thickness: **233 m**

Sedimentology:

The Malo-Orlovka section is characterised by a very high amount of shales and silt versus sandstones. It is also the longest studied section and the outcrop even permits to study more stratigraphy in future. Only three sandstone units are present that have a grainsize larger than very fine sand. These all exhibit characteristics of Group C. Other present sandstone are very fine, intercalate with silts and belong to Group D.

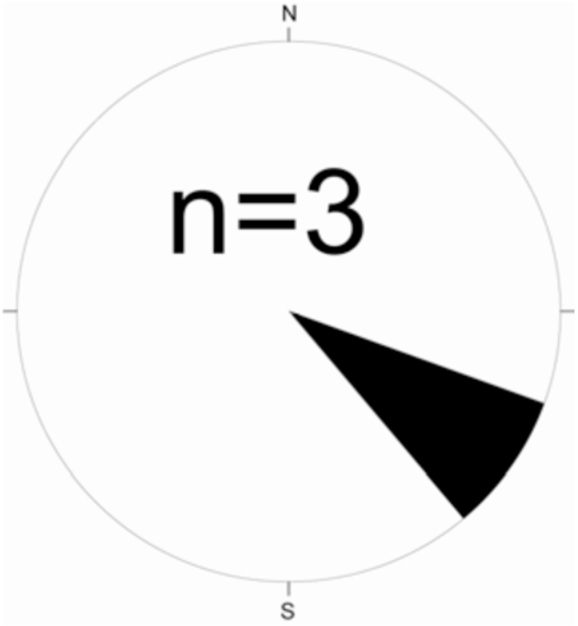

Figure XDS. Rose diagram of all measured paleocurrent directions in the Dried-Source section. Petals in groups of 10°, largest petal contains 1 measurement and 33% of the data.

Environmental interpretation:

The sediments of the Malo-Orlovka section all show characteristics of middle to lower shoreface and shelf environments (Group C and D and shales). The section clearly shows the intercalation of lower shoreface sandstones (Group D) in between huge intervals of shales and silts, and therefore strengthens the relatively deep water interpretation of these fine sediments. The large intervals without clear outcrops are not at all expected to contain considerable sandstones because these would have been weathered out. Although these intervals might contain some extra sandstones of Group D or more well possible some extra silts.

MACROSCOPIC DESCRIPTION of MALO-ORLOVKA section 1/2

Stratigraphic Units	Photographs	Columnar Section - scale 1 : 500 (Relief / Compos. Texture / grain size)	Transport Direction	Type of Sst.	Additional DESCRIPTION and remarks

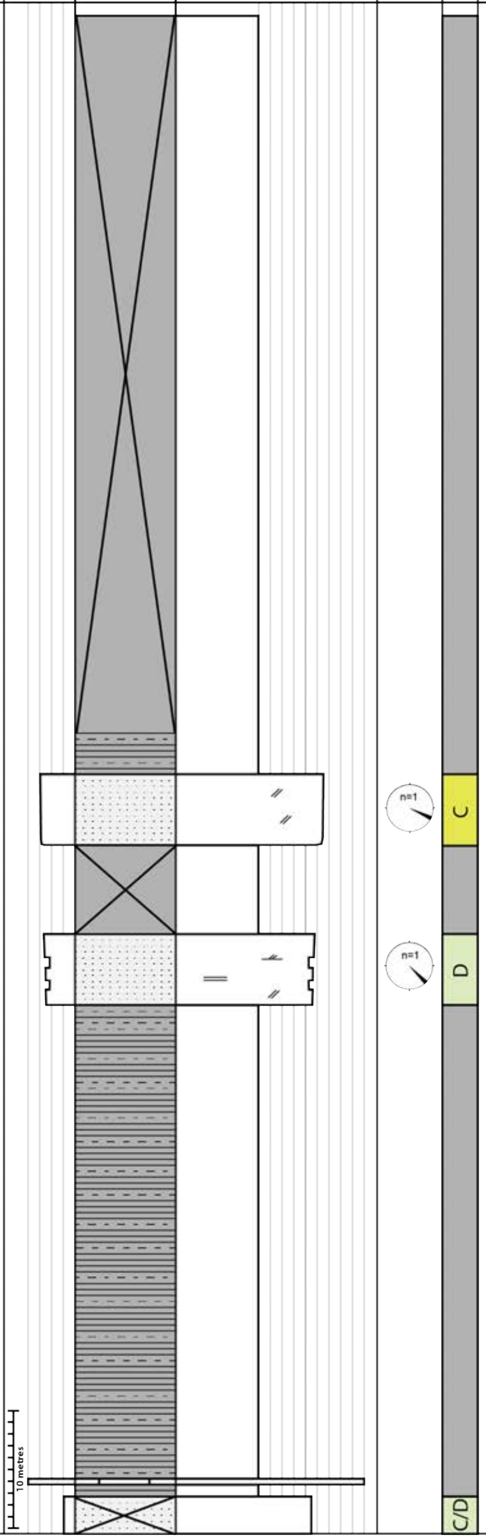

Stratigraphic Units: DS.A DS.B

Photographs: 2974

Type of Sst.: C; D; C/D

Continued on page 2/2

non-outcrop interval with one thin coarse and one thin finer sandstone bed, no indication for other sandstone layers have been found besides some sandstone boulders for which no layer could be found.

Bedded (<50 cm) moderately to well sorted light coloured middle to coarse grained sandstone, current induced large scale cross-beds (~50 cm), 70% quartz, few oxidised minerals (feldspar), few black minerals, spherical angular grains, BP 102/45 S

Fine to middle sandstone unit with some silty layers, foresets up to 20 cm, also cross-beds at 5-8 cm, current ripples, 40% qtz, 30% ox.minerals, 30% black minerals, also beds with lower plane bed lamination of thickness 1 cm, in fine to middle sand

interval of shales and silts, interval crosses road, at road sample DSp-3 is taken.

limestone, not very prominent

End of Section No direct continuous outcrop found. Might be there.

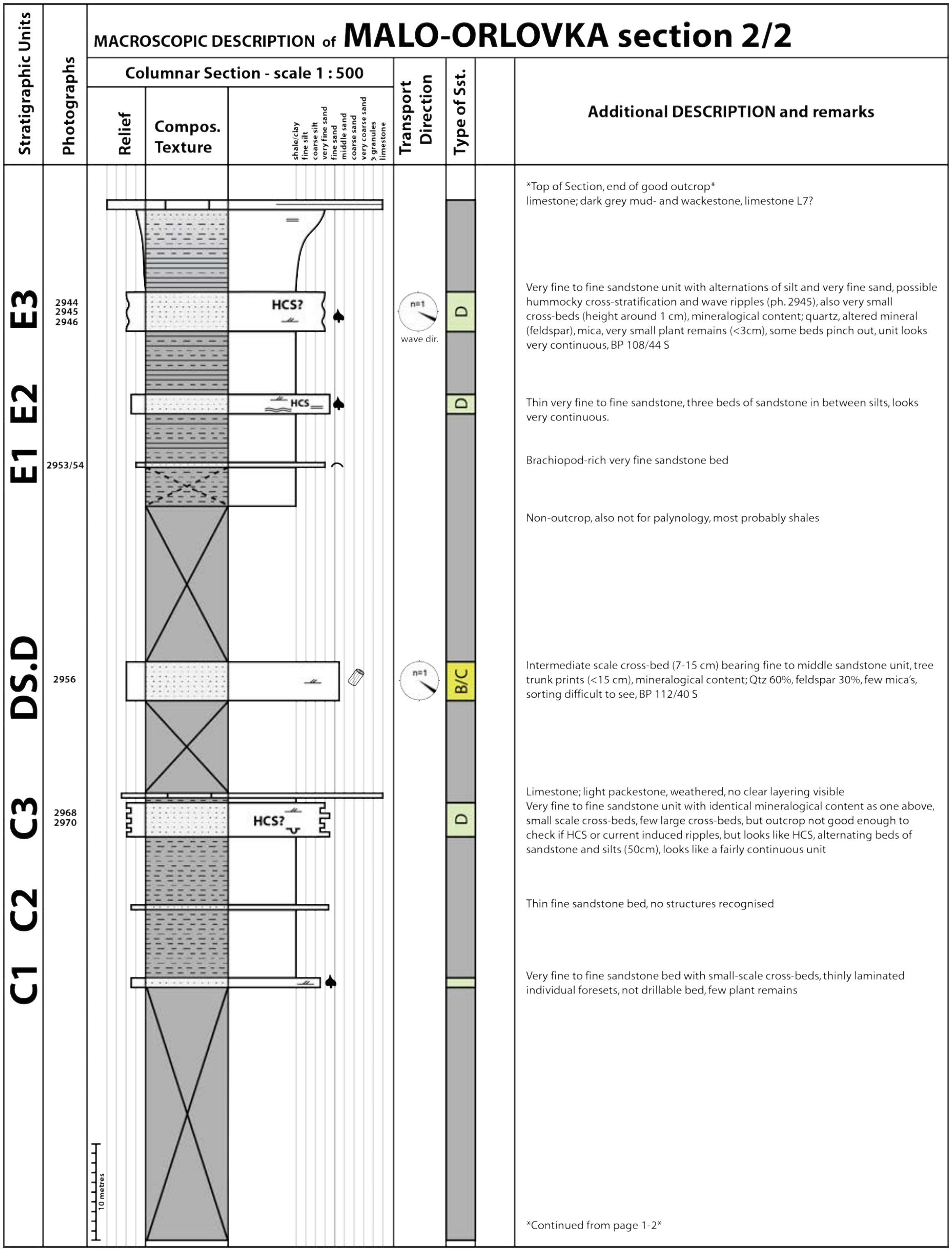
MACROSCOPIC DESCRIPTION of MALO-ORLOVKA section 2/2
Stratigraphic Units
Photographs
Columnar Section - scale 1 : 500
Relief
Compos. Texture
shale/clay
fine silt
coarse silt
very fine sand
fine sand
middle sand
coarse sand
very coarse sand
granules
limestone
Transport Direction
Type of Sst.
Additional DESCRIPTION and remarks
E3
E2
E1
DS.D
C3
C2
C1
2944
2945
2946
2953/54
2956
2968
2970
HCS?
HCS
HCS?
n=1
wave dir.
n=1
D
D
B/C
D
10 metres
Top of Section, end of good outcrop
limestone; dark grey mud- and wackestone, limestone L7?
Very fine to fine sandstone unit with alternations of silt and very fine sand, possible hummocky cross-stratification and wave ripples (ph. 2945), also very small cross-beds (height around 1 cm), mineralogical content; quartz, altered mineral (feldspar), mica, very small plant remains (<3cm), some beds pinch out, unit looks very continuous, BP 108/44 S
Thin very fine to fine sandstone, three beds of sandstone in between silts, looks very continuous.
Brachiopod-rich very fine sandstone bed
Non-outcrop, also not for palynology, most probably shales
Intermediate scale cross-bed (7-15 cm) bearing fine to middle sandstone unit, tree trunk prints (<15 cm), mineralogical content; Qtz 60%, feldspar 30%, few mica's, sorting difficult to see, BP 112/40 S
Limestone; light packestone, weathered, no clear layering visible
Very fine to fine sandstone unit with identical mineralogical content as one above, small scale cross-beds, few large cross-beds, but outcrop not good enough to check if HCS or current induced ripples, but looks like HCS, alternating beds of sandstone and silts (50cm), looks like a fairly continuous unit
Thin fine sandstone bed, no structures recognised
Very fine to fine sandstone bed with small-scale cross-beds, thinly laminated individual foresets, not drillable bed, few plant remains
Continued from page 1-2

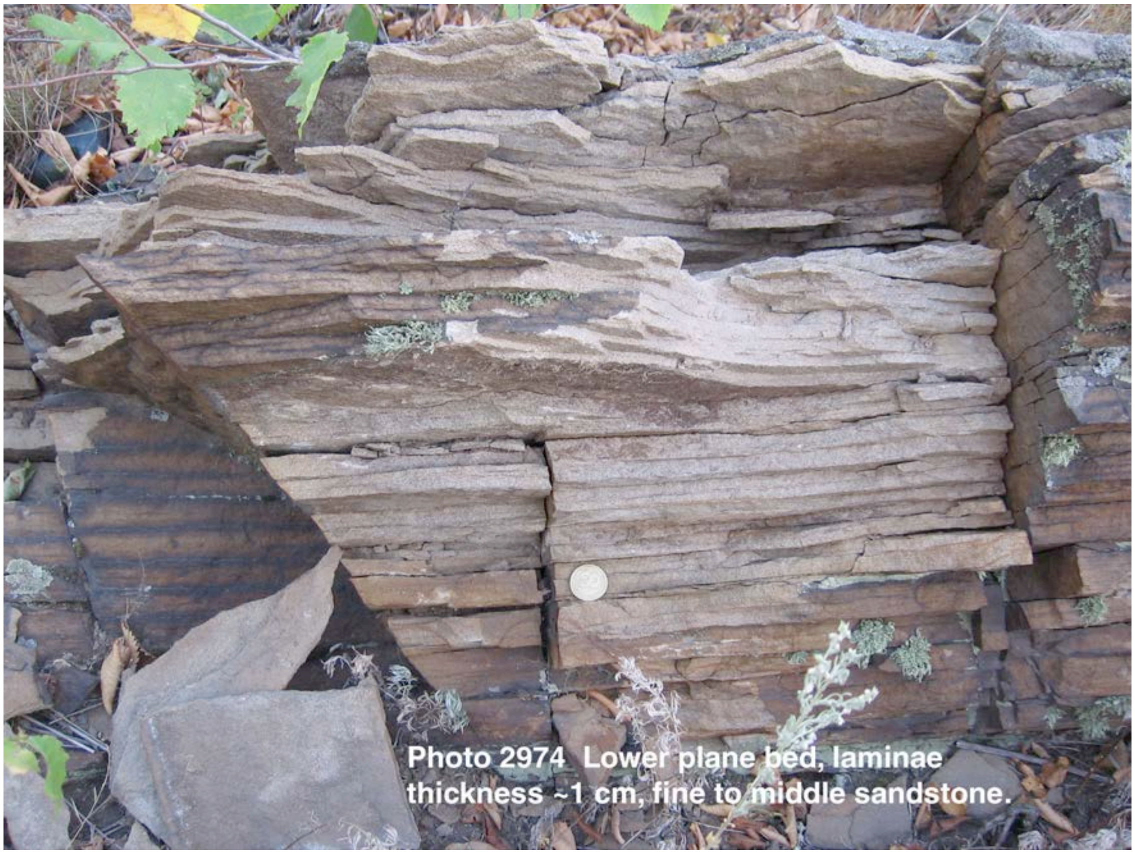
Photo 2974 Lower plane bed, laminae thickness ~1 cm, fine to middle sandstone.

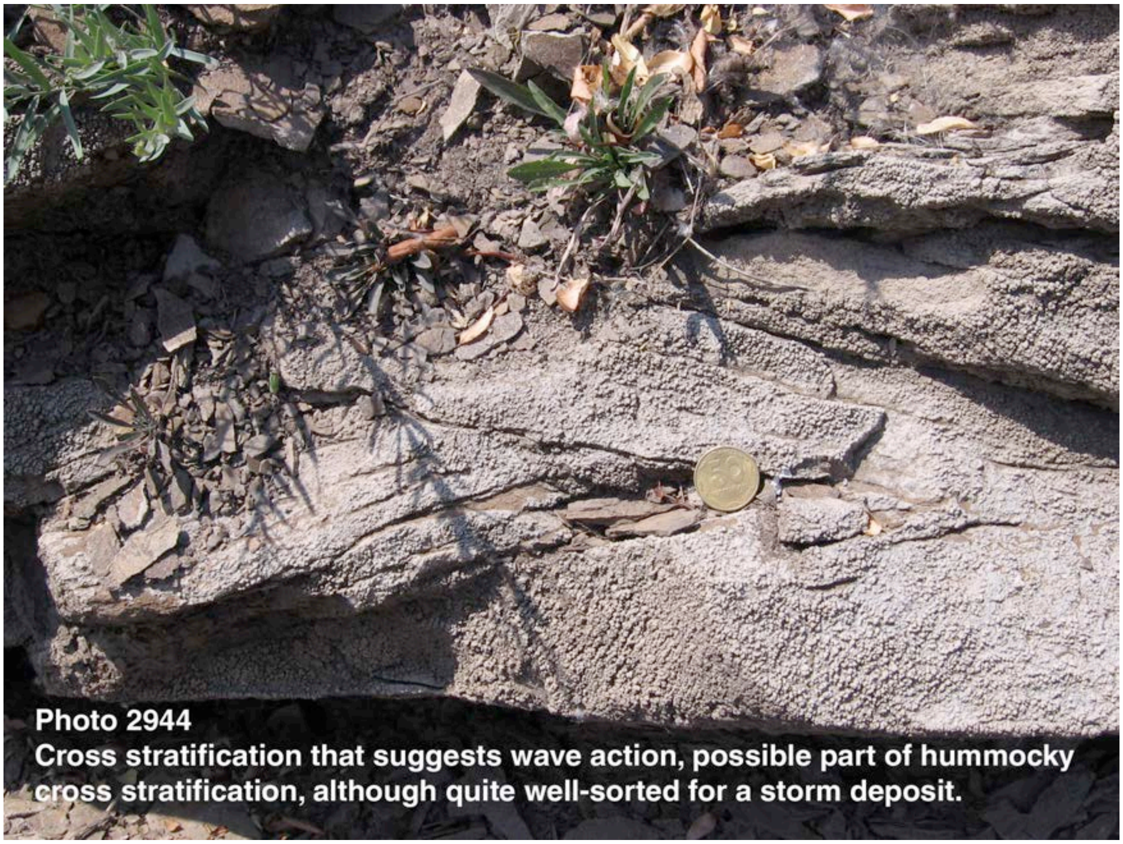
Photo 2944
Cross stratification that suggests wave action, possible part of hummocky cross stratification, although quite well-sorted for a storm deposit.

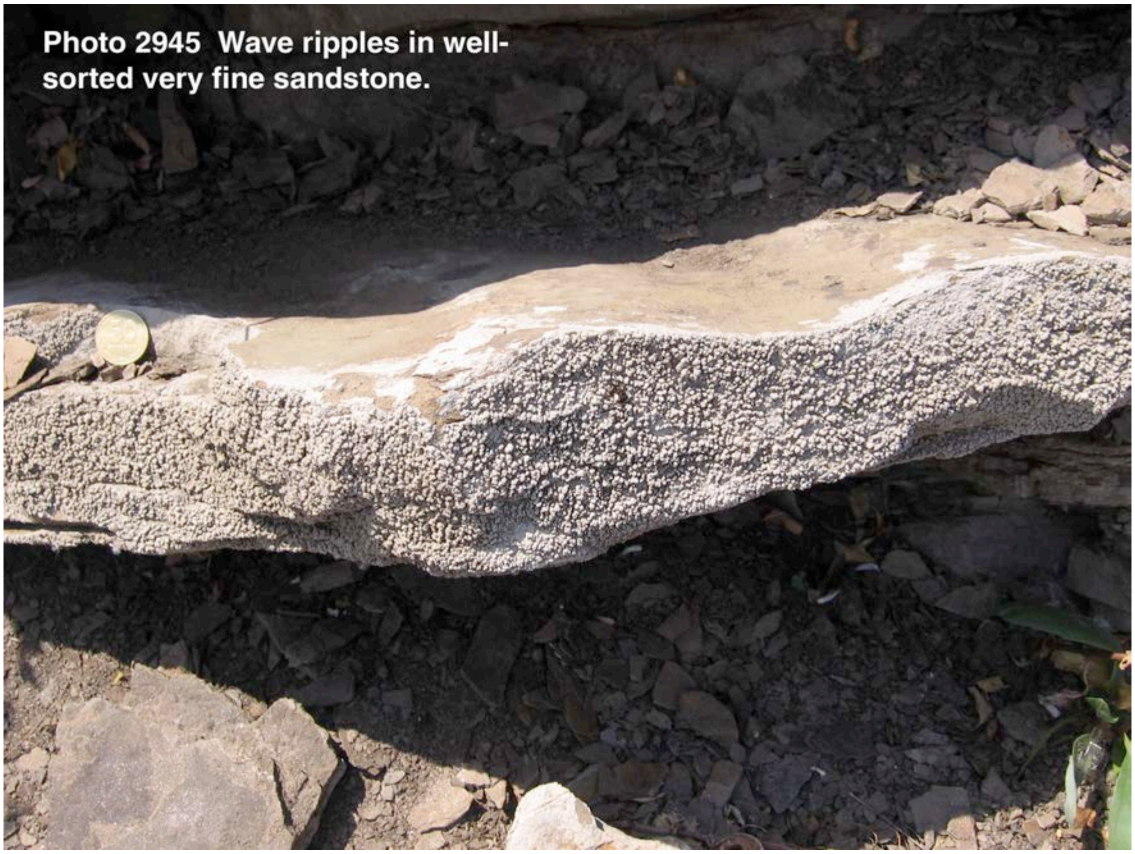
Photo 2945 Wave ripples in well-sorted very fine sandstone.

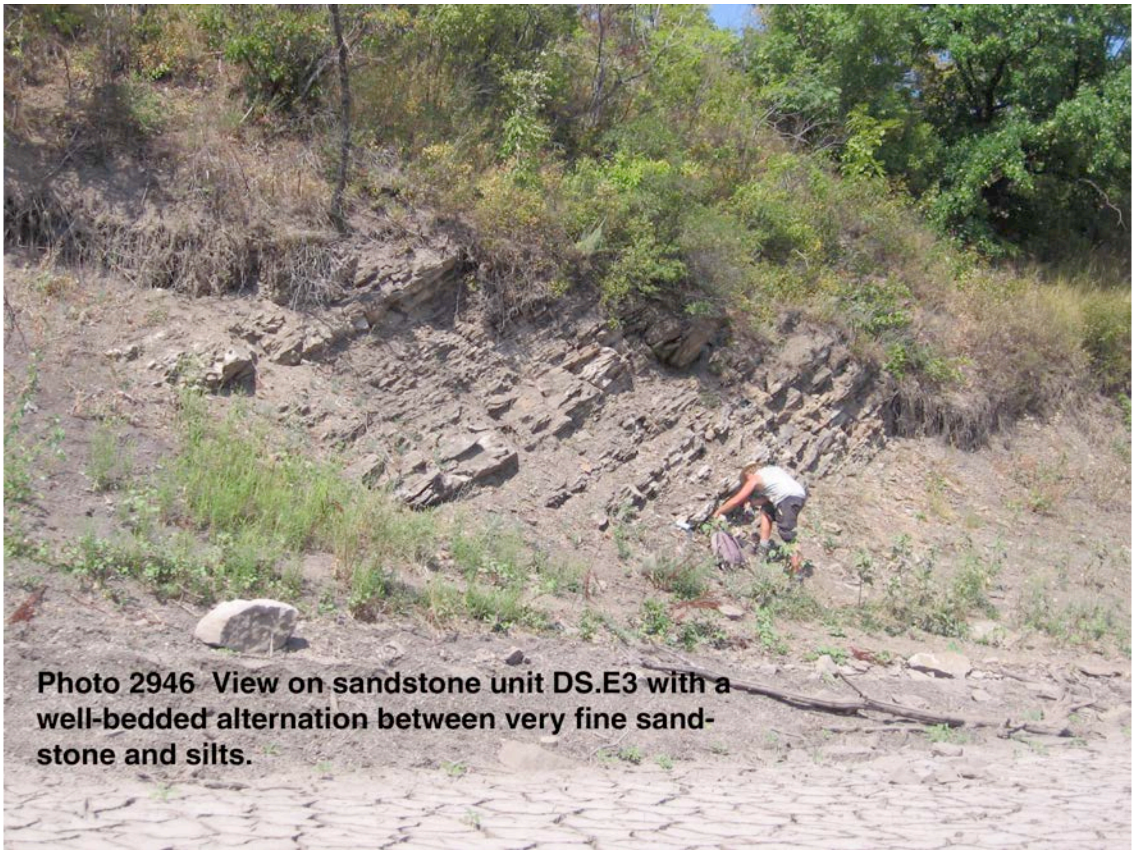
Photo 2946 View on sandstone unit DS.E3 with a well-bedded alternation between very fine sandstone and silts.

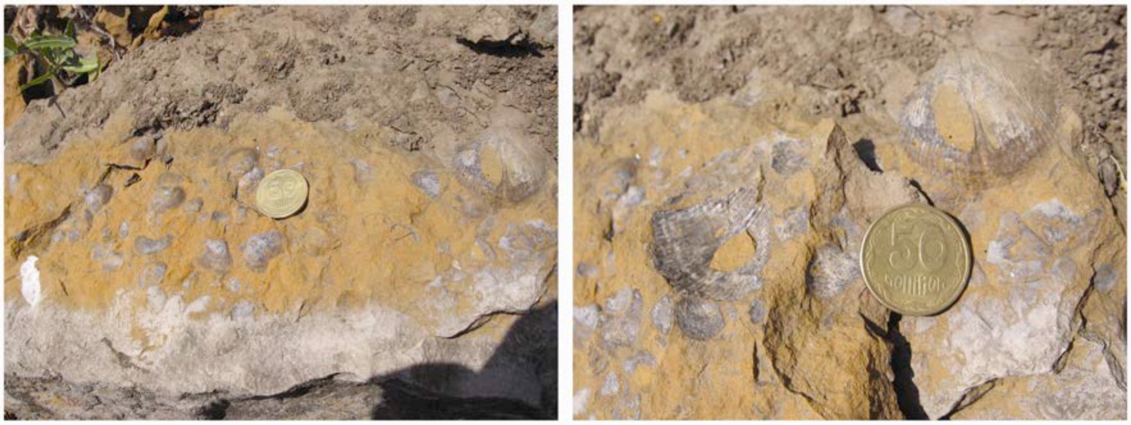

Photos 2953 (right) and 2954   Brachiopod-rich very fine sandstone bed.

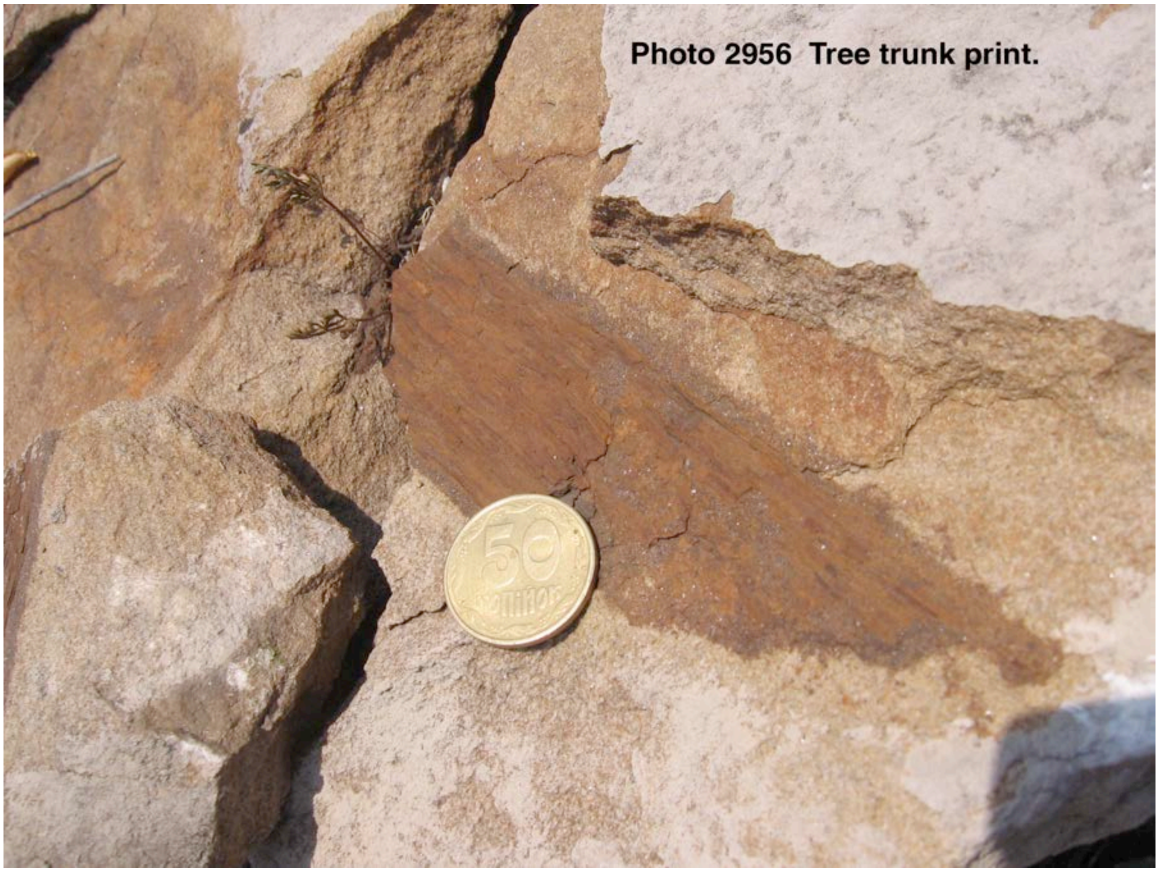

Photo 2956 Tree trunk print.

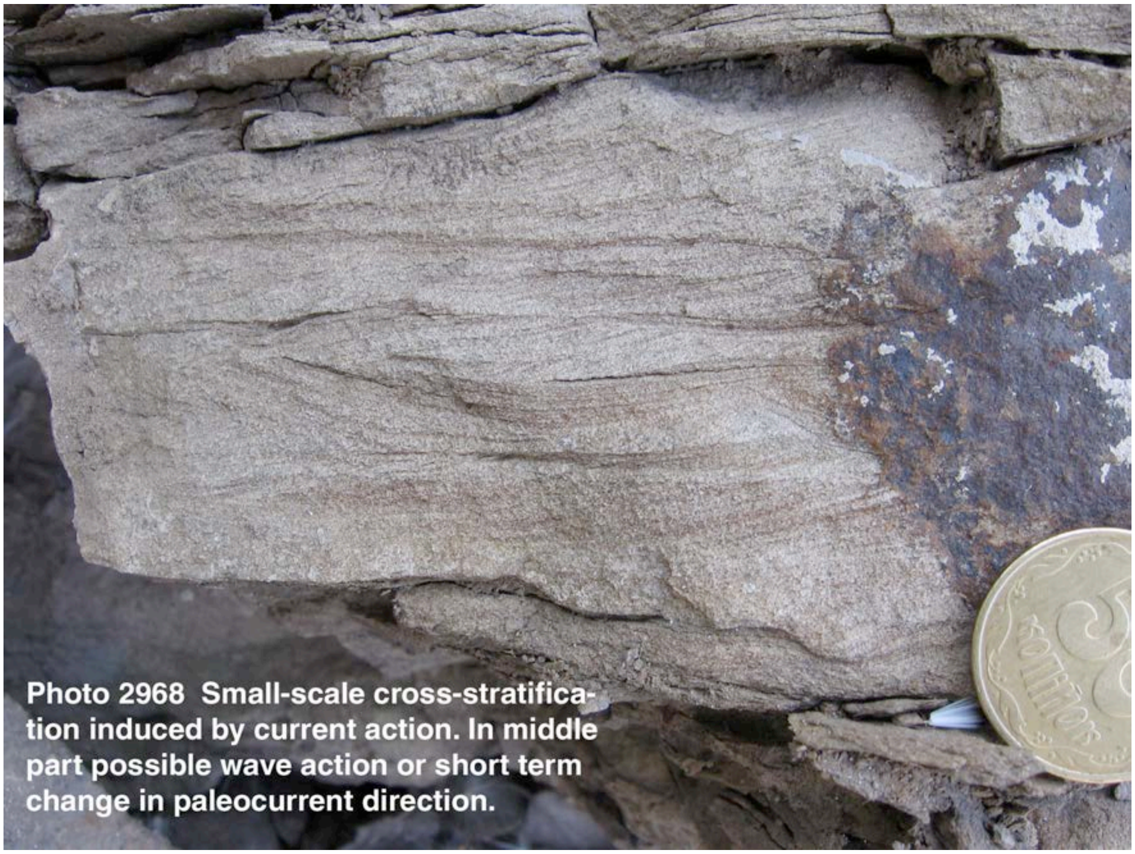

Photo 2968 Small-scale cross-stratification induced by current action. In middle part possible wave action or short term change in paleocurrent direction.

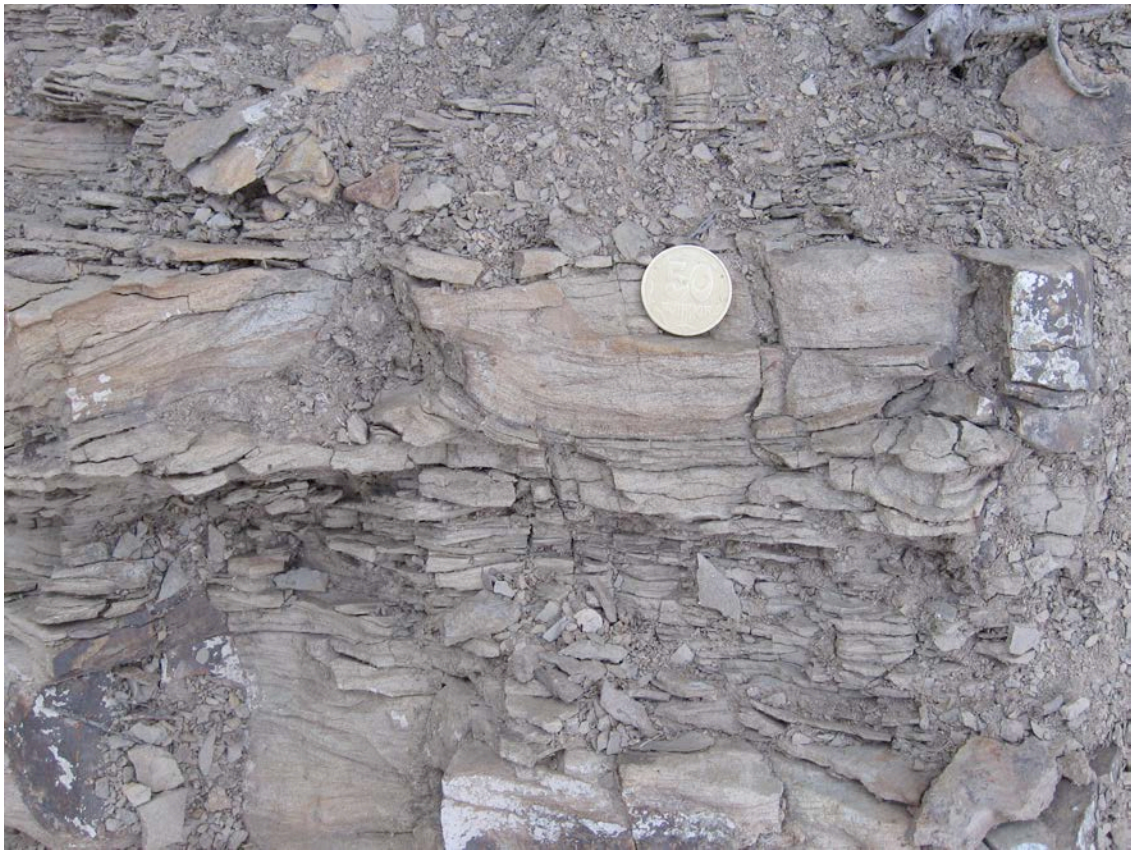

Section: ***Orlovo-Ivanivka***

Location: 37U 0459526 UTM 5334117, central area

Situation: Orlovo-Ivanivka section is named after the nearby called Орлово-Ивановка (Orlovo-Ivanovka). The section is situated on the west flank of the biggest lake just south of the village and can be reached by the unpaved road that departs just before the first building of the village from the paved road into the village from the west. The section is situated along the lake shoreline.

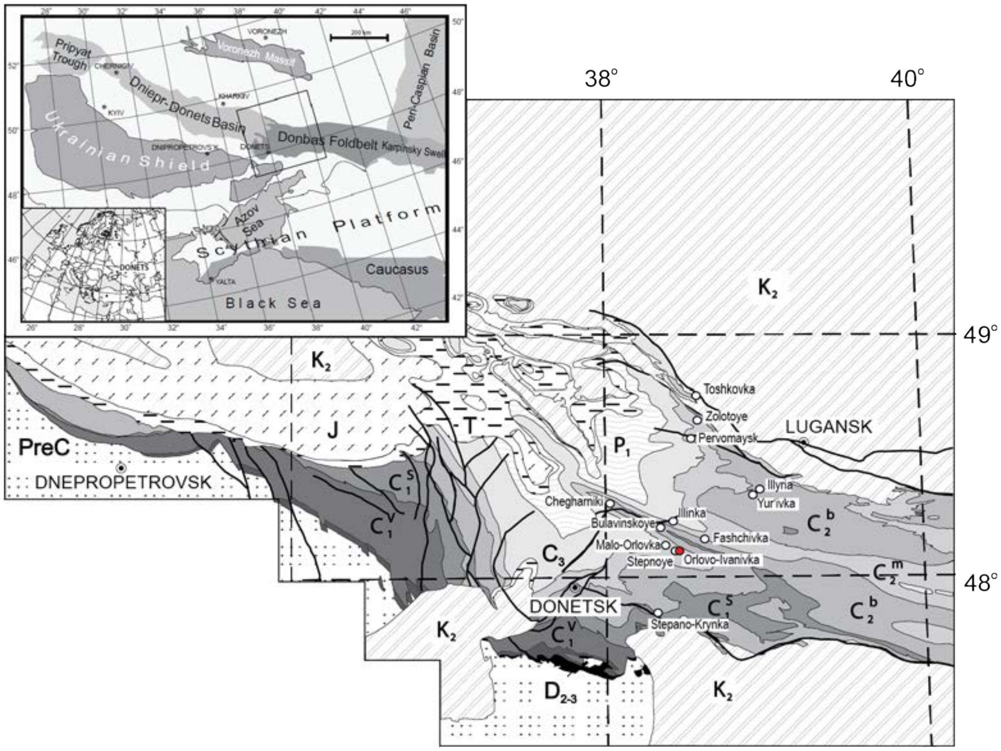

Age: **upper Bashkirian, C_2^4** and **C_2^5**, limestone K_1 is situated in the section.

% Sand: **28 %**

Thickness: **190 m**

Sedimentology:

The upper part of the stratigraphy of the Orlovo-Ivanivka section is characterised differently than the larger lower part. The lower part is characterised by long shale and silt intervals with well sorted very fine to fine sandstone of Group D and some features of Group C. In the upper part sandstone OI.C1 and OI.C2 show characteristics of Group B and C, while topmost unit (OI.D) has features from Group A. In the village Orlovo-Ivanovka sandstone have been found that belong older stratigraphy than the studied section, while in between not much outcrop has been found.

Environmental interpretation:

The Orlovo-Ivanivka section shows for the larger lower part mainly shelf environments and some lower to middle shoreface environments during sandstone deposition. A gradual shoaling is revealed by the sandstones in the upper part of the section. The section uppermost sandstone shows fluvial characteristics of Group A. The long probably shaley interval below the section until the sandstones in the village itself might indicate deep environments for a long time, such that no big sandstone were deposited, which is seen as a 3rd order sea level cycle.

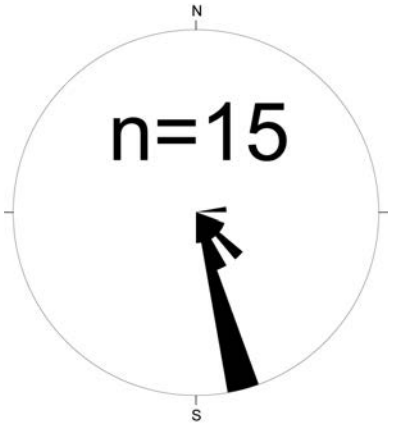

Figure XOI. Rose diagram of the all measured paleocurrent directions in the Orlovo-Ivanivka section. Petals in groups of 10°, largest petal represent 6 measurements and 40% of the data.

MACROSCOPIC DESCRIPTION of Orlovo-Ivanivka 1/2

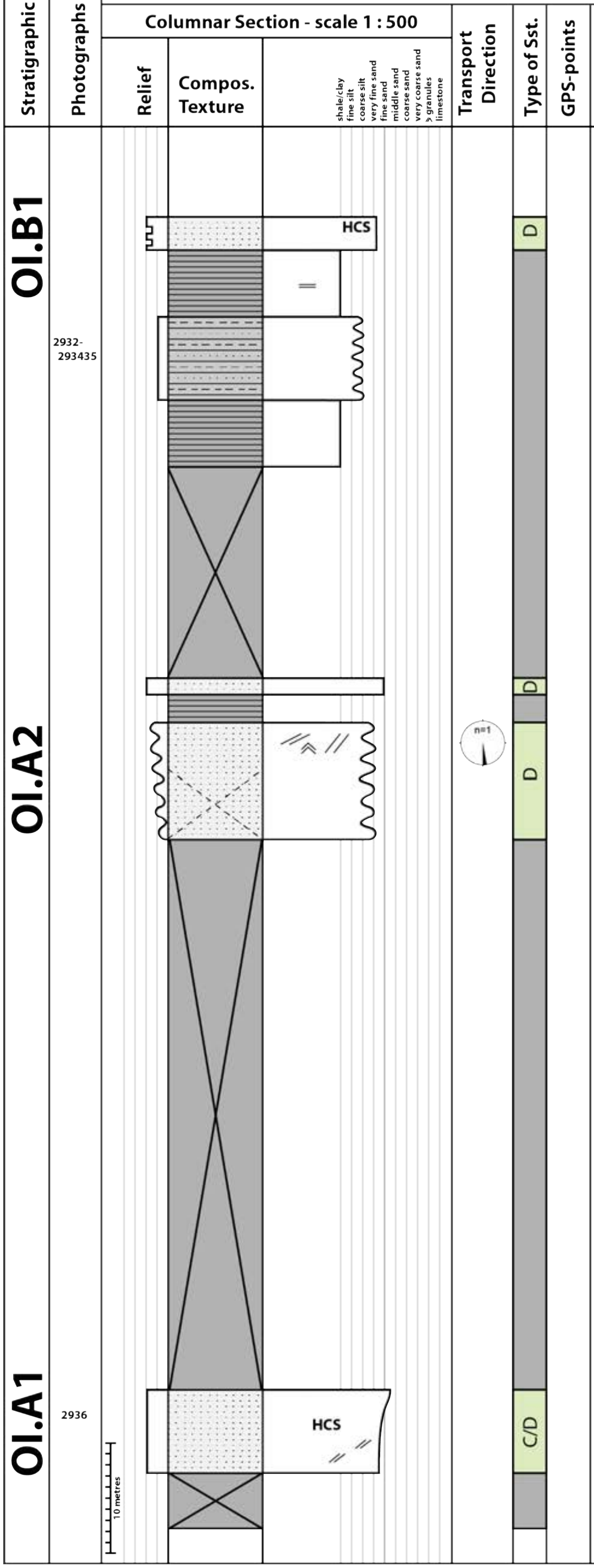

Additional DESCRIPTION and remarks

Continued on page 2-2
hummocky cross stratified very fine sandstone, beds and non-outcrop alternate of height 40 cm, no grading, well-sorted

grey shale interval, no iron carbonate or limestone beds found

interval with an alternation between shales (45%), silt (45%), and very fine sandstone (10%), cross-beds in sands, look like mini-hummocks, see photos

grey shale interval

non-outcrop, most probably shales and no sandstone units missed

interval with alternating soft (silt) and harder (very fine sandstone) beds with height 50 cm-100 cm, mega-foresets with height up to 40 cm with small back-flow ripples of height ~1 cm, BP 115/54 S

dominantly fine sandstone unit with large foresets and in top part one interval of hummocky cross-stratification, sorting good, red oxidised feldspar, qtz, mica (no black minerals), very hard quarzite

End of Section outcrop continues with bit lower outcrop grade untill limestone J1, where nice sandstone crop out

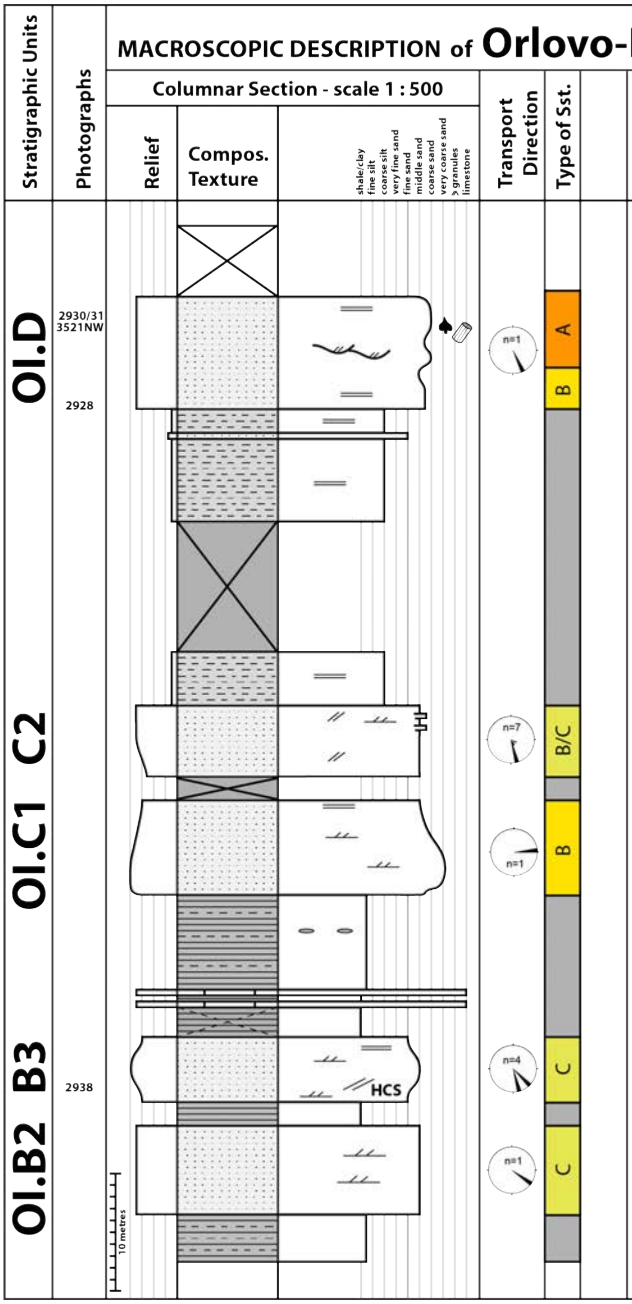

Additional DESCRIPTION and remarks

Top of Section end at dam, no directly continuous outcrop

Interval with coarse poorly sorted beds with tree trunk prints and intervals of fine to middle well-sorted sandstone with through cross-stratification, outcrop not very good looks disturbed by fault, bottom sharp, unit in total poorly sorted and CU, BP 119/50 S

Silt interval with some very thin very fine to fine sandstone beds

Middle to coarse sandstone unit with cross-beds of height up to 30 cm, most are smaller, all point to same direction (outcrop observation), sorting is moderate to good, black minerals, qtz, mic, BP 120/50 S

Fining upwards from very coarse to middle sandstone unit, with mega-ripples up to height of 30 cm (average 15 cm), coarser parts seem to contain most quartz, BP 133/44 S

Silty shale interval, dark colour, some iron carbonates

Hard limestone, mud- to wackestone, some parts packestone, bioclasts not identifiable in field, dark grey colour, 2 beds of 50 cm, possibly limestone K1

Middle to fine sandstone unit with ripples up to height of 20 cm, also horizontal or low angle lamination, especially in bottom part, moderate sorting, lateral very continuous (visible 200-300 m), in middle of unit up to coarse sand

Grey shale

Middle sand unit full of large foresets of height 5 to 20 cm, relatively well-sorted, high to middle spherical angular grains, Qtz, feldspar, black mineral, little mica, no change in grain size through unit, only one direction found, BP 117/48 S

Silty shale unit, grey colour, micas

Continuation from page 1-2

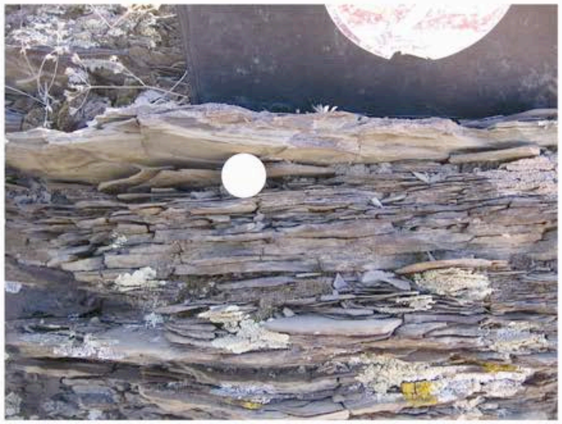

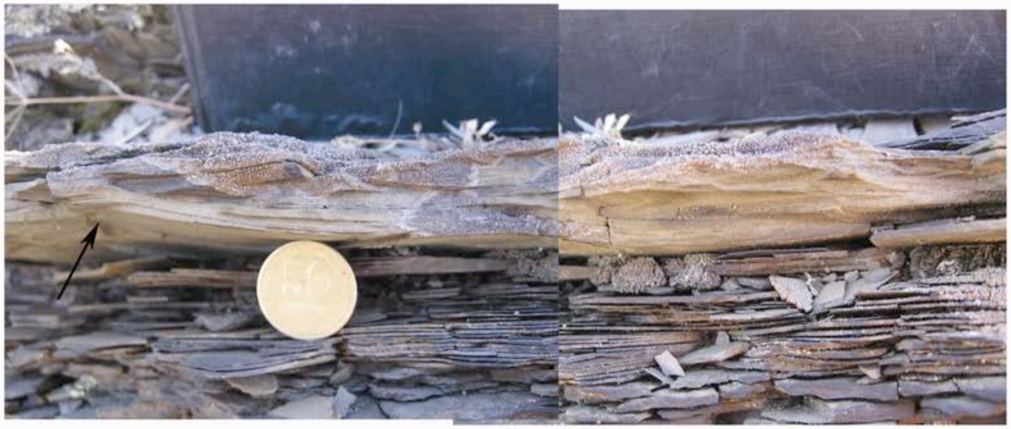

Photos 2932 (left) and 2934/35 Silty shale interval with some very fine sandstones intercalating, on this picture some very small cross-beds can be seen that seem to be induced by wave action.

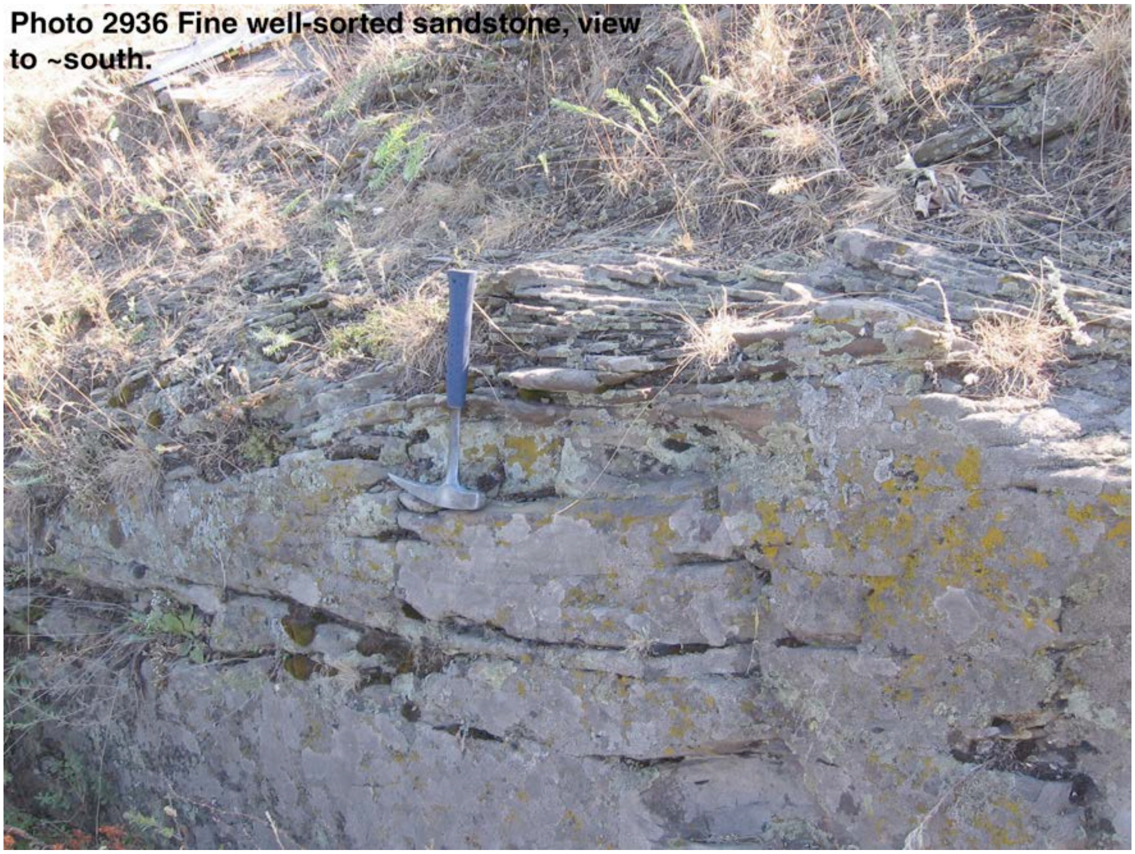

Photo 2936 Fine well-sorted sandstone, view to ~south.

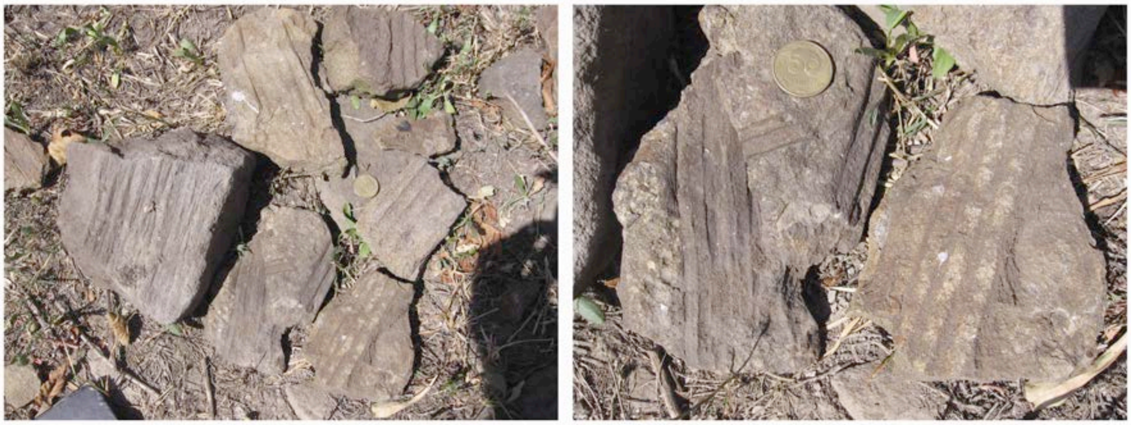

Photos 2930 (left) and 2931 Tree trunk prints in coarse sandstones.

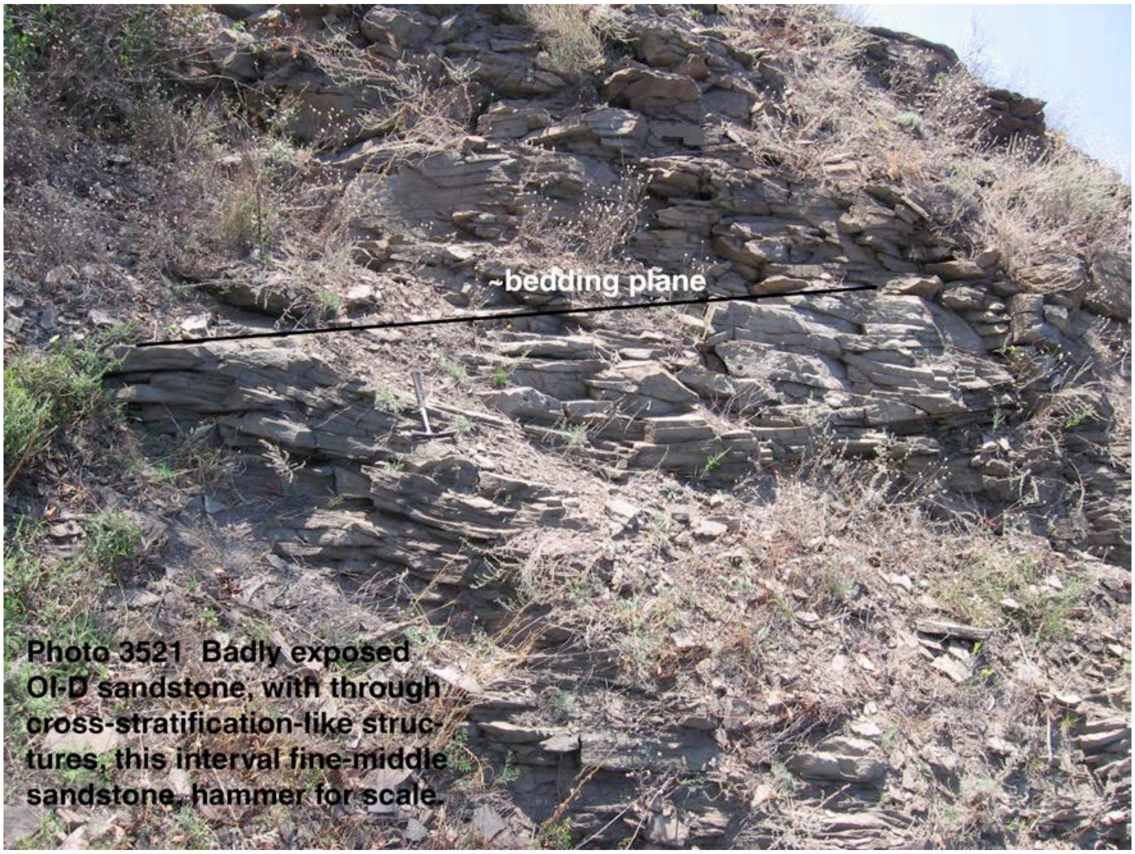

Photo 3521 Badly exposed OI-D sandstone, with through cross-stratification-like structures, this interval fine-middle sandstone, hammer for scale.

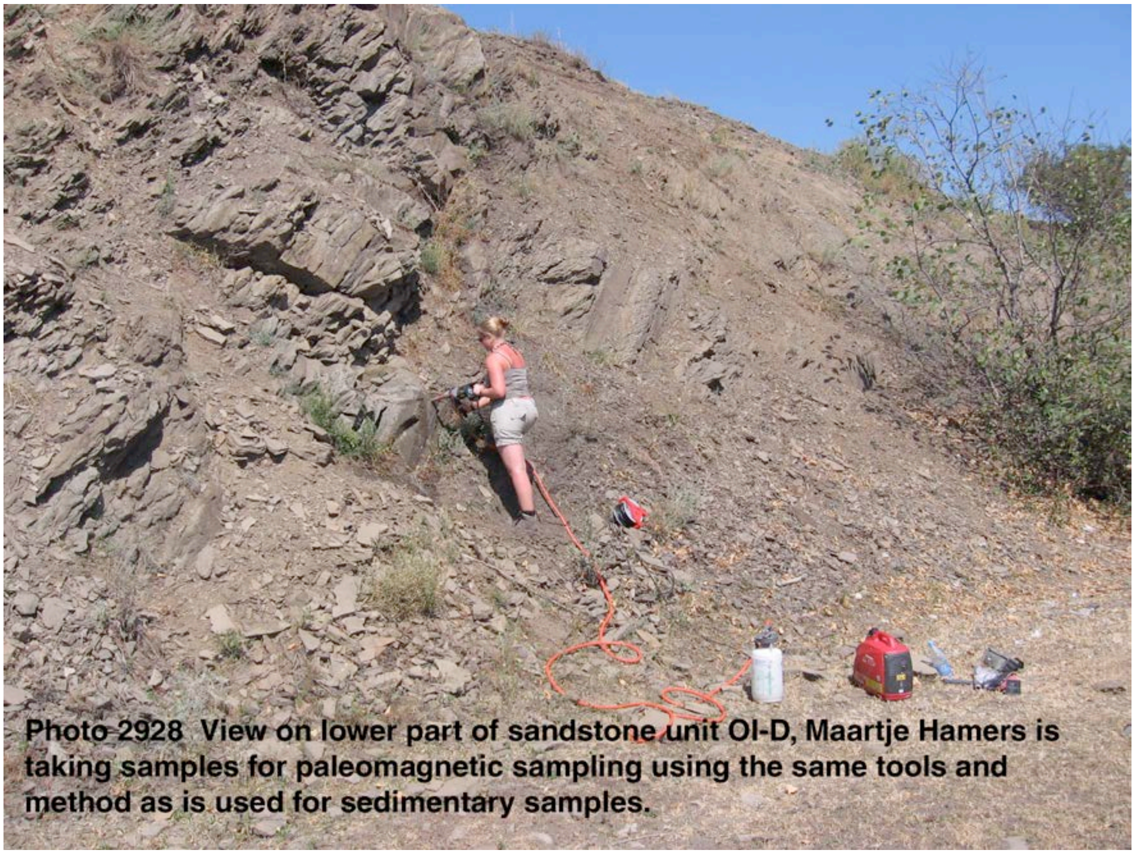

Photo 2928 View on lower part of sandstone unit OI-D, Maartje Hamers is taking samples for paleomagnetic sampling using the same tools and method as is used for sedimentary samples.

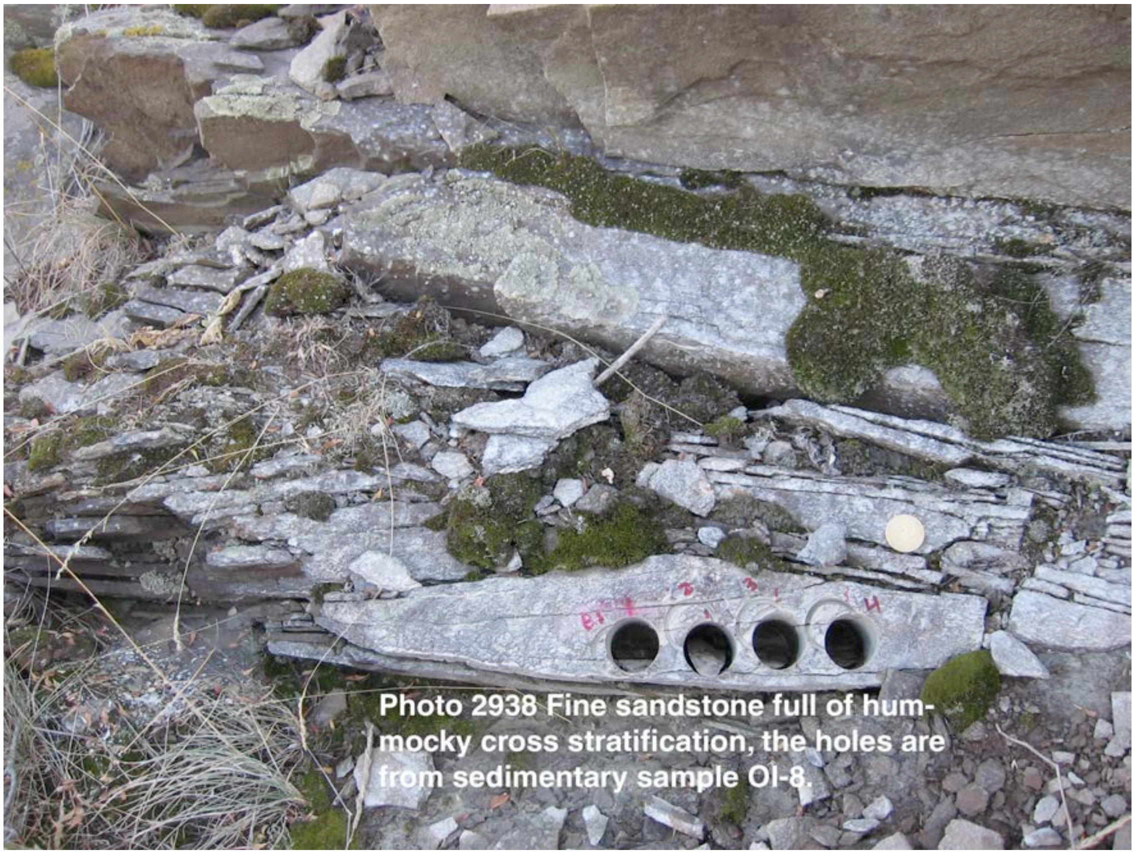

Photo 2938 Fine sandstone full of hummocky cross stratification, the holes are from sedimentary sample OI-8.

Section: ***Pervomaysk***

Location: 37U 0459526 UTM 5334117, central area

Situation: The Pervomaysk sandstone is named after the nearby city Первомайск (Pervomaysk). To reach the sandstone a small paved road that starts to the south on the west side of the river from the bridge between the cities Первомайск (Pervomaysk) and Теплогорск (Teplogorsk) has to be followed for a while. At a split to the southwest and south a very small bushy footpath starts between two houses to the west. The sandstone is a few hundreds of metres to the west along this path.

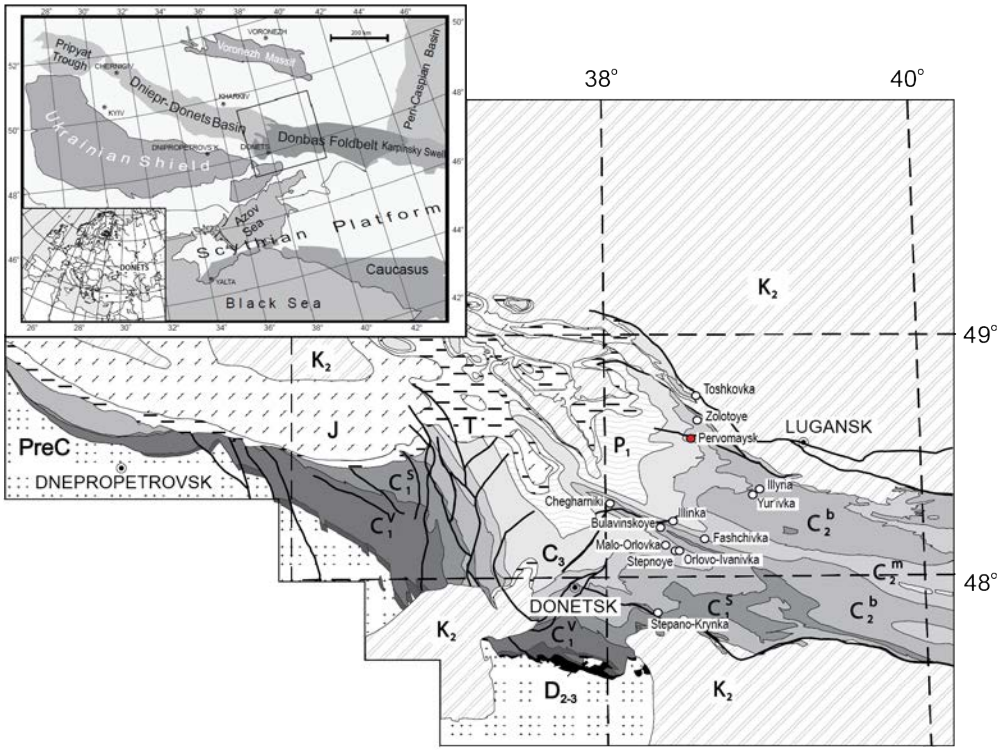

Age: **upper Moscovian, C_2^7.**

% Sand: **n/a**

Thickness: **10 m**

Sedimentology:

The Pervomaysk sandstone is characterised by features of coarsening upwards to the middle and fining upwards to the top of the unit. The lower fine to middle sand part is characterised by current induced cross bedding and belongs to Group B or C. The middle part is much coarser grained and less sorted. Big trough structures and tree trunk prints occur, and this part must belong to Group A. The top part is finer again and full of mega and intermediate scale current induced cross bedding. This part is grouped within Group B. The three measured paleocurrent directions aim at an average southeast direction with around 100° variability. The unit fits within a short outcropping stratigraphy below it. This consists of mainly shales with a limestone (see photo

in log) and just below the Pervomaysk sandstone a transition interval of sandy silts occurs.

Environmental interpretation:

The Pervomaysk sandstone shows a change from middle to upper shoreface to clearly fluvial environments and back to deltaic to upper shoreface environments in the top. It can therefore be seen as a 4th order sea-level lowstand deposit.

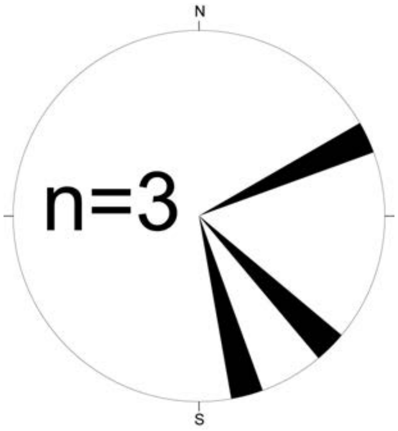

Figure XIK1. Rose diagram of the three measured paleocurrent directions in the Orlovo-Ivanovskaia section. Petals in groups of 10°.

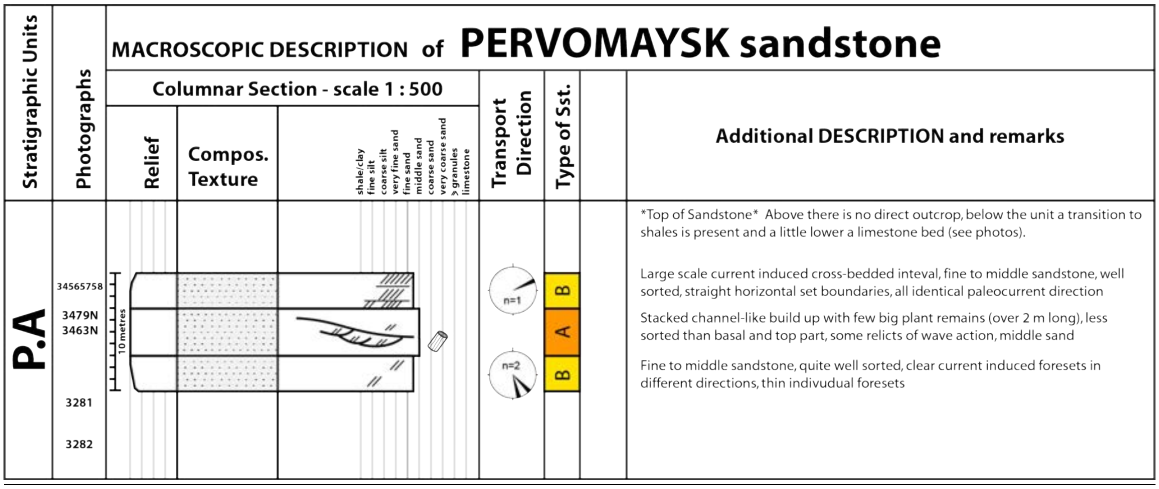

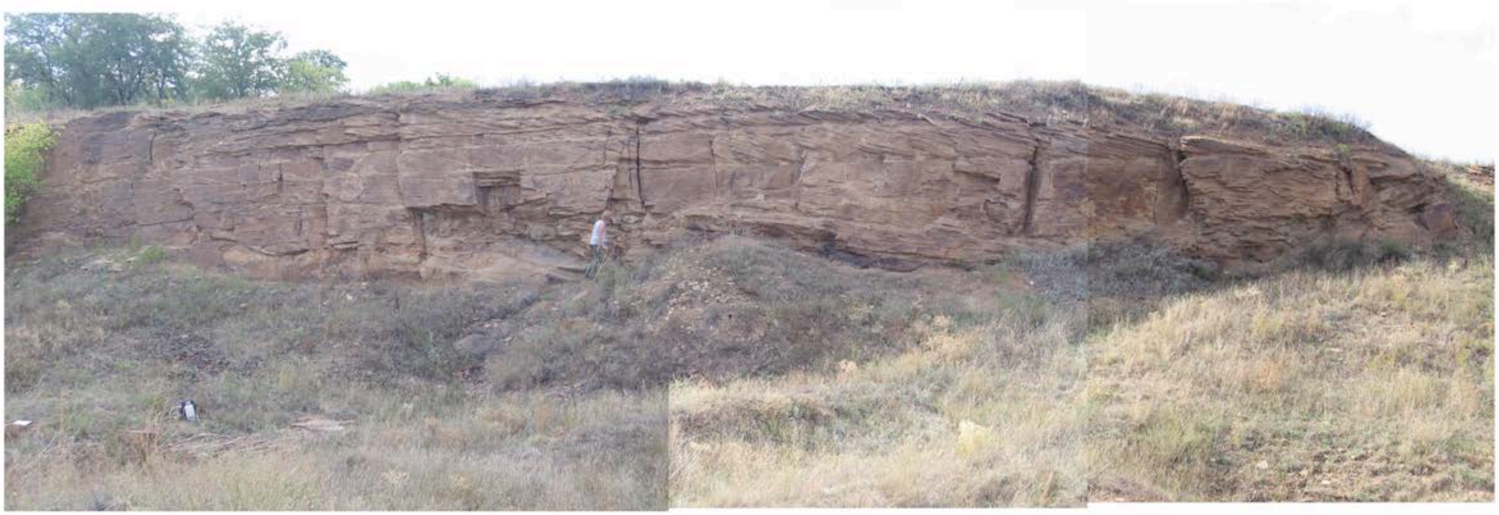

Photo 3456/57/58 View to west on middle and upper part of the Pervomaisk sandstone.

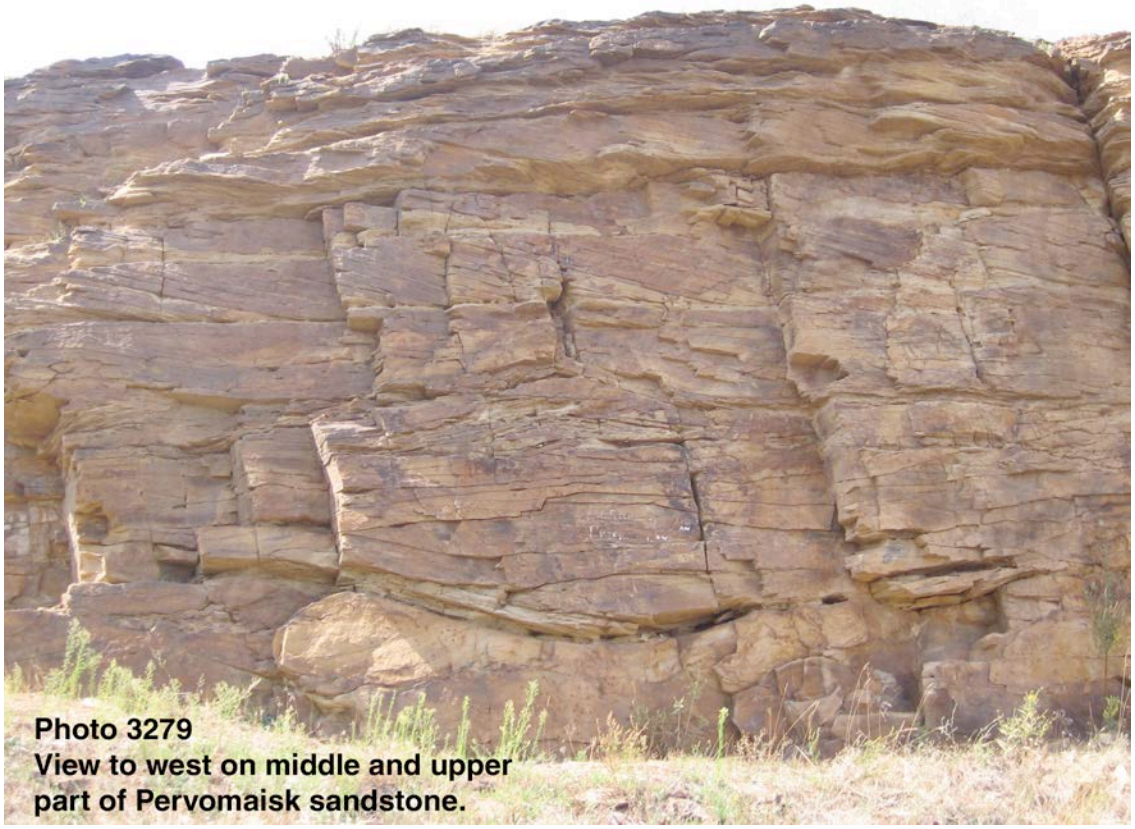

Photo 3279
View to west on middle and upper part of Pervomaisk sandstone.

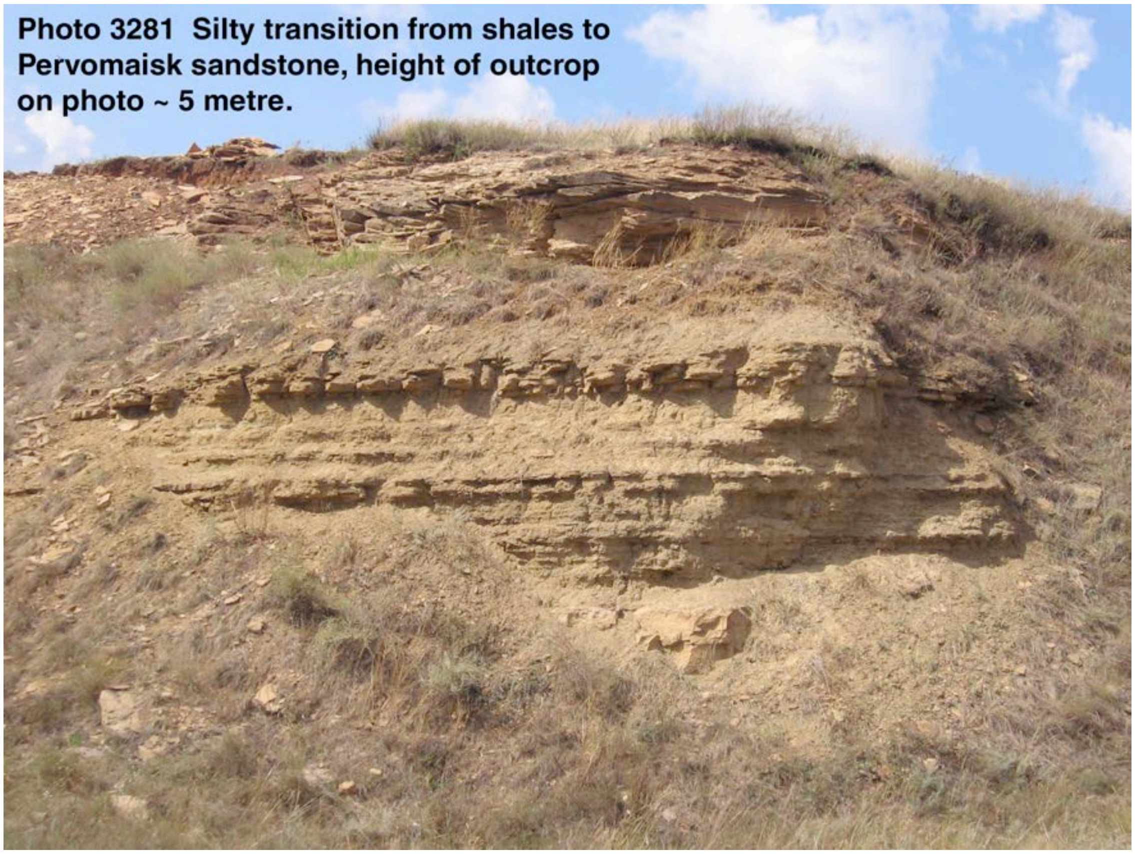

Photo 3281 Silty transition from shales to Pervomaisk sandstone, height of outcrop on photo ~ 5 metre.

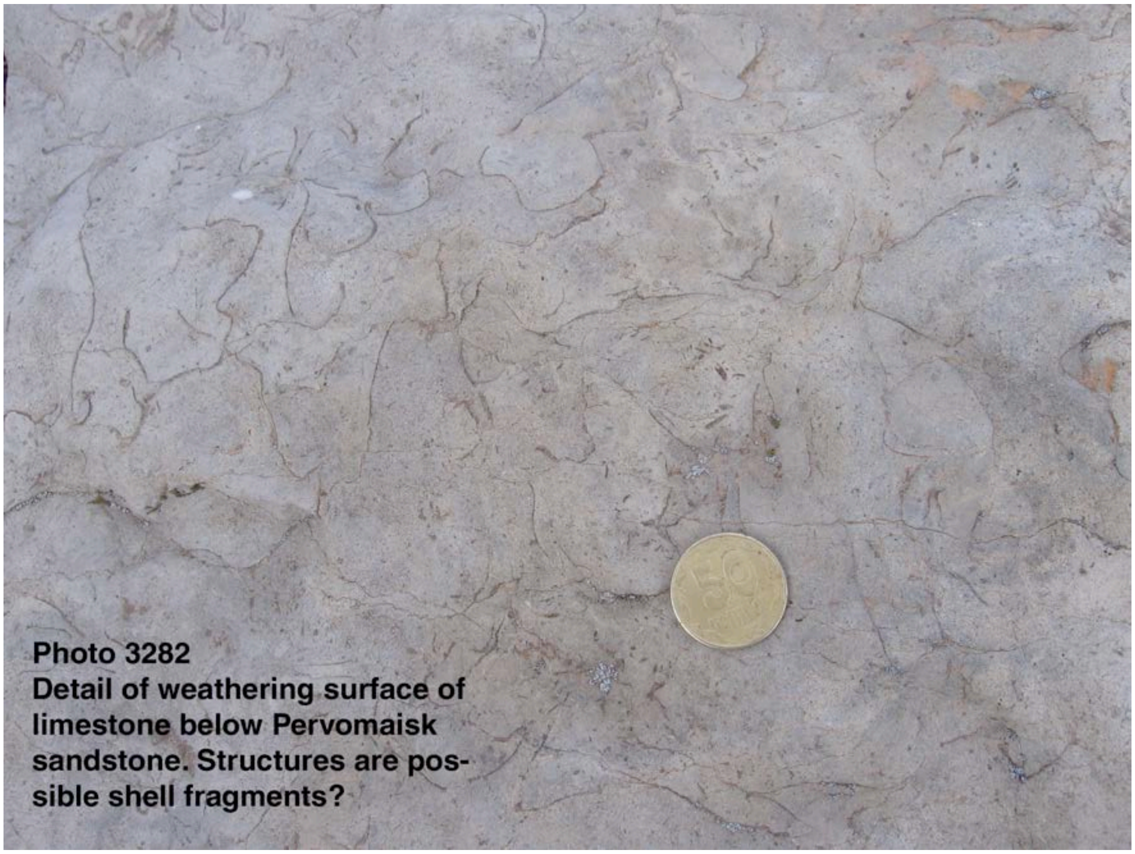

Photo 3282
Detail of weathering surface of limestone below Pervomaisk sandstone. Structures are possible shell fragments?

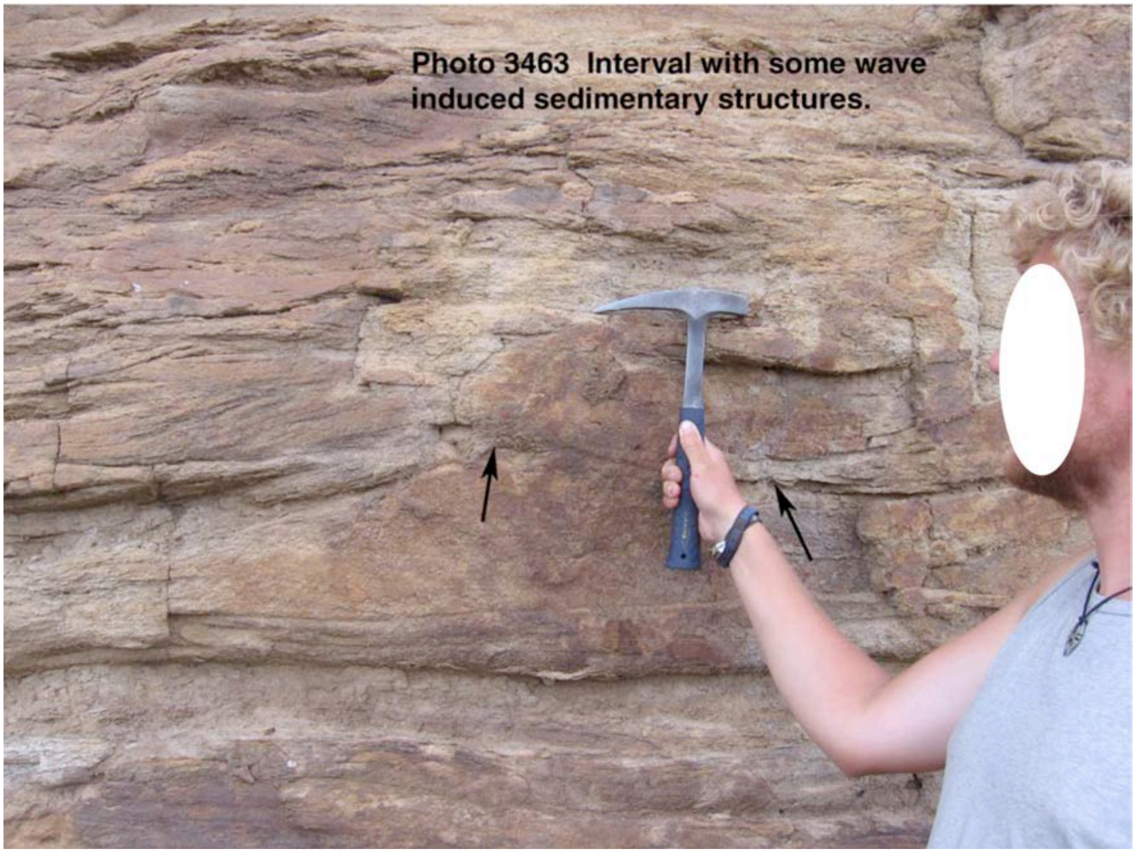

Photo 3463 Interval with some wave induced sedimentary structures.

Section: ***Stepano-Krynka***

Location: 37T 0450178 UTM 5306144, southern area

Situation: The Stepano-Krynka section is named after the village called Стелано-Крынка, that is situated around 3 kilometre north-east of the section. The section can be reached from the road between Стелано-Крынка and Кутейниково and is situated just north of a small lake a few hundreds of meters at the west side of the road.

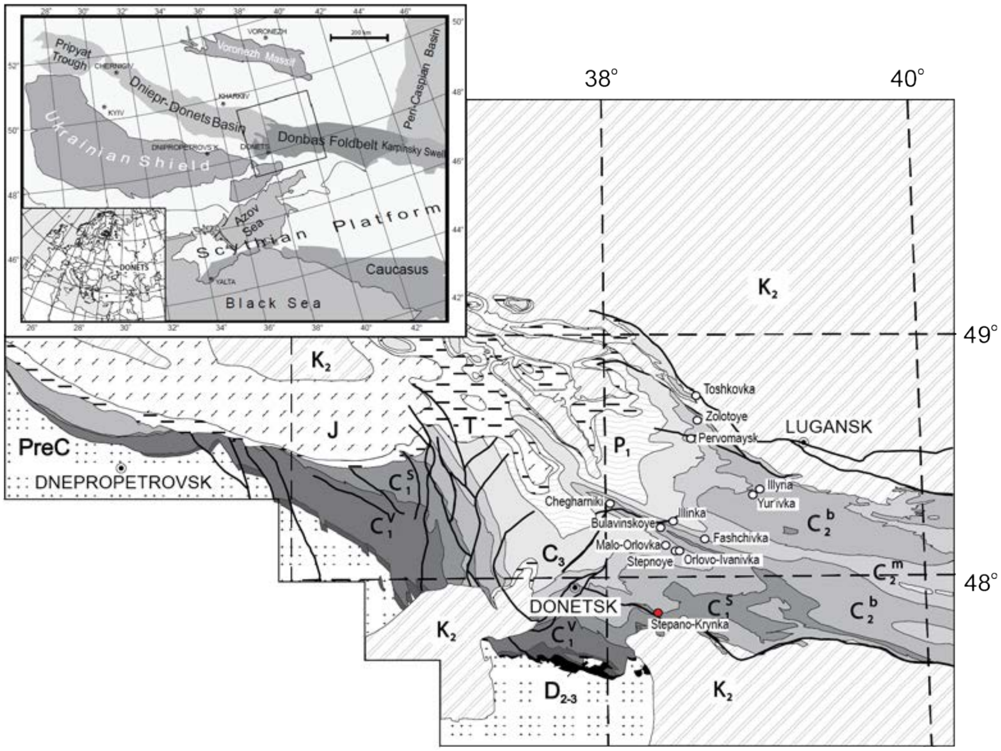

Age: **lower Bashkirian, $C_1^n e_1$ or e_2** (Note that on the bigger geological map this section is positioned in two suites younger, however the more detailed map clearly showed the in this report used age)

% Sand: **77 %**

Thickness: **65 m**

Sedimentology:

The Stepano-Krynka section is characterised by a very high amount of sandstone versus clays and silts, that is not seen in any of the other studied sections. Most sandstones in the section are characterised as well-sorted middle sand that are full of intermediate scale current induced cross bedding. These sandstones belong to Group B or sometimes C. Short intervals exist of coarser and less sorted sandstones with tree trunk prints. These sandstones belong to Group A. One relatively thin sandstone (SK.2A) is present that is much finer grained and contains hummocky cross stratification. This unit belongs to Group D. There is not a lot of variation in paleocurrent directions measured that are mainly pointing at an east-southeast direction (see Figure XSK). One coal layer

has been found that however could not be sampled due to its very weathered character.

Environmental interpretation:

The sandstone characteristics of the Stepano-Krynka section aim at a deltaic to upper shoreface depositional environment with intercalations of fluvial regimes. Between sandstone unit SK.2B and SK.1 there was a short period of deeper water environment, interpreted to be a lower shoreface environment. The low variability in paleocurrents might indicate the open water environments in which the sediments where deposited, instead of more continental (fluvial) systems. This section is interpreted as a 3rd order but even also 2nd order sea level low-stand.

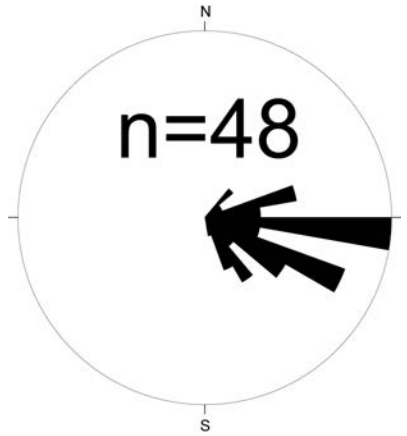

Figure XSK. Rose diagram of all measured paleocurrent directions in the Stepano-Krynka section. Petals given in groups of 10°, largest petal represents 10 data points and 20% of the total data.

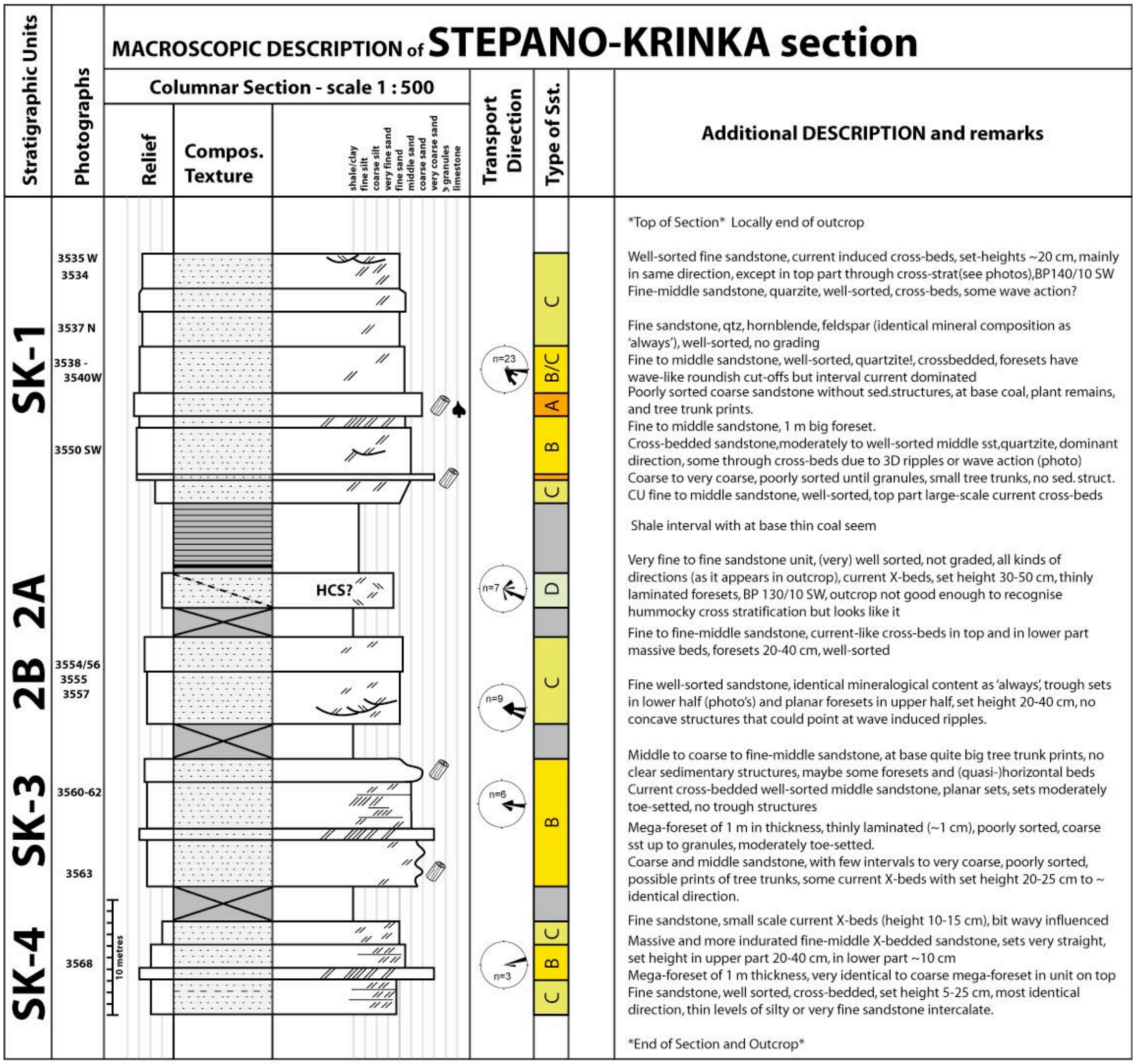
MACROSCOPIC DESCRIPTION of STEPANO-KRINKA section
Stratigraphic Units
Photographs
Columnar Section - scale 1 : 500
Relief
Compos. Texture
shale/clay
fine silt
coarse silt
very fine sand
fine sand
middle sand
coarse sand
very coarse sand
granules
limestone
Transport Direction
Type of Sst.
Additional DESCRIPTION and remarks
SK-1
2A
2B
SK-3
SK-4
3535 W
3534
3537 N
3538 - 3540W
3550 SW
3554/56
3555
3557
3560-62
3563
3568
10 metres
HCS?
n=23
n=7
n=9
n=6
n=3
C
B/C
A
B
C
D
C
B
C
B
C
Top of Section Locally end of outcrop
Well-sorted fine sandstone, current induced cross-beds, set-heights ~20 cm, mainly in same direction, except in top part through cross-strat(see photos),BP140/10 SW
Fine-middle sandstone, quarzite, well-sorted, cross-beds, some wave action?
Fine sandstone, qtz, hornblende, feldspar (identical mineral composition as 'always'), well-sorted, no grading
Fine to middle sandstone, well-sorted, quartzite!, crossbedded, foresets have wave-like roundish cut-offs but interval current dominated
Poorly sorted coarse sandstone without sed.structures, at base coal, plant remains, and tree trunk prints.
Fine to middle sandstone, 1 m big foreset.
Cross-bedded sandstone,moderately to well-sorted middle sst,quartzite, dominant direction, some through cross-beds due to 3D ripples or wave action (photo)
Coarse to very coarse, poorly sorted until granules, small tree trunks, no sed. struct.
CU fine to middle sandstone, well-sorted, top part large-scale current cross-beds
Shale interval with at base thin coal seem
Very fine to fine sandstone unit, (very) well sorted, not graded, all kinds of directions (as it appears in outcrop), current X-beds, set height 30-50 cm, thinly laminated foresets, BP 130/10 SW, outcrop not good enough to recognise hummocky cross stratification but looks like it
Fine to fine-middle sandstone, current-like cross-beds in top and in lower part massive beds, foresets 20-40 cm, well-sorted
Fine well-sorted sandstone, identical mineralogical content as 'always', trough sets in lower half (photo's) and planar foresets in upper half, set height 20-40 cm, no concave structures that could point at wave induced ripples.
Middle to coarse to fine-middle sandstone, at base quite big tree trunk prints, no clear sedimentary structures, maybe some foresets and (quasi-)horizontal beds
Current cross-bedded well-sorted middle sandstone, planar sets, sets moderately toe-setted, no trough structures
Mega-foreset of 1 m in thickness, thinly laminated (~1 cm), poorly sorted, coarse sst up to granules, moderately toe-setted.
Coarse and middle sandstone, with few intervals to very coarse, poorly sorted, possible prints of tree trunks, some current X-beds with set height 20-25 cm to ~ identical direction.
Fine sandstone, small scale current X-beds (height 10-15 cm), bit wavy influenced
Massive and more indurated fine-middle X-bedded sandstone, sets very straight, set height in upper part 20-40 cm, in lower part ~10 cm
Mega-foreset of 1 m thickness, very identical to coarse mega-foreset in unit on top
Fine sandstone, well sorted, cross-bedded, set height 5-25 cm, most identical direction, thin levels of silty or very fine sandstone intercalate.
End of Section and Outcrop

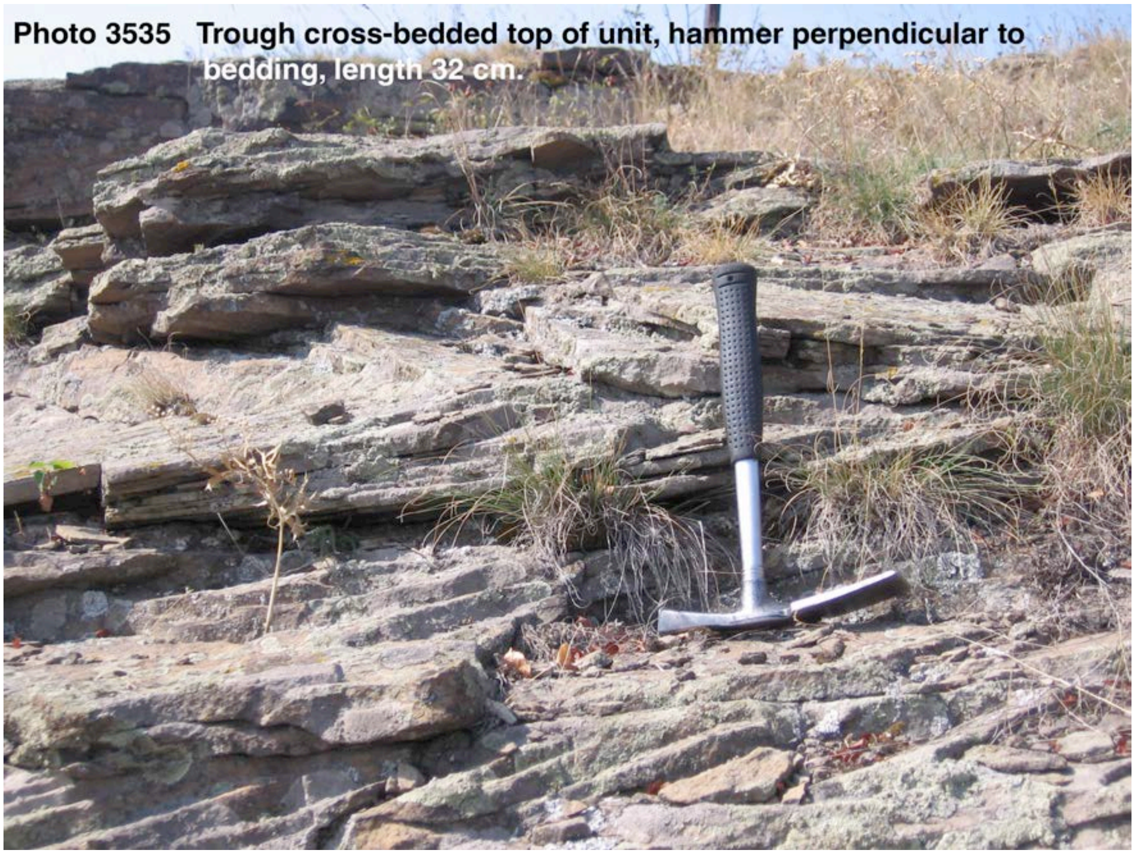

Photo 3535 Trough cross-bedded top of unit, hammer perpendicular to bedding, length 32 cm.

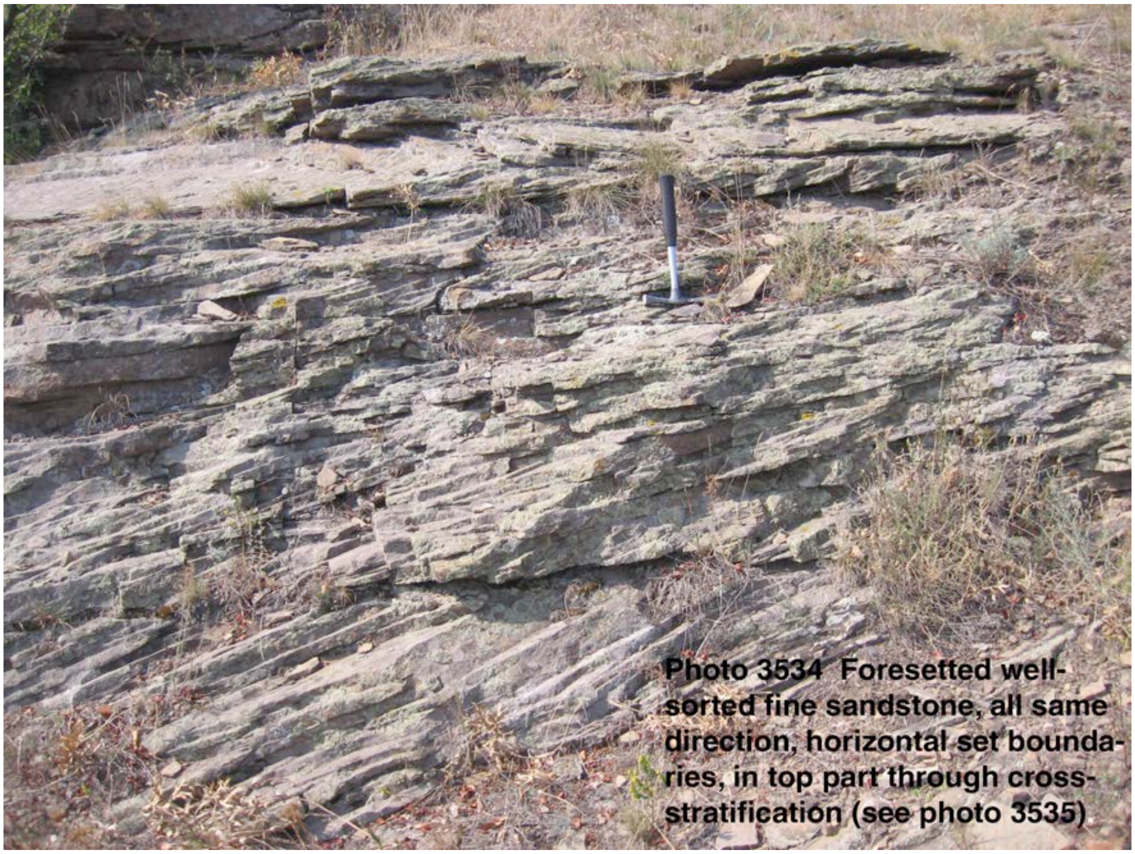

Photo 3534 Foresetted well-sorted fine sandstone, all same direction, horizontal set boundaries, in top part through cross-stratification (see photo 3535)

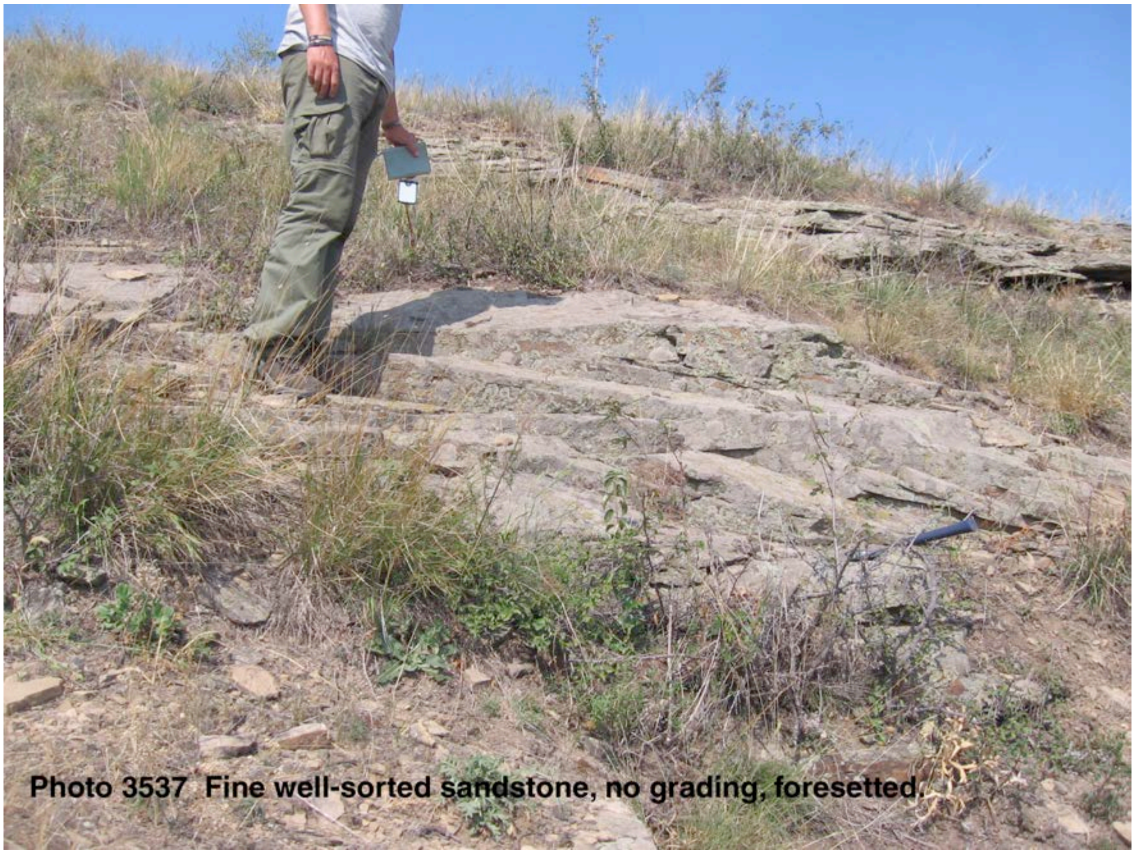

Photo 3537 Fine well-sorted sandstone, no grading, foresetted.

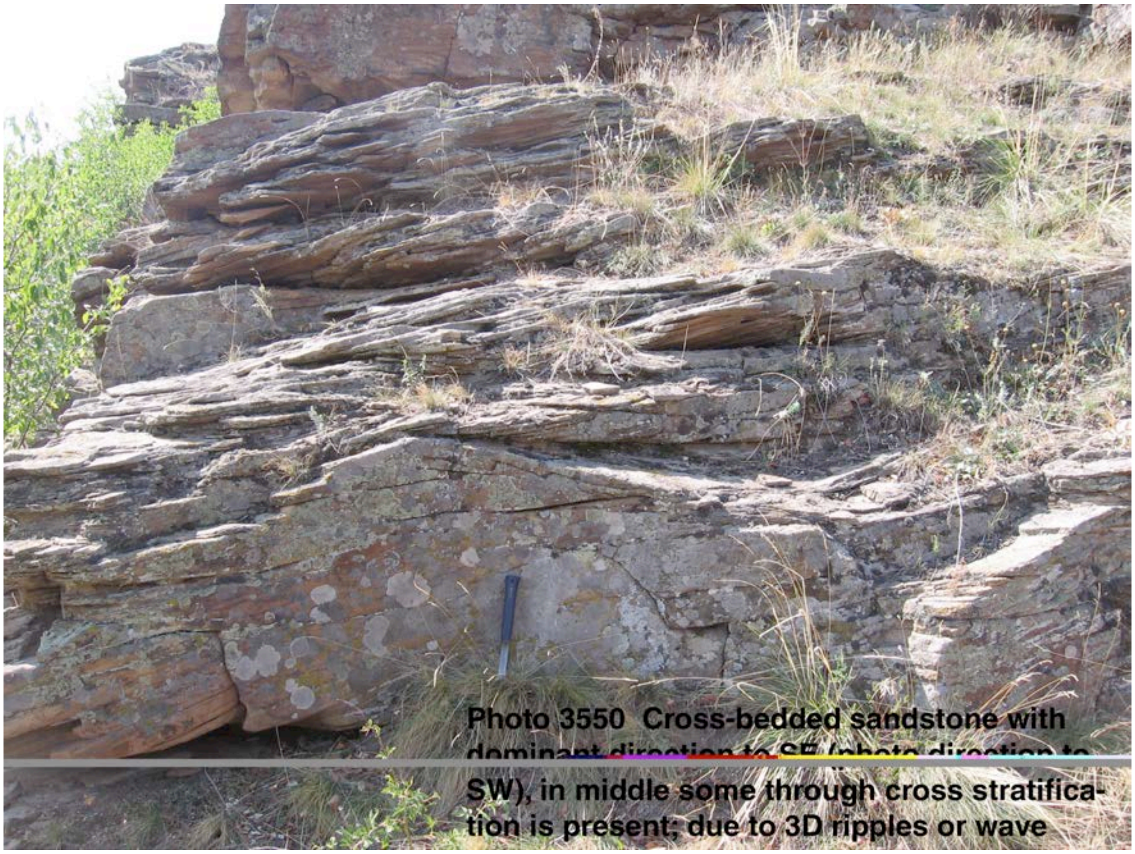

Photo 3550 Cross-bedded sandstone with dominant direction to SE (photo direction to SW), in middle some through cross stratification is present; due to 3D ripples or wave

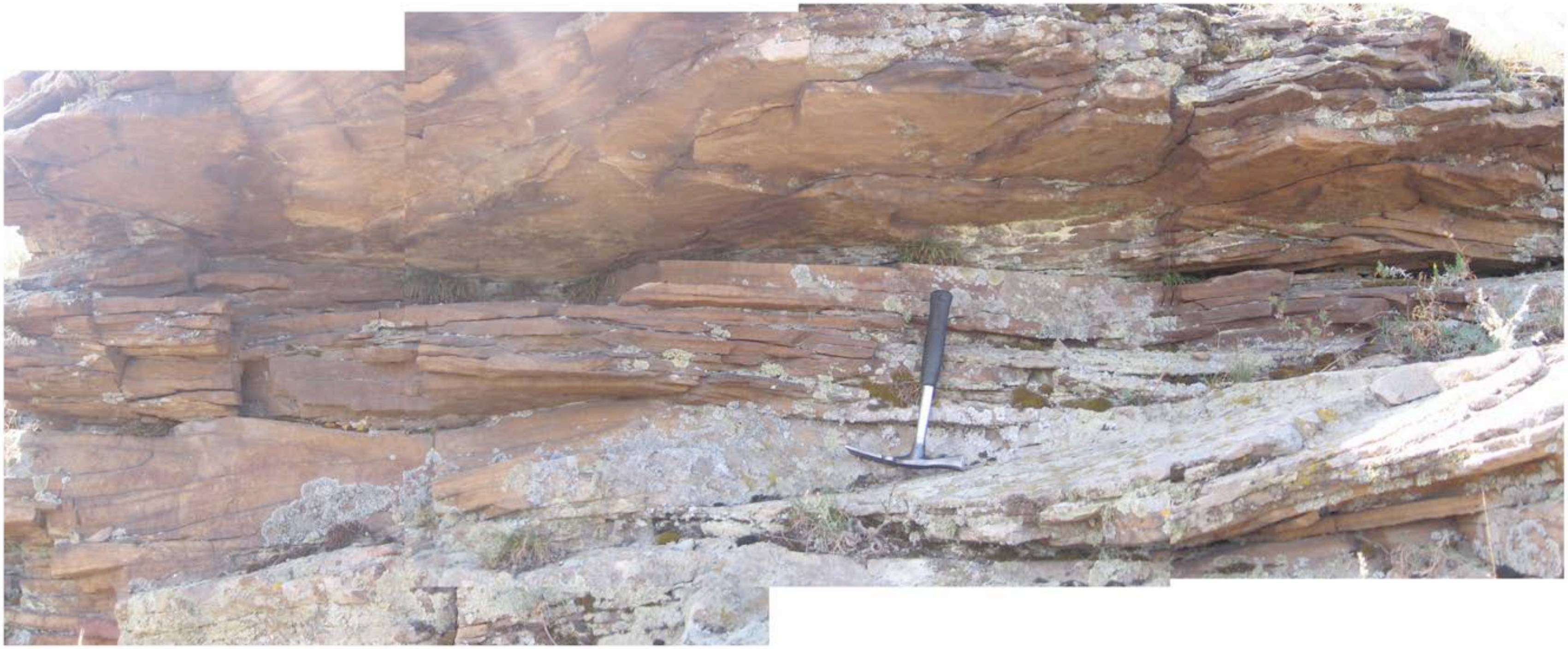

Photo 3538-39-40 Wave influenced current dominated well-sorted fine to middle sandstone.

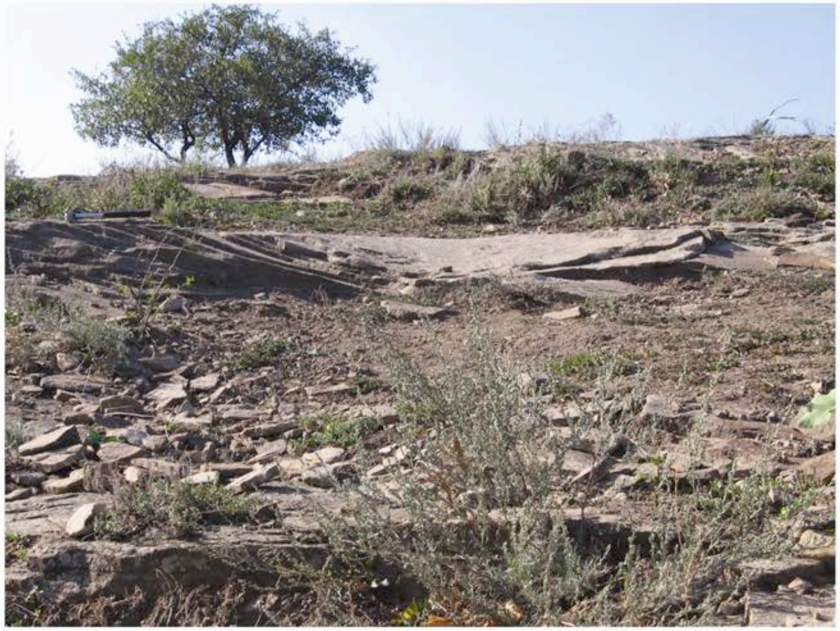

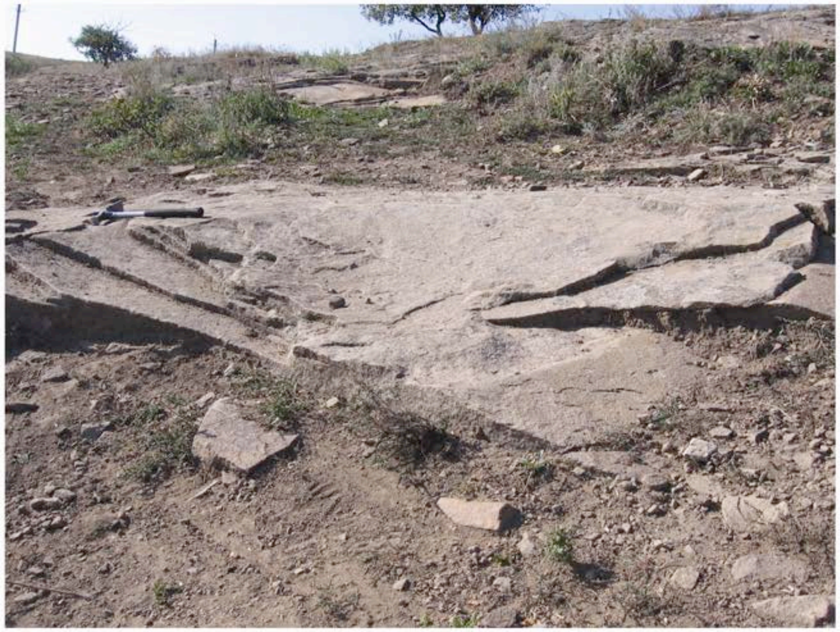

Photo 3556 (left) and 3554
3D mega-ripple in fine well-sorted sandstone, hammer for scale lays on bedding plane in both pictures.

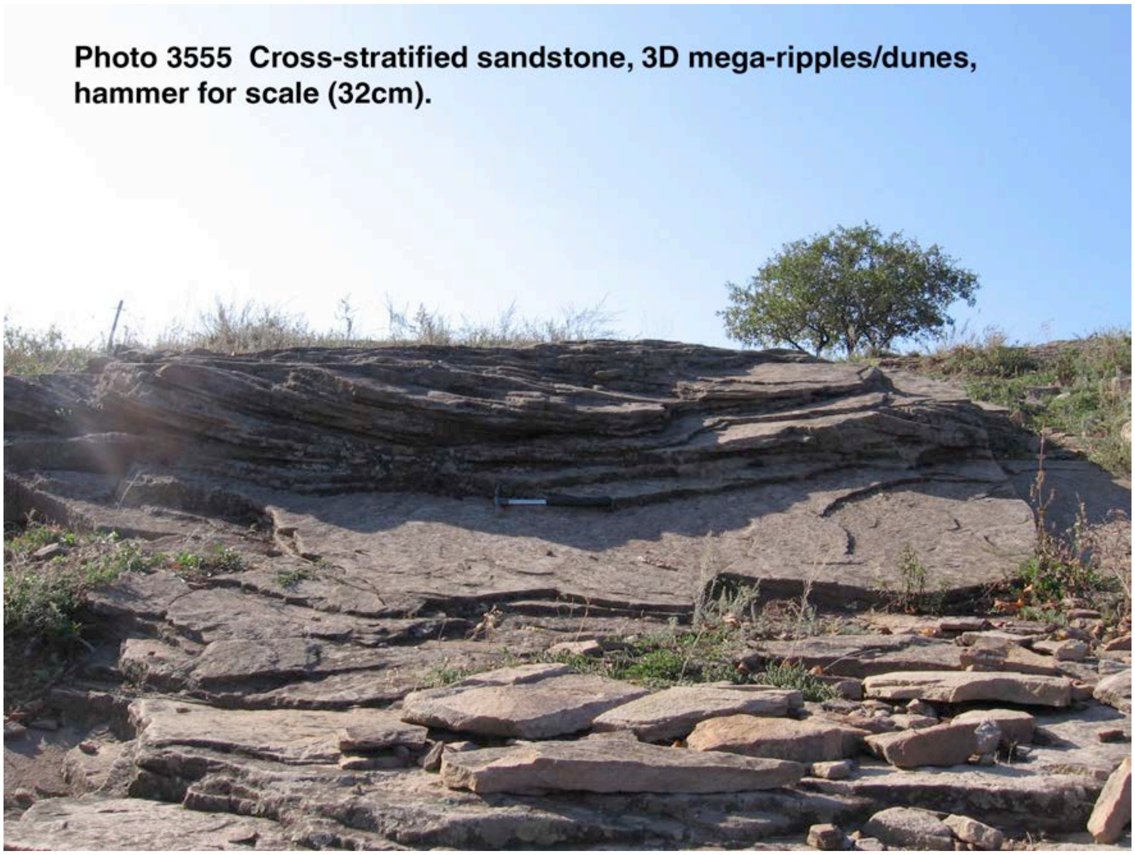

Photo 3555 Cross-stratified sandstone, 3D mega-ripples/dunes, hammer for scale (32cm).

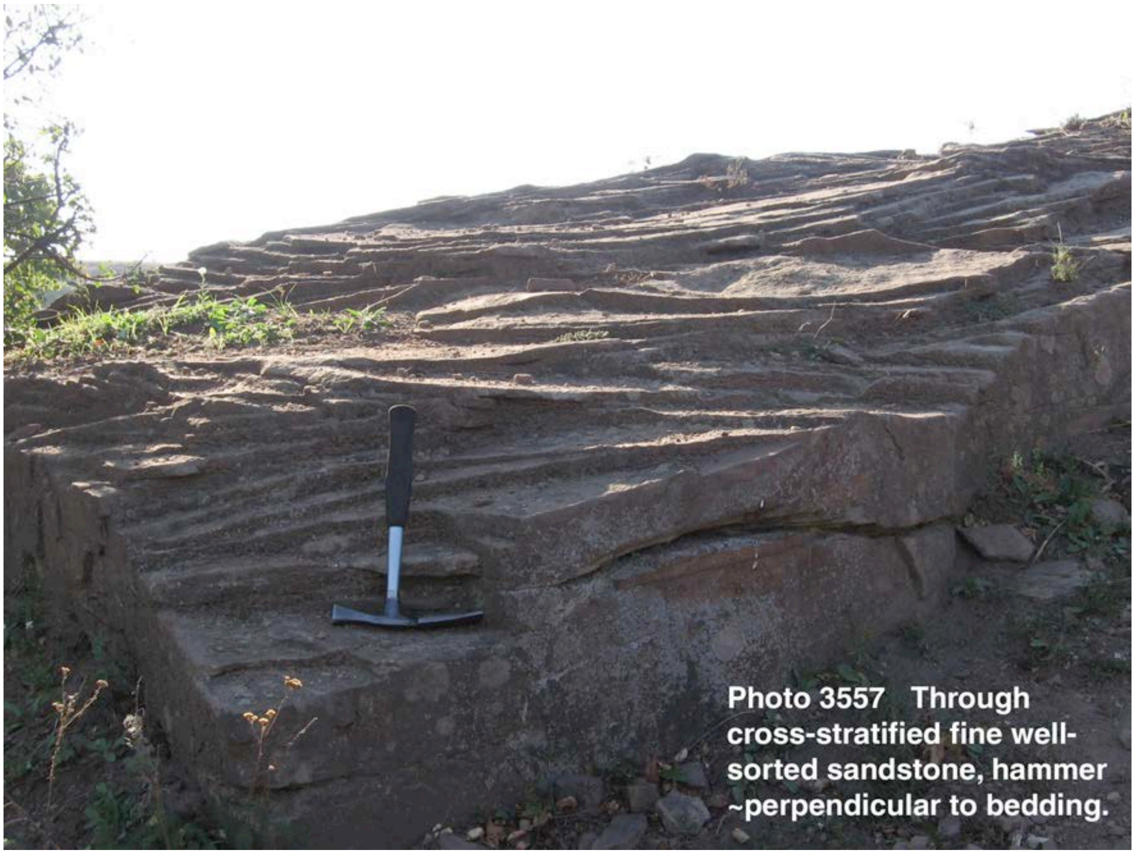

Photo 3557 Through cross-stratified fine well-sorted sandstone, hammer ~perpendicular to bedding.

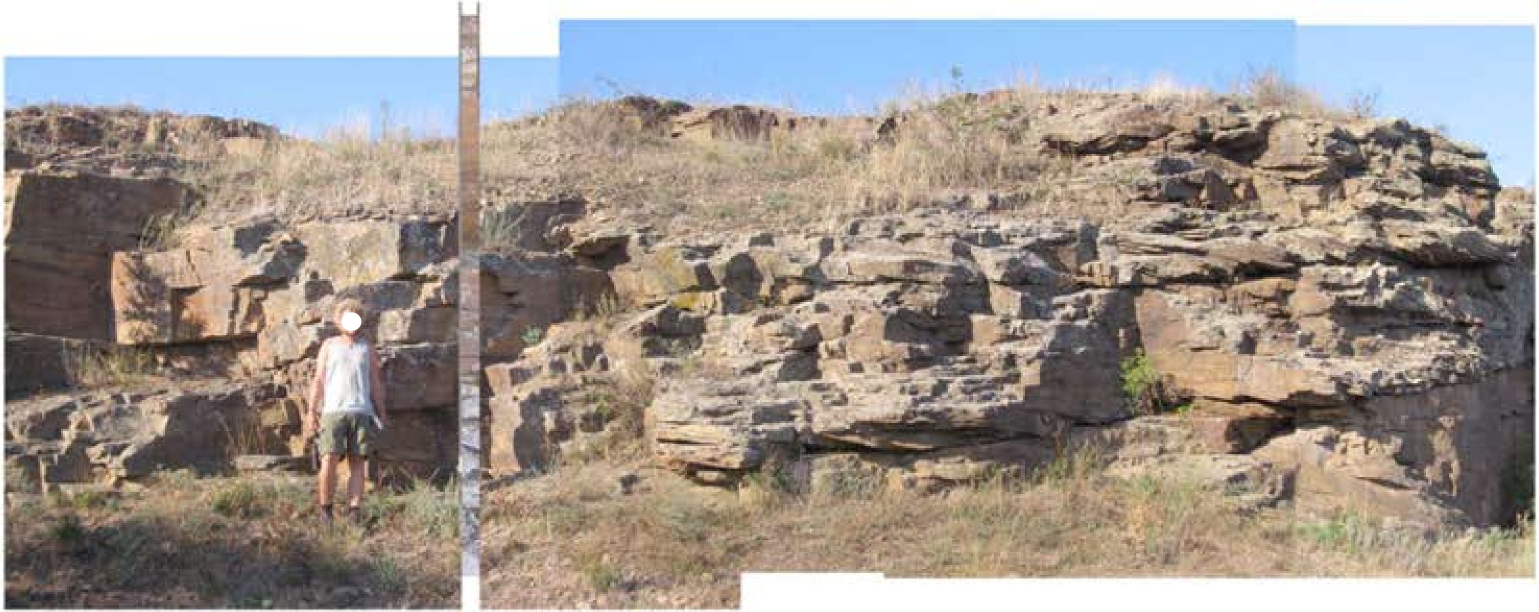

Photo 3560/61/62 View to ~NV of sandstone SK-3

e

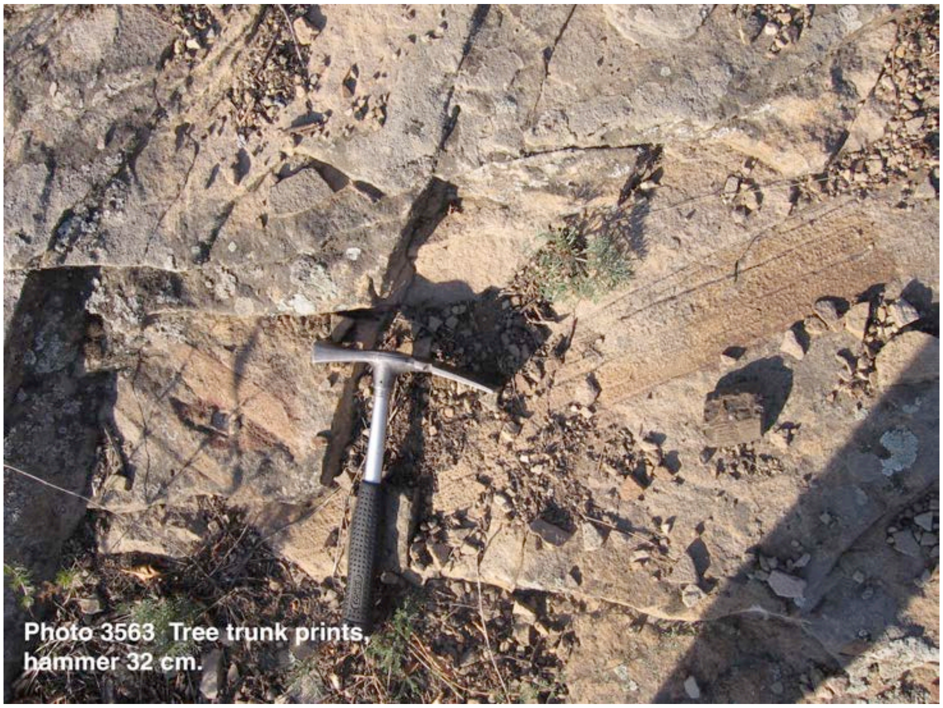

Photo 3563 Tree trunk prints, hammer 32 cm.

Photo 3568 View to N of sandstone SK-4, cross bedded in ~horizontal sets, one dominant direction to E

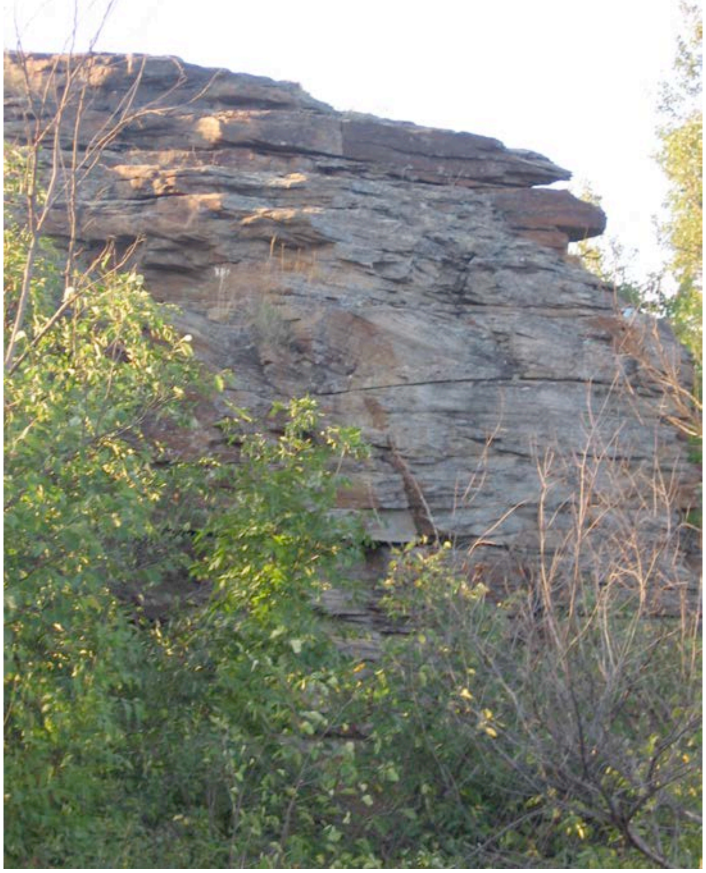

Section: ***Stepnoye***

Location: 37U 0458711 UTM 5333739, central area

Situation: The Stepnoye section is named after the settlement Смепное (Stepnoye) that is close to the village Михайловка (Michailovka). The section can be reached by taking the right split of the road (in bad shape) to the north from the latter village and going sharp right after passing small lakes. The section then starts in between the trees near the river and continuous along the road.

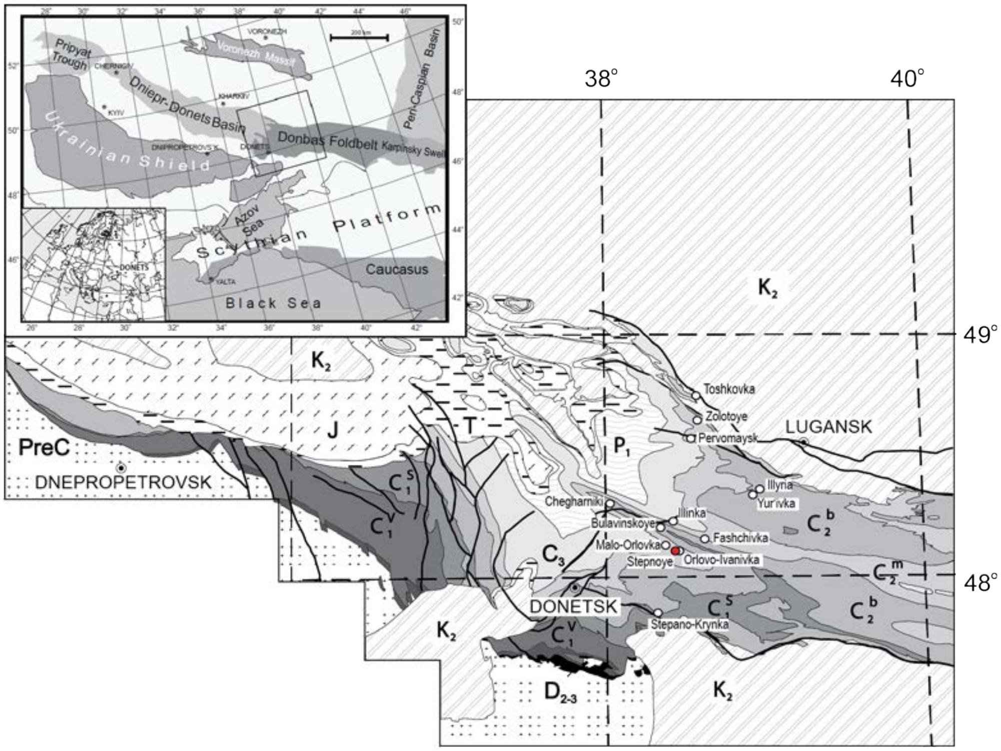

Age: **upper Bashkirian, C_2^5**

% Sand: **19 %**

Thickness: **157 m**

Sedimentology:

The Stepnoye section has a low amount of sand with respect to shales, which might be biased by the bad outcrop scale in between big sandstone units due to which some thinner sandstone might be missed. The sandstones present in the section consist mainly of moderately and well sorted middle to fine sand that have small to large scale current induced cross bedding. Paleocurrent measurements aim at a consistent southeast direction with around 120° of variation. These sandstones are grouped within sandstone Group B and C. At the base of the section one thin sandstone that is coarser and less sorted and that belongs to Group A occurs.

Environmental interpretation:

The sandstones of the Stepnoye section are interpreted to be deposited in a upper to lower shoreface setting (Group B and C) and the shales in a deeper environment. The sandstone interpretation is strengthened by the low amount of variation in paleocurrent directions measured (see Figure XST). One short interval of fluvial environment or close proximity of the continent is documented in the base of the section.

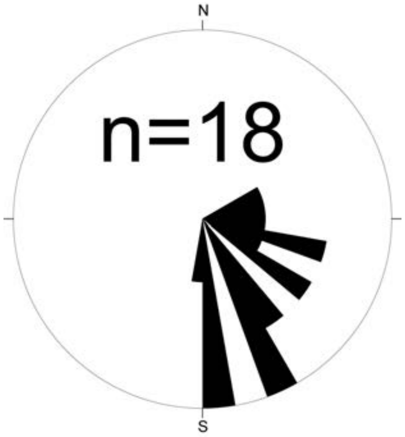

Figure XST. Rose diagram of all measured paleocurrent directions in the Stepnoye section. Petals given in groups of 10°, largest petal represents 3 data points and 16% of the total data.

MACROSCOPIC DESCRIPTION of STEPNOYE section 1/2

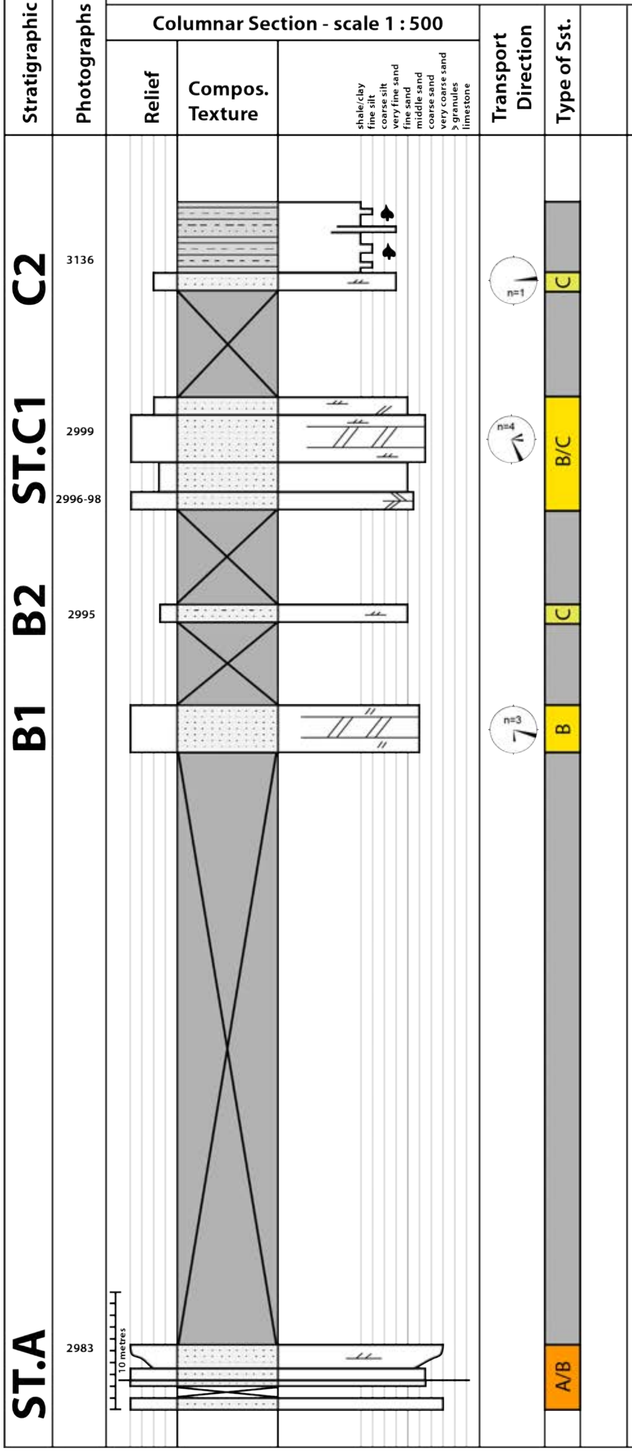

Additional DESCRIPTION and remarks

CONTINUED on page 2/2

silty shales with thin beds of coarse silt and very fine (at present) yellow sandstone, small plant remains, leafs < 5 cm, also rounded elongated curving things --> burrows

small-scale foreset bearing very fine sandstone unit with two paleocurrent directions (outcrop observation), BP 118/40 S

non-outcrop interval, except for one thin sandstone

fine sandstone with foresets of ~60 cm height and ~10 cm scale, same direction as bed below

middle sand with small foresets (height 5-10 cm) and big foreset of height 1.5 m, 50% qtz, 30% oxidised feldspar, few black minerals, one direction, clay drapes on set boundaries, BP 106/45 S

fine to middle sandstone with herringbone-like structure or at least directly on top of each other two directions, sorting not visible on outcrop scale, BP 118/44 S

fine sandstone with current ripples of height 5-10 cm, with two direction (outcrop obs.) shales and silts between sandstone beds, remnants of clay drapes floating in sandstone (photo)

moderately sorted middle sand (fine to coarse), 50% qtz, rest oxidised feldspar and black minerals, large scale foresets of height up to 40 cm, few in opposite direction, some parts thinly laminated foresets (thickness 2 cm) due to clay drapes?, other foresets massive, BP 115/54 S

non-outcrop, no palynological sampling possible

middle to coarse sandstone with poor sorting, grains in some beds up to small pebbles, cross-beds of flow and waves (photo), very hard sandstone (quartzite) disturbed by faults through thin coal seem in middle, possibly two flow directions?, in middle no structures recognised, maybe channel-like, BP 113/46 S

Base of Section

MACROSCOPIC DESCRIPTION of STEPNOYE section 2/2

Stratigraphic Units | Photographs | Columnar Section - scale 1 : 500 (Relief; Compos. Texture; shale/clay, fine silt, coarse silt, very fine sand, fine sand, middle sand, coarse sand, very coarse sand, granules, limestone) | Transport Direction | Type of Sst. | Additional DESCRIPTION and remarks

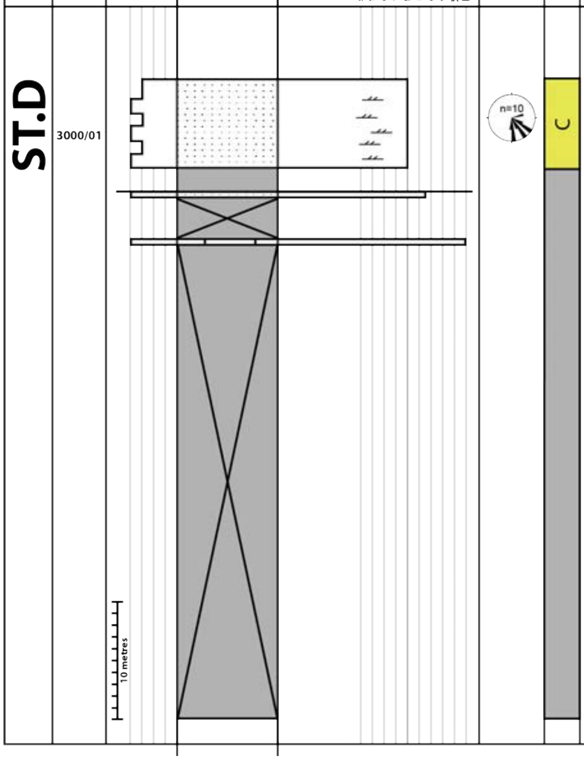

Additional DESCRIPTION and remarks

Top of section few more sandstone-units on top

Fine sandstone unit build up of small scale foresets of height ~10 cm, current ripples, well sorted, 40% Qtz, 35% oxides feldspar, 30% black minerals, no clay drapes recognised, but foreset laminae well-separated, all current same direction, no channell-like structures, quite continuous in outcrop, no grading in foreset, BP 108/54 S

Fault plane directly on top of thin sandstone, coarse to very coarse sandstone, horizontal laminated or low angle, lamination height ~3 cm, clay flakes floating in sandstone (size 1 cm), one fine sandstone with transported crinoids
LIMESTONE; black mud- to wackestone with crinoids (size 1 mm) and brachiopods

Non-outcrop except for one thin sandstone

Continuation from page 1-2

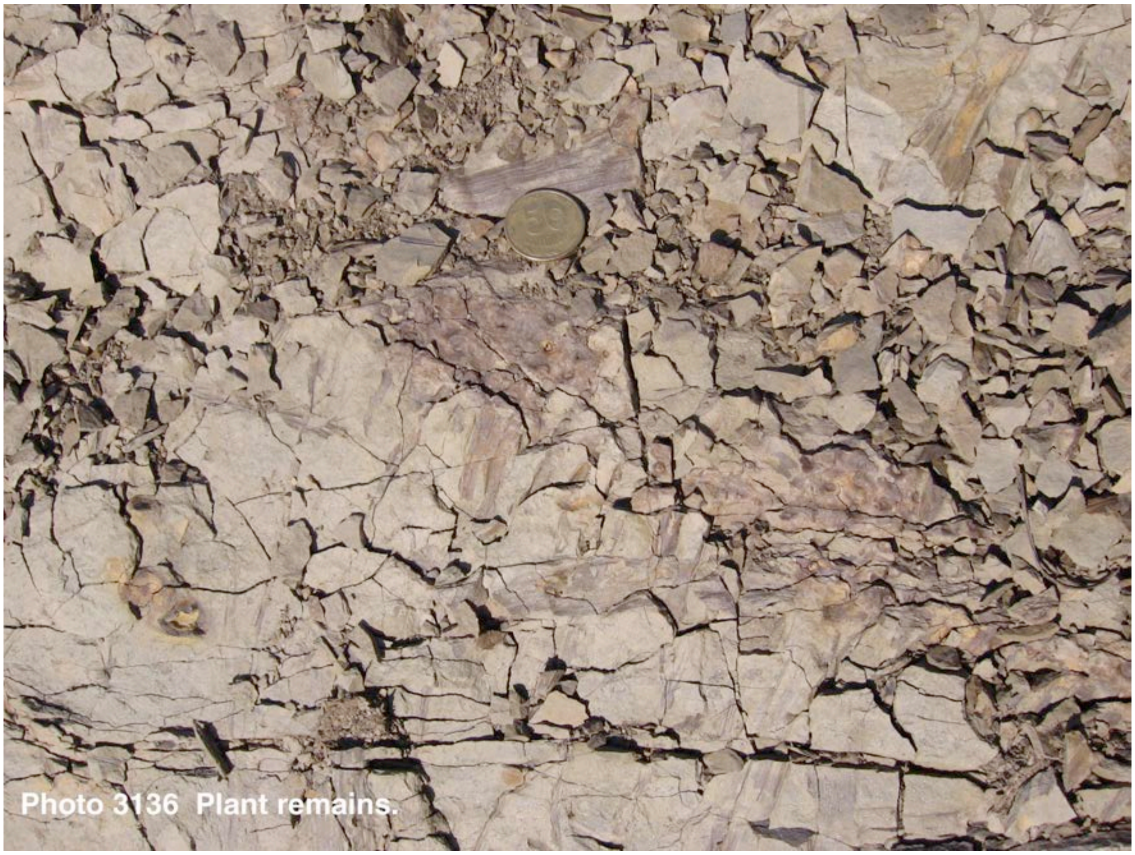

Photo 3136 Plant remains.

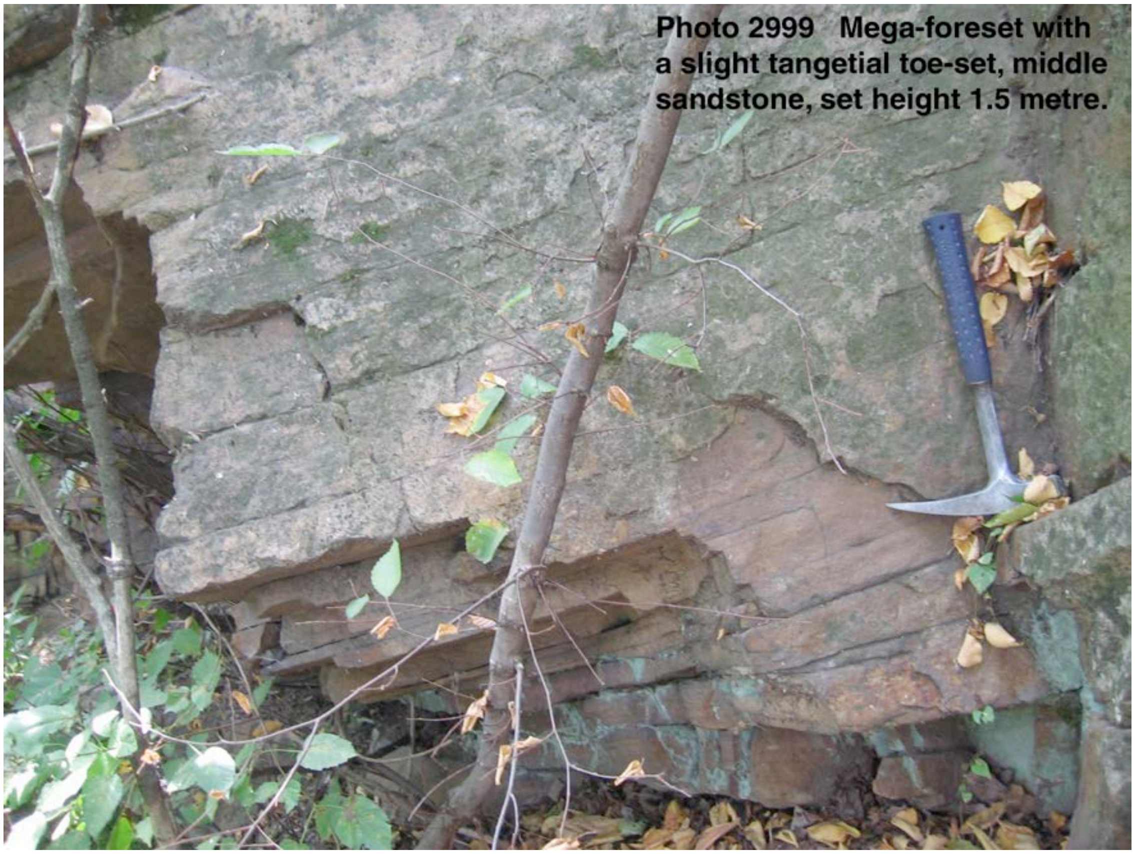

Photo 2999 Mega-foreset with a slight tangetial toe-set, middle sandstone, set height 1.5 metre.

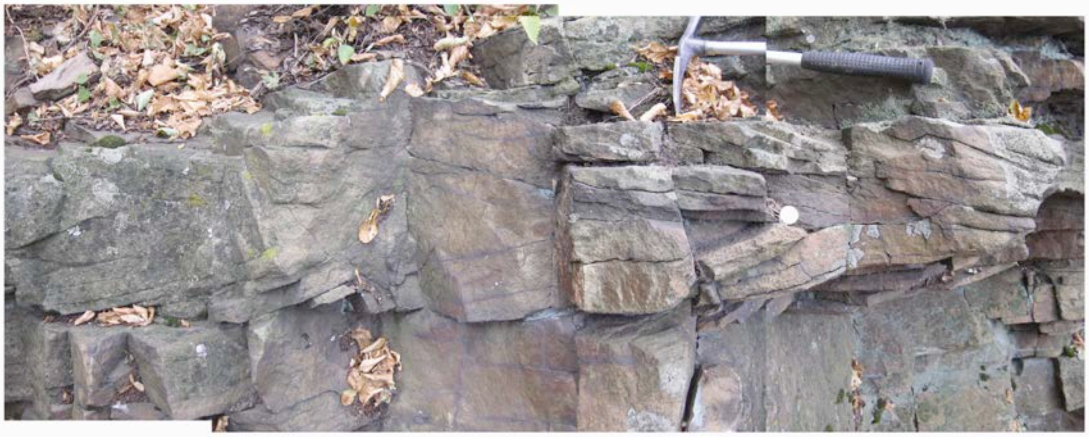

Photo 2996/97/98   Fine to middle sandstone that shows foresets directly on top of each other with paleocurrent directions that are almost opposite.

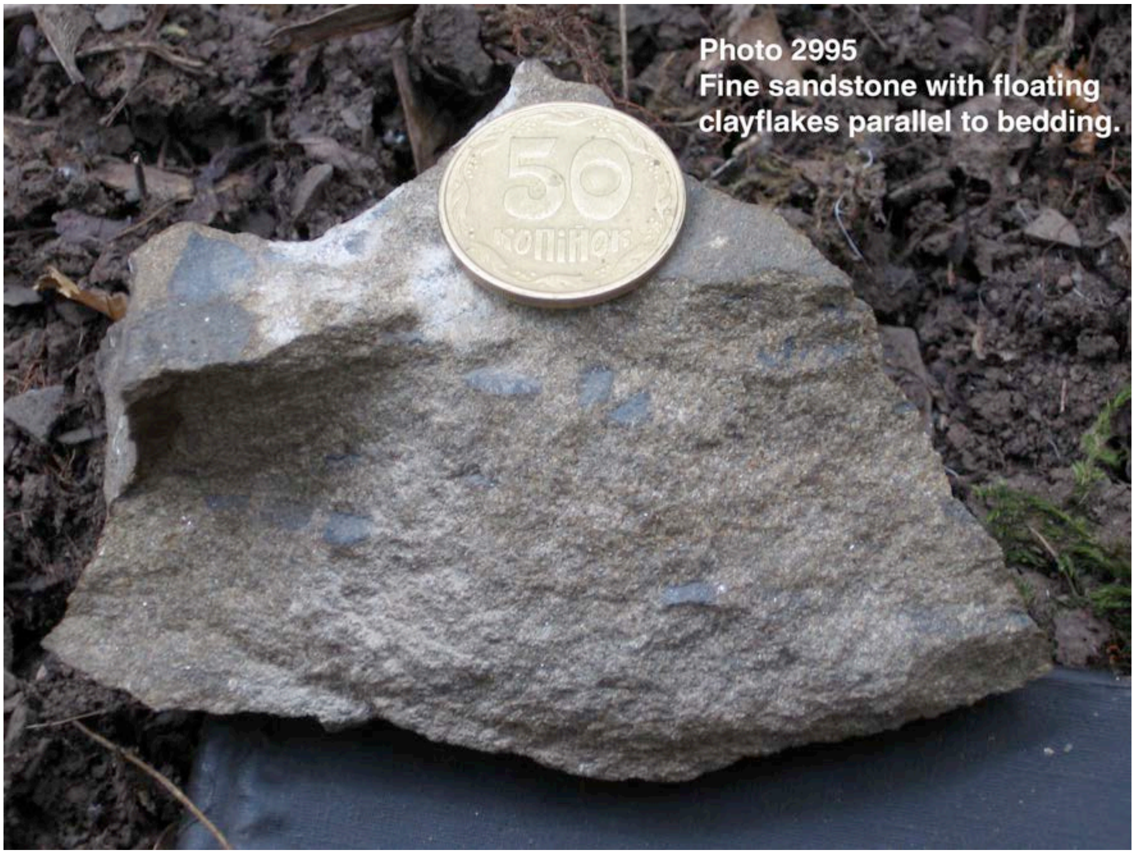

Photo 2995
Fine sandstone with floating clayflakes parallel to bedding.

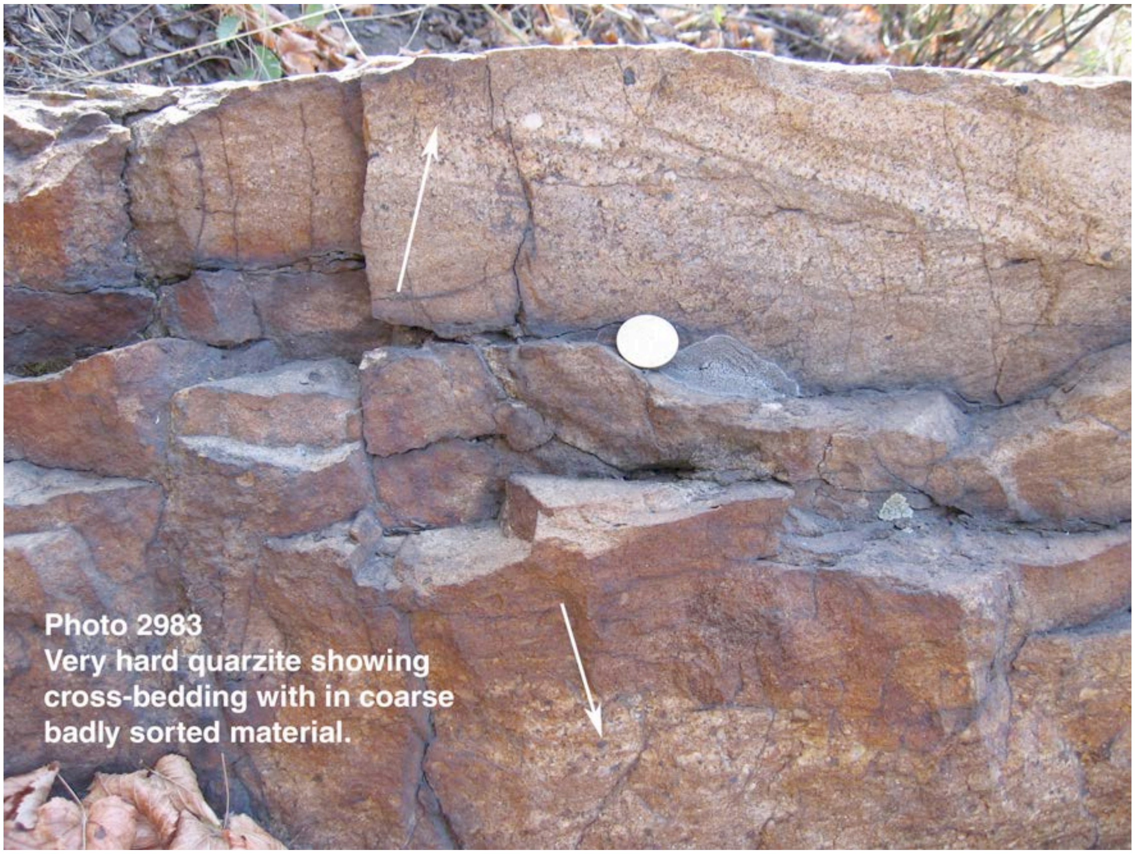

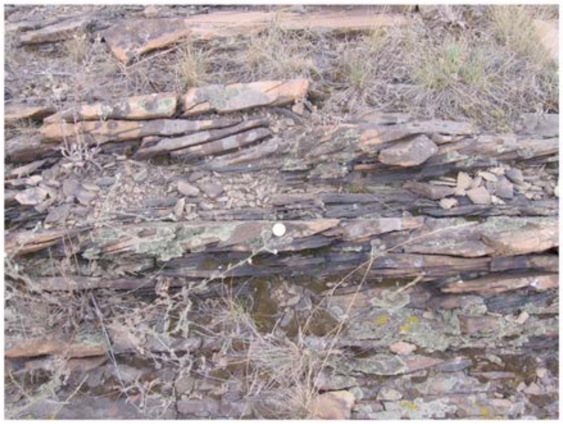
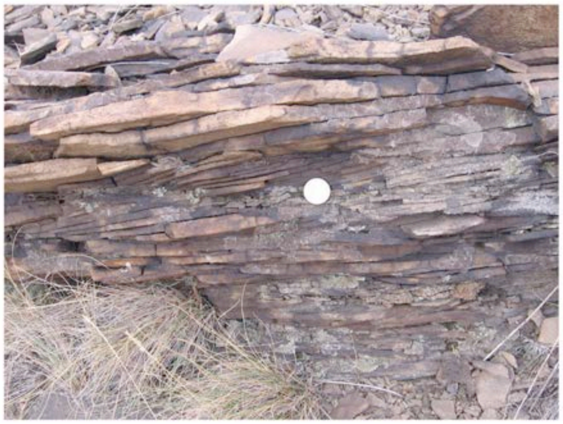

Photos 3000 (left) and 3001 Well-sorted fine sandstone unit ST-D build up of small-scale foresets of height ~10cm, all paleocurrent directions in around same direction, set boundaries ~horizontally, individual foresets clearly separated which may aim at tidal influence.

Section: ***Toshkovka***

Location: 37U 0468392 UTM 5403293, northern area

Situation: The Toshkovka section is named after the village Тошковка (Toshkovka). The section can be reached from the cemetery by walking downwards to the west. The cemetery is situated a few hundreds of metres south of the main road at the eastern edge of the village towards the village called Нижнее (Nizhnee).

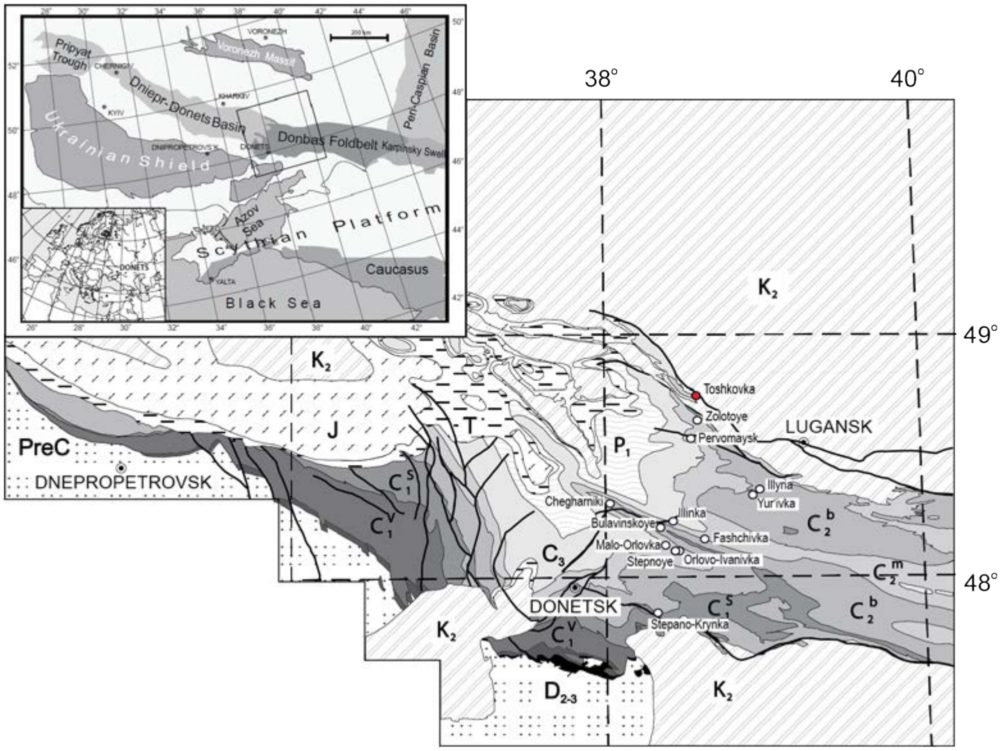

Age: **upper Bashkirian – lower Moscovian, C_2^5**

% Sand: **25 %**

Thickness: **119 m**

Sedimentology:

The lower part of the Toshkovka section is characterised by coarse poorly sorted sometimes massive and tree trunk bearing sandstone units. Some large scale trough cross stratification is present and well graded intervals exist. These units are grouped within sandstone Group A. Above this interval a huge amount of shale exist with a thick limestone in between. Then a few well sorted very fine to fine sandstones occur that are grouped within group D and C. Paleocurrent directions aim at flow to the southeast, although two measurements to other sides considerably bias the rose diagram (Figure XT), as not too many measurements could be taken. One of these two is a paleocurrent measurement to the northwest that has been measured on 3D very small-scale ripples, which might be caused by wave induced currents. The section ends with a thick light

limestone. Two coal layers, of which one has been samples in this project, are present within a shale interval not far below the latter limestone.

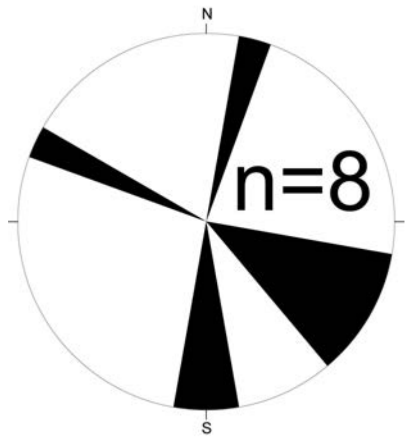

Figure XT. Rose diagram of all measured paleocurrent directions in the Toshkovka section. Petals in groups of 10°, largest petal 1 measurement and 16% of the data.

Environmental interpretation:

The sedimentary characteristics of the Toshkovka section show a deepening from fluvial (Group A) to shallow marine environments (Group C and D). The uppermost thick limestone has to be deposited in clear water environments without clay suspension, while the close co-occurrence with coal layers possibly requires fast short term sea level variability. The fact that around these layers no shallow sandstones have been deposited is not fully understood.

MACROSCOPIC DESCRIPTION of TOSHKOVKA section

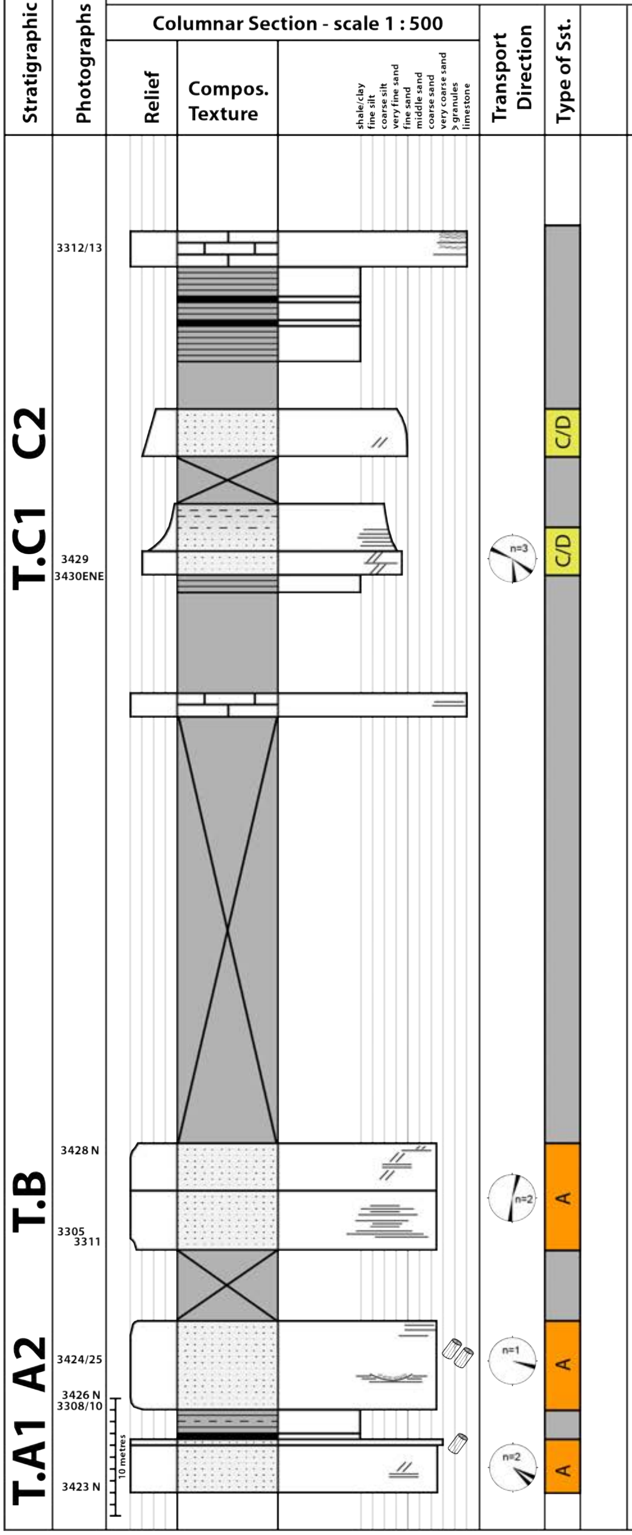

Additional DESCRIPTION and remarks

Top of Section
End of outcrop is this area.

LIMESTONE: 2.70 metre thick lst with a nodular character increasing to the top, foram-rich light yellow packe- to wackestone (on outcrop scale). BP ~132/12 NE

Two thin coal seems, sample taken

Light, well-sorted, foresetted, fine to very-fine FU sandstone unit, foresets, small outcrop so no direction caught, Qtz, feldspar (oxidised), blakc mineral (hornblende?), porous, soft material

Light yellow very fine to fine well sorted current induced cross-bedded sandstone unit, foreset height ~20 cm, foreset laminae thin, planar bedded unit, bit variation in direction (apparant on outcrop-scale), some silty intercalations, 3-D ripples with flow completely opposite, BP 121/23 N

LIMESTONE; dark limestone, 170 cm thick, mud- to wackestone, crinoids and possibly charophytes or forams.

Non-outcrop --> shales?

Poorly sorted, well-graded to moderately graded very coarse sandstone, foresets in mainly measured direction (019/40 E), some more to W

Poorly sorted, well-graded to moderately graded massive sandstone unit, full of upper plane bed that FU from very coarse to middle sst, BUT; there is the possibility that some of the plane beds flow rock in or out, no evidence found for this, BP 112/30 N

Massive tree-trunk rich middle to coarse sandstone unit, moderately sorted, seems to be porous, feldspar not weathered, light yellowish in colour, Qtz, feldspar, in lower part gully-like truncation, some foresets, mainly chaotic, not a quartzite (!) --> fluvial, a bit braided? unit also disturbed by fault movement

Coal seem disturbed by fault
Poorly sorted, bad- to moderately graded coarse sandstone unit with grains from middle sst to granules size, current induced cross-bedded, upper planar beds, and massive homogenous intervals, BP 105/34 N

Base of Section close to cemetery, end of outcrop

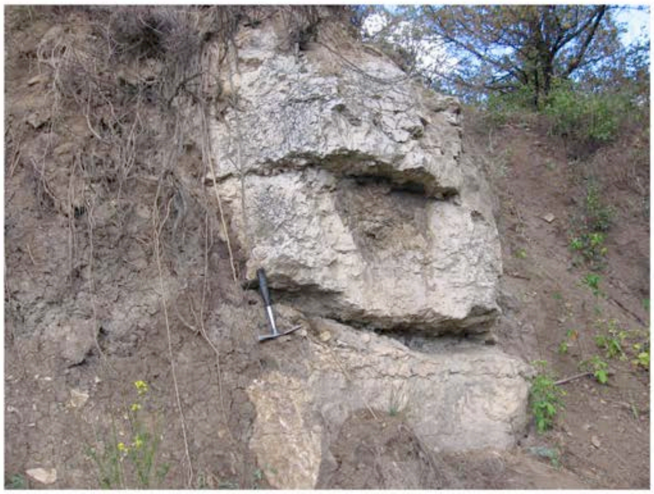

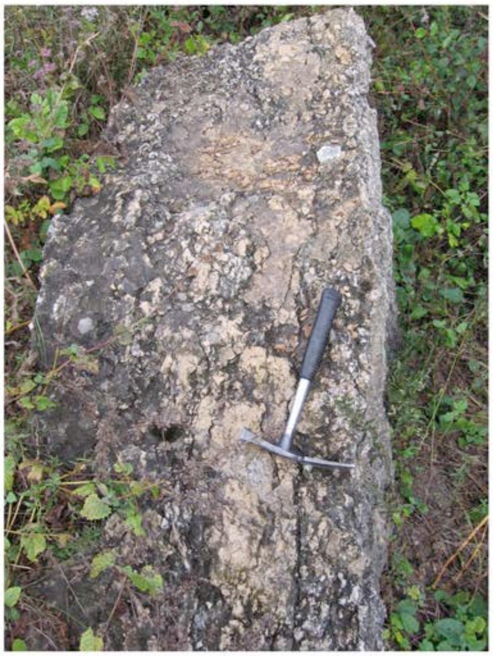

Photos 3312 (right) and 3313
Thick limestone bed divided into three beds showing increasing nodular character to top, at right top view of uppermost bed.

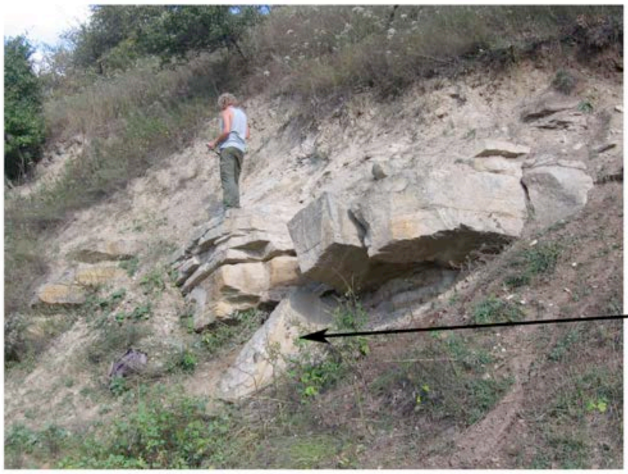

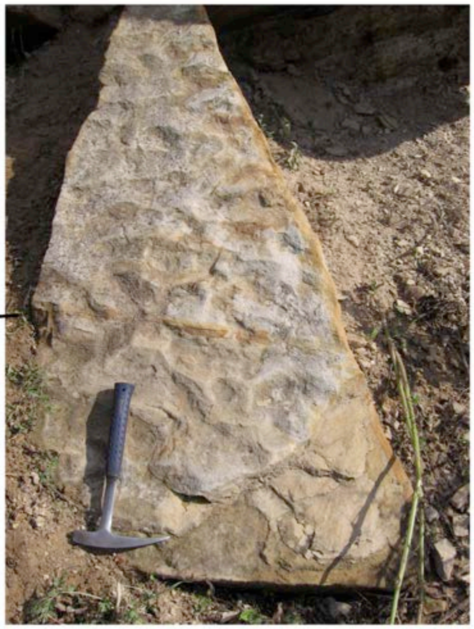

Photos 3329 (right) and 3330
Very fine to fine well-sorted sandstone showing cross-beds and (at right) small-scale 3D-ripples with a flow component opposite to other paleo-current directions.

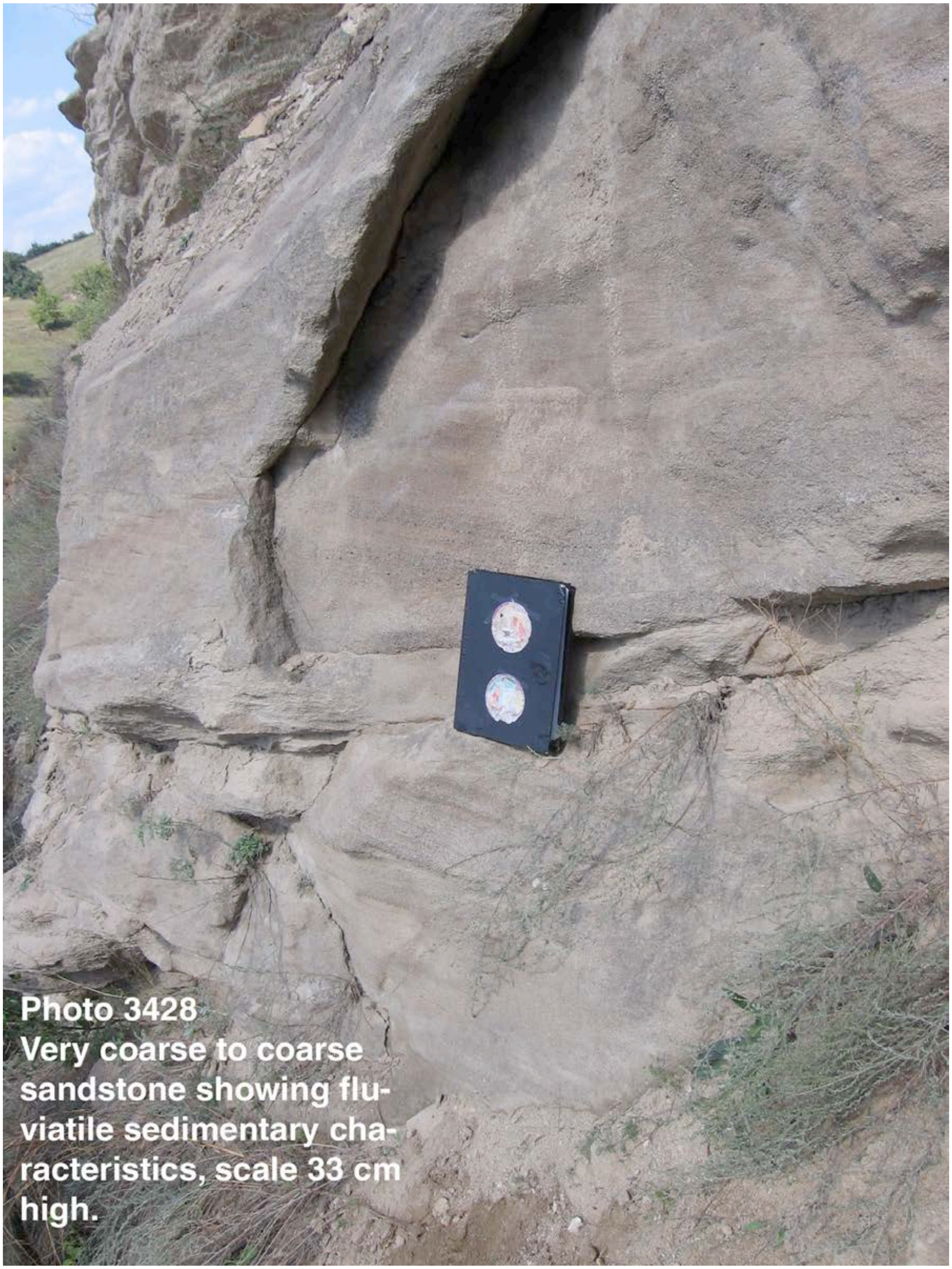

Photo 3428
Very coarse to coarse sandstone showing fluviatile sedimentary characteristics, scale 33 cm high.

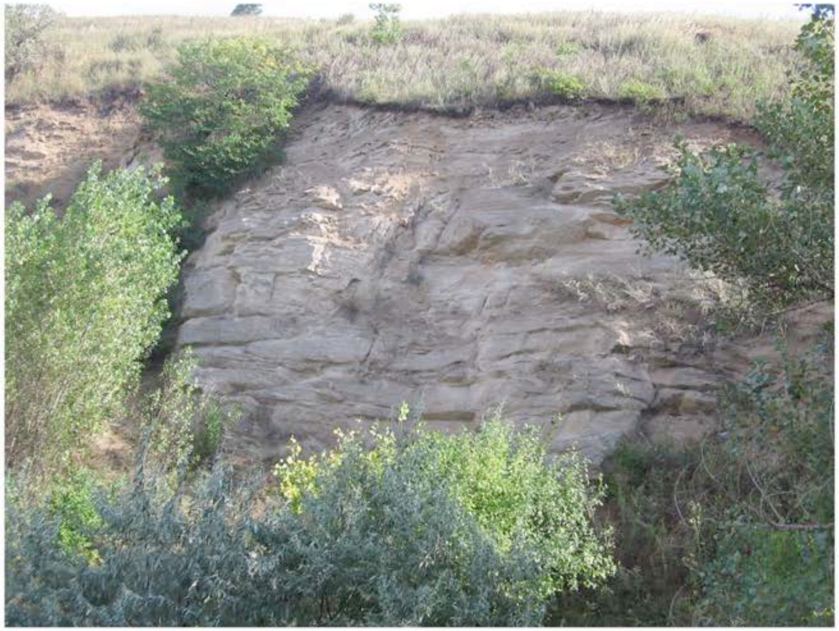

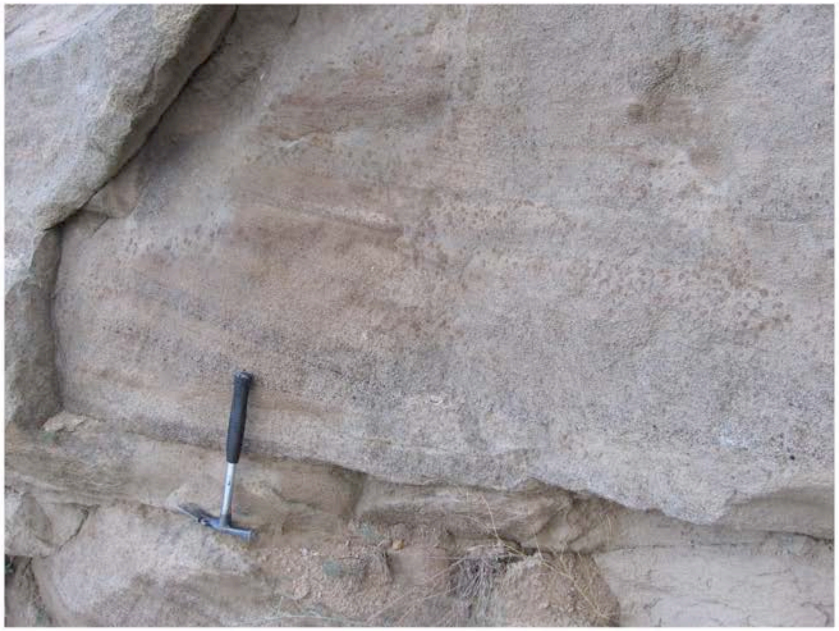

Photos 3305 (left) and 3311
View on sandstone unit TB and at right detail of nicely grading indivual foresets from granule-size to coarse sandstone, toe-set fairly tangential.

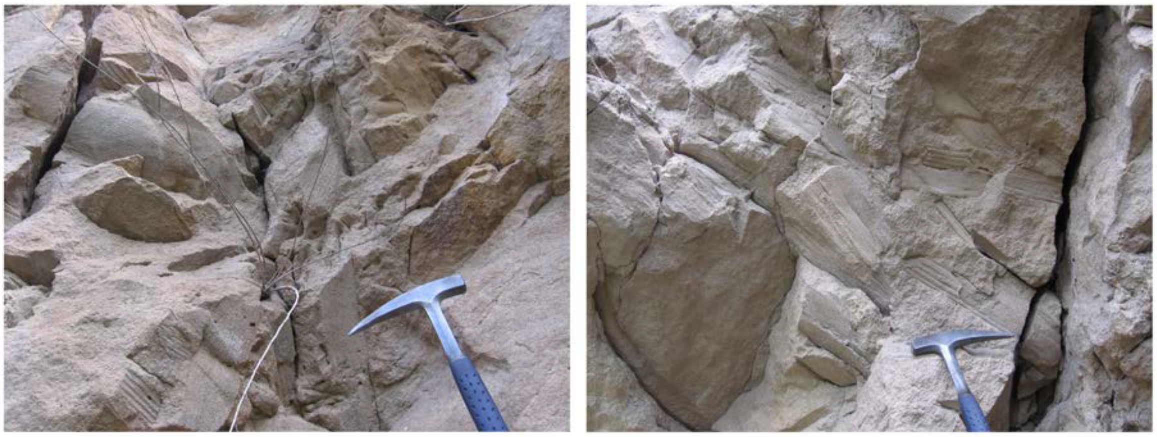

Photos 3324 (left) and 3325   Tree trunk prints.

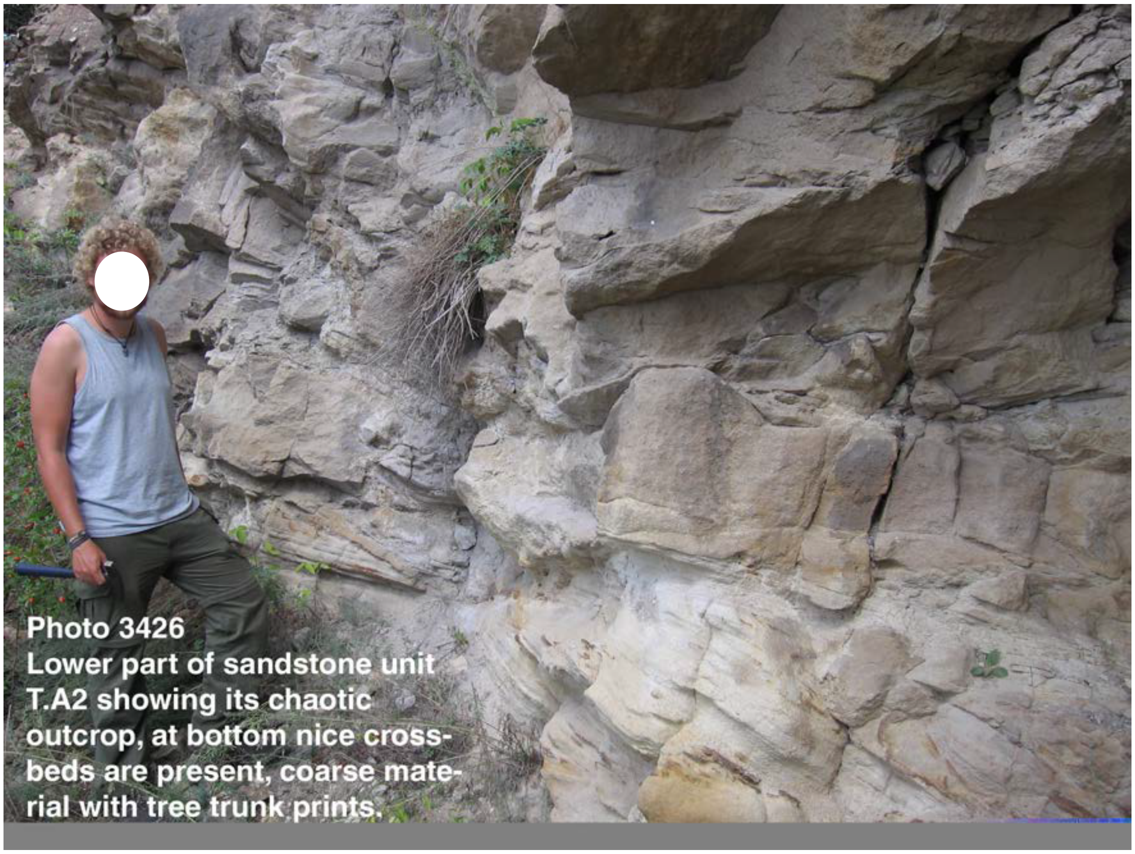

Photo 3426
Lower part of sandstone unit T.A2 showing its chaotic outcrop, at bottom nice cross-beds are present, coarse material with tree trunk prints.

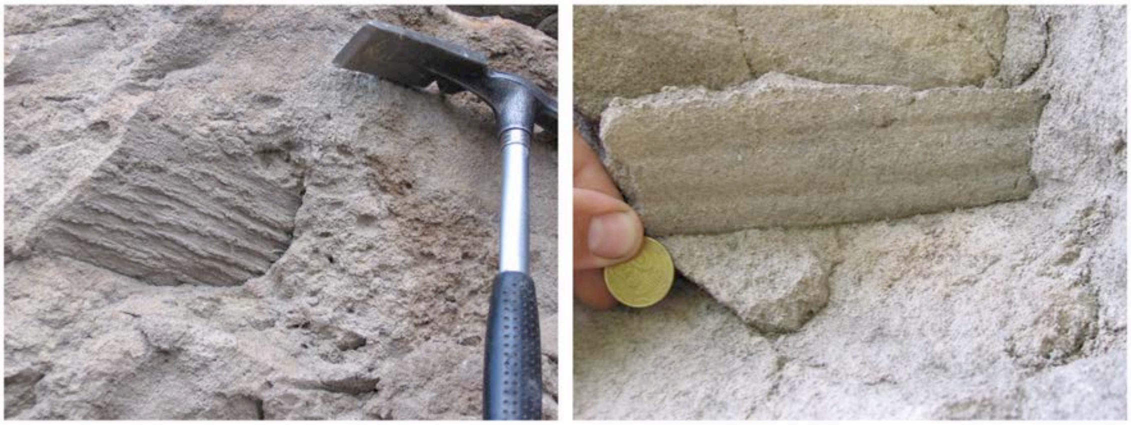

Photos 3308 (left) and 3310   Details of tree trunk prints.

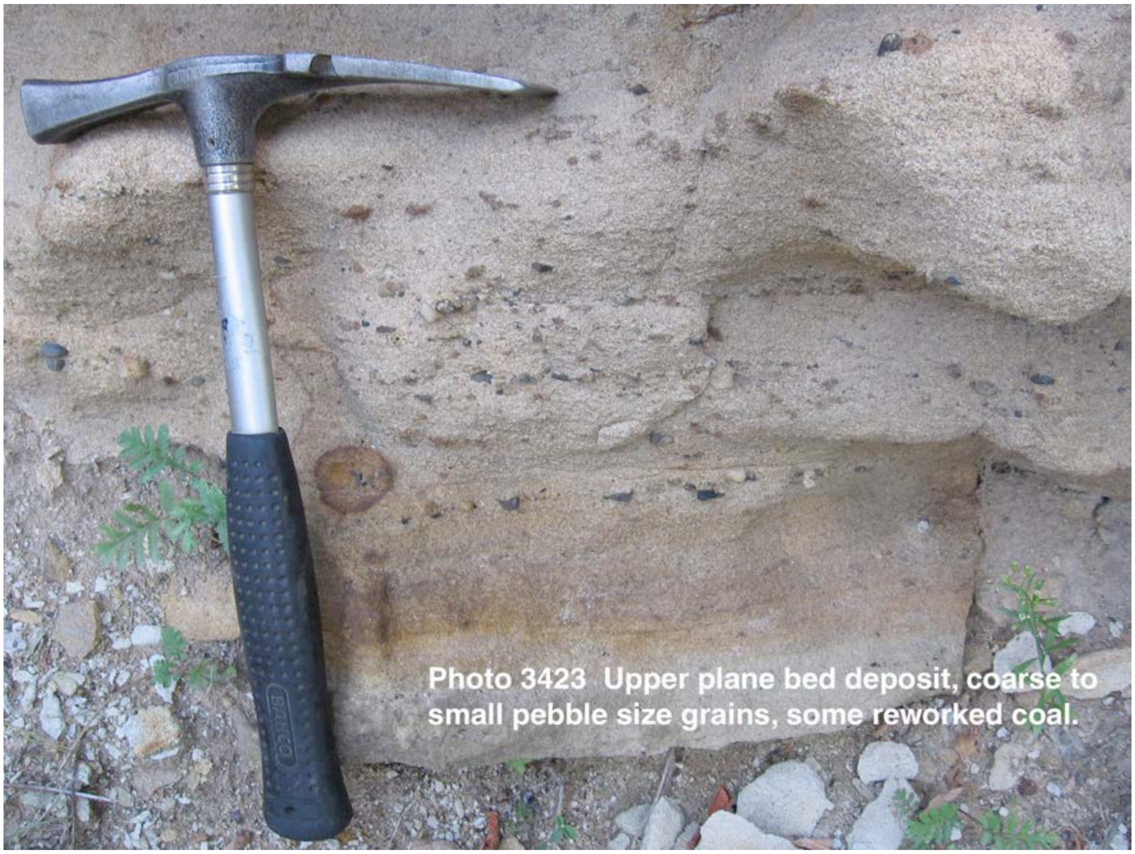

Photo 3423 Upper plane bed deposit, coarse to small pebble size grains, some reworked coal.

Section: ***Yur'ivka***

Location: 37U 0496636 UTM 5359154, northern area

Situation: Yur'ivka section is named after the village Малая Юрьевка (Malaia Yur'ivka). The section is situated at the right side of the paved road just before the village when approaching the village from the northeast (from Illiria village).

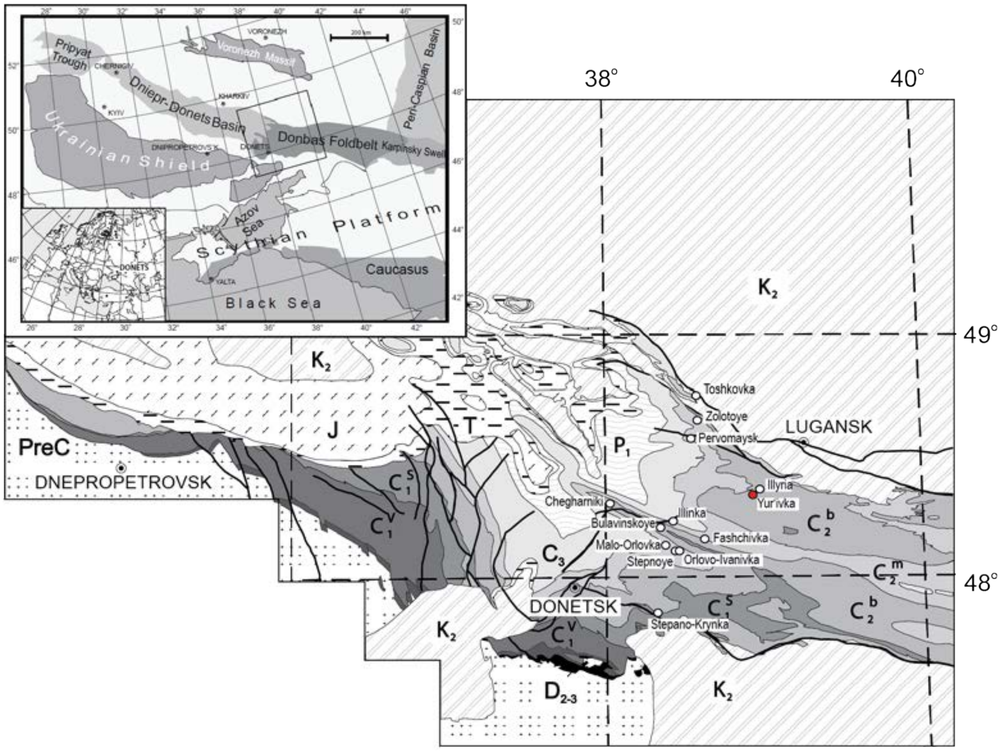

Age: **upper Bashkirian, C_2^3** and **C_2^4**, limestone J_1 and J_4 are close to the base and top of the section, respectively.

% Sand: **35 %**

Thickness: **123 m**

Sedimentology:

The sandstones from the Yur'ivka section mainly show good sorting, plane bed lamination, fine to middle grains, and hummocky cross stratification. These characteristics can best be grouped in sandstone Group C. The uppermost sandstone shows some characteristics of Group B with large scale current induced cross bedding. Thick limestones are present in the shale intervals. Below the section more sandstones with Group C and D characteristics are present and huge shale intervals.

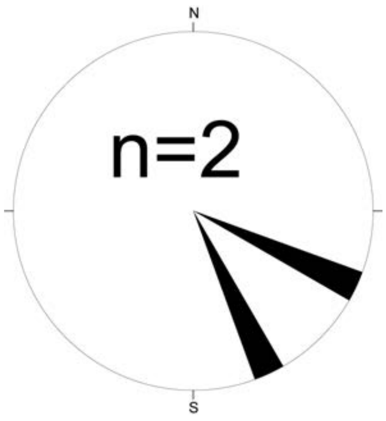

Figure XUI. Rose diagram of the two measured paleocurrent directions in the Yur'ivka section. Petals in groups of 10°.

Environmental interpretation:

The Yur'ivka section reveals sandstones of middle to lower shoreface depositional environments. No fluvial impulses have been found which strengthens this interpretation. The shale-rich interval below the section might be seen as a 3rd order sea level high-stand and the section itself a 3rd order sea level low-stand. This section shows that low-stands of the 3rd order sea level cycles do not involve fluvial impulses to be deposited, and thus that they are relatively high with respect to low-stands at the base and top of the middle Carboniferous.

MACROSCOPIC DESCRIPTION of YUR'IVKA section

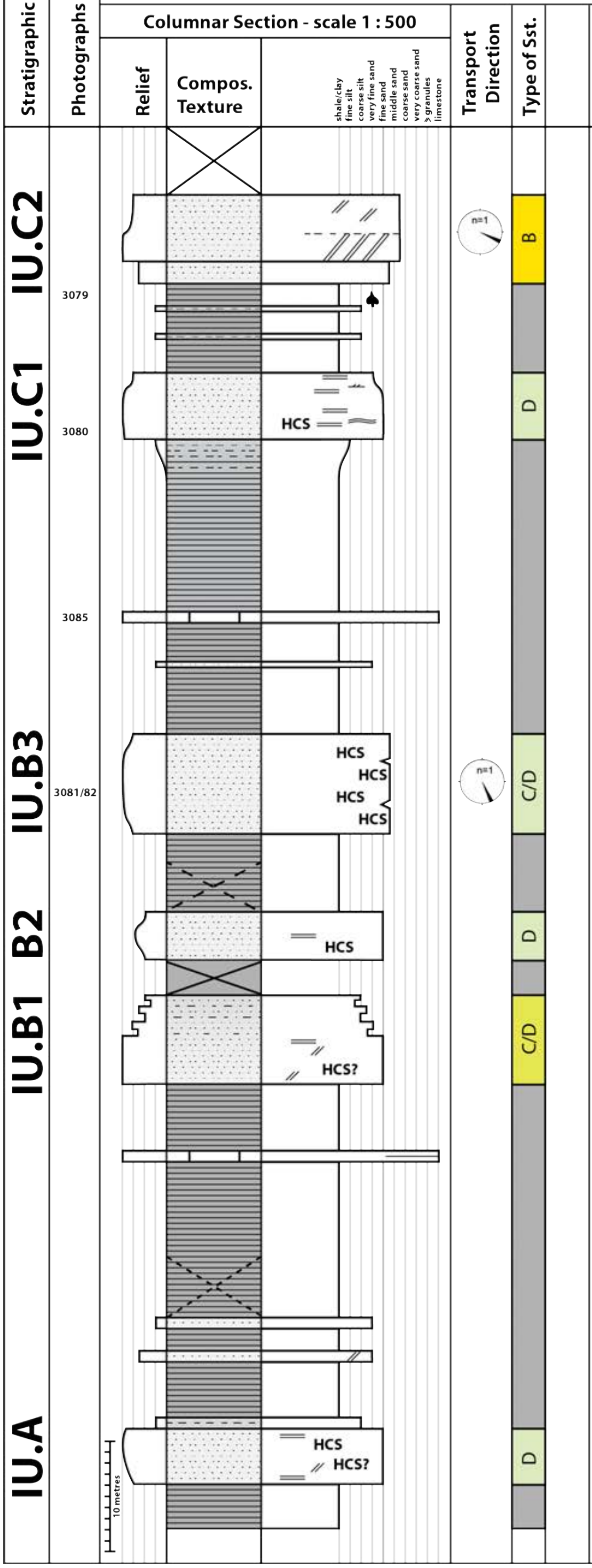

Additional DESCRIPTION and remarks

* Top of Section, also end of outcrop --> shales? *

Middle to coarse sandstone unit, full of mega-foresets up to 2 m, unit has weathered outcrop; structures hard to see, or only foresets present. Possibly few indications for some big waves (HCS?), no clear cut offs found, unit fairly continuous. Qtz, feldspar, some black minerals, angular clasts, some low spherical, moderate sorting. Bedding Plane (BP) orientation ~106/61 S (in shales)

Shale interval with two coarse silt beds, no structures, plant remains are small twigs?

Horizontally laminated fine sandstone unit with only one layer of small-scale cross-beds. In softer part some wavy lamination, with small wave ripples of thickness 1 cm. Horizontal laminae are lower plane bed deposits. Qtz, black minerals, few feldspar and mica. At bottom interval big swales (photo).

Shale interval, no plant remains seen

Limestone: 75 cm thick black mudstone, no fossils on outcrop scale (handlense 10X), few orange oxidised breccia-like structures, BP 090/51 S

Very thin and fine sandstone bed, no structures recognised, within shale interval

Fine to fine-middle sandstone unit full of or even built up of hummocky cross stratification or maybe swales, probably some parts mega-foresets or at least a dominant direction in HCS. Qtz, black minerals, feldspar, and little mica, angular spherical grains, lower part seems to be not continuous, but this can be due to outcrop, in good outcropping part, the sst-unit seems to fairly continuous (>50 m)

Shale interval without plant remains (not found) and bad outcrop in lower part

Fine sandstone unit with a bad outcrop, possible HCS, horizontal lamination, laminae thickness 1 à 2 cm's, lots of mica

Greenish fine sandstone unit with FU to top with increasing intercalation of silt and shales. In top of lower part horizontal and very low angle lamination, in bottom part mega-foresets up to 50 cm with different cut-off plane orientations (due to wave action?) HCS not seen in field, but looks like current and storm-wave interaction.

Limestone: black mudstone with few crinoids in top half, lower half lacks bioclasts BP 090/50 SW

Fine sandstone unit with HCS in top and cross-beds that are possibly HCS in rest of unit, some apparent horizontal lamination. Minerals hard to see, but qtz, feldspar, black minerals, and quite some mica.

End of Section Stratigraphically lower a thin limestone is found and a few thin fine sandstones within a mainly shaley interval.

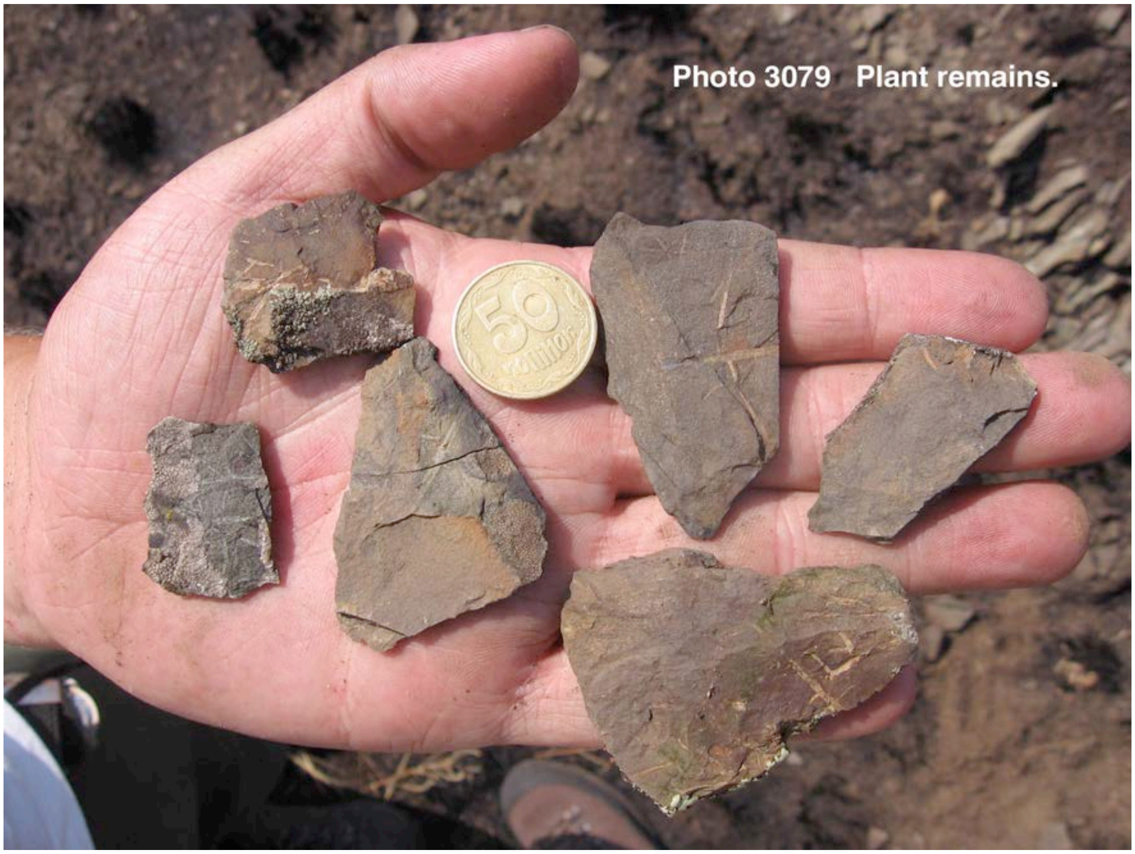

Photo 3079 Plant remains.

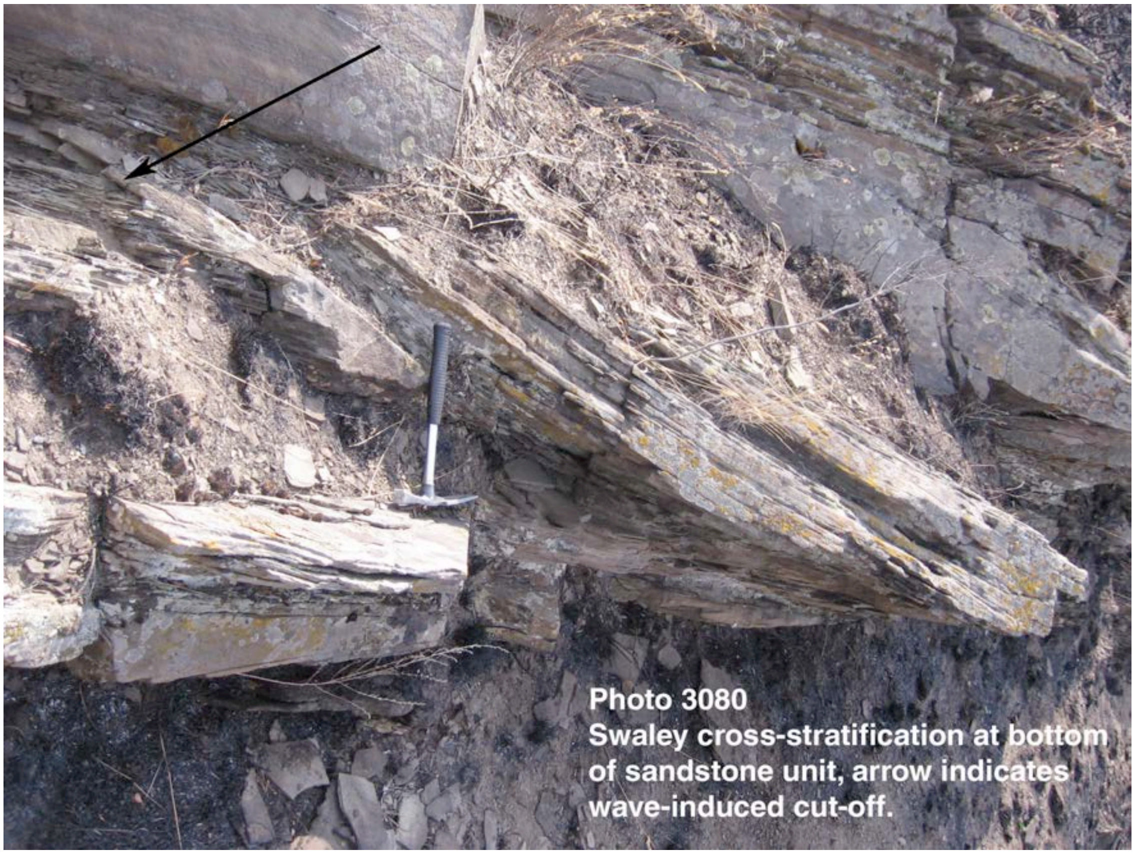

Photo 3080
Swaley cross-stratification at bottom of sandstone unit, arrow indicates wave-induced cut-off.

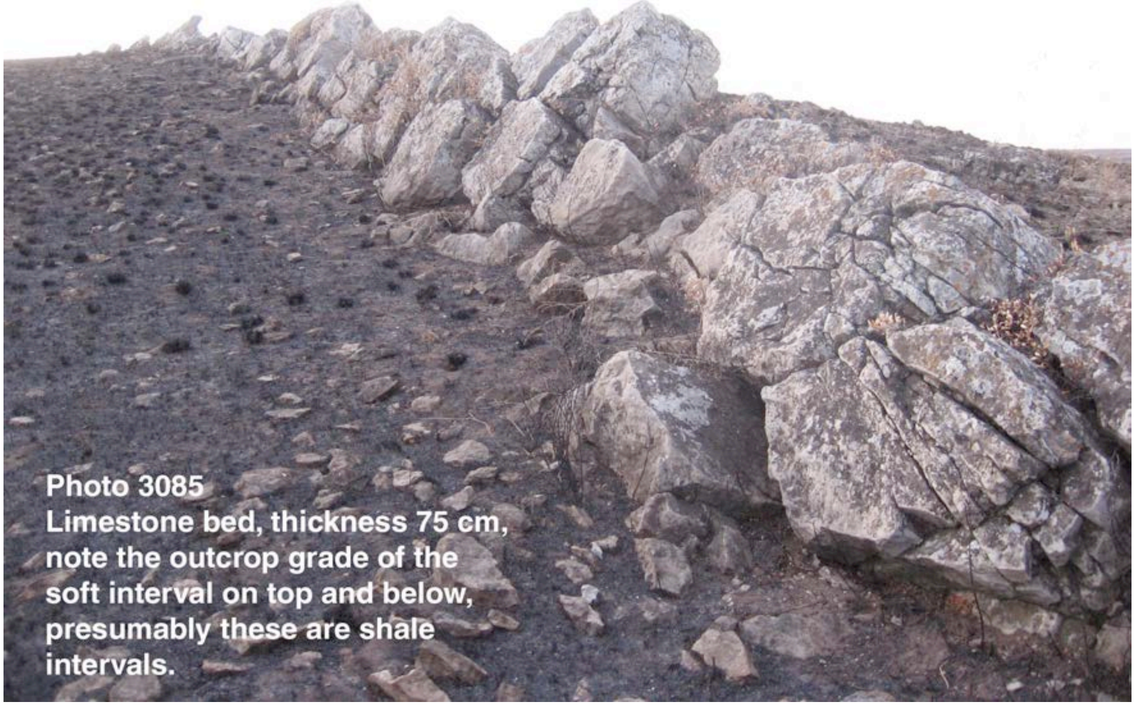

Photo 3085
Limestone bed, thickness 75 cm, note the outcrop grade of the soft interval on top and below, presumably these are shale intervals.

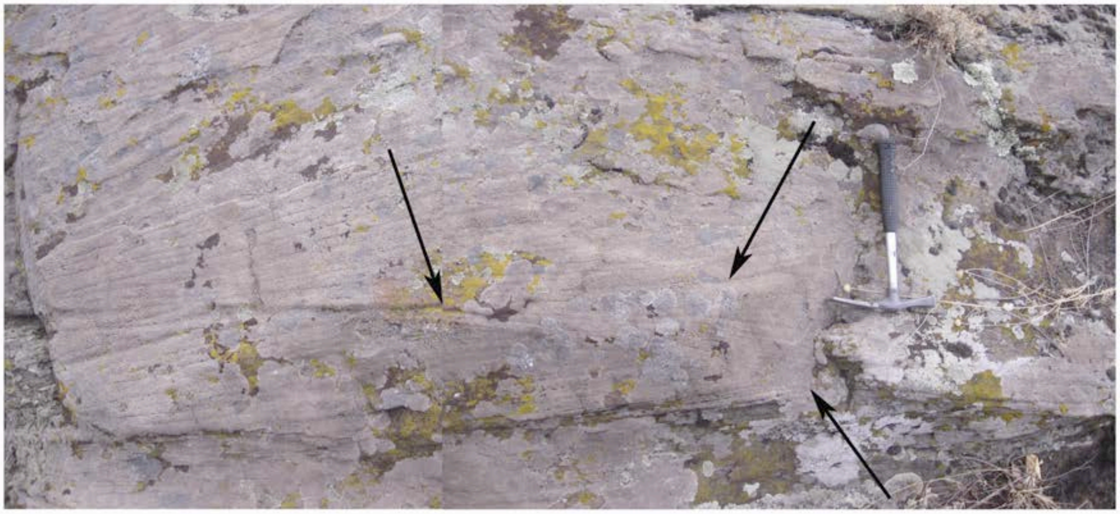

Photo 3081/82 Swaley cross-stratification in an intermediate quality outcrop, fine-middle well-sorted sandstone, hammer 32 cm.

Section: ***Zolotoye sandstone***

Location: 017D, northern area

Situation: The Zolotoye sandstone is named after villages Эолотое (Zolotoye), of which there are several around the section. The sandstone is situated in a small non-official quarry at the northside of the Камыщеваха river at the junction in the road between two Эолотое-villages and Голубовское (Golybovskoie).

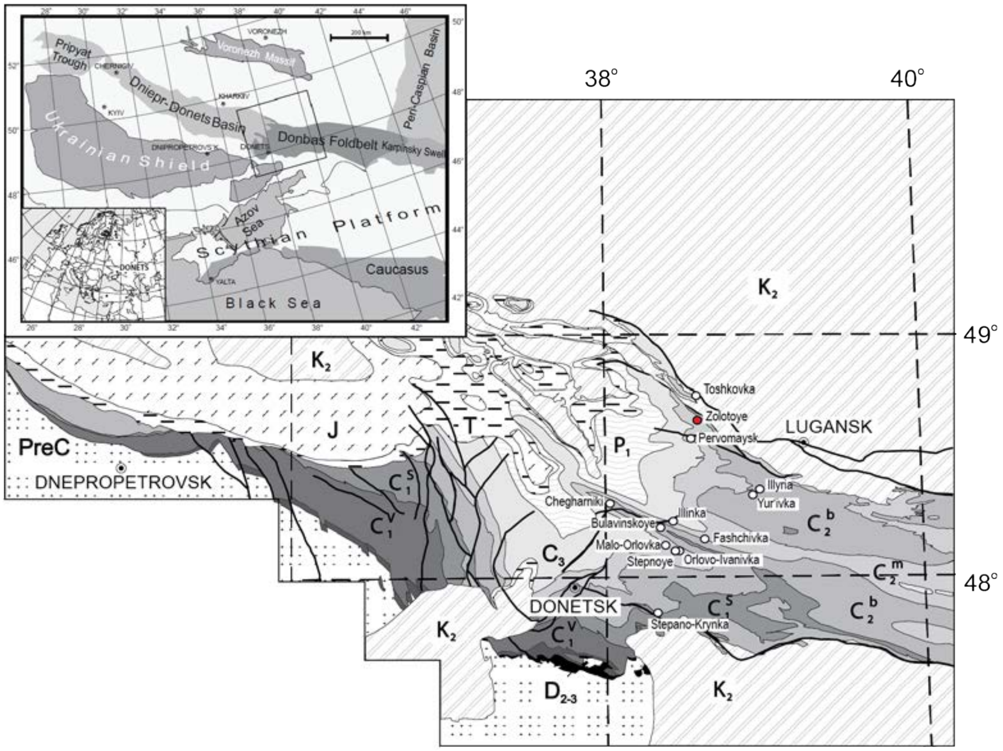

Age: **lower Moscovian, C_2^5**

% Sand: **n/a**

Thickness: **12 m**

Sedimentology:

The Zolotoye sandstone can be divided into two halves that show distinct sedimentary characteristics. The lower part shows poorly sorted bedded massive coarse sandstone, and can be grouped into Group A. The upper half consists of better sorted coarse sand with large-scale current induced cross bedding. Without success there has been searched for stratigraphy around the Zolotoye sandstone.

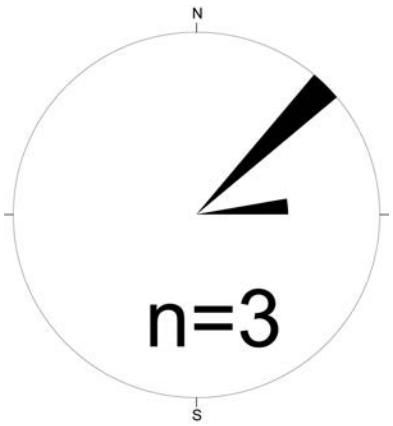

Figure XT. Rose diagram of the three measured paleocurrent directions in the Zolotoye section. Petals in groups of 10°.

Environmental interpretation:

The lower part of the Zolotoye sandstone is interpreted to be deposited in a fluvial environment. The upper part has characteristics of deltaic and upper shoreface environments (Group B).

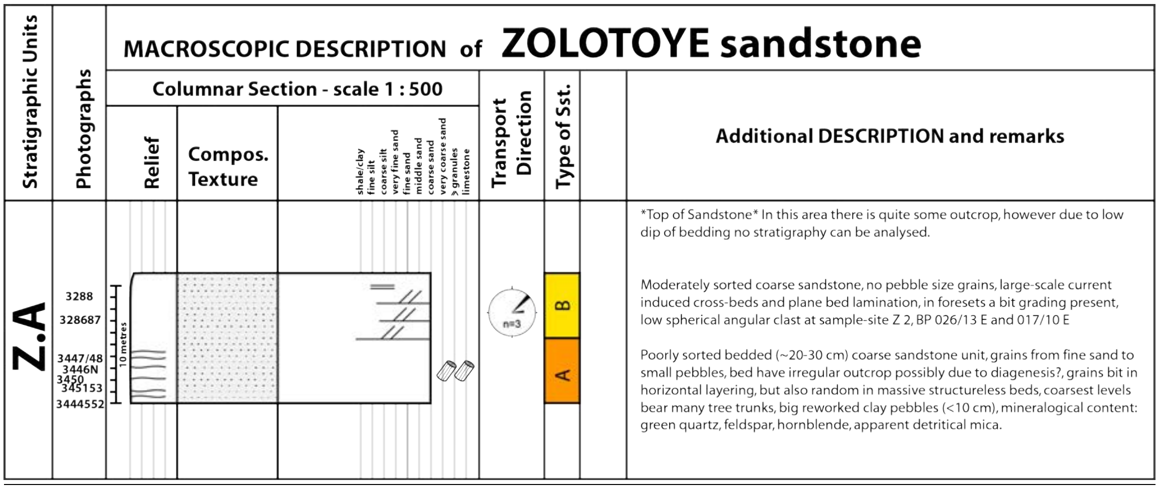

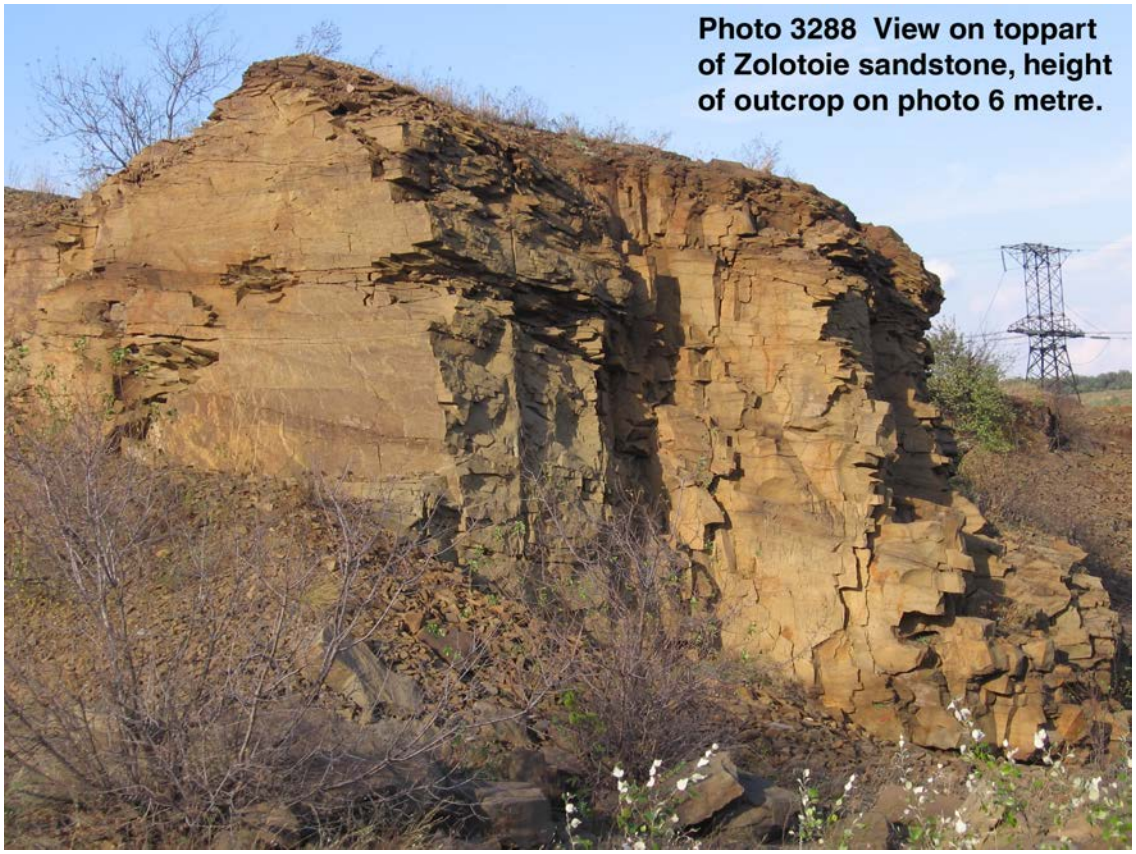

Photo 3288 View on toppart of Zolotoie sandstone, height of outcrop on photo 6 metre.

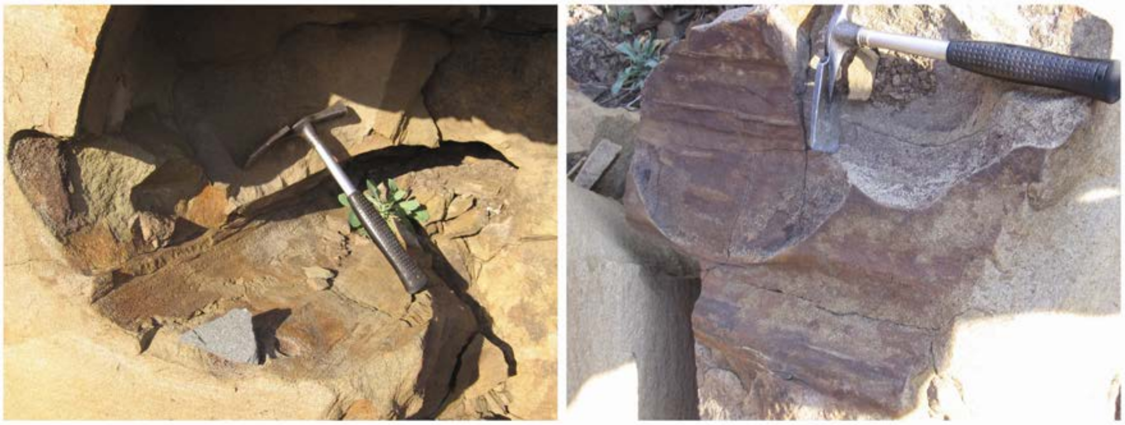

Photos 3286 (left) and 3287 Tree trunk prints.

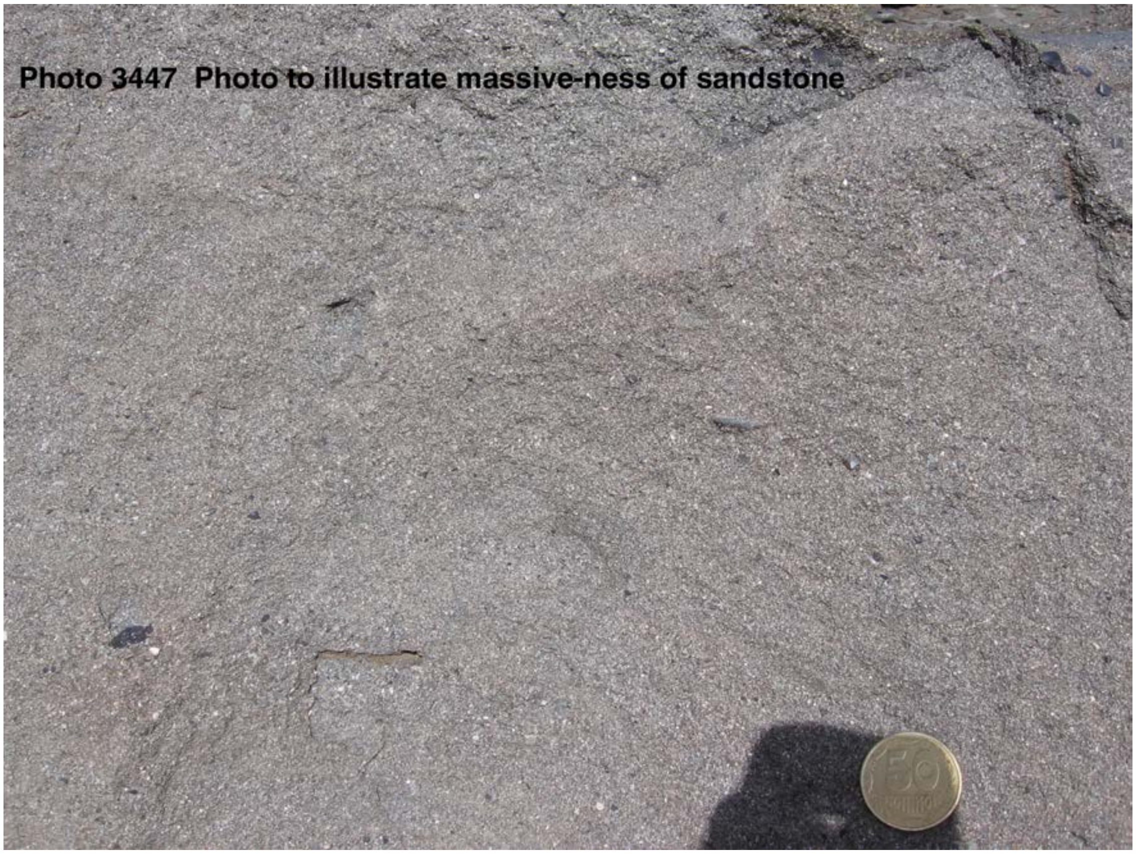
Photo 3447 Photo to illustrate massive-ness of sandstone

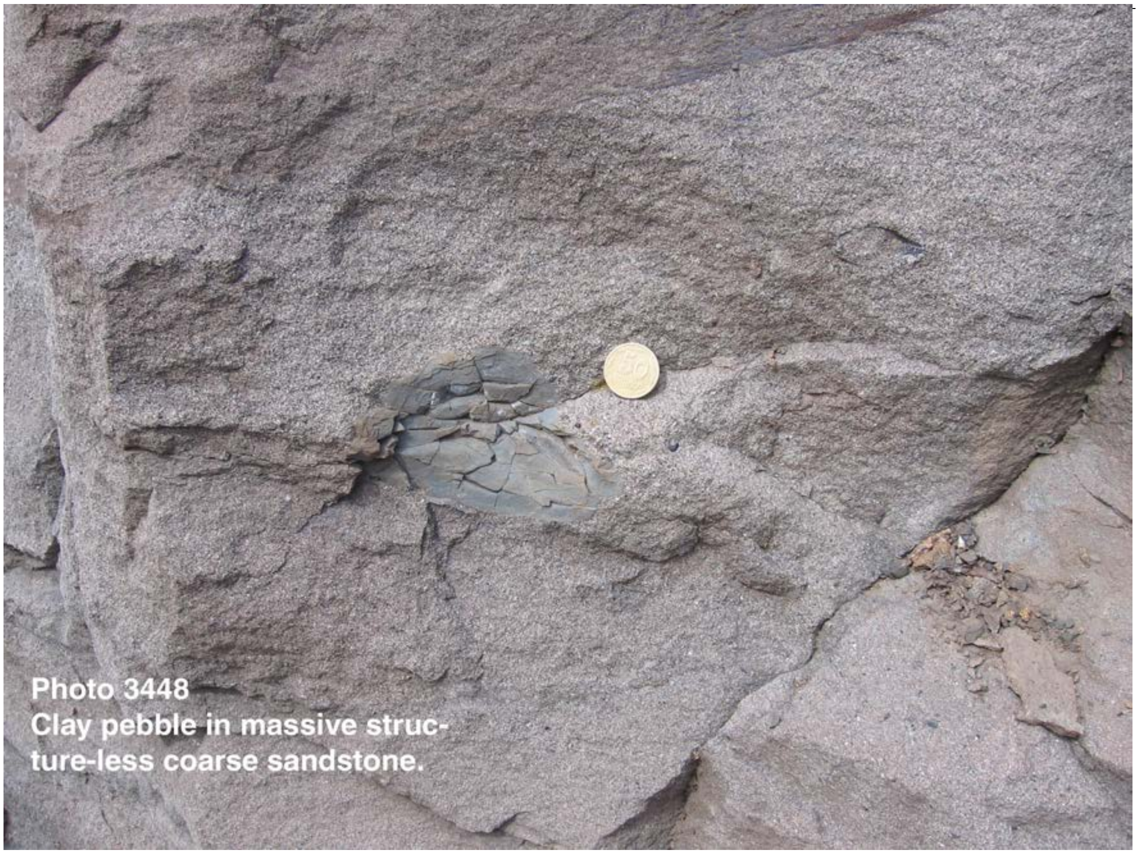
Photo 3448
Clay pebble in massive structure-less coarse sandstone.

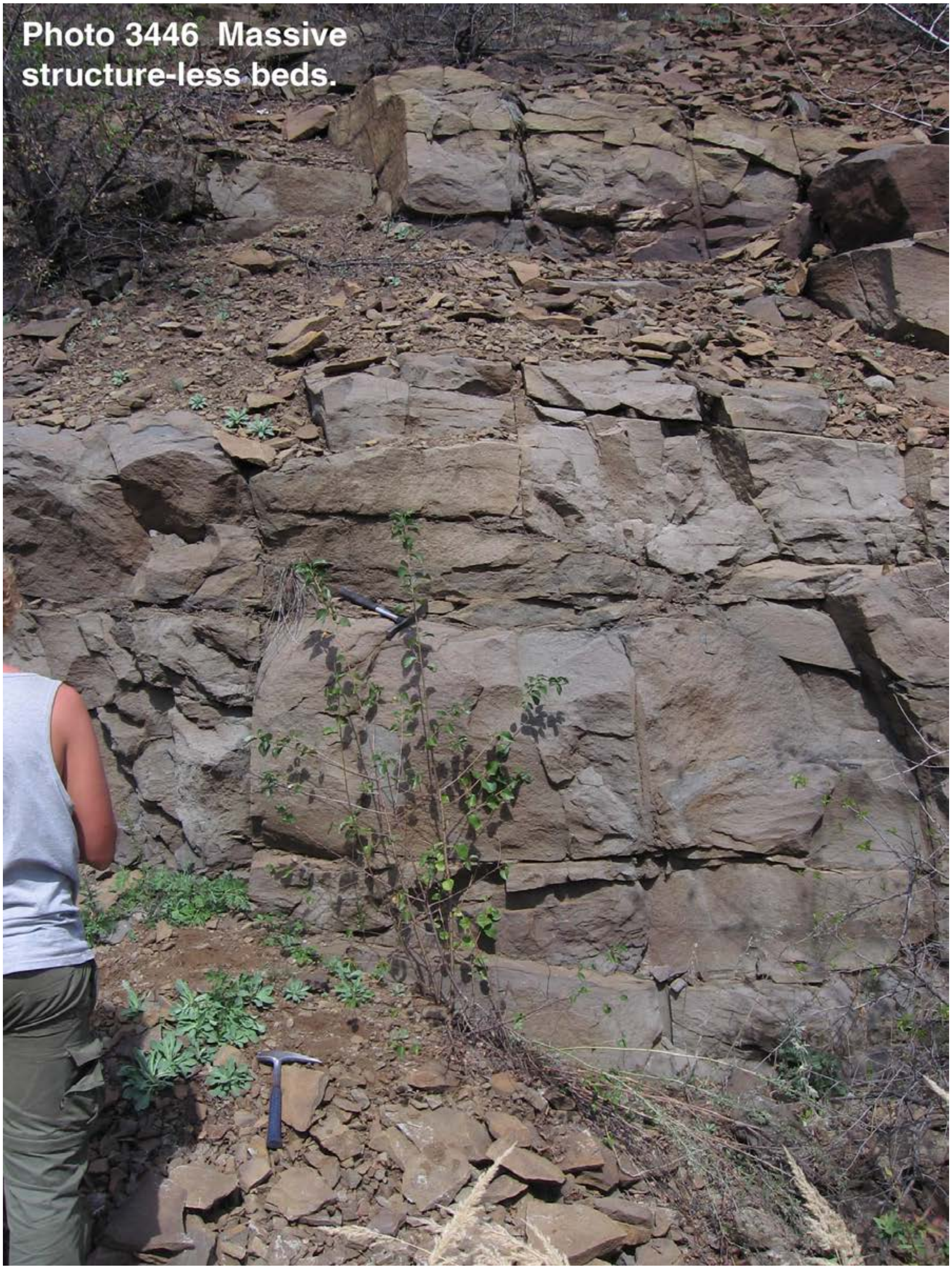

Photo 3446 Massive structure-less beds.

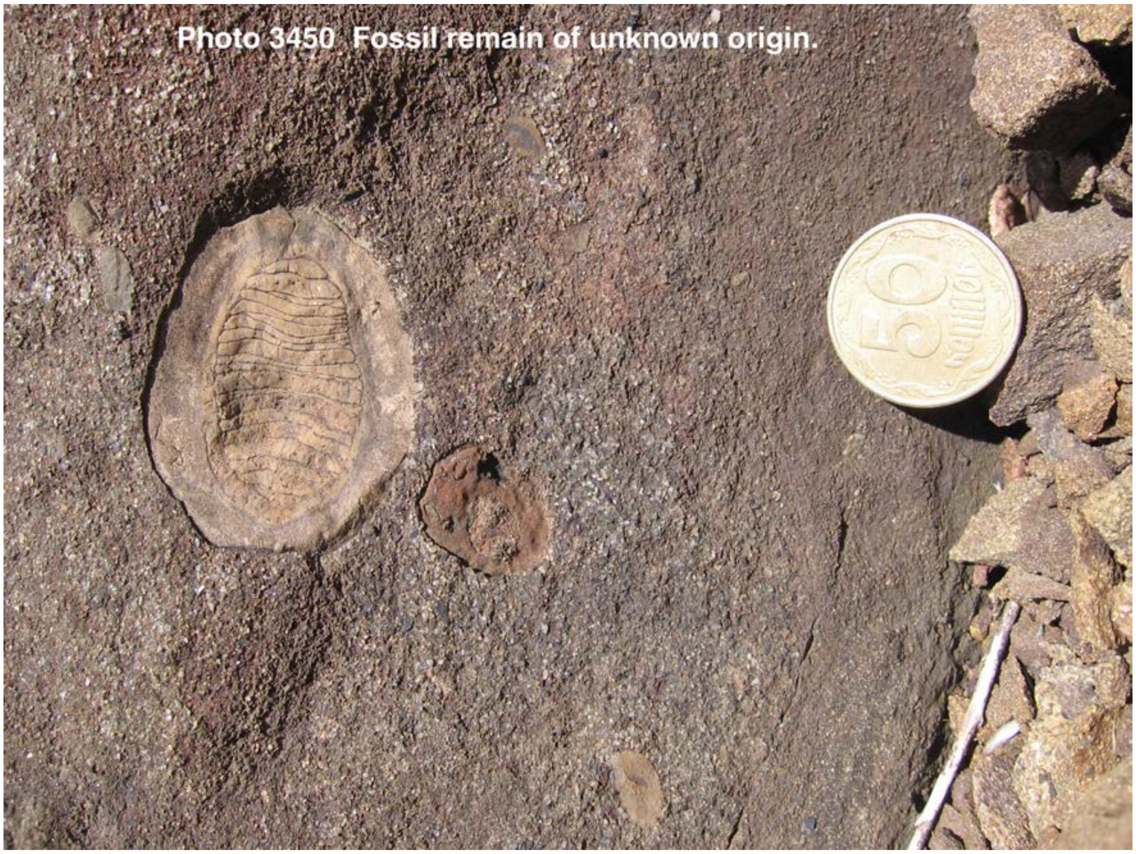

Photo 3450 Fossil remain of unknown origin.

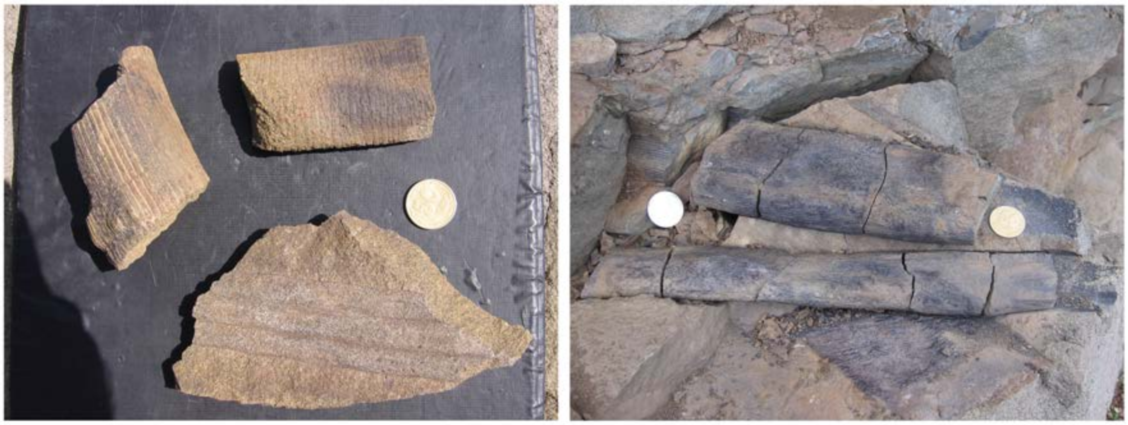

Photos 3451 and 3453 Tree trunks in coarse sandstone.

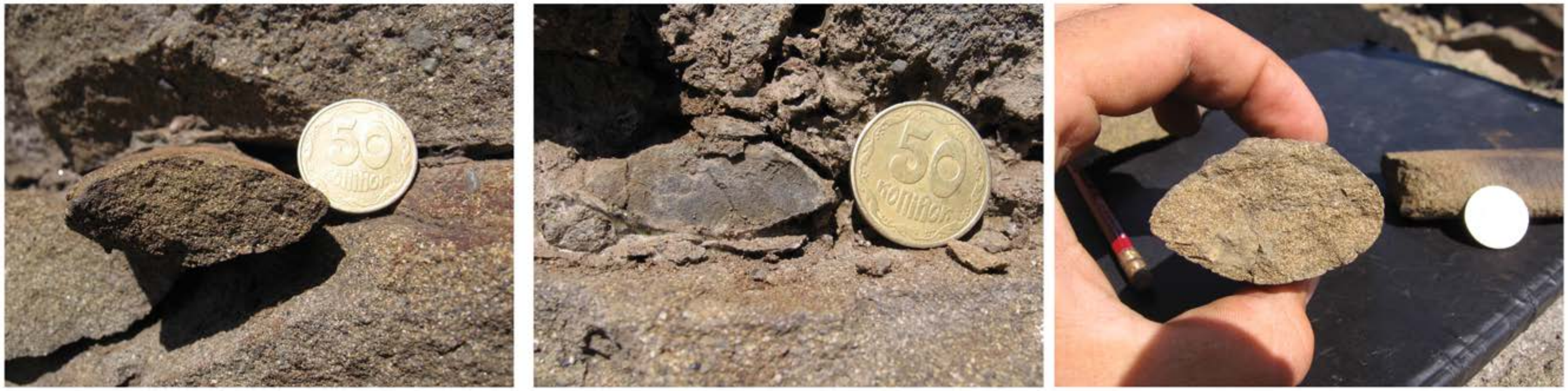

Photos 3444 (left), 3445 (middle), and 3452 Tree trunk stems photographed because possibly compaction of the sediments may be calculated with these flattened and originally round (?) structures.
